# Molecular Hydrogen Generation from Neat Formic Acid Catalyzed by Ruthenium–Cymene α‑Diimine Complexes

**DOI:** 10.1021/acsomega.5c05610

**Published:** 2025-11-13

**Authors:** Cássio R. A. do Prado, Lucas da S. dos Santos, Ellen C. Guimarães, Laís A. Tomaz, Lucas F. Martins, Luciano M. Lião, Leonardo T. Ueno, Valdemiro P. Carvalho-Jr, Alexandre B. de Carvalho, Javier Ellena, Luís R. Dinelli, André L. Bogado

**Affiliations:** † Institute of Exact and Natural Sciences of Pontal, 28119 Federal University of Uberlândia, ICENP − UFU, Ituiutaba, MG 38304-402, Brazil; ‡ Institute of Chemistry, Federal University of Uberlândia, IQ - UFU, Uberlândia, MG 38400-902, Brazil; § Institute of Chemistry, 67824 Federal University of Goiás, IQ - UFG, Goiânia, GO 74690-900, Brazil; ∥ Department of Chemistry, Aeronautics Institute of Technology, General Command for Aerospace Technology, ITA, São José dos Campos, SP 12228-900, Brazil; ⊥ School of Technology and Sciences, São Paulo State University, UNESP, Presidente Prudente, SP 19060-900, Brazil; # São Carlos Institute of Physics, 28133 University of Sao Paulo, IFSC − USP, São Carlos, SP 13566-950, Brazil

## Abstract

A series of half-sandwich ruthenium­(II) complexes bearing substituted α-diimine ligands with the general formula [RuCl­(*p*-cym)­(**N–N** ^*n*^)]­(PF_6_) was synthesized (where ***n*** = **1**–**7**; **N–N** ^**1**^ = N1,N2-*bis*(2,6-dimethylphenyl)­ethane-1,2-diimine, **N–N** ^**2**^ = N1,N2-*bis*(2,4-dimethylphenyl)­ethane-1,2-diimine, **N–N** ^**3**^ = N1,N2-*bis*(2,4,6-trimethylphenyl)­ethane-1,2-diimine, **N–N** ^**4**^ = N1,N2-*bis*[2,6-bis­(propan-2-yl)­phenyl]­ethane-1,2-diimine, **N–N** ^**5**^ = N1,N2-*bis*(4-fluorophenyl)­ethane-1,2-diimine, **N–N** ^**6**^ = N1,N2-*bis*(4-chlorophenyl)­ethane-1,2-diimine and **N–N** ^**7**^ = N1,N2-dicyclohexylethane-1,2-diimine). Ligands and complexes were fully characterized by elemental analysis, NMR, FTIR, UV–vis spectroscopy, and single-crystal X-ray diffraction for **2**, **5,** and **6.CH**_**2**_ **Cl**_**2**_. The complexes displayed a distorted pseudo-octahedral “piano-stool” geometry, with the α-diimine ligands coordinating in a bidentate manner. The *p*-cymene ring was observed to rotate around its bond to the metal, as evidenced by variable-temperature ^1^H NMR spectra and NOE measurements. DFT calculations were used to investigate the electronic structures of complexes **1**–**4**, revealing how different substituents affect their stability and HOMO–LUMO energy gaps. Additionally, the most nucleophilic and electrophilic regions in the optimized structures were identified using the Hirshfeld charge method applied to the Fukui function. All complexes were evaluated as precatalysts in the solvent-free dehydrogenation of formic acid, in the presence of a Bro̷nsted–Lowry base, achieving up to 94.8% conversion in a first run and a maximum turnover frequency (TOF) of 627 h^–1^ under mild conditions (60 °C, 1:1204:843 molar ratio of Ru/FA/base). Total conversion and improvement in TOF values were observed in a subsequent run. A detailed mechanistic study combining kinetic data and DFT modeling supports a chloride displacement–initiated cycle involving a β-hydride elimination pathway for H_2_ and CO_2_ release. This methodology is consistent with the observed induction period and activation parameters (Δ*G* ^‡^ = 24.5 kcal mol^–1^; Δ*S* ^‡^ = +137 cal mol^–1^ K^–1^), and Δ*H* ^‡^ = 70.3 kcal mol^–1^, which are in excellent agreement with *E*_a_ = 70.9 kcal mol^–1^). The catalytic activity was strongly influenced by both the electronic nature and steric hindrance of the α-diimine ligands, as well as the Bro̷nsted–Lowry character of the base.

## Graphical abstract

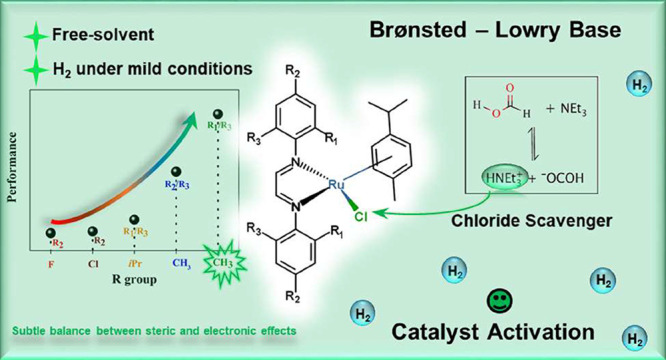

## 1 Introduction

The global pursuit of sustainable and carbon-neutral energy sources has intensified interest in molecular hydrogen (H_2_) as a promising clean fuel.^1–6^ Among the various hydrogen storage and delivery systems under investigation, formic acid (FA) (HCOOH) has emerged as an attractive liquid hydrogen carrier due to its high hydrogen content (4.4 wt%), low toxicity, and ease of handling under ambient conditions.^7–10^ Efficient and selective dehydrogenation of FA into H_2_ and CO, preferably under mild conditions and in the absence of additives or organic solvents, remains a key challenge in the field of hydrogen storage and catalysis.

It was not until 2008 that Beller^11^ and LaurenczAy^12^ independently reported the use of ruthenium complexes as catalysts for the dehydrogenation of F in the absence of CO formation. Two years later, Dupont and co-workers^13^ demonstrated that the dimeric complex [RuCl­(μ-Cl)­(*p*-cym)]_2_, when dissolved in an ionic liquid and employed without any added base, effectively promoted FA decomposition with tiny CO evolution, exhibiting outstanding catalytic performance over successive recycling runs.

In recent times, transition metal complexes, particularly those based on iron,^14–16^ cobalt,^15,17–20^ manganese,^15,21^ nickel,^15,21^ copper,^15,21^ zinc,^15^ aluminum,^21^ rhodium,^19,22^ iridium,^23–26^ osmium^27^ and ruthenium,^16,28–34^ have demonstrated remarkable catalytic activity for FA dehydrogenation, owing to their tunable coordination environments, accessible oxidation states, and robust ligand frameworks. Among these, half-sandwich ruthenium­(II) complexes bearing η^6^-arene ligands have gained considerable attention for their structural versatility and reactivity.^30,31,35–39^ A wide type of ligands, such as substituted-pyridines,^30,31^ amide-phosphine,^35^ bis-imidazole,^36,37^ quinoline,^38^ and *N*,*N*′-diimine,^39^ have been employed to modulate the electronic properties of the metal center and enhance catalytic efficiency. However, there is a growing need to expand the scope of ancillary ligands to fine-tune their catalytic activity through electronic and steric modifications.

In this context, α-diimine ligands stand out as attractive candidates due to their strong σ-donating and π-accepting capabilities as well as their ability to stabilize reactive metal intermediates. The incorporation of sterically and electronically diverse substituents on the aromatic rings of α-diimines offers a powerful strategy to control the reactivity and selectivity of metal-based catalysts. Despite their well-documented coordination chemistry, the use of α-diimine ligands in ruthenium­(II) arene complexes for catalytic hydrogen evolution from FA has been relatively unexplored.

Herein, we report the synthesis and full characterization of a series of [RuCl­(*p*-cymene)­(N–N)]­PF_6_ complexes in which N–N represents a family of α-diimine ligands bearing various substituents with differing electronic and steric properties. The complexes were investigated using a combination of spectroscopic techniques, X-ray crystallography, and density functional theory (DFT) calculations to elucidate their structural and electronic features. Their catalytic performance in the solvent-free dehydrogenation of FA was evaluated under mild conditions, and mechanistic insights were obtained through kinetic studies and theoretical modeling.

## 2 Experimental Section

### 2.1 Materials and Methods

All reactions were carried out under an argon atmosphere using standard Schlenk techniques. Solvents were purchased from Alphatec or Synth and purified by standard methods^40^ and all chemicals used were of reagent grade or comparable purity, which were supplied and used as received from Aldrich: RuCl_3_·*x*H_2_O, FA 98%, glyoxal, α-phellandrene, ammonium hexafluorophosphate, 2,6-dimethylaniline, 2,4-dimethylaniline, 2,4,6-trimethylaniline, 2,6-di*iso*propylaniline, 4-fluoroaniline, 4-chloroaniline, cyclohexylaniline, triethylamine, pyridine, tripropylamine, NaBH_4_, HCOONa, HCOOK, and *tert*-BuOK.

### 2.2 Instrumentation

Elemental analyses were performed with a Thermo Scientific CHNS-O FLASH 2000 micro analyzer, coupled with an ultramicrobalance Mettler Toledo Model XP6.

All NMR experiments were recorded on a Bruker Avance III 500 spectrometer operated at 11.75 T; ^1^H was observed at 500.13 MHz, using a broadband inverse probehead (BBI) at 25^ο^C, in a CDCl_3_ solution. TMS signal at δ 0.00 as an internal reference and the signals were labeled as *s* = singlet, *br* = broad, *d* = doublet, *dd* = double doublet, *ddd* = double double doublet, *dsept* = double septet, *t* = triplet, *td* = triple doublet, *tt* = triple triplet, *tt* = triple triplet, *q* = quartet, *sept* = septet, and *m* = multiplet.

FTIR spectra of the ligands **N–N** ^**1**^ – **N–N** ^**7**^ were recorded on an Agilent spectrophotometer, model Cary 630, in the range 4000–650 cm^–1^. Spectra for the organometallic ruthenium complexes **1**–**7** were acquired over the 4000–200 cm^–1^ range using a PerkinElmer model FT-IR Frontier Single Range – MIR. The samples were measured in the solid state using an ATR apparatus with a diamond cell support.

UV/vis spectra were recorded on a Shimadzu spectrophotometer, model UV-1800, coupled with a thermoelectrically temperature-controlled cell TCC-100 (at 25.0 ± 0.1 °C), using a quartz cell (1 cm) between 200 and 800 nm.

Molar conductivity of solutions of the half-sandwich ruthenium complexes **1**–**7** (10^–3^ mol L^–1^ in CH_2_Cl_2_ or CH_3_CN) was measured at a Mettler Toledo conductivity meter model FE30, using a Pt electrode from Mettler Toledo model inLab 710 (cell constant = 0.55 cm) with sensor temperature coupled.

The gas obtained from the catalytic experiments was analyzed on a Shimadzu GC-2010 Pro gas chromatograph with a thermal conductivity detector (TCD). Data acquisition and processing were performed with LabSolutions Software version 5.111 (Shimadzu Corporation). The column used was a ShinCarbon ST 100/120 mesh type (2.0 m × 1.00 mm i.d., 1.00 μm film thickness, 1/16″ OD, Silco). The WBI injector, oven, and detector (TCD) temperatures were 100, 80, and 200 °C, respectively. Argon was used as the carrier gas, with a total flow of 10.0 mL min^–1^, purge flow of 3.0 mL min^–1^, column flow of 5.0 mL min^–1^, and makeup flow of 2.0 mL min^–1^. The volume of gases (250 μL) was injected with a headspace PAL system syringe of 2.5 mL, gauge 23, and PST 5. On these conditions, the retention times (min.) are H_2_ (0.701), N_2_ (1.289), CO (1.488), and CO_2_ (6.251). MarvinSketch was used for drawing and displaying chemical structures.^41^

### 2.3 General Procedure to Synthesize the α-Diimines

The symmetric α-diimines were prepared as an adapted procedure of the literature,^42^ and a general protocol is described as follows: in a 500 mL round-bottom flask equipped with a magnetic stirring bar, the corresponding substituted aniline in the *ortho* or *para* position (300 mmol), and isopropanol (150 mL) were added. In an Erlenmeyer flask, glyoxal (40% aqueous solution, 150 mmol) was diluted with distilled water (50 mL) and isopropanol (50 mL). The colorless solution of glyoxal was added to the solution containing the substituted aniline in only one portion. The resulting mixture was stirred for 24 h at room temperature, and in general, a yellow-brown precipitate appeared after this period. Then, the suspension was filtered in a Büchner funnel, and the precipitate was washed twice with water (2 × 100 mL), dried, and stored under vacuum.

#### 2.3.1 N1,N2-bis­(2,6-dimethylphenyl)­ethane-1,2-diimine (N–N^1^)

The ligand **N–N** ^**1**^ was obtained as yellow crystals. Molecular weight: 264.372 g mol^–1^. Yield: 57.07% (*m* = 2.2631 g). Elemental analysis calculated for C_18_H_20_N_2_ (%): calc. (exp.): C 81.78 (81.67) %, H 7.63 (7.82) %, N 10.60 (10.31) %. FTIR (ATR; cm^–1^): 3023 (ν_(Csp2‑H)_); 2967 (ν_(Csp3‑H)_); 1618 (ν_(C=N)_); 1473 (ν_(C=C)_). UV/vis (CH_2_Cl_2_, 0.1 μmol L^–1^, 25 °C): λ = nm (log ε, L cm^–1^ mol^–1^); 230 (7.87); 253 (7.55); 356 (7.07). ^1^H NMR (CDCl_3_; 500.13 MHz, δ): 8.12 (*s*, H-1); 7.08 (*d*, *J*_H–H_ = 7.50 Hz; H-4, H-6); 6.99 (*t*, *J*_H–H_ = 7.50 Hz, H-5); 2.18 (*s*, CH_3_-3, CH_3_-7). ^13^C NMR (CDCl_3_; 125.75 MHz, δ): 163.7 (C-1); 150.1 (C-2); 128.5 (C-3, C-7); 126.6 (C-4, C-6); 125.0 (C-5); 18.4 (CH_3_-3, CH_3_-7).

#### 2.3.2 N1,N2-bis­(2,4-dimethylphenyl)­ethane-1,2-diimine (N–N^2^)

The ligand **N–N** ^**2**^ was obtained as yellow crystals. Molecular weight: 264.372 g mol^–1^. Yield: 65.24% (*m* = 2.5870 g). Elemental analysis calculated for C_18_H_20_N_2_ (%): calc. (exp.): C 81.78 (81.88) %, H 7.63 (7.86) %, N 10.60 (10.39) %. FTIR (ATR; cm^–1^): 3008 (ν_(Csp2‑H)_); 2945 (ν_(Csp3‑H)_); 1596 (ν_(C=N)_); 1490 (ν_(C=C)_). UV/vis (CH_2_Cl_2_, 0.1 μmol L^–1^, 25 °C): λ = nm (log ε, L cm^–1^ mol^–1^); 230 (7.28); 249 (7.26); 280 (7.23); 361 (7.31). ^1^H NMR (CDCl_3_; 500.13 MHz, δ): 8.31 (*s*, H-1); 7.07 (*d*, *J*_H–H_ = 2.0 Hz, H-4); 7.02 (*dd*, *J*_H–H_ = 8.1; 2.0 Hz, H-6); 6.94 (*d*, *J*_H–H_ = 8.1 Hz, H-6); 2.37 (*s*, CH_3_-3); 2.33 (*s*, CH_3_-5). ^13^C NMR (CDCl_3_; 125.75 MHz, δ): 159.0 (C-1); 147.0 (C-2); 137.3 (C-5); 133.1 (C-3); 131.4 (C-4); 127.1 (C-6); 117.1 (C-7); 21.0 (CH_3_-3); 17.8 (CH_3_-5).

#### 2.3.3 N1,N2-bis­(2,4,6-trimethylphenyl)­ethane-1,2-diimine (N–N^3^)

The ligand **N–N** ^**3**^ was obtained as yellow crystals. Molecular weight: 292.426 g mol^–1^. Yield: 80.19% (*m* = 3.836 g). Elemental analysis calculated for C_20_H_24_N_2_ (%): calc. (exp.): C 81.78 (82.87) %; H 7.63 (8.43) %; N 10.60 (9.29) %. FTIR (ATR; cm^–1^): 3023 (ν_(Csp2‑H)_); 2915 (ν_(Csp3‑H)_); 1617 (ν_(C=N)_); 1475 (ν_(C=C)_). UV/vis (CH_2_Cl_2_, 0.1 μmol L^–1^, 25 °C): λ = nm (log ε, L cm^–1^ mol^–1^); 230 (7.58); 259 (7.28); 364 (6.89). ^1^H NMR (CDCl_3_; 500.13 MHz, δ): 8.10 (*s*, H-1); 6.91 (*s*, H-4, H-6); 2.29 (*s*, CH_3_-5); 2.16 (*s*, CH_3_-3, CH_3_-7). ^13^C NMR (CDCl_3_; 125.75 MHz, δ): 163.5 (C-1); 147.4 (C-2); 134.2 (C-5); 129.0 (C-3, C-7); 126.5 (C-4); 20.8 (CH_3_-5); 18.2 (CH_3_-3, CH_3_-7).

#### 2.3.4 N1,N2-bis­[2,6-bis­(propan-2-yl)­phenyl]­ethane-1,2-diimine (N–N^4^)

The ligand **N–N** ^**4**^ was obtained as yellow crystals. Molecular weight: 376.588 g mol^–1^. Yield: 63.51% (*m* = 3.9462 g). Elemental analysis calculated for C_26_H_36_N_2_ (%): calc. (exp.): C 81.78 (82.51) %, H 7.63 (8.10) %, N 7.33 (7.44) %. FTIR (ATR; cm^–1^): 3064 (ν_(Csp2‑H)_); 2961 (ν_(Csp3‑H)_); 1626 (ν_(C=N)_); 1460 (ν_(C=C)_). UV/vis (CH_2_Cl_2_, 0.1 μmol L^–1^, 25 °C): λ = nm (log ε, L cm^–1^ mol^–1^); 230 (7.94); 257 (7.51); 361 (6.92). ^1^H NMR (CDCl_3_; 500.13 MHz, δ): 8.10 (*s*, H-1); 7.21–7.13 (*m*, H-4, H-5, H-6); 2.94 (*sept*, *J*_H–H_ = 6.9 Hz, H-3′ H-7′); 1.21 (*d*, *J*_H–H_ = 6.9 Hz, CH_3_-3″, CH_3_-7″). ^13^C NMR (CDCl_3_; 125.75 MHz, δ): 163.1 (C-1); 148.0 (C-2); 136.7 (C-3, C-7); 125.1 (C-5); 123.2 (C-4, C6); 28.0 (C-3′, C-7′); 23.42 (CH_3_-3″, CH_3_-7″).

#### 2.3.5 N1,N2-bis­(4-fluorophenyl)­ethane-1,2-diimine (N–N^5^)

The ligand **N–N** ^**5**^ was obtained as yellow-orange crystals. Molecular weight: 244.24 g mol^–1^. Yield: 89.41% (*m* = 2.012 g). Elemental analysis calculated for C_14_H_10_F_2_N_2_ (%): calc. (exp.): C 68.85 (69.13) %, H 4.13 (4.14) %, N 11.47 (11.28) %. FTIR (ATR; cm^–1^): 3063 (ν_(Csp2‑H)_); 2974 (ν_(Csp3‑H)_); 1612 (ν_(C=N)_); 1502 (ν_(C–F)_); 1366–1305 (ν_(C=C)_). UV/vis (CH_2_Cl_2_, 0.1 μmol L^–1^, 25 °C): λ = nm (log ε, L cm^–1^ mol^–1^); 235 (7.44); 282 (7.22); 340 (6.99). ^1^H NMR (CDCl_3_; 500.13 MHz, δ): 8.36 (*s*, H-1); 7.30 (*dd*_AB_*, *J*_H–H_ = *dd*, 9.0; 5.0 Hz, H-3, H-7, *second-order system); 7.12 (*t*_AB_*, *J*_H–H_ = *dd*, 8.5 Hz, H-4, H-6, *second-order system). ^13^C NMR (CDCl_3_; 125.75 MHz, δ): 162.6 (*J*_CF_ = 248.6 Hz, C-5); 159.5 (*J*_CF_ = 2.1 Hz, C-1); 146.3 (*J*_C–F_ = 3.3 Hz, C-2); 123.3 (*J*_C–F_ = 8.7 Hz, C-3, C-7); 116.5 (*J*_C–F_ = 23.2 Hz, C-4, C-6).

#### 2.3.6 N1,N2-bis­(4-chlorophenyl)­ethane-1,2-diimine (N–N^6^)

The ligand **N–N** ^**6**^ was obtained as yellow crystals. Molecular weight: 277.15 g mol^–1^. Yield: 65.57% (*m* = 4.0751 g). Elemental analysis calculated for C_14_H_10_Cl_2_N_2_ (%): calc. (exp.): C 60.67 (59.44) %, H 3.64 (3.63) %, N 10.11 (9.84) %. FTIR (ATR; cm^–1^): 3088–3026 (ν_(Csp2‑H)_); 2971 (ν_(Csp3‑H)_); 1606 (ν_(C=N)_); 1483 (ν_(C=C)_); 828–807­(ν_(C–Cl)_). UV/vis (CH_2_Cl_2_, 0.1 μmol L^–1^, 25 °C): λ = nm (log ε, L cm^–1^ mol^–1^); 254 (7.24); 289 (7.00). ^1^H NMR (CDCl_3_; 500.13 MHz, δ): 8.35 (*s*, H-1); 7.40 (*d*_AB_*, *J*_H–H_ = *dd*, 8.7 Hz, H-4, H-6, *second-order system); 7.23 (*d*, *J*_H–H_ = *d*_AB_*, *J*_H–H_ = *dd*, 8.7 Hz, H-3, H-7, *second-order system). ^13^C NMR (CDCl_3_; 125.75 MHz, δ): 159.9 (C-1); 148.4 (C-2); 133.8 (C-5); 129.6 (C-4, C-6); 122.6 (C-3, C-7).

#### 2.3.7 N1,N2-dicyclohexylethane-1,2-diimine (N–N^7^)

The ligand **N–N** ^**7**^ was obtained as bright white crystals. Molecular weight: 220.36 g mol^–1^. Yield: 55.78% (*m* = 1.552 g). Elemental analysis calculated for C_14_H_24_N_2_ (%): (exp.): C 76.31 (76.48) %, H 10.98 (10.77) %, N 12.71 (12.56) %. FTIR (ATR; cm^–1^): 2923–2853­(ν_(Csp3‑H)_); 1622 (ν_(C=N)_); 1449 (ν_(C=C)_). UV/vis (CH_2_Cl_2_, 0.1 μmol L^–1^, 25 °C): λ = nm (log ε, L cm^–1^ mol^–1^); 230 (8.02); 262 (7.77). ^1^H NMR (CDCl_3_; 500.13 MHz, δ): 7.94 (s, H-1); 3.16 (*tt*, *J*_H–H_ = 10.6, 4.1 Hz, H-2); 1.81 (*dqui*, *J*_H–H_ = 13.3, 3.6 Hz; H-4, H-6); 1.76–1.69 (*m*, H-3, H-7); 1.57–1.46 (*m*, H-3, H-7); 1.69–1.63 (*m*, H-5); 1.35 (*dtt*, *J*_H–H_ = 13.3, 12.3; 3.4 Hz; H-4, H-6); 1.23 (*dtt*, *J*_H–H_ = 12.6, 12.3; 3.2 Hz; H-5). ^13^C NMR (CDCl_3_; 125.75 MHz, δ): 160.1 (C-1); 69.4 (C-2); 33.9 (C-3, C-7); 25.5 (C-5); 24.6 (C-4, C-6).

### 2.4 Syntheses of the Half-Sandwich Ruthenium­(II) Complexes

The general synthetic route used to synthesize the half-sandwich ruthenium­(II) complexes with the general formula [RuCl­(*p*-cym)­(**N–N** ^*n*^)]­(PF_6_), where *n* = **1**–**7** α-diimines, is described as follows: in a Schlenk tube (100 mL), under an inert atmosphere of argon, the [RuCl­(μ-Cl)­(*p*-cym)]_2_ (0.15 mmol) was dissolved in toluene (10 mL) and the appropriate α-diimine ligand (0.30 mmol) and NH_4_PF_6_ (0.30 mmol) were added after total dissolution of the ruthenium precursor. The resulting solution was kept at 27 °C and magnetically stirred for 24 h. During this period, the color changed, and the mixture was passed through a Celite pad to remove NH_4_Cl. The filtered mixture was collected in a Schlenk flask (100 mL), the solvent was removed under reduced pressure until approximately 1 mL of toluene remained, and the addition of *n*-hexane (5 mL) yielded a solid. The solid was separated by filtration, washed with *n*-hexane (3 × 5 mL), and dried under reduced pressure.

#### 2.4.1 [RuCl­(*p*-cymene)­(N–N^1^)]­PF_6_ (**1**)

The complex **1** was obtained as an orange powder. Molecular weight: 680.078 g mol^–1^. Yield: 66.7% (*m* = 136 mg). Elemental analysis calculated for C_28_H_34_ClN_2_RuPF_6_ (%) (exp.): C 49.45 (49.40) %, H 5.04 (5.23) %, N 4.12 (4.03) %; FTIR (ATR; cm^–1^): 3023 (ν_Csp2‑H_); 2967 (ν_Csp3‑H_); 1575 (ν_C=N_); 1472 (ν_C=C_); 833 (ν_(P–F)_); 465 (ν_(Ru–N)_); 274 (ν_(Ru–Cl)_); UV/vis (CH_2_Cl_2_, 1.79 × 10^–4^ mol L^–1^), λ = nm (log ε, L cm^–1^ mol^–1^): 346 (3.23), 435 (2.99). Ionic molar conductivity at 25 °C (1.0 × 10^–3^ mol L^–1^, Λ_m_: ohm^–1^ cm^2^ mol^–1^): 153.9 (CH_3_CN solution); 20.4 (CH_2_Cl_2_ solution). ^1^H NMR (CDCl_3_, 500.13 MHz, δ): 8.33 (*s*, H-1); 7.10–6.90 (*m*, H-4, H-5, H-6); 5.56, 5.40, 5.38, 5.26 (*d*_AB_*, *J*_H–H_ = 6.0 Hz, H-10, H-11, H-13, H-14, *second-order system); 2.68 (*sept*, *J*_H–H_ = 6.9 Hz, H-15); 2.37 (*s*, CH_3_-3, CH_3_-7); 2.14 (*s*, CH_3_-8); 1.22 (*d*, *J*_H–H_ = 6.9 Hz, CH_3_-16, CH_3_-17). ^13^C NMR (CDCl_3_, 125.75 MHz, δ): 171.3 (C-1); 150.2 (C-2); 130.1 (C-3, C-7); 129.3 (C-4, C-6); 125.4 (C-5); 90.7–78.3 (C-9, C-10, C-11, C-12, C-13, C-14); 31.6 (C-15); 22.3, 22.2, 18.6 (CH_3_-3, CH_3_-7, CH_3_-16, CH_3_-17); 20.6 (CH_3_-8).

#### 2.4.2 [RuCl­(*p*-cymene)­(N–N^2^)]­PF_6_ (**2**)

The complex **2** was obtained as a brown powder. Molecular weight: 680.078 g mol^–1^. Yield: 96.6% (*m* = 197 mg). Elemental analysis calculated for [C_28_H_34_ClN_2_Ru]­PF_6_ (%) (exp.): C 49.45 (49.40) %, H 5.04 (5.23) %, N 4.12 (4.03) %; FTIR (ATR; cm^–1^): 3049–3027 (ν_Csp2‑H_); 2969–2959 (ν_Csp3‑H_); 1608 (ν_C=N_); 1496 (ν_C=C_); 834 (ν_(P–F)_); 449 (ν_(Ru–N)_); 281 (ν_(Ru–Cl)_); UV/vis (CH_2_Cl_2_, 1.42 × 10^–4^ mol L^–1^), λ (nm) (log ε (L cm^–1^ mol^–1^)): 230 (3.99), 286 (3.54), 399 (3.59). Ionic molar conductivity at 25 °C (1.0 × 10^–3^ mol L^–1^, Λ_m_: ohm^–1^ cm^2^ mol^–1^): 155.6 (CH_3_CN solution); 21.5 (CH_2_Cl_2_ solution).^1^H NMR (CDCl_3_, 500.13 MHz, δ): 8.19 (*s*, H-1); 7.68 (*d*, *J*_H–H_ = 8.1 Hz, H-7); 7.13 (*d*, *J*_H–H_ = 1.9 Hz, H-4); 7.08 (*dd*, *J*_H–H_ = 8.1; 1.9 Hz, H-6); 5.17, 5.2 (*d*_AB_*, *J*_H–H_ = 6.7 Hz, H-10, H-11, H-13, H-14, *second-order system); 2.68 (*sept*, *J*_H–H_ = 6.9 Hz, H-15); 2.37, 2.35 (*s*, CH_3_-3, CH_3_-5); 2.00 (*s*, CH_3_-8); 1.03 (*d*, *J*_H–H_ = 6.9 Hz, CH_3_-16, CH_3_-17). RMN ^13^C (CDCl_3_, 125.75 MHz, δ): 167.8 (C-1); 149.5 (C-2); 140.3 (C-5); 132.7 (C-3); 127.7 (C-4); 123.8 (C-6); 120.4 (C-7); 82.9–78.3 (C-9, C-10, C-11, C-12, C-13, C-14); 31.6–31.0 (C-15); 22.3 (CH_3_-3); 22.2 (CH_3_-5); 21.3 (C-8); 18.8, 18.3 (CH_3_-16, CH_3_-17). Suitable crystals of **2** grew up by slow diffusion of a dichloromethane-hexamethyldisiloxane solution at low temperature (−8 °C), and the structure was determined by X-ray analysis.

#### 2.4.3 [RuCl­(*p*-cymene)­(N–N^3^)]­PF_6_ (**3**)

The complex **3** was obtained as an orange powder. Molecular weight: 708.132 g mol^–1^. Yield: 76.0% (*m* = 172 mg). Elemental analysis calculated for [C_30_H_38_ClN_2_Ru]­PF_6_ (%) (exp.): C 50.88 (49.91) %, H 5.41 (5.08) %, N 3.96 (3.94) %; FTIR (ATR; cm^–1^): 3043 (ν_Csp2‑H_); 2964 (ν_Csp3‑H_); 1606 (ν_C=N_); 1473–1442 (ν_C=C_); 831 (ν_(P–F)_); 439 (ν_(Ru–N)_); 275 (ν_(Ru–Cl)_); UV/vis (CH_2_Cl_2_, 1.38 × 10^–4^ mol L^–1^), λ (nm) (log ε (L cm^–1^ mol^–1^)): 281 (4.07), 364 (3.60), 436 (3.67). Ionic molar conductivity at 25 °C (1.0 × 10^–3^ mol L^–1^, Λ_m_: ohm^–1^ cm^2^ mol^–1^): 160.1 (CH_3_CN solution); 22.4 (CH_2_Cl_2_ solution). ^1^H NMR (CDCl_3_, 500.13 MHz, δ): 8.26 (*s*, H-1); 6.98 (*s*, H-6); 6.95 (*s*, H-4); 5.55, 5.42, 5.38, 5.23 (*d*_AB_*, *J*_H–H_ = 6.1 Hz, H-10, H-11, H-13, H-14, *second-order system); 2.68 (*sept*, *J*_H–H_ = 6.9 Hz, H-15); 2.30, 2.29 (*s*, CH_3_-3, CH_3_-7); 2.08 (*s*, CH_3_-5); 1.28 (*s*, CH_3_-8); 0.95 (*d*, *J*_H–H_ = 6.9 Hz, CH_3_-16, CH_3_-17). RMN ^13^C (CDCl_3_, 125.75 MHz, δ): 171.1 (C-1); 148.3 (C-2); 139.2 (C-5); 130.6 (C-4); 130.4 (C-3); 129.8 (C-6); 129.2 (C-7); 113.6, 109.0 (C-9, C-12); 90.1, 87.6, 79.0, 78.3 (C-10, C-11, C-13, C-14); 22.0 (CH_3_-16, CH_3_-17); 21.0 (CH_3_-3, CH_3_-7); 18.7 (CH_3_-5); 16.6 (CH_3_-8).

#### 2.4.4 [RuCl­(*p*-cymene)­(N–N^4^)]­PF_6_ (**4**)

The complex **4** was obtained as a red powder. Molecular weight: 792.294 g mol^–1^. Yield: 57.0% (*m* = 135 mg). Elemental analysis calculated for [C_36_H_50_ClN_2_Ru]­PF_6_ (%) (exp.): C 45.48 (44.99) %, H 4.83 (5.38) %, N 3.54 (3.99) %; FTIR (ATR; cm^–1^): 3063 (ν_Csp2‑H_); 2947 (ν_Csp3‑H_); 1583 (ν_C=N_); 1440 (ν_C=C_); 838 (ν_(P–F)_); 479 (ν_(Ru–N)_); 306 (ν_(Ru–Cl)_); UV/vis (CH_2_Cl_2_, 8.38 × 10^–5^ mol L^–1^), λ (nm) (log ε (L cm^–1^ mol^–1^)): 230 (4.31), 344 (3.44), 488 (3.6). Ionic molar conductivity at 25 °C (1.0 × 10^–3^ mol L^–1^, Λ_m_: ohm^–1^ cm^2^ mol^–1^): 163.2 (CH_3_CN solution); 25.2 (CH_2_Cl_2_ solution). ^1^H NMR (CDCl_3_, 500.13 MHz, δ): 8.51 (*s*, H-1); 7.33 (*t*, *J*_H–H_ = 7.6 Hz, H-5); 7.28, 7.08 (*dd*, *J*_H–H_ = 7.5; 1.5 Hz, H-4, H-6); 5.42, 5.26 (*d*_AB_*, *J*_H–H_ = 5.5 Hz, H-10, H-11, H-13, H-14, *second-order system); 4.17, 2.08 (*sept*, *J*_H–H_ = 6.5 Hz, H-3′, H-7′); 2.71 (*sept*, *J*_H–H_ = 6.9 Hz, H-15); 2.21 (*s*, CH_3_-8); 1.37, 1.11, 1.09, 1.01 (*d*, *J*_H–H_ = 6.5 Hz, CH_3_-3″, CH_3_-7″); 1.16 (*d*, *J*_H–H_ = 6.9 Hz, CH_3_-16, CH_3_-17). ^13^C NMR (CDCl_3_, 125.75 MHz, δ): 165.8 (C-1); 150.0 (C-2); 144.4, 141.9 (C-3, C-7); 127.6 (C-5); 124.2, 123.0 (C-4, C-6); 100.1 (C-12); 96.4 (C-9); 79.2, 77,9 (C-10, C-11, C-13, C-14); 31.3 (C-15); 28.5, 27.7 (CH_3_-3′, CH_3_-7′); 26.9, 26.4, 23.6, 23.4 (C H_3_-3″, C H_3_-7″); 22.4 (CH_3_-16, CH_3_-17); 18.9 (CH_3_-8).

#### 2.4.5 [RuCl­(*p*-cymene)­(N–N^5^)]­PF_6_ (**5**)

The complex **5** was obtained as a brown powder. Molecular weight: 659.946 g mol^–1^. Yield: 80.0% (*m* = 215 mg). Elemental analysis calculated for [C_36_H_34_ClN_2_Ru]­PF_6_ (%) (exp.): 43.68 (43.29) %, H 3.67 (3.59) %, N 4.24 (4.28) %; FTIR (ATR; cm^–1^): 3145–3050 (ν_Csp2‑H_); 2970 (ν_Csp3‑H_); 1600 (ν_C=N_); 1509 (ν_C=C_); 1405 (ν_C–F_); 829 (ν_(P–F)_); 557 (ν_(Ru–N)_); 301 (ν_(Ru–Cl)_). UV/vis (CH_2_Cl_2_, 1.38 × 10^–4^ mol L^–1^), λ (nm) (log ε (L cm^–1^ mol^–1^)): 285 (3.82), 358 (3.76), 441 (3.43). Ionic molar conductivity at 25 °C (1.0 × 10^–3^ mol L^–1^, Λ_m_: ohm^–1^ cm^2^ mol^–1^): 130.7 (CH_3_CN solution); 9.3 (CH_2_Cl_2_ solution). ^1^H NMR (CDCl_3_; 500.13 MHz, δ): 8.26 (*s*, H-1); 7.85 (*dd*_AB_*, *J*_H–H_ = *dd*, 8.9; 4.7 Hz, H-3, H-7, *second-order system); 7.26 (*dd*_AB_*, *J*_H–H_ = *dd*, 8.9; 8.0 Hz, H-4, H-6, *second-order system); 5.21, 4.94 (*d*_AB_*, *J*_H–H_ = 5.5 and 5.9 Hz, H-10, H-11, H-13, H-14, *second-order system); 2.67 (*sept*, *J*_H–H_ = 6.9 Hz, H-15); 2.09 (*s*, CH_3_-8); 1.17 (*d*, *J*_H–H_ = 6.9 Hz, CH_3_-16, CH_3_-17). ^13^C NMR (CDCl_3_; 125.75 MHz, δ): 165.4 (C-1); 163.9 (*J*_CF_ = 252.8 Hz, C-5); 148.8 (*J*_C–F_ = 2.8 Hz, C-2); 124.4 (*J*_C–F_ = 8.9 Hz, C-3, C-7); 117.1 (*J*_C–F_ = 23.2 Hz, C-4, C-6); 88.4, 81.7 (C-10, C-11, C-13, C-14); 111.3 (C-12); 103.9 (C-9); 29.9 (C-15); 22.2 (C-16, C-17); 18.4 (C-8). Suitable crystals of **5** grew up by slow diffusion of a dichloromethane-hexamethyldisiloxane solution at low temperature (−8 °C), and the structure was determined by X-ray analysis.

#### 2.4.6 [RuCl­(*p*-cymene)­(N–N^6^)]­PF_6_ (**6**)

The complex **6** was obtained as a brown powder. Molecular weight: 702.933 g mol^–1^. Yield: 79% (*m* = 189 mg). Elemental analysis calculated for [C_24_H_34_Cl_3_N_2_Ru]­PF_6_ (%) (exp.): C 41.01 (40.95) %, H 4.88 (4.72) %, N 3.99 (4.01) %; FTIR (ATR; cm^–1^): 3104–3055 (ν_Csp2‑H_); 2962 (ν_Csp3‑H_); 1596 (ν_C=N_); 1405 (ν_C=C_); 830 (ν_(P–F)_); 651 (ν_C–Cl_); 557 (ν_(Ru–N)_); 290 (ν_(Ru–Cl)_). UV/vis (CH_2_Cl_2_, 1.38 × 10^–4^ mol L^–1^), λ (nm) (log ε (L cm^–1^ mol^–1^)): 304 (3.80); 375 (3.82); 465 (3.41). Ionic molar conductivity at 25 °C (1.0 × 10^–3^ mol L^–1^, Λ_m_: ohm^–1^ cm^2^ mol^–1^): 134.4 (CH_3_CN solution); 7.8 (CH_2_Cl_2_ solution). ^1^H NMR (CDCl_3_, 500.13 MHz, δ): 8.27 (*s*, H-1); 7.78 (*d*_AB_*, *J*_H–H_ = 8.6 Hz, H-4, H-6, *second-order system); 7.54 (*d*_AB_*, *J*_H–H_ = 8.6 Hz, H-3, H-7, *second-order system); 5.05, 4.94 (*d*_AB_*, *J*_H–H_ = 5.4 Hz, H-10, H-11, H-13, H-14, *second-order system); 2.84 (*sept*, *J*_H–H_ = 6.9 Hz, H-15); 2.12 (*s*, CH_3_-8); 1.22 (*d*, *J*_H–H_ = 6.9 Hz, CH_3_-16, CH_3_-17). ^13^C NMR (CDCl_3_, 125.75 MHz, δ): 165.5 (C-1); 150.8 (C-2); 137.0 (C-5); 129.6 (C-3, C-7); 123.5 (C-4, C-6); 103.7 (C-12); 95.9 (C-9); 81.6, 79.5 (C-10, C-11, C-13, C-14); 30.6 (C-15); 22.0 (C-16, C-17); 18.6 (C-8). Suitable crystals of **6.CH**_**2**_ **Cl**_**2**_ grew up by slow diffusion of a dichloromethane-hexamethyldisiloxane solution at low temperature (−8 °C), and the structure was determined by X-ray analysis.

#### 2.4.7 [RuCl­(*p*-cymene)­(N–N^7^)]­PF_6_ (**7**)

The complex **7** was obtained as a brown powder. Molecular weight: 637.067 g mol^–1^. Yield: 67.0% (*m* = 169 mg). Elemental analysis calculated for [C_24_H_38_ClN_2_Ru]­PF_6_ (%) (exp.): C 45.32 (44.98) %, H 6.02 (5.99) %, N 4.40 (4.25) %; FTIR (ATR; cm^–1^): 2923–2853 (ν_Csp3‑H_); 1616 (ν_C=N_); 1449 (ν_C=C_); 829 (ν_P–F_). UV/vis (CH_2_Cl_2_, 1.38 × 10^–4^ mol L^–1^), λ (nm) (log ε (L cm^–1^ mol^–1^)): 231 (4.21); 288 (3.68); 369 (3.50); 430 (3.61). Ionic molar conductivity at 25 °C (1.0 × 10^–3^ mol L^–1^, Λ_m_: ohm^–1^ cm^2^ mol^–1^): 75.3 (CH_3_CN solution); 16.1 (CH_2_Cl_2_ solution). ^1^H NMR (CDCl_3_, 500.13 MHz, δ): 88.18 (*s*, H-1); 5.78 (*d*_AB_*, *J*_H–H_ = 6.0 Hz, H-11, H-13, *second-order system); 5.61 (*d*_AB_*, *J*_H–H_ = 6.0 Hz, H-10, H-14, *second-order system); 4.31 (*tt*, *J*_H–H_ = 11.6, 3.2 Hz, H-2); 2.81 (*sept*, *J*_H–H_ = 6.9 Hz, H-15); 2.27 (*s*, CH_3_-8); 2.53, 2.35 (*brd*, *J*_H–H_ = 12.8, 11.9 Hz, H-3, H-7); 1.36–1.10, 1.72–1.62 (*m*, H-3, H-7); 1.99, 1.90 (*brd*, *J*_H–H_ = 13.2 Hz, H-4, H-6); 1.60–1.49, 1.49–1.39 (*m*, H-4, H-6); 1.77 (*brd*, *J*_H–H_ = 13.2 Hz, H-5); 1.21 (*d*, *J*_H–H_ = 6.9 Hz, CH_3_-16, CH_3_-17). ^13^C NMR (CDCl_3_, 125.75 MHz, δ): 163.3 (C-1); 109.4 (C-9); 104.5 (C-12); 87.4 (C-11, C-13); 86.9 (C-10, C-14); 76.2 (C-2); 35.4, 33.3 (C-3, C-7); 31.8 (C-15); 26.0, 25.6 (C-4, C-6); 25.4 (C-5); 22.4 (C-16, C-17); 19.1­(C-8).

### 2.5 Catalytic Experiments

The dehydrogenation reaction of FA was carried out using a similar method previously published by Treigerman and Sasson.^43^ In a 50 mL round-bottom flask, triethylamine (2.0 mL; 14 mmol) and the synthesized organometallic ruthenium complexes **1**–**7** (16.61 μmol) were added. The system was degassed with argon for 15 min and heated to 60 °C, then FA (0.75 mL; 20 mmol) was added. The evolved gas was collected and quantified via cannula into a graduated water column at 25.0 °C. The maximum volume of gas (H_2_ + CO_2_) in each run, considering 100% of conversion, was 0.896 L. The gas composition was analyzed by gas chromatography using a Shimadzu GC-2010 Pro Gas Chromatograph with a TCD detector and calibrated for H_2_, N_2_, CO, and CO_2_.

### 2.6 Theoretical Calculations

All structures were optimized with the DFT method based on the B3LYP functional.^44^ The basis set employed was the Los Alamos effective core potential and double-ζ valence basis set (LanL2DZ)^45^ for ruthenium and 6-31+G­(d)^46^ for *p*-cymene and 6-31G­(d)^47^ for the remaining atoms in dichloromethane. UV/vis spectra were simulated by using the time-dependent DFT approach (TD-DFT) with the same functional and basis set. The Natural Transition Orbitals (NTO), which are useful in the analysis of electronic transitions, and the Fukui functions, which are used to identify the reactive sites of a molecule, were obtained using the Multiwfn program (a Multifunctional Wave function Analyzer).^48^ All calculations were obtained using the software package Gaussian 16, Revision C.02.^49^

### 2.7 X-Ray Diffraction Data

X-ray diffraction data were collected at 100.00(2) K on a Rigaku XtaLAB Synergy-S Dualflex diffractometer equipped with a HyPix 6000HE detector using Cu Kα radiation (1.54184 Å). CrysAlisPro was used for data collection and reduction, cell refinement, and absorption correction.^50^ The solution of the structures was performed using the Intrinsic Phasing method from the SHELXT-2018/2 program,^51^ while the refinement of the non-hydrogen atoms was conducted using the least-squares full matrix on F^2^ using the SHELXL-2019/2 program,^52^ with both programs hosted on Olex2.^53^ Non-hydrogen atoms were refined by considering anisotropic displacement parameters, while the hydrogen atoms were refined isotropically at idealized positions using the riding model. Structures **2**, **5**, and **6.CH**_**2**_ **Cl**_**2**_ were deposited at the Cambridge Structural Database under CCDC numbers 2450155, 2450156, and 245157. An ORTEP view^54^ of each complex is available in Figure 4. Table 1 summarizes the data collection and experimental details for **2**, **5,** and **6**. **CH**_**2**_ **Cl**_**2**_, respectively.

**Table 1:** Crystallographic Data and Structure Refinement Parameters of Complexes **2**, **5**, and **6.CH**_**2**_ **Cl**_**2**_

**complex**	**2**	**5**	**6.CH**_**2**_ **Cl**_**2**_
formula	C_28_H_34_ClF_6_N_2_PRu	C_24_H_24_ClF_8_N_2_PRu	C_49_H_50_Cl_8_F_12_N_4_P_2_Ru_2_
*D*_calc*.*_/ g cm^–3^	1.578	1.738	1.722
μ/mm^–1^	6.357	7.313	9.021
formula weight	181.35	659.94	1468.59
color	red	yellow	red
shape	prism	block	block
size/mm^3^	0.01 × 0.07 × 0.08	0.17 × 0.17 × 0.10	0.015 × 0.01 × 0.01
crystal System	monoclinic	monoclinic	monoclinic
space group	*P*2_1_/*c*	*P*2_1_/*c*	*I*2/*a*
*a*/Å	15.08171(9)	10.42187(11)	16.6760(2)
*b*/Å	11.97569(8)	20.3425(2)	15.4841(2)
*c*/Å	15.87022(11)	12.84344(15)	22.2792(3)
α/°	90	90	90
β/°	92.9253(6)	112.1083(13)	99.9480(10)
γ/°	90	90	90
*V*/Å^3^	2862.65(3)	2522.69(5)	5666.28(13)
*Z*	4	4	4
*Z*′	1	1	0.5
Θ_min_/°	4.628	4.579	5.283
Θ_max_/°	74.493	74.498	74.469
measured refl.	38,086	30,268	28,751
independent refl.	5834	5151	5781
reflections with *I* > 2(*I*)	5564	4756	5307
*R*_int_	0.0369	0.0436	0.0434
parameters	359	337	288
GooF	1.059	1.076	1.054
*wR*_*2*_ (all data)	0.0552	0.0737	0.0898
*wR*_*2*_	0.0543	0.0721	0.0874
*R*_*1*_ (all data)	0.0231	0.0297	0.0385
*R*_*1*_	0.0219	0.0272	0.0352
largest peak	0.596	0.612	0.634
deepest hole	–0.481	–0.819	–0.698

## 3 Results and Discussion

### 3.1 Synthesis and Structural Characterization

The α-diimines, or diazadiene, are common precursors for imidazolium salts,^42^ and they can be obtained with high purity by condensation reaction between glyoxal (40% aqueous solution) and 2.0 eq. of the corresponding substituted aniline in isopropanol/and distilled water (1/1).^55^ The **N–N** ^**1**^ - **N–N** ^**7**^ α-diimine ligands were synthesized and characterized prior to use, including elemental analysis (Table S.1), UV/vis spectroscopy (Table S.2, Figures S.1–S.7), FTIR/ATR spectroscopy (Table S.3, Figures S.8–S.14) and NMR spectra (^1^H and ^13^C for all, ^1^H–^13^C HSQC and HMBC for **N–N** ^**5**^, Figures S.15–S.30). Table 2 summarizes the reaction route, structure, and labeling of them.

**Table 2:**
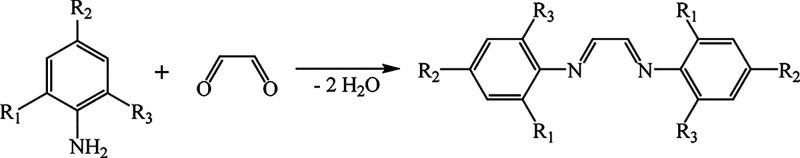
General Route, Structure, and Labeling of the Synthesized α-diimines

	functional group in the aromatic ring		
N–N–ligand	R_1_	R_2_	R_3_
**N–N** ^**1**^	CH_3_	H	CH_3_
**N–N** ^**2**^	CH_3_	CH_3_	H
**N–N** ^**3**^	CH_3_	CH_3_	CH_3_
**N–N** ^**4**^	*i*Pr	H	*i*Pr
**N–N** ^**5**^	H	F	H
**N–N** ^**6**^	H	Cl	H
**N–N** ^**7**^ ^a^	H_2_	H_2_	H_2_

a N1,N2-dicyclohexylethane-1,2-diimine.

The half-sandwich ruthenium­(II) complexes with **N–N** ^**1**^ – **N–N** ^**7**^ ligands were obtained from the [RuCl­(μ-Cl)­(*p*-cym)]_2_ ^56^ as precursor. The chloride bridge between the metal centers was broken in the presence of these ligands, in toluene solution containing PF_6_^–^ salt, producing two equivalents of mononuclear complexes with the general formula [RuCl­(*p*-cym)­(N–N*^n^*)]­(PF_6_) {*n* = **1**–**7**}. Elemental analysis percentage (C, H, N) agrees with the proposed formulation for the complexes from **1** to **7** (Table S.4), and the molar conductivity measurements agree with an electrolyte 1:1 in CH_2_Cl_2_ or CH_3_CN solutions (Table S.5). Scheme 1 contains the route and general structure of the half-sandwich ruthenium complexes reported here. All complexes are air stable and soluble in polar solvents such as dichloromethane, acetone, or acetonitrile and insoluble in apolar solvents, such as hexane, diethyl ether, or hexamethyldisiloxane (HMDSO).

**Scheme 1:**
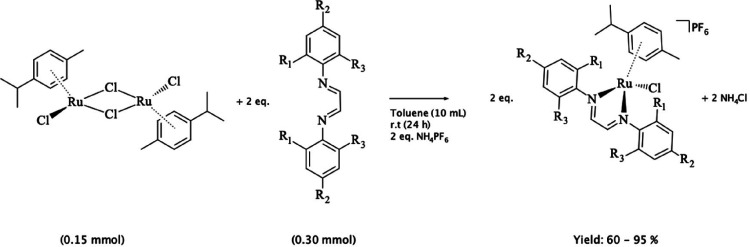
Synthetic Route, General Structure of the Half-Sandwich Ruthenium Complexes **1**–**7**

The spectral features of complexes **1**–**7** in the range between 200–800 nm present typical MLCT absorption maxima as broad transition at around 399–488 nm, due to the coordination of the synthesized α-diimine ligands (Table S.6, Figures S.31–S.37). This lowest absorption feature is well-known for ruthenium complexes bearing commercial aromatic diimines, such as 2,2′-bypiridine^57,58^ or 1.10-phenantroline.^59^ The shortest wavelength absorption maxima at around 230–375 nm presumably arise from the π-conjugated system of the aromatic rings in the *p*-cymene and α-diimine ligands, except for **N–N** ^**7**^, which is attributed only to the *p*-cymene ring.

The coordination of the synthesized α-diimine ligands was confirmed by infrared spectroscopy data (Table S.7, Figures S.38–S.44) and ^1^H, ^13^C NMR spectra for all, ^1^H-^13^C HSQC and HMBC for **2**, **5**, and **7** (Figures S.45–S.64). Table 3 summarizes the main results by comparing the FTIR, ^1^H and ^13^C NMR data of the free ligands with the complexes **1**–**7**.

**Table 3:** Selected IR Stretching, ^1^H and ^13^C NMR Chemical Shifts of the α-Diimine Ligands and Complexes **1**–**7**

		**IR stretching (cm** ^**–1**^ **)**	^**1**^ **H NMR (ppm)**	^**13**^ **C NMR (ppm)**
**free ligand**	**complex**	ν_C= N imine_	HC=N_imine_	HC=N_imine_
NN^1^		1618	8.12	163.7
	**1**	1575	8.33	171.3
NN^2^		1596	8.31	159.0
	**2**	1609	8.19	167.8
NN^3^		1617	8.10	163.5
	**3**	1606	8.26	171.1
NN^4^		1626	8.10	163.1
	**4**	1583	8.51	165.8
NN^5^		1612	8.36	162.6
	**5**	1600	8.26	165.4
NN^6^		1602	8.35	159.9
	**6**	1596	8.27	165.5
NN^7^		1622	7.94	160.1
	**7**	1616	8.18	163.1

The ^1^H NMR spectra of complexes **1**–**7** show a singlet signal in the range 8.18–8.51 ppm for the hydrogen nucleus attributed to the azomethine group (HC=N; H-1), which is slightly downfield-shifted compared to the same hydrogen signal in the free ligand (7.94–8.35 ppm). The ^13^C NMR spectra showed that coordination also affects the chemical shift of the methine group of imine ligands observed in the range 159.0–163.7 ppm, for the free ligand, while for the complexes, the range is shifted to 163.1–171.3 ppm.

The infrared spectra of complexes **1**–**7** further corroborated the coordination of the α-diimine ligands; the ν_C=Nimine_ stretching frequencies are shifted from the range 1626–1602 cm^–1^ for the ligands to 1609–1575 cm^–1^ after coordination. These shifts are due to the electron-withdrawing nature of the α-diimine ligands, which allows the metal-to-ligand (M→L) back-bonding interaction, causing the weakening of the C=N bond. As a consequence, the ν_C=Nimine_ stretching frequencies are shifted to lower wavenumbers, and the intensity of the bands decreases in the ruthenium complexes, which indicates the loss of freedom upon coordination of the α-diimine ligands.

The ^1^H NMR spectrum of complex **5** was recorded over a temperature range from −18 to 30 °C (Figure 1). As the temperature decreases, the septet signal on the far-left shifts toward a lower chemical shift region, merging with another nearby signal. This behavior indicates that the *p*-cymene ring undergoes rotation motion around its bond to the metal, providing different species, labeled herein as rotomers. As the temperature decreases, this rotational motion becomes more restricted, leading to a reduced number of observable signals in the spectrum, due to the slower transition between rotomers.

**Figure 1:**
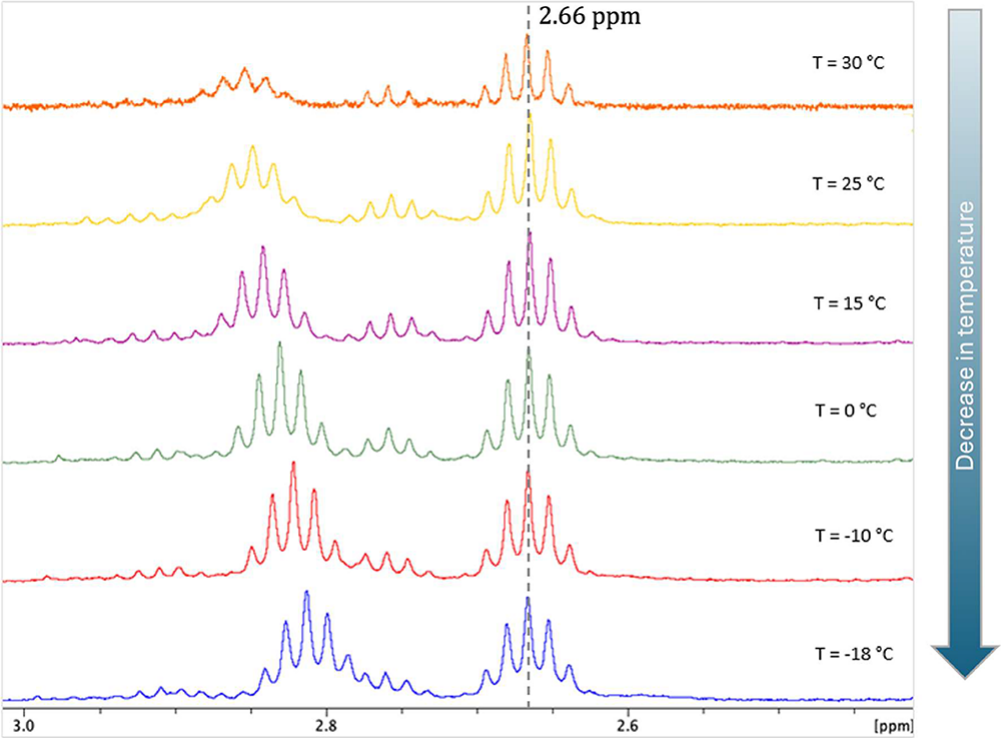
Cutout of the 2.4–3.0 ppm region of ^1^H NMR spectra for complex **5**, performed at different temperatures. (500.13 MHz, CDCl_3_).

NOE spectroscopic measurements were performed for complex **5**, targeting the signals corresponding to the septets of the isopropyl and methyl groups of the *p*-cymene ligand. The set of obtained spectra is presented in Figures 2 and 3. It is observed that the three septet signals exhibited distinct irradiation transfer patterns. The septet at the highest chemical shift (in red) transferred irradiation to the first doublet located above 5.0 ppm, while for the central septet (in green), the most distant doublet received the irradiation. The septet at the lowest chemical shift (in purple) transferred irradiation to the doublet centered at 5.3 ppm.

**Figure 2:**
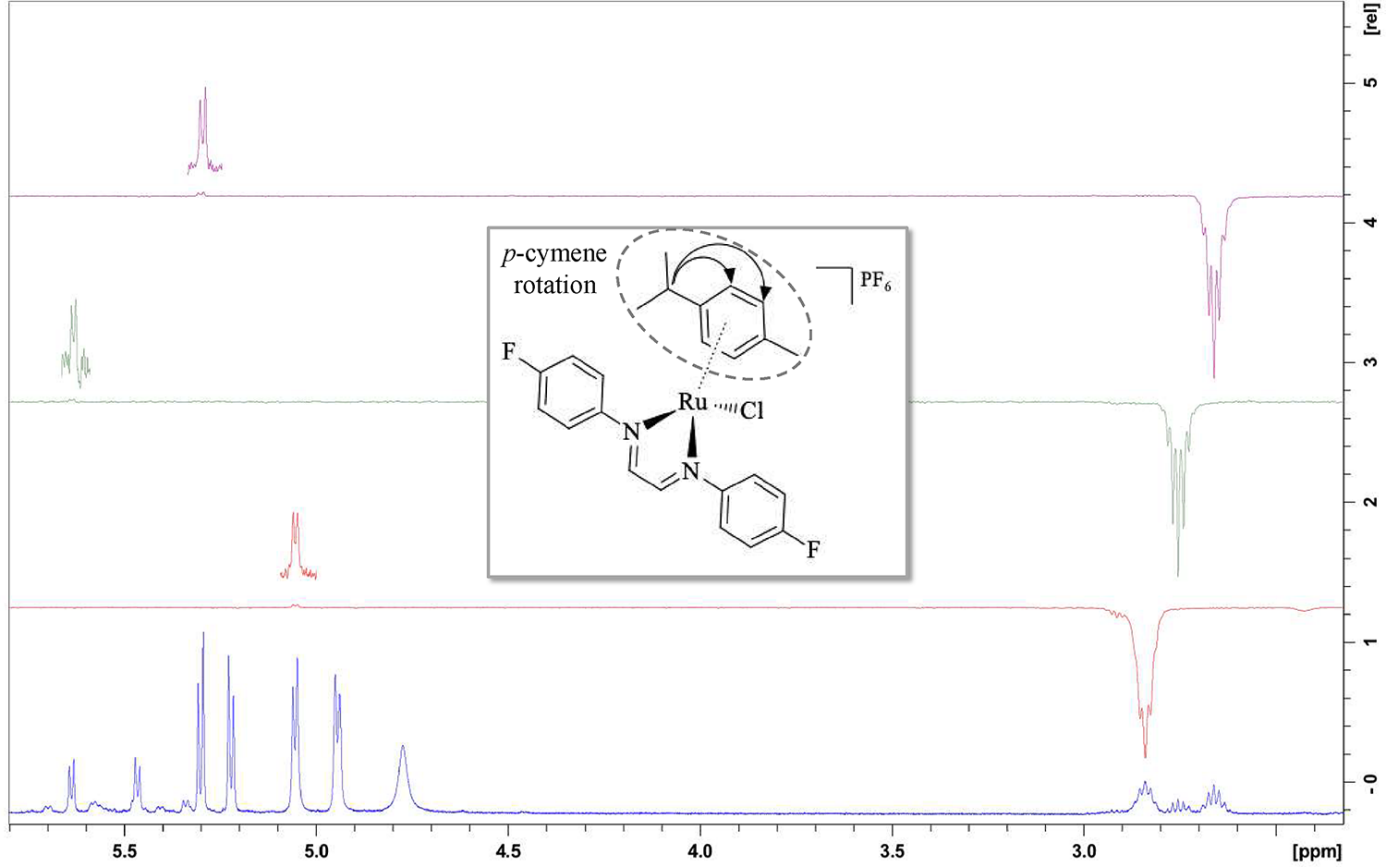
NOE spectrum for complex **5**, with irradiations in the isopropyl septets.

**Figure 3:**
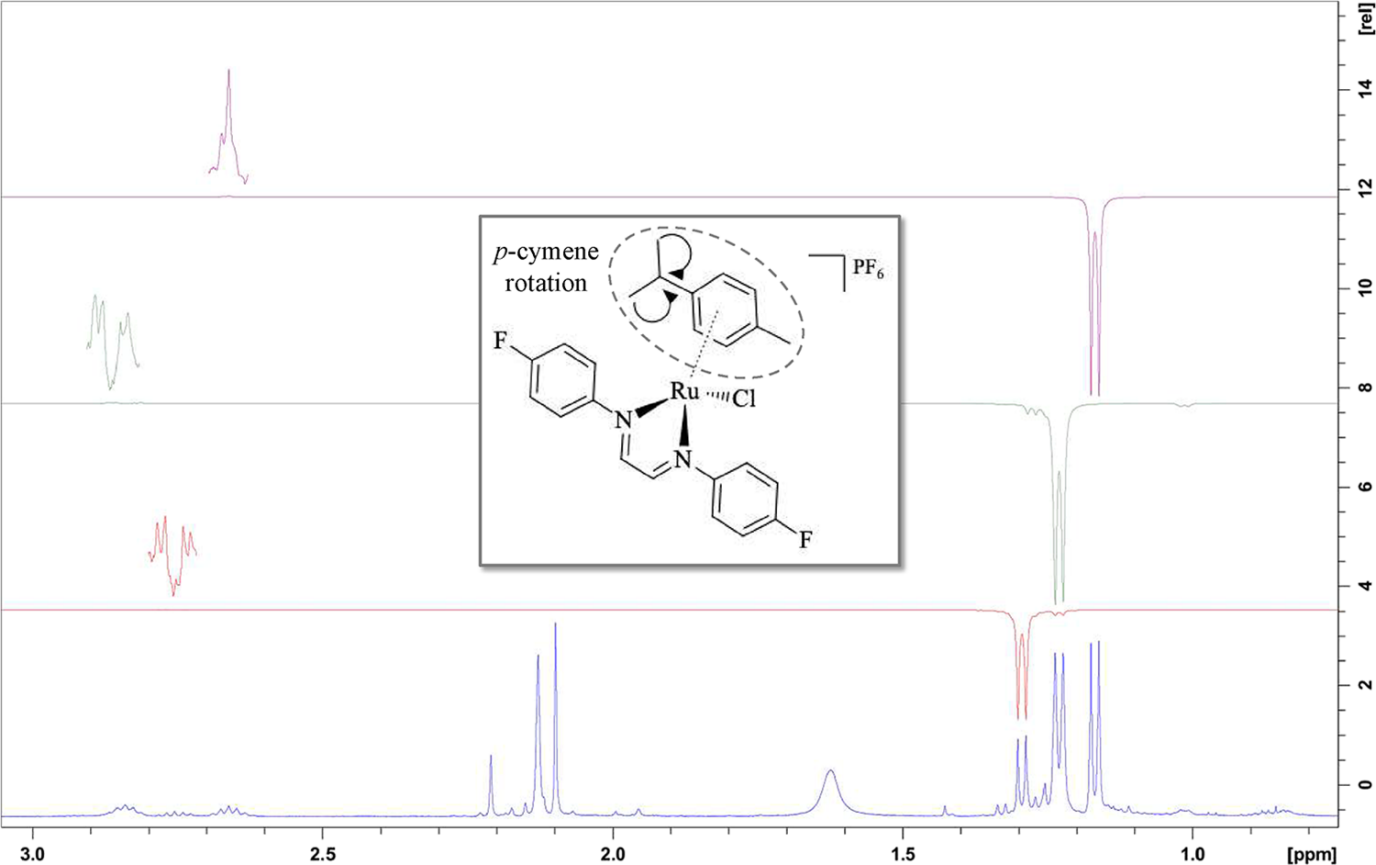
NOE spectrum for complex **5**, with irradiations in the methyl doublets of the isopropyl group of the *p*-cymene ligand.

This same behavior was also observed when the irradiation targets were the methyl doublets of the *p*-cymene ligand, located in the 1.16–1.30 ppm region (Figure 3). The doublet signal at the lowest chemical shift at 1.16 ppm transferred irradiation to the first septet at 2.66 ppm (in purple), the central doublet at 1.22 ppm transferred irradiation to the septet with the highest chemical shift at 2.84 ppm (in green), and the doublet at 1.29 ppm (in red) transferred irradiation to the septet located at 2.76 ppm, as illustrated in Figure 3.

Suitable crystals of the complexes **2**, **5**, and **6.CH**_**2**_ **Cl**_**2**_ grew up by slow diffusion of a dichloromethane-hexamethyldisiloxane solution of each complex at low temperature (−8 °C), and the structures were determined by X-ray analysis. Figure 4 depicts the X-ray structure of **2,** **5**, and **6**, respectively. The CH_2_Cl_2_ molecule in **6** was omitted for clarity. Details about these structures are available in the X-ray data section in the Supporting Information (Tables S.8–S.26).

**Figure 4:**
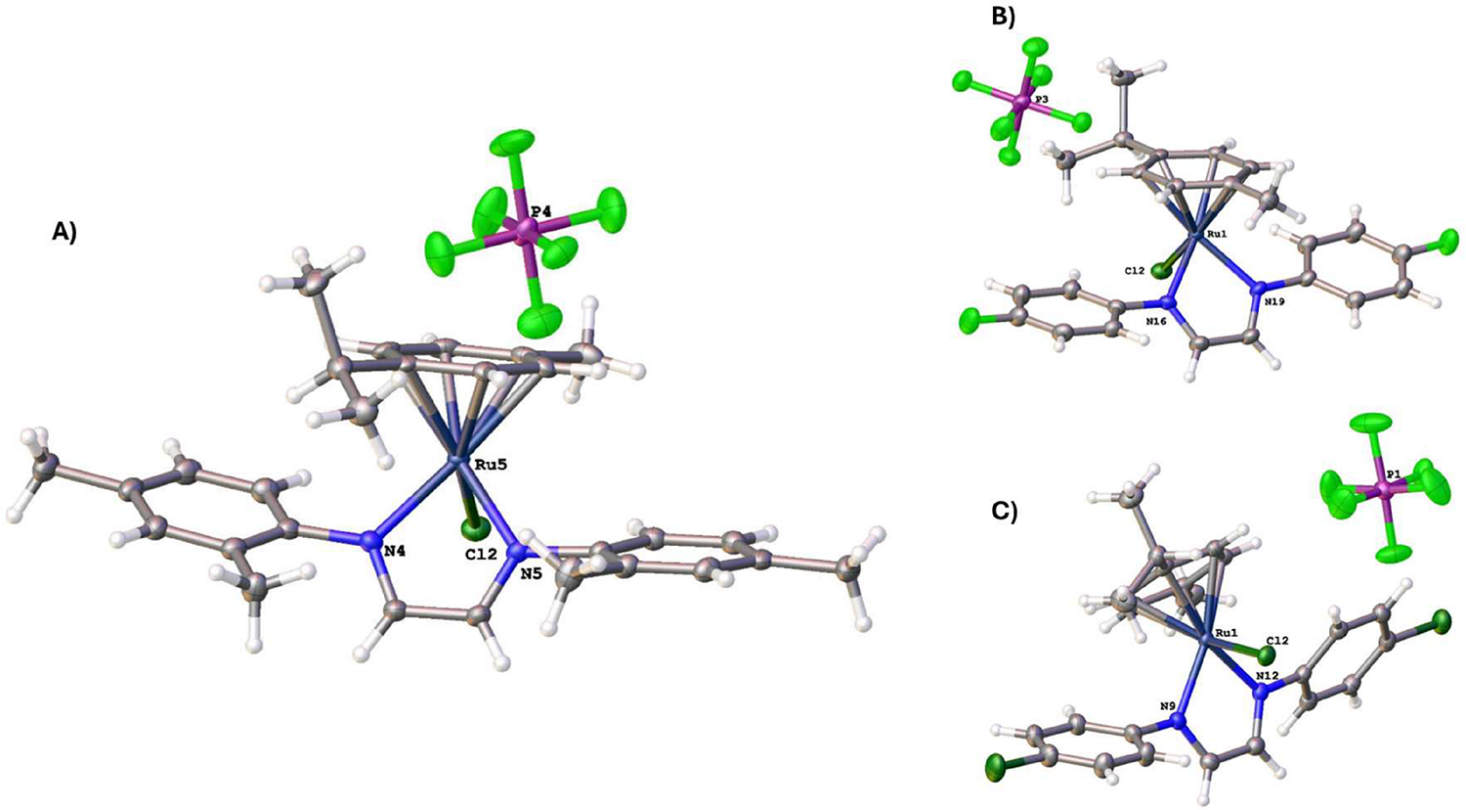
ORTEP-type view of the asymmetric unit showing the selected atom labeling and 50% probability of ellipsoids. (A) complex **2**, (B) complex **5**, and (C) complex **6**, where the CH_2_Cl_2_ molecule was omitted for clarity.

The crystallographic structures of **2**, **5,** and **6.CH**_**2**_ **Cl**_**2**_ reveals distinct characteristics associated with its respective space groups: *P*2_1_/*c* and *I*2/*a* (Table 1). Complex **2** crystallizes in the *P*2_1_/*c* space group, exhibiting a monoclinic unit cell with lattice parameters of *a* = 15.0817(1) Å, *b* = 11.9757(1) Å, *c* = 15.8702(1) Å, and β = 92.9253(6)°. The Ru–Cl bond length is 2.3744(4) Å, while the Ru–N distances are 2.0765(13) Å and 2.0526(13) Å. The average Ru–C bond lengths range from 2.1970(15) Å to 2.2387(16) Å, indicating slight variations in *p*-cymene ligand interactions, but in agreement with similar compounds described in the literature.^39^ The bond distance from the Ru center to the centroid (C*_t_*) of the η^6^-*p*-cymene ring was 1.463, 1.455, and 1.459 Å for **2**, **5**, and **6.CH**_**2**_ **Cl**_**2**_ respectively. These results are similar to those observed by Singh and co-workers^36^ for a similar complex bearing a bis-imidazole methane-based, as an ancillary ligand, labeled as [**C-4**], where the Ru center to the C*_t_* was 1.445 Å. The N–Ru–Cl angle is 84.73(4)° for **2**, contributing to the overall geometry of the coordination sphere in a typical piano-stool geometry, which was observed in all three structures.

Similarly, compound **5** also crystallizes in the *P*2_1/_ *c* space group with a monoclinic unit cell. The structural parameters are comparable to those of compound **2**, with minor deviations in bond lengths and angles due to different ligand substitutions, which compose the **N–N** ^**2**^ and **N–N** ^**5**^ α-diimine ligands. In contrast, compound **6.CH**_**2**_ **Cl**_**2**_ belongs to the *I*2/*a* space group and displays larger unit cell dimensions with *a* = 16.6760(2) Å, *b* = 15.4841(2) Å, *c* = 22.2792(3) Å, and β = 99.9480(10)°. The Ru–Cl bond length of 2.3781(7) Å is slightly longer than in compounds **2** and **5**, whereas the Ru–N distances (2.079(2) Å and 2.077(2) Å) remain comparable, and in agreement with the literature.^36–39^ The Ru–C bond lengths range from 2.192(3) to 2.230(3) Å, suggesting a similar coordination environment to the *P*2_1_/*c* counterparts. The N–Ru–Cl bond angles are 83.33(7)° and 85.97(6)°, slightly deviating from the values observed in compounds **2** and **5,** but like related complexes described in the literature.^36–39^

These variations in bond lengths and angles reflect the influence of space group symmetry on the structural organization of the complexes. The *I*2/*a* structure of **6.CH**_**2**_ **Cl**_**2**_ exhibits a more expanded lattice, which may contribute to slightly elongated metal–ligand bonds. The geometric parameters, particularly the Ru–N and Ru–Cl distances, suggest a conserved coordination environment, while minor angular deviations indicate subtle effects from crystal packing forces. These observations highlight the impact of crystallographic symmetry on the coordination sphere of Ru­(II) complexes, which may influence their electronic and catalytic properties.

### 3.2 DFT

To investigate the structures of complexes **1–** **4**, DFT calculations were performed to determine the optimized structural parameters and electronic properties. These four complexes were chosen for DFT calculation because they exhibited the best catalytic performance in the dehydrogenation of FA (see next section).

For complexes **1**, **3**, and **4**, the calculations revealed two different optimized geometries: the first with the methyl group of *p*-cymene positioned over the chlorine (designated as **a**) and the second with the isopropyl group of *p*-cymene over the chlorine (designated as **b**). In contrast, for complex **2**, eight distinct geometries were identified, always featuring the **a** and **b** orientations, along with additional variations due to the positions of methyl groups in the ortho position of the α-diimine rings (see Figure 5). These possible variations of the *p*-cymene ring over the α-diimine ligands and chlorine are consistent with the rotamers observed in solution by ^1^H NMR and NOE analyses.

**Figure 5:**
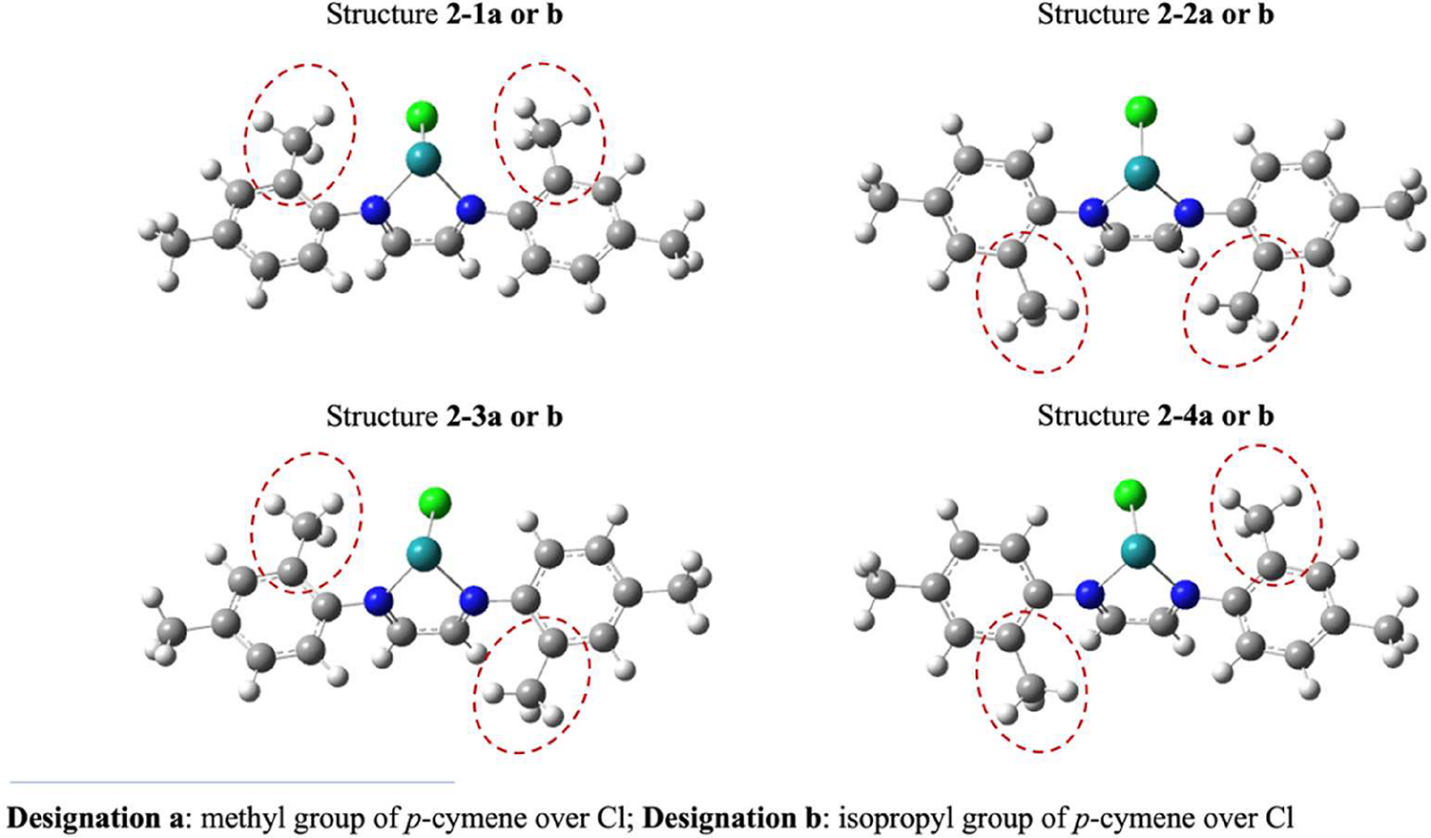
Description of the optimized structures for complex **2**. The *p*-cymene ligand was omitted for clarity and better visualization.

In structures **2–1a** and **b**, the ortho-positioned methyl groups are oriented in the upper plane of the diimine molecule. Conversely, in structures **2–2a** and **b**, the ortho methyl groups are positioned in the lower plane of the diimine ligand. The structures **2–3** and **2–4** (**a** and **b)** have a mixture of both cases.

The structure **2–2b**, described in Figure 5, exhibited the smallest HOMO–LUMO gap (2.924 eV), which led to the assignment of a stabilization energy of 0.00 kcal mol^–1^ for this structure. The relative values for the other structures are presented in Table 4, and the representation of HOMO and LUMO orbitals for each optimized structure **1**–**4** is available in the DFT section of the Supporting Information.

**Table 4:** Calculated HOMO-LUMO Gap and Stabilization Energy for Each Optimized Structure of Complex **2**

structure	GAP HOMO–LUMO (eV)	relative stabilization energy (kcal mol^–1^)
2–1a	2.952	+4.85
2–1b	3.055	+4.18
2–2a	2.927	+1.05
2–2b	2.924	0.00
2–3a	2.935	+1.69
2–3b	2.949	+2.41
2–4a	2.949	+3.27
2–4b	3.026	+1.63

The relative stabilization energy of structure **2–2a** is only 1.05 kcal mol^–1^ higher than that of structure **2–2b** and exhibits a HOMO–LUMO gap that is 0.003 eV larger than that of the most stable structure. In other words, structures **2–2a** and **2–2b** exhibit very similar stabilization energies and coexist in solution, as observed in the ^1^H NMR spectrum of complex **2** (Figure S.47). This complex displayed duplicated signals, both for the septet of the isopropyl hydrogen of the *p*-cymene ligand and for the methyl groups of the same moiety.

The composition of the HOMO orbital in structure **2–2a** is 29.52% (Ru), 12.25% (Cl), 12.10% (*p*-cymene), and 46.13% (N–N^2^), while the composition of the LUMO orbital is 7.71% (Ru), 2.51% (Cl), 3.64% (*p*-cymene), and 86.14% (N–N^2^). This composition is very similar for all complexes, with a slight difference among them. Therefore, these results endorse the MLCT assignment in the UV/vis data section, as reported in section 2 and Table S.6. Natural transition orbitals (NTO) were used to describe the excited states for structures **1**–**4**, with a good fit to the experimental UV/vis data, which are described in detail in the DFT section of Supporting Information.

Related to complex **2**, UV/vis spectra determined by TD-DFT revealed that structures **2–2a** and **2–2b** (Figures S.73 and S.74 respectively) most closely resembles the experimentally obtained spectrum (Figure S.32). The transitions at shorter wavelengths in **2–2a** (Figure S.73) refer to predominantly intraligand interactions, with a small participation of the metal center, excited states 1–5 (Schemes S.21–S.25). While the excited states 6 and 7, centered at 443 and 454 nm, respectively, revealed electronic transitions centered in the orbitals of the Ru and Cl atoms to regions of the azomethine group of the α-diimine ligand (Schemes S.26 and S.27).

The accuracy of the DFT results aligns well with the data obtained from X-ray diffraction, as the crystalline structure of compound **2** (Figure 4) corresponds to the structure **2–2a** described in Figure 6, where the methyl group of the *p*-cymene ligand is oriented over the chlorine atom coordinated to ruthenium.

**Figure 6:**
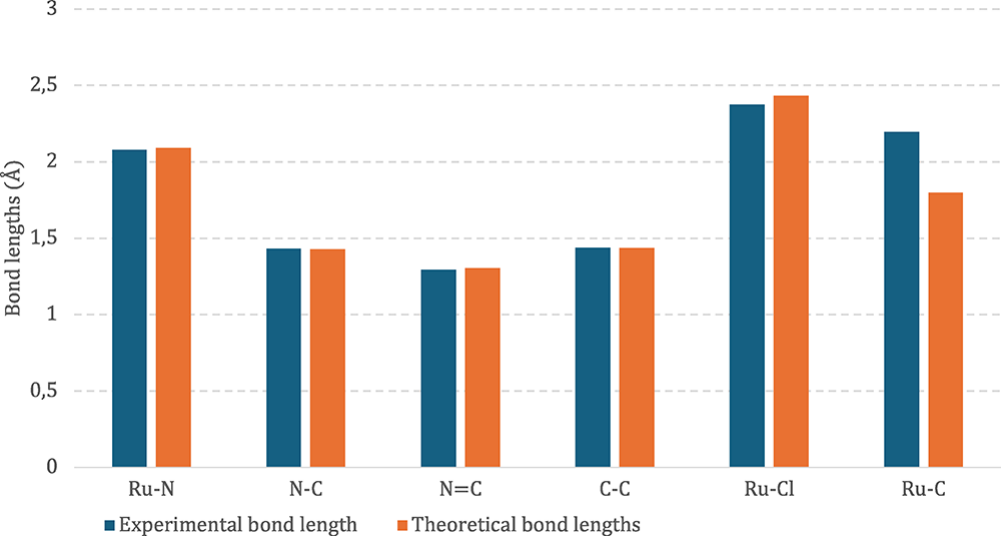
Correlation of experimental and theoretical bond lengths of **2** (blue) and **2–2a** (orange).

Figure 6 exhibits a good correlation between theoretical and experimental bond lengths, except for Ru-η^6^-*p*-cymene bonds, which are shorter than expected for the Ru–C average distance. However, this theoretical value is close to the Ru center relative to the centroid (C*_t_*) of the η^6^-*p*-cymene ring, which was found at 1.463 Å and presented in section 3.1.

Additionally, the values of *f*+ and *f*– were calculated by the Hirshfeld charge method^60^ for each optimized structure for complexes **2–2 (a** and **b)** (Tables S.35 and S.36) and the results applied to the Fukui function. Therefore, Figure 7 represents the removal of an electron from the molecule, indicating the initial stage of an electrophilic attack, while the Fukui function *f*+ represents the addition of an electron to the molecule, indicating the initial stage of a nucleophilic attack. The most electrophile-prone regions of the molecules are located on the ruthenium center and the chlorine atom, whereas the regions over the nitrogen atoms and the bridge of the iminic carbons are the most susceptible to nucleophilic attack.

**Figure 7:**
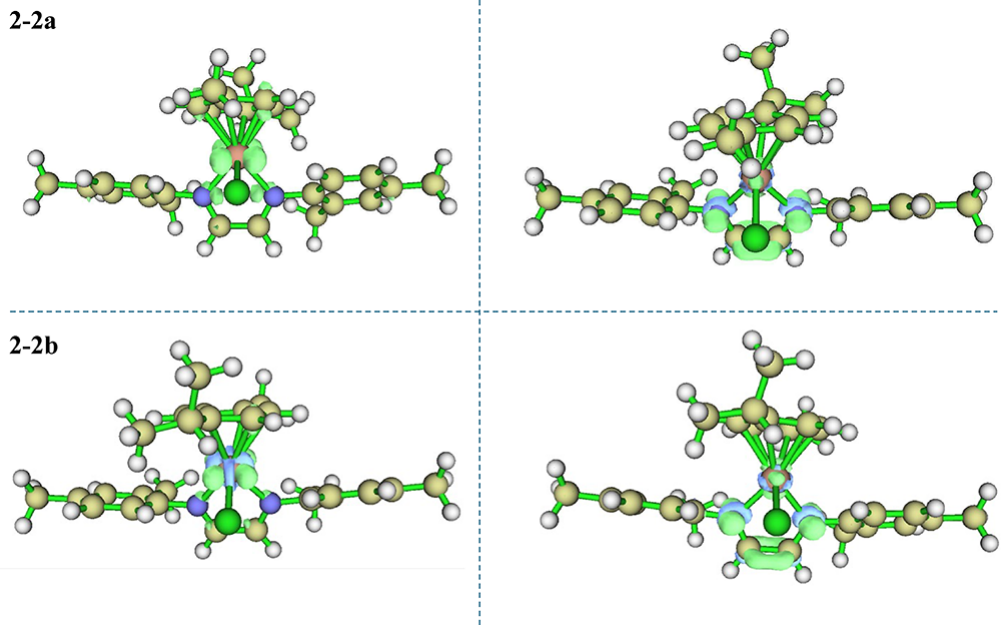
Fukui function for the optimized structures **2–2a** and **2–2b**, *f*– (left) represents an electrophilic attack region and *f*+ (right) represents a nucleophilic attack region.

Representative molecular structures for optimized structures **2–2 (a** and **b**) are shown in Figure 7. A similar approach was carried out for **1**, **2**, and **3** complexes, and the results are summarized in the DFT section in the Supporting Information.

### 3.3 Catalysis

The complexes from **1** to **7** were applied as precatalysts in the dehydrogenation of FA. After some screenings, the best molar ratio was established as 1/1200/843 Ru/FA/Base. All catalytic runs were replicated 3-fold at 60 °C, free of solvents or further additives. The results are summarized in Table 5.

**Table 5:** Conversion and Turnover Frequency for Dehydrogenation of Neat Formic Acid Using the Complexes from **1** to **7** as Pre-Catalysts at 60 °C

complex	conversion^a^ ± SD %	TOF^b^ ± SD h^–1^	time (min.) 50% conversion	TOF 50%^b^ ± SD h^–1^
**1** ^c^	95 ± 1	627 ± 22	82	437 ± 11
**1** ^d^	100	956	40	898 ± 9
**2**	90 ± 1	335 ± 6	142	253 ± 4
**3**	91 ± 1	361 ± 4	130	276 ± 3
**4**	27 ± 2	92 ± 3		
**5**	9 ± 5	33 ± 5		
**6**	6 ± 4	24 ± 8		
**7**	3 ± 5	15 ± 3		

a Average of 3-fold reactions.

b Turnover frequency due to H_2_ production.

c First run.

d Second and third run, catalyst recycling.

Complexes bearing electron-donating methyl groups at the *ortho* or *para* positions of the imine aromatic ring, complexes **1**, **2**, and **3**, exhibited the highest conversion values, with complex **1** standing out due to the presence of a methyl group at each *ortho* position. At 50% conversion, the fastest catalyst is clearly the complex bearing the **N–N** ^1^ ligand substituted with methyl groups at the ortho positions. Complete conversion and an enhanced TOF value (956 h^–1^) were observed in the second run, suggesting that the active catalytic species were generated in situ during the first run, which agrees with the induction period observed in the first run (Figures 8 and S.83). Similar behavior was also observed by Treigerman and Sasson in a related system using the [RuCl­(μ-Cl)­(*p*-cym)]_2_ as catalytic precursor.^43^

**Figure 8:**
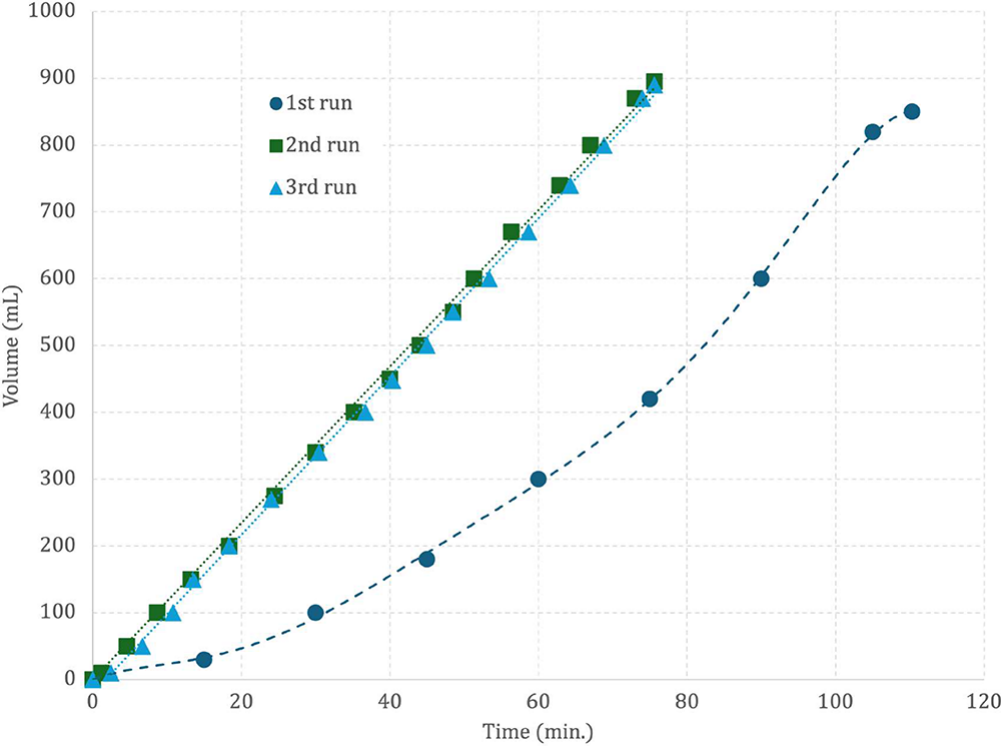
Dehydrogenation reaction of formic acid using **1** as pre-catalyst at 60 °C, with molar ratio Ru/FA/NEt_3_ = 1/1204/843.

Interestingly, increasing the steric hindrance at the *ortho* positions by introducing two isopropyl groups (complex **4**) led to a significant drop in catalytic conversion by 68%, from 94.8% to 27.2%, for complexes **1** and **4**, respectively. This result clearly indicates that enhanced steric hindrance at the *ortho* positions reduces the catalytic activity for FA dehydrogenation within the studied complex framework.

When complexes **5** and **6** were employed as precatalysts, the catalytic activity drastically decreased. The presence of electron-withdrawing substituents, especially F and Cl at the *para* position of the imine ring, resulted in low, but comparable, conversion rates of 8.5 and 6.4%, respectively. These results demonstrate that electron-withdrawing substituents on the imine ring diminish the catalytic activity toward FA dehydrogenation.

The substitution of the aromatic ring within the diazomethine moiety with a cyclohexyl group (complex **7**) resulted in the lowest conversion observed among all of the studied complexes. Under the tested conditions, only 3.2% of FA was converted into H_2_ and CO_2_ using complex **7**. This finding suggests that strong and bulky electron-donating groups, such as cyclohexyl, significantly alter the electronic structure of the complexes, thereby reducing their catalytic activity as precatalysts in the FA dehydrogenation reaction.

The influence of different bases was tested using complex **1** on the dehydrogenation of neat FA, maintaining a constant molar ratio of Ru/FA/Base = 1/1200/843 at 60 °C. In addition to triethylamine, tripropylamine, triethanolamine, *tert*-BuOK, HCOOK, HCOONa, ethanol, water, NaBH_4_, and pyridine were evaluated (Figure 9).

**Figure 9:**
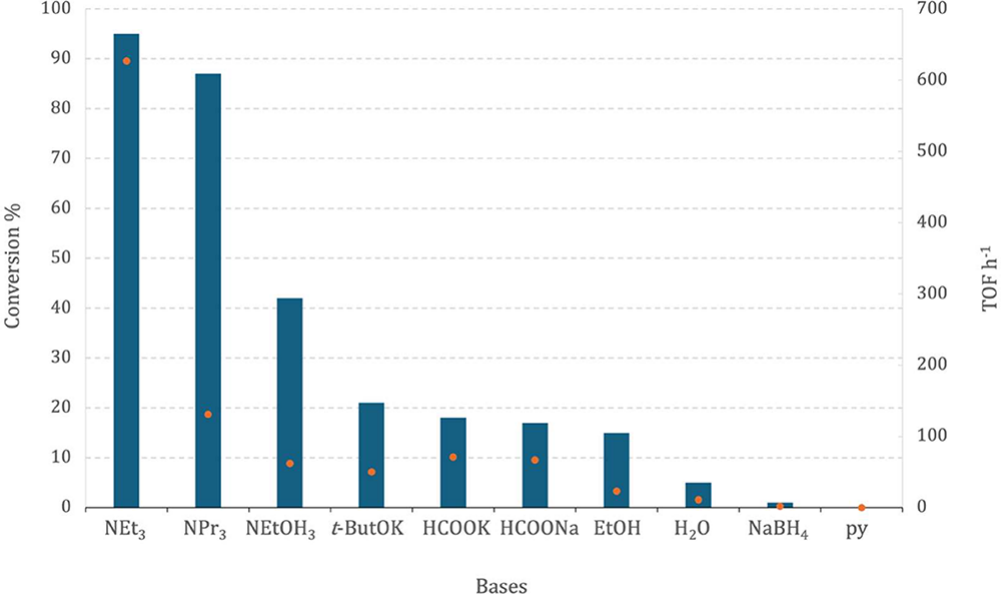
Comparison of different bases on the dehydrogenation of formic acid using complex **1** as a precatalyst (blue bars = conversion, orange dot = TOF).

The tertiary amines triethylamine (NEt_3_) and tripropylamine (NPr_3_), with p*K*_a_ values of 10.75 and 10.65, respectively, exhibited the highest catalytic activity, achieving conversions above 85% and a TOF value of around 627 h^–1^ in the case of NEt_3_. These results suggest that bases with moderate strength are optimal for promoting the reaction, possibly due to a favorable balance between basicity and nucleophilicity that facilitates key steps in the catalytic cycle, such as proton abstraction and stabilization of reactive intermediates.

When a suitable Bro̷nsted–Lowry base such as NEt_3_ or NPr_3_ is employed, FA dehydrogenation is observed, even though steric hindrance may influence the reaction rate. The corresponding protonated amines (NEt_3_H^+^ and NPr_3_H^+^) may play a role as a chloride scavenger, promoting the dissociation of chloride ligands from the ruthenium precatalyst, exchanging Cl^–^ for HCOO^–^.

In contrast, the stronger base potassium *tert*-butoxide (*t*-ButOK, p*K*_a_ = 17) resulted in significantly lower activity, with conversion values below 25% and TOF values under 100 h^–1^. This behavior may be attributed to possible catalyst deactivation, unfavorable coordination equilibria, or steric hindrance associated with bulky alkoxide bases. Similarly, ethanol and water led to only modest conversions. Considering the p*K*_a_ values of their conjugate acids (EtOH_2_^+^, p*K*_a_ ≈ −2; H_3_O^+^, p*K*_a_ ≈ 1.7), both solvents behave as very weak bases compared to FA (p*K*_a_ 3.75). This observation reinforces the idea that their limited basicity is insufficient to enhance the catalytic process.

Weaker bases such as triethanolamine (NEtOH_3_, p*K*_a_ = 7.74) and pyridine (p*K*_a_ = 5.2) were clearly less effective, with the latter showing negligible catalytic activity. This lack of reactivity is consistent with insufficient basicity to promote the proton transfer steps necessary for efficient hydrogen evolution.

Formate salts (HCOONa and HCOOK) and sodium borohydride (NaBH_4_), although not true Bro̷nsted–Lowry bases under the tested conditions, displayed low catalytic activity. Their roles may be limited due to their ionic nature, low solubility, or, in the case of NaBH_4_, potential side reactions such as hydride transfer or catalyst degradation. While the dissolution of formate salts in FA leads to an acid–base equilibrium, no net reaction occurs, as FA and the formate ion represent a conjugate acid–base pair. Consequently, no additional driving force is introduced into the system. In contrast, the presence of a Bro̷nsted–Lowry base such as NEt_3_ not only facilitates formate generation but also produces the triethylammonium species, potentially acting as a more efficient chloride scavenger under these conditions.

However, in the presence of a good nucleophile instead of a base, computational data suggest that a nucleophilic attack is likely to occur directly on the diazomethine moiety, triggering the decomposition of the synthesized complex and, consequently, inhibiting its catalytic activity (Figures 7, S.69, S.79, and S.82).

The observed rate constants (*k*_obs_) for the FA dehydrogenation catalyzed by complex **1** follow both Arrhenius and Eyring models (Figures S.84 and S.85) within the range of 40–60 °C (Tables S.47 and S.48).

As described by Mayer and co-workers,^61^ who emphasized the importance of considering the induction period in kinetic models, an analysis based on the final-time conversion was adopted. This approach captures the overall kinetic behavior, including the induction period, thereby avoiding underestimation or overestimation of apparent rate constants. Similar observations were reported by Treigerman and Sasson,^43^ who noted that induction periods are a recurrent feature in homogeneous FA decomposition and are related to the activation of the precatalyst. According to their study, this behavior can be altered in the first run, without an induction period, when the tertiary amine, the solvent (DMF), the ruthenium precursor, and the ligand are premixed at 60 °C for a period of 1 h prior to the introduction of the FA.

In the present work, such pretreatment was not applied since the reactions were carried out in neat FA. Therefore, the conversion rate at the end-time was used as the basis for determining *k*_obs_. To ensure the robustness of this method, its results were validated by comparison with the induction–growth kinetic model, labeled here as *X*_model_. While the end-point method provides a straightforward treatment, the *X*_model_ offers additional mechanistic insight by explicitly separating the induction time (τ) from the pseudo–first-order growth constant (*k*). Both approaches yielded consistent activation parameters, thereby confirming the reliability of the end-point treatment to represent the catalytic kinetics across the 40–60 °C range. Further details are provided in the kinetic section of the Supporting Information.

The activation entropy (Δ*S* ^‡^) was determined to be +137 cal mol^–1^ K^–1^, indicating a transition state characterized by significantly greater disorder compared with the reactant state. This positive entropy change is consistent with a dissociative mechanism and supports the hypothesis of Cl^–^ dissociation as a key step in the catalytic cycle. Furthermore, the enthalpy of activation (Δ*H* ^‡^) was determined to be 70.3 kcal mol^–1^, which is in excellent agreement with the activation energy (*E*_a_) obtained from the Arrhenius plot (*E*_a_ = 70.9 kcal mol^–1^), reconfirming the interpretation of a substantial structural reorganization occurring during the transition state.

The Gibbs free energy of activation (Δ*G* ^‡^) was calculated as 24.5 kcal mol^–1^, indicating that the reaction is thermodynamically accessible under the experimental conditions. Despite the high activation enthalpy, the significant entropic contribution favors transition state formation, facilitating the catalytic process at moderate temperatures.

Based on kinetic experiments and DFT calculations, a plausible mechanism has been proposed for FA dehydrogenation, Scheme 2. The process begins with an acid–base equilibrium between FA and triethylamine, generating a triethylammonium cation capable of displacing the chloride ligand from the ruthenium center. This substitution creates a vacant coordination site, which is subsequently occupied by a formate anion to yield intermediate species III, identified as the catalytically active complex. This intermediate then undergoes β-hydride elimination, releasing carbon dioxide and forming the hydride complex IV. In the presence of an additional FA molecule, a hydrogen-bonding interaction facilitates the regeneration of intermediate III, accompanied by the release of molecular hydrogen, thereby completing the catalytic cycle.

**Scheme 2:**
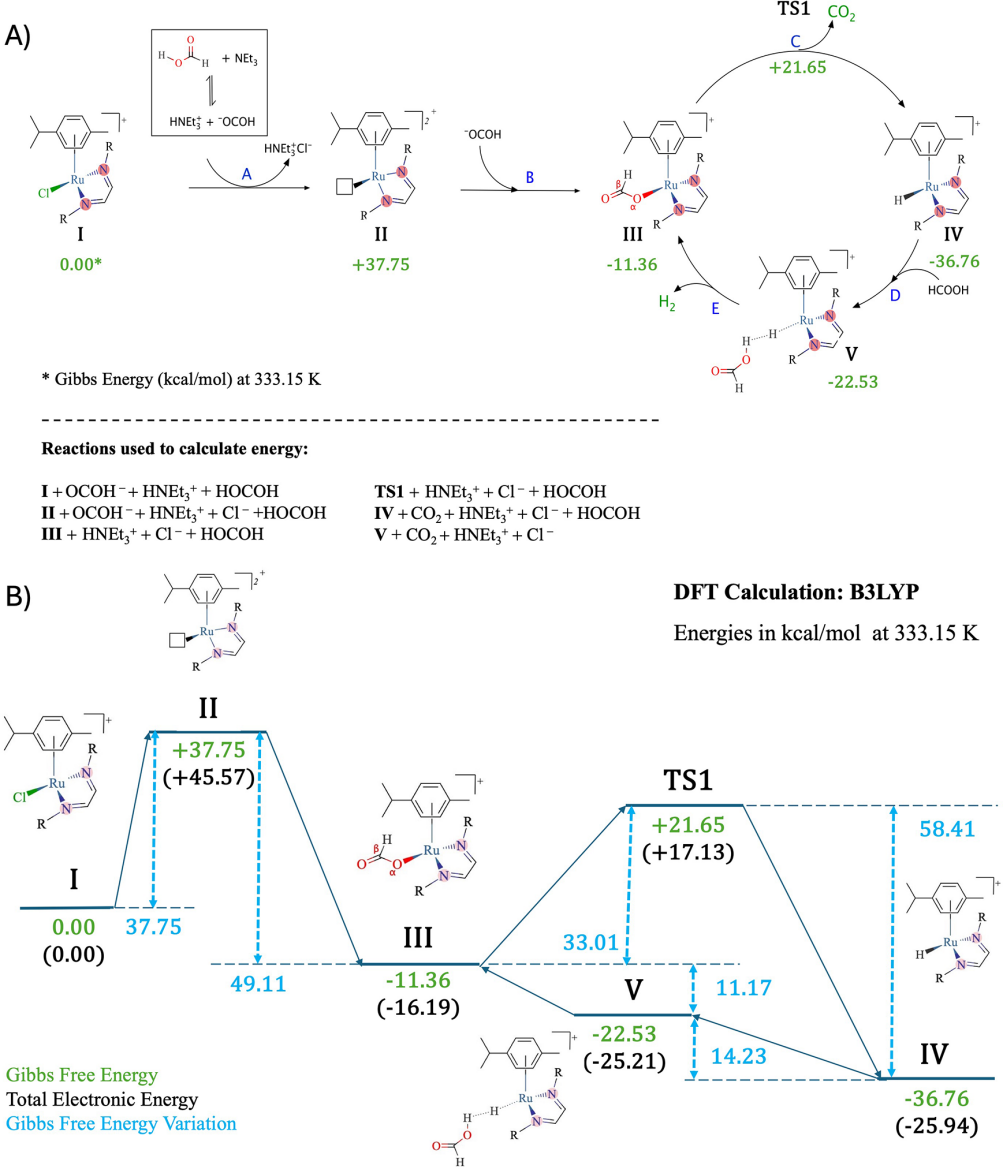
(A) Mechanism Proposed for the Neat Formic Acid Dehydrogenation by Related Complexes. (B) DFT Energy Profile

Thermodynamic data derived from DFT calculations indicate that the initial steps are endergonic, with Gibbs free energy changes (Δ*G*) of +37.75, + 26.39, and +33.01 kcal mol^–1^ for the transitions from I to II, II to III, and III to the corresponding transition state TS1, respectively. Although DFT calculations indicate a high energy barrier for these steps, it is important to consider that solvent effects, hydrogen bonding with FA, and potential assistance from the base may significantly reduce this barrier in solution. Osipova et al.,^62^ demonstrated that hydrogen bond donation from Et_3_NH^+^ or excess HCOOH can substantially lower the decarboxylation barrier in related systems. These noncovalent interactions may also contribute to the activation of metal–ligand bonds, such as Ru–Cl, by stabilizing transition states or facilitating ligand exchange.

Therefore, it is likely that the actual energy barriers under the experimental conditions are significantly lower than those calculated in the absence of such interactions. The observed catalytic activity at mild temperatures supports this interpretation, where the Δ*G* ^‡^ = 24.5 kcal mol^–1^ confirms that the overall process remains thermodynamically accessible under the applied conditions.

The transformation from TS1 to intermediate IV is highly exergonic, with Δ*G* = −58.41 kcal mol^–1^, indicating a strongly favorable hydride formation step. Subsequent steps involving the formation of hydrogen-bonded adduct V (IV···HCOOH) and the regeneration of species III are endergonic, with Δ*G* values of +14.23 and +11.17 kcal mol^–1^, respectively. These thermodynamic features suggest that the hydride intermediate IV may establish a dihydrogen bond (M–H···H–X) with FA. As discussed by Belkova et al.,^63^ dihydrogen bonding is characterized by weak to moderate enthalpic stabilization and can significantly modulate the electronic structure of both the hydride and the protic species involved.

In the current work, the formation of complex V may act as a thermodynamically stable but kinetically inert resting state, consistent with the observed Gibbs free energy of +14.2 kcal mol^–1^. Moreover, the weak formation constant associated with V suggests that this equilibrium lies toward dissociation, further supporting a scenario in which V serves as a kinetic bottleneck, slowing the turnover of hydrogen evolution.^64^

Overall, the proposed mechanism is consistent with the catalytic dehydrogenation of FA, releasing molecular hydrogen and carbon dioxide in a cycle in which intermediate III acts as the true catalyst. The experimentally observed induction period agrees with the proposed pathway, particularly the energetically demanding initial steps, in which chloride dissociation appears to be the rate-limiting step.

## 4 Conclusions

In this study, a series of seven ruthenium­(II) half-sandwich complexes bearing structurally diverse α-diimine ligands was successfully synthesized and characterized. The structural features of the complexes, including their solution behavior and solid-state configurations, were elucidated by using a combination of spectroscopic techniques, elemental analysis, and single-crystal X-ray diffraction. DFT calculations provided further insights into their electronic structures, highlighting the subtle balance between steric and electronic effects imparted by the substituents on the α-diimine ligands. All complexes were evaluated as precatalysts for the solvent-free dehydrogenation of FA under mild conditions. Complex **1**, featuring methyl groups at both ortho positions in the iminic ring, displayed superior catalytic activity, reaching nearly complete conversion and the highest TOF value among the series for the first cycle. Complete conversion and improvement in TOF value were observed in the second and subsequent cycles. The introduction of bulky or electron-withdrawing substituents led to a significant decrease in catalytic efficiency, emphasizing the sensitivity of the catalytic cycle to both steric and electronic modulations at the ligand framework. The experimental activation parameters obtained via Eyring and Arrhenius analyses indicate an entropically driven process, consistent with a transition state involving CO_2_ release and ligand dissociation. These values are consistent with the DFT-calculated mechanism, which identifies the initial induction steps as endergonic as well as the last two steps to close the catalytic cycle. Furthermore, the nature of the base employed was shown to play a critical role in the catalytic performance, proving essential for efficient dehydrogenation.

## Supplementary Material



## Notes

The Supporting Information provides detailed experimental and analytical data for all ligands and complexes described in the main text. It includes elemental analysis, UV/vis, FTIR, and NMR (^1^H, ^13^C, HSQC, and HMBC) spectra of the α-diimine ligands, as well as comprehensive characterization of the ruthenium­(II) complexes, including conductivity measurements and spectroscopic profiles. Additionally, single-crystal X-ray diffraction data and crystallographic parameters for **2**, **5,** and **6.CH**_**2**_ **Cl**_**2**_ complexes are presented. DFT computational details, optimized structures, frontier molecular orbital data, Natural Transition Orbitals (NTO), Hirshfeld charge tables, and the optimized structures using Fukui function are also included, along with kinetic data and calculated energy profiles supporting the proposed catalytic mechanism (PDF)
