# Supplementary material for: Molecular Hydrogen Generation from Neat Formic Acid Catalyzed by Ruthenium–Cymene α‑Diimine Complexes
Source: ACS Omega. 2025 Nov 13;10(46):55404–19. doi: 10.1021/acsomega.5c05610 (PMC12658794; doi:10.1021/acsomega.5c05610)
Supplement: Supplementary file 1 [file ao5c05610_si_001.pdf]

Supplementary Material

Molecular Hydrogen Generation from Neat Formic Acid Catalyzed by Ruthenium–Cymene α-Diimine Complexes

Cássio R. A. do Prado [a,b], Lucas da S. dos Santos [a,b], Ellen C. Guimarães [a], Laís A. Tomaz [a], Lucas F. Martins [c], Luciano M. Lião [c], Leonardo T. Ueno [d], Valdemiro P. Carvalho-Jr [e], Alexandre B. de Carvalho [f], Javier Ellena [f], Luís R. Dinelli [a], André L. Bogado [a,]*

[a] *Institute of Exact and Natural Sciences of Pontal, Federal University of Uberlândia, ICENP – UFU, 38304-402, Ituiutaba, MG, Brazil*
[b] *Institute of Chemistry, Federal University of Uberlândia, IQ - UFU, 38400-902,Uberlândia, MG, Brasil*
[c] *Institute of Chemistry, Federal University of Goiás, IQ - UFG, 74690-900, Goiânia, GO, Brazil*
[d] *Department of Chemistry, Aeronautics Institute of Technology, General Command for Aerospace Technology, ITA, 12228-900, São José dos Campos, SP, Brazil*
[e] *São Paulo State University, Unesp, School of Technology and Sciences, Presidente Prudente, SP, 19060-900, Brazil*
[f] *Sao Carlos Institute of Physics, University of Sao Paulo, IFSC – USP, 13566-950, Sao Carlos, SP, Brazil*

Table of Contents

Elemental analysis of the ligands 3
UV/vis of ligands 3
FTIR of ligands 7
^{1}H and ^{13}C NMR data of ligands 12
Elemental Analysis of Complexes. 20
Conductivity 20
UV/vis data of complexes 21
FTIR data of the complexes. 25
NMR spectroscopy of complexes. 29
X-ray data 39
Crystal Data and Experimental for **2** 39
Crystal Data and Experimental for **5** 49
Crystal Data and Experimental for **6.CH_2Cl_2.** 58
DFT calculation 68
Complex **1** 68
Complex **2** 84
Complex **3** 96
Complex **4** 106
Kinetic 119
Definitions and symbols 120
Equations 120
Worked example (50°C) 121
Arrhenius and Eyring plots. 122
Arrhenius (using ln k_{obs} *vs* 1/T) 122
Eyring (using ln (k_{obs}/T) *vs* 1/T). 123
Step-by-step: how to obtain each parameter 124
(A) Activation energy E_a (Arrhenius) 124
(B) Enthalpy of activation $\Delta H^{\ddagger}$ (Eyring) 124
(C) Entropy of activation $\Delta S^{\ddagger}$ (Eyring) 124
(D) Gibbs free energy of activation $\Delta G^{\ddagger}$ (from Gibbs). 124
Induction–growth kinetic model (X_{model}) 125
Data at 60°C (values from 1st run in the Fig. 8 in the main text) 126
Step-by-step how to use the Induction–growth model (X_{model}) in the Excel. 127
Induction – growth kinetic model applied in all temperature curves of Fig. S.83. 129
Comparative analysis of end-point and X_{model} approaches. 134
Comparison of the activation parameters using rate constants from both methods 135

Elemental analysis of the ligands

Table S.1: Elemental analysis of α-diimines.

Ligand	% C found (theoretical)	% H found (theoretical)	% N found (theoretical)
**N-N¹**	81.67 (81.78)	7.82 (7.63)	10.31 (10.60)
**N-N²**	81.88 (81.78)	7.86 (7.63)	10.39 (10.60)
**N-N³**	82.27 (82.15)	8.10 (8.27)	9.29 (9.58)
**N-N⁴**	82.51 (82.93)	9.21 (9.64)	7.33 (7.44)
**N-N⁵**	69.13 (68.85)	4.14 (4.13)	11.28 (11.48)
**N-N⁶**	60.44 (60.67)	3.63 (3.64)	9.84 (10.11)
**N-N⁷**	76.48 (76.31)	10.77 (10.98)	12.56 (12.71)

N-N^{1} = N1,N2-bis(2,6-dimethylphenyl)ethane-1,2-diimine; **N-N^{2}** = N1,N2-bis(2,4-dimethylphenyl)ethane-1,2-diimine; **N-N^{3}** = N1,N2-bis(2,4,6-trimethylphenyl)ethane-1,2-diimine ; **N-N^{4}** = N1,N2-bis[2,6-bis(propan-2-yl)phenyl]ethane-1,2-diimine; **N-N^{5}** = N1,N2-bis(4-fluorophenyl)ethane-1,2-diimine; **N-N^{6}** = N1,N2-bis(4-chlorophenyl)ethane-1,2-diimine and N-N^{7} = N1,N2-dicyclohexylethane-1,2-diimine.

UV/vis of ligands

Table S.2: UV/vis data of α-diimines.

Ligand	λ (nm)	log ε (L cm^{-1} mol^{-1})
**N-N^{1}**	230, 253, 356	7.87, 7.55, 7.07
**N-N^{2}**	230, 249, 280, 361	7.28, 7.26, 7.23, 7.31
**N-N^{3}**	230, 259, 364	7.58, 7.28, 6.89
**N-N^{4}**	230, 257, 361	7.94, 7.51, 6.92
**N-N^{5}**	235, 282, 340	7.44, 7.22, 6.99
**N-N^{6}**	254, 289	7.24, 7.00
**N-N^{7}**	230, 262	8.02, 7.77

The ligand solutions were prepared in CH_2Cl_2 (10^{-8} mol L^{-1}), scanned in the range of 200 to 500 nm and 25°C. **N-N^{1}** = N1,N2-bis(2,6-dimethylphenyl)ethane-1,2-diimine; **N-N^{2}** = N1,N2-bis(2,4-dimethylphenyl)ethane-1,2-diimine; **N-N^{3}** = N1,N2-bis(2,4,6-trimethylphenyl)ethane-1,2-diimine ; **N-N^{4}** = N1,N2-bis[2,6-bis(propan-2-yl)phenyl]ethane-1,2-diimine; **N-N^{5}** = N1,N2-bis(4-fluorophenyl)ethane-1,2-diimine; **N-N^{6}** = N1,N2-bis(4-chlorophenyl)ethane-1,2-diimine and **N-N^{7}** = N1,N2-dicyclohexylethane-1,2-diimine.

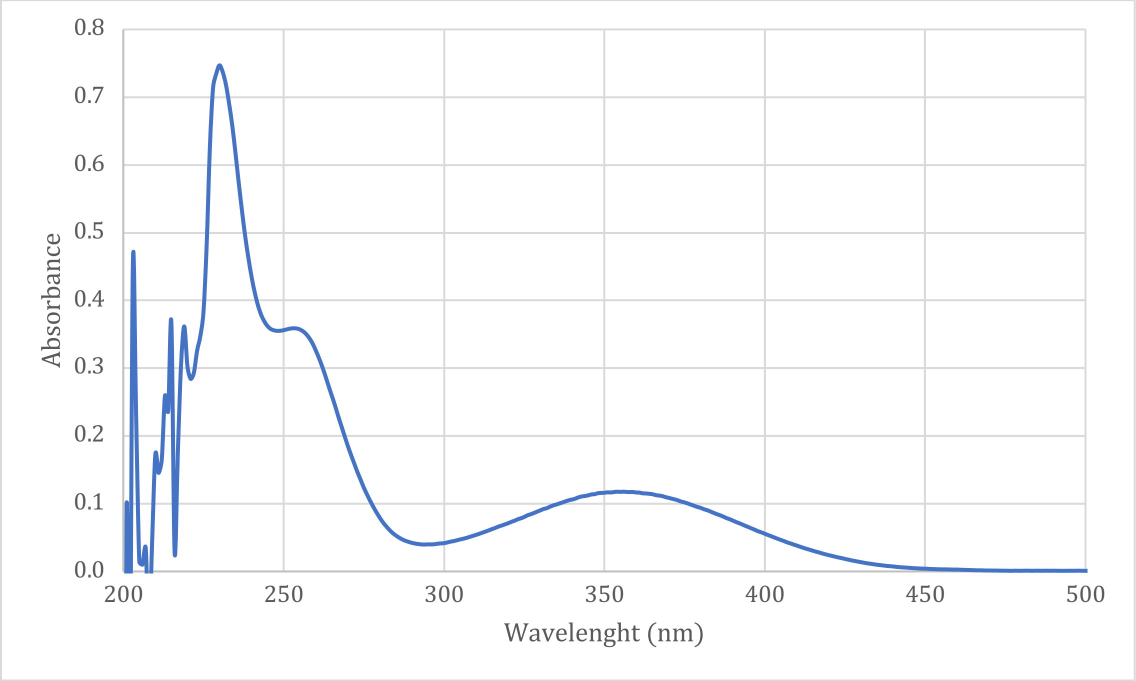

Fig.S.1: UV/vis spectrum of N1,N2-bis(2,6-dimethylphenyl)ethane-1,2-diimine (**N-N**[1]).

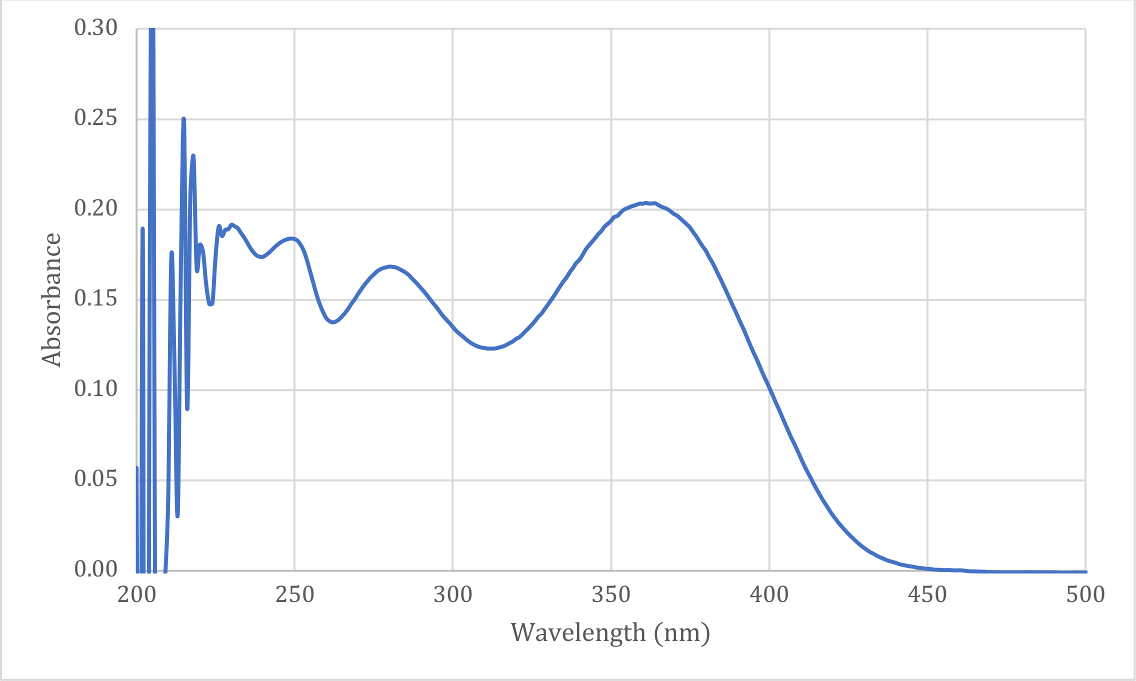

Fig.S.2: UV/vis spectrum of N1,N2-bis(2,4-dimethylphenyl)ethane-1,2-diimine ligand (**N-N**[2]).

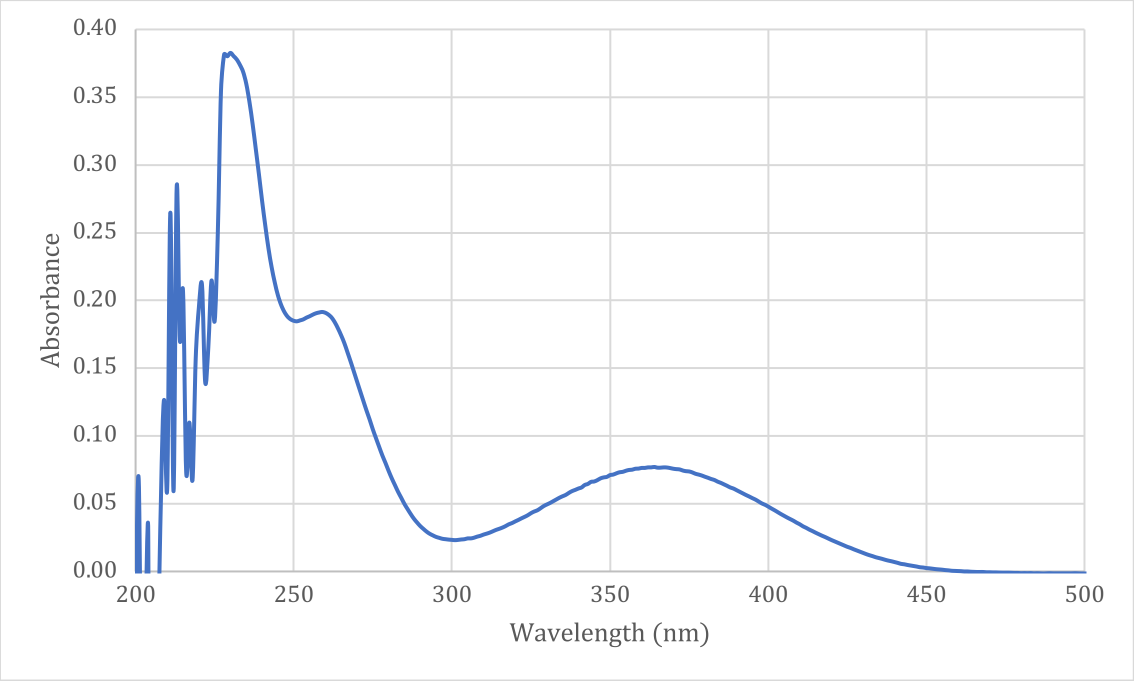

Fig.S.3: UV/vis spectrum of N1,N2-bis(2,4,6-trimethylphenyl)ethane-1,2-diimine (**N-N**[3])

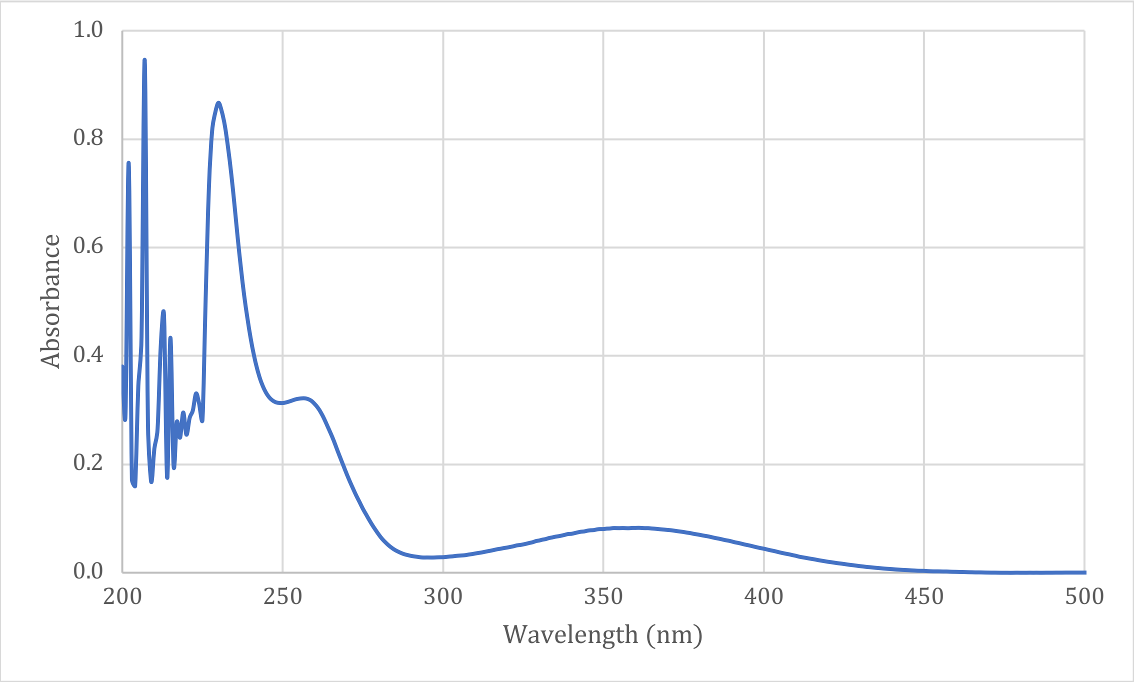

Fig.S.4: UV/vis spectrum of N1,N2-bis[2,6-bis(propan-2-yl)phenyl]ethane-1,2-diimine (**N-N**[4]**).**

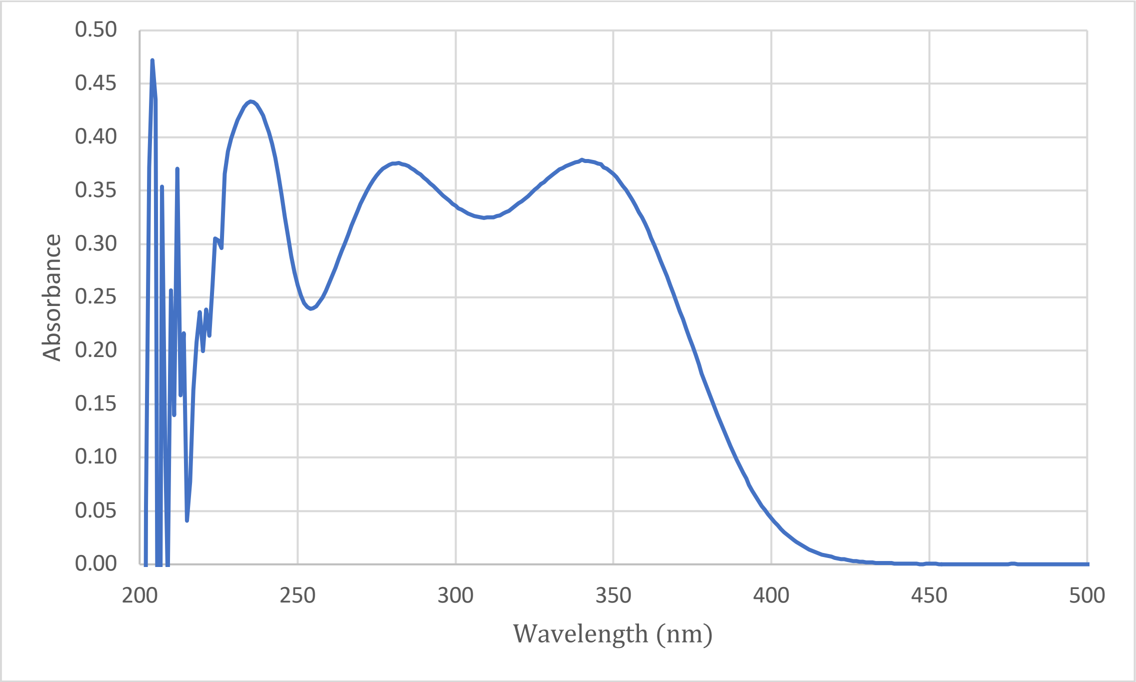

Fig.S.5: UV/vis spectrum of N1,N2-bis(4-fluorophenyl)ethane-1,2-diimine (**N-N[5]**).

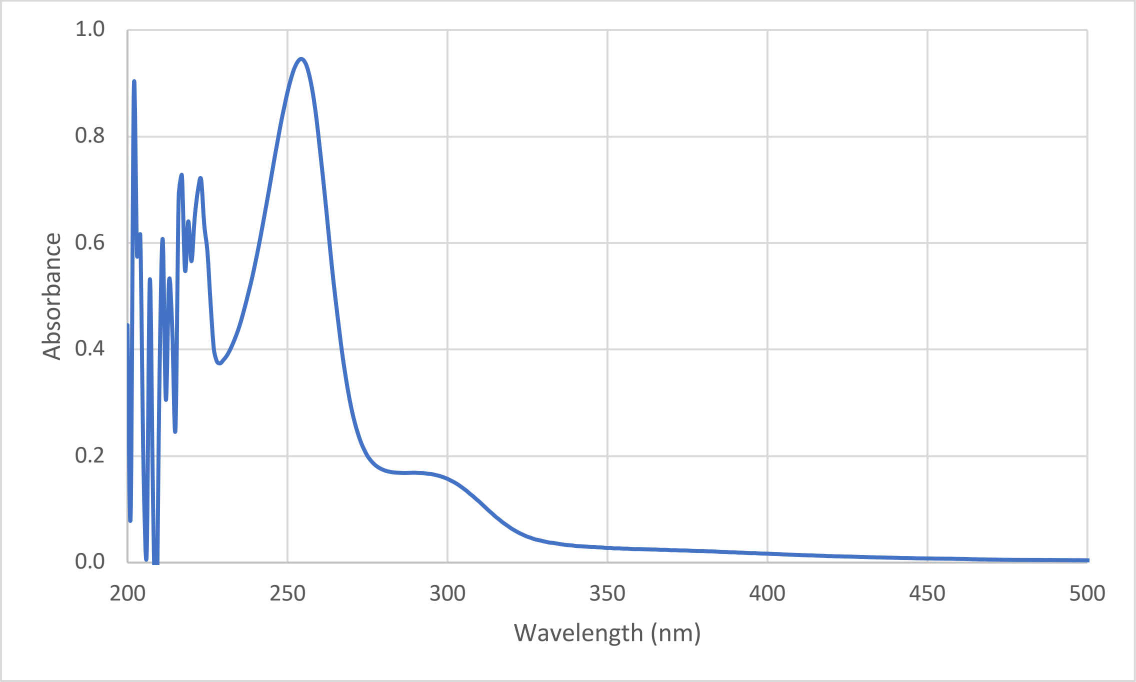

Fig.S.6: UV/vis spectrum of N1,N2-bis(4-chlorophenyl)ethane-1,2-diimine (**N-N[6]).**

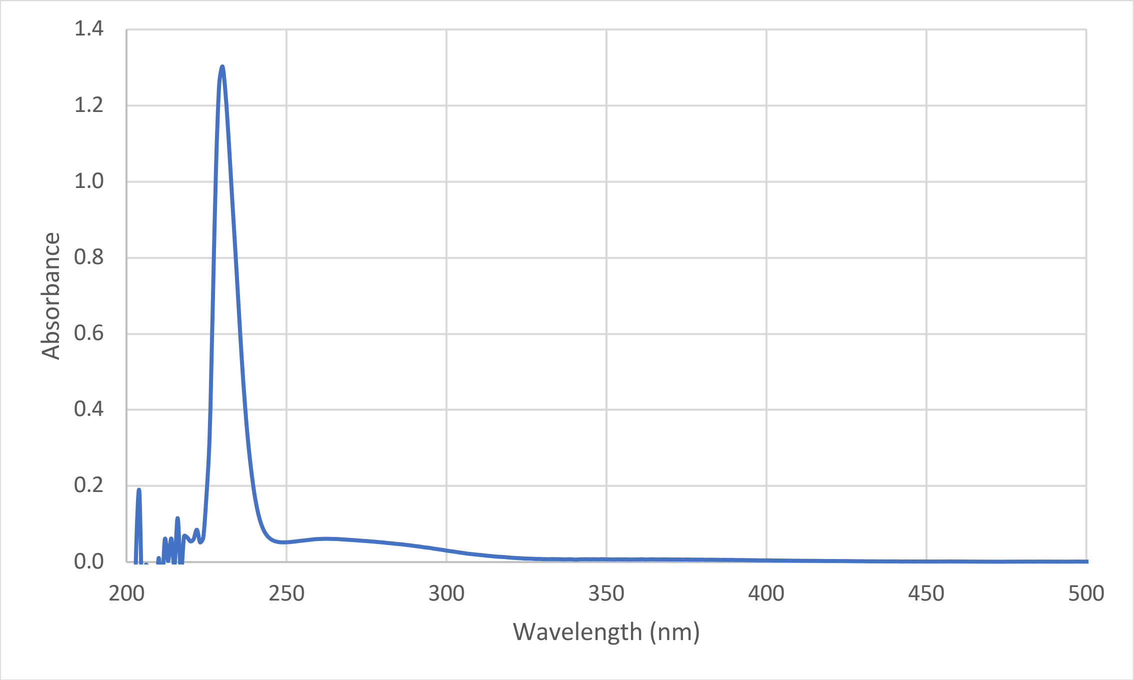

Fig.S.7: UV/vis spectrum of N1,N2-dicyclohexylethane-1,2-diimine (**N-N^7).**

FTIR of ligands

Table S.3: FTIR/ATR data of α-diimines.

Ligand	wavenumber (cm^{-1})	Vibrational modes assigned
**N-N^1**	3023, 2967, 1618, 1473	$\nu_{Csp2\text{-}H}$, $\nu_{Csp3\text{-}H}$, $\nu_{C=N}$, $\nu_{C=C}$
**N-N^2**	3008, 2945, 1596, 1490	$\nu_{Csp2\text{-}H}$, $\nu_{Csp3\text{-}H}$, $\nu_{C=N}$, $\nu_{C=C}$
**N-N^3**	3023, 2915, 1617, 1475	$\nu_{Csp2\text{-}H}$, $\nu_{Csp3\text{-}H}$, $\nu_{C=N}$, $\nu_{C=C}$
**N-N^4**	3064, 2961, 1626, 1460	$\nu_{Csp2\text{-}H}$, $\nu_{Csp3\text{-}H}$, $\nu_{C=N}$, $\nu_{C=C}$
**N-N^5**	3063, 2974, 1612, 1502, 1366-1305	$\nu_{Csp2\text{-}H}$, $\nu_{Csp3\text{-}H}$, $\nu_{C=N}$, $\nu_{C\text{-}F}$, $\nu_{C=C}$
**N-N^6**	3088-3026, 2971, 1606, 1483, 828-807	$\nu_{Csp2\text{-}H}$, $\nu_{Csp3\text{-}H}$, $\nu_{C=N}$, $\nu_{C=C}$, $\nu_{C\text{-}Cl}$
**N-N^7**	2923-2853, 1622, 1449	$\nu_{Csp3\text{-}H}$, $\nu_{C=N}$, $\nu_{ins\cdot cy.}$

N-N^1 = N1,N2-bis(2,6-dimethylphenyl)ethane-1,2-diimine; **N-N^2** = N1,N2-bis(2,4-dimethylphenyl)ethane-1,2-diimine; **N-N^3** = N1,N2-bis(2,4,6-trimethylphenyl)ethane-1,2-diimine; **N-N^4** = N1,N2-bis[2,6-bis(propan-2-yl)phenyl]ethane-1,2-diimine; **N-N^5** = N1,N2-bis(4-fluorophenyl)ethane-1,2-diimine; **N-N^6** = N1,N2-bis(4-chlorophenyl)ethane-1,2-diimine and **N-N^7** = N1,N2-dicyclohexylethane-1,2-diimine.

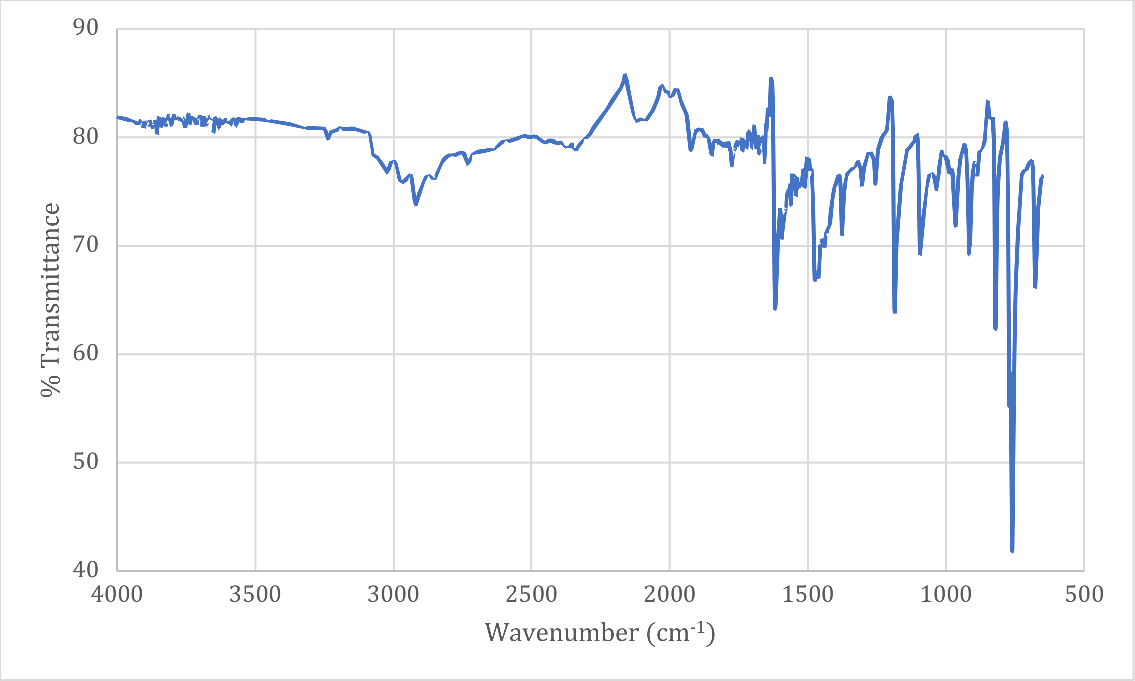

Fig.S.8: FTIR/ATR spectrum of N1,N2-bis(2,6-dimethylphenyl)ethane-1,2-diimine (**N-N**[1]).

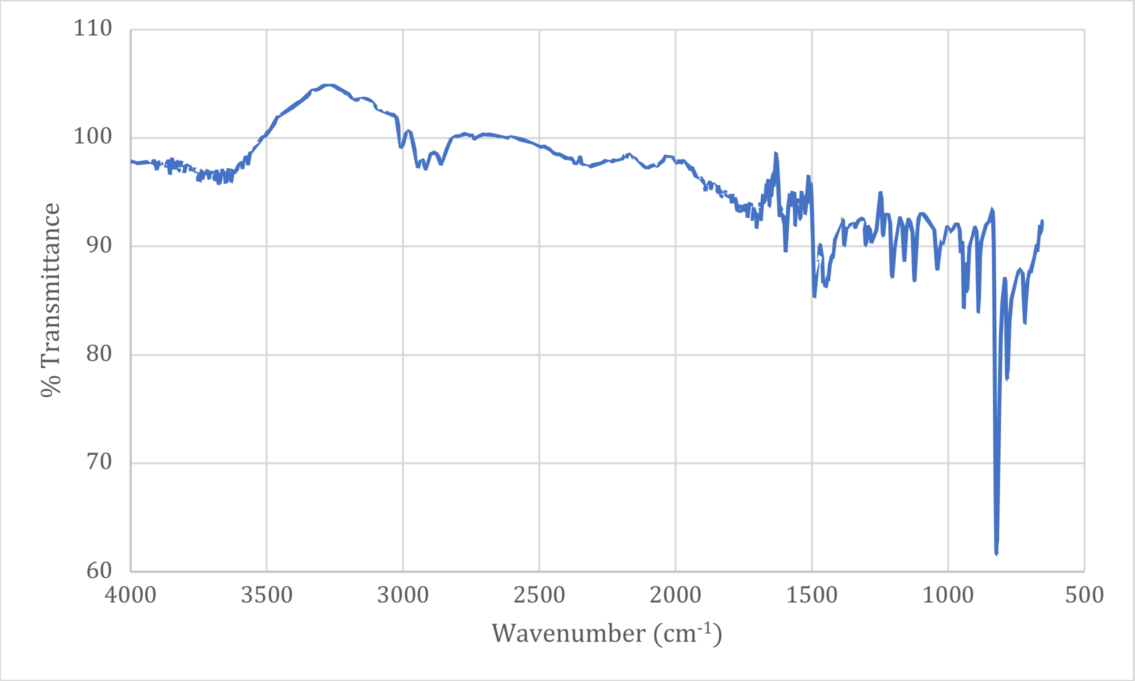

Fig.S.9: FTIR/ATR spectrum of N1,N2-bis(2,4-dimethylphenyl)ethane-1,2-diimine (**N-N**[2]**).**

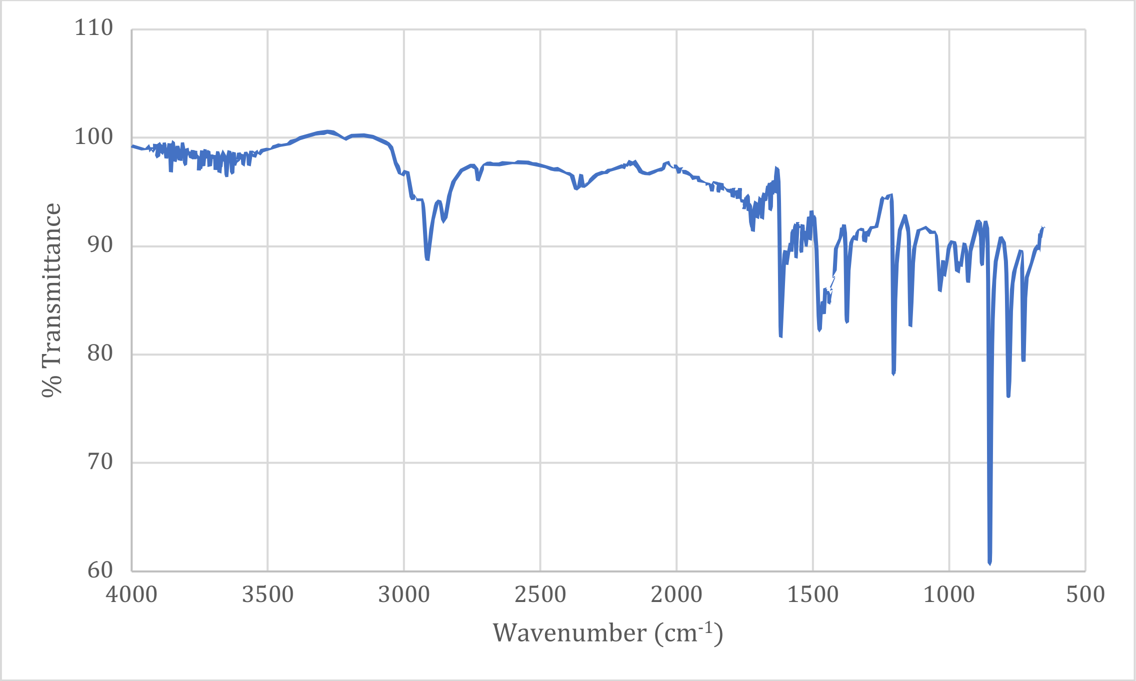

Fig.S.10: FTIR/ATR spectrum of N1,N2-bis(2,4,6-trimethylphenyl)ethane-1,2-diimine (**N-N^3**).

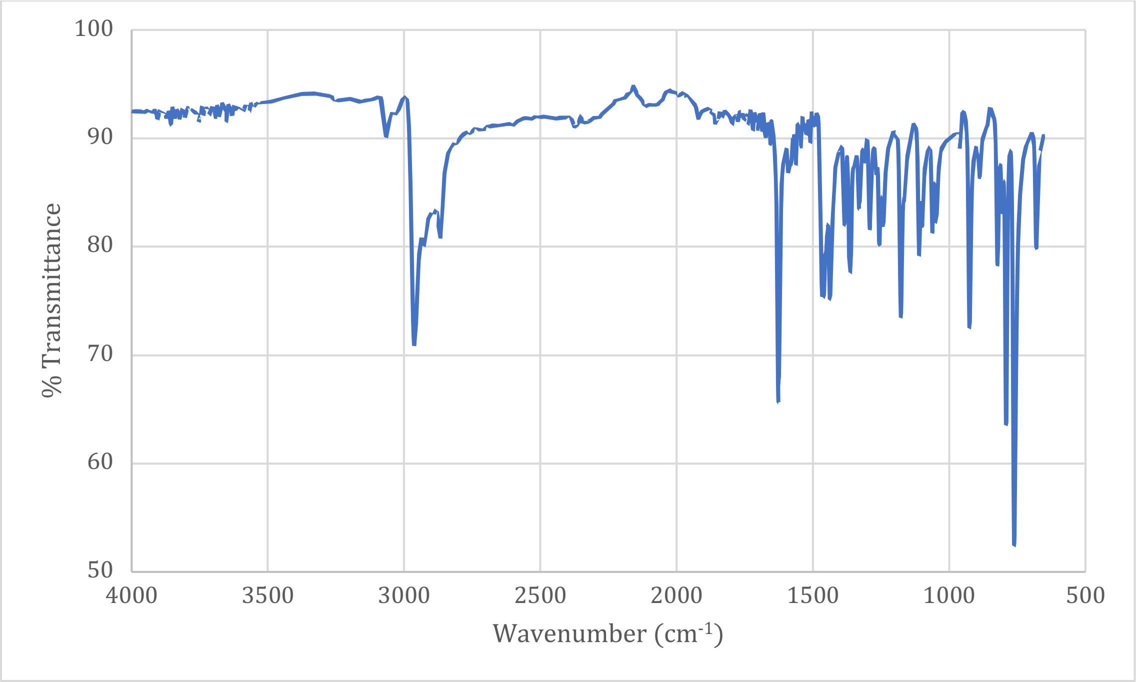

Fig.S.11: FTIR/ATR spectrum of N1,N2-bis[2,6-bis(propan-2-yl)phenyl]ethane-1,2-diimine (**N-N^4**).

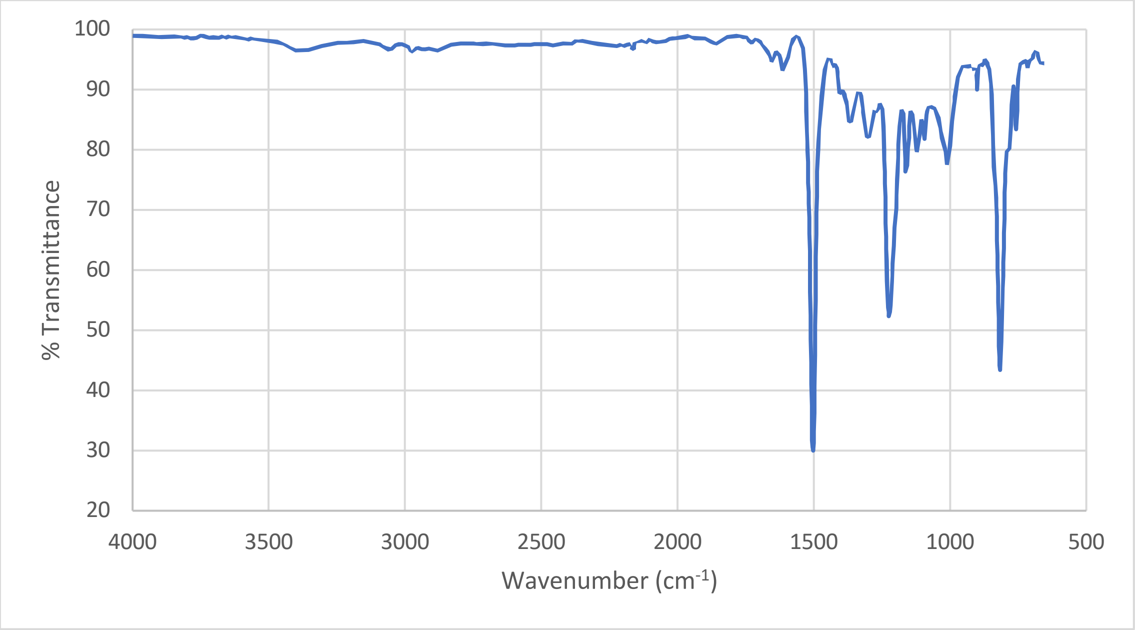

Fig.S.12: FTIR/ATR spectrum of N1,N2-bis(4-fluorophenyl)ethane-1,2-diimine (**N-N[5]**).

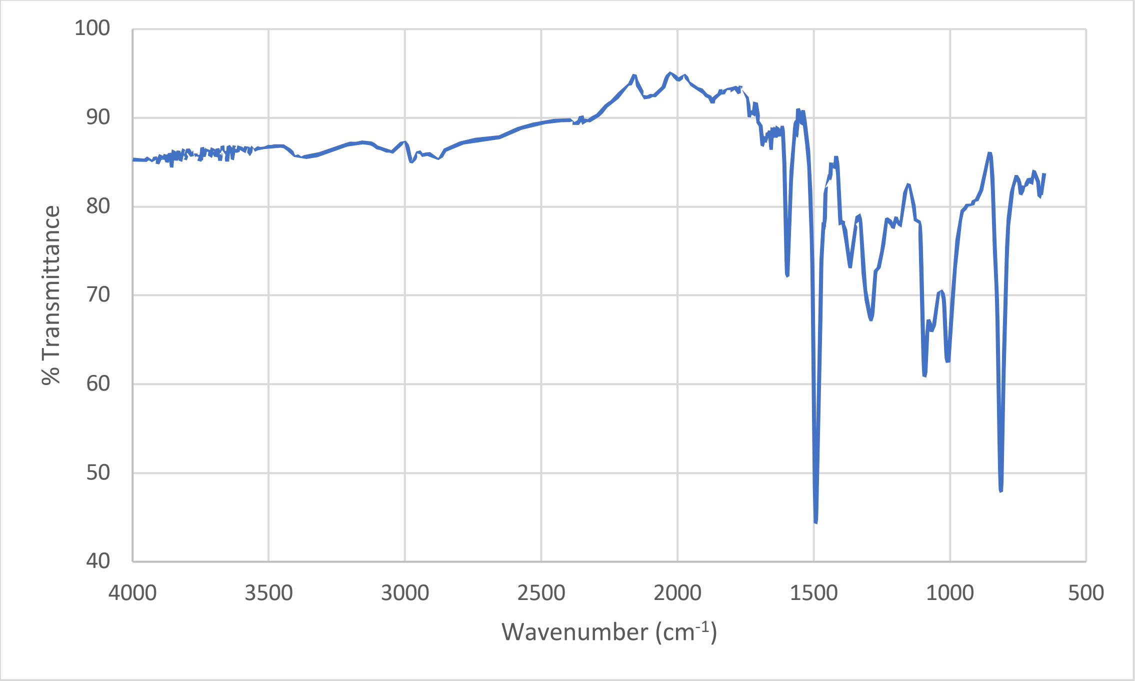

Fig.S.13: FTIR/ATR spectrum of N1,N2-bis(4-chlorophenyl)ethane-1,2-diimine (**N-N[6]**).

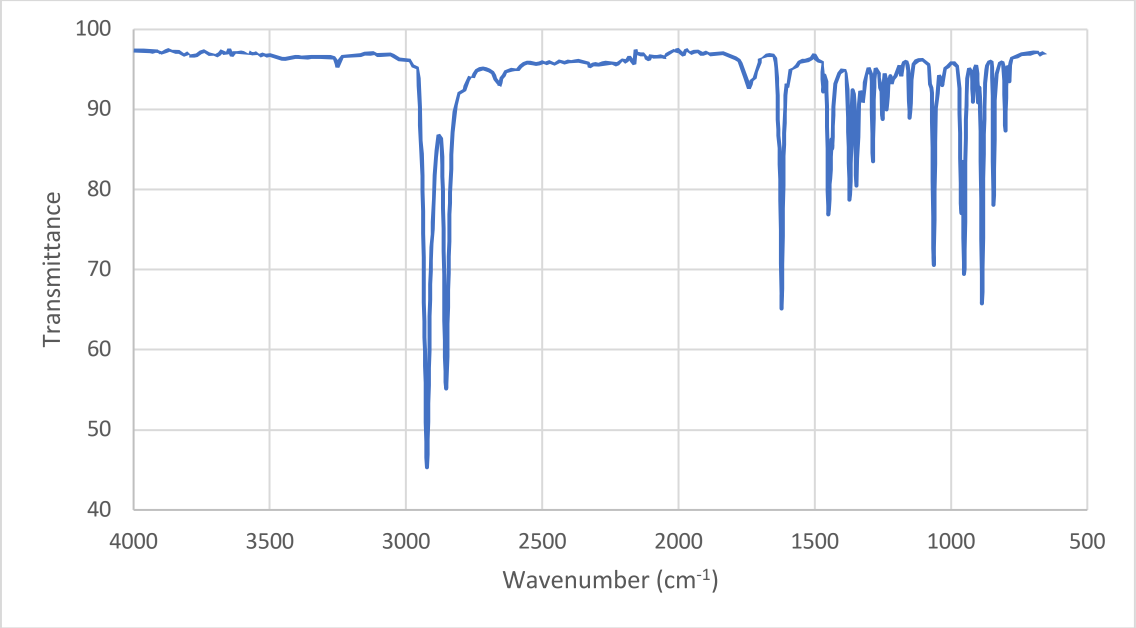

Fig.S.14: FTIR/ATR spectrum of N1,N2-dicyclohexylethane-1,2-diimine (**N-N[7]**).

^{1}H and ^{13}C NMR data of ligands

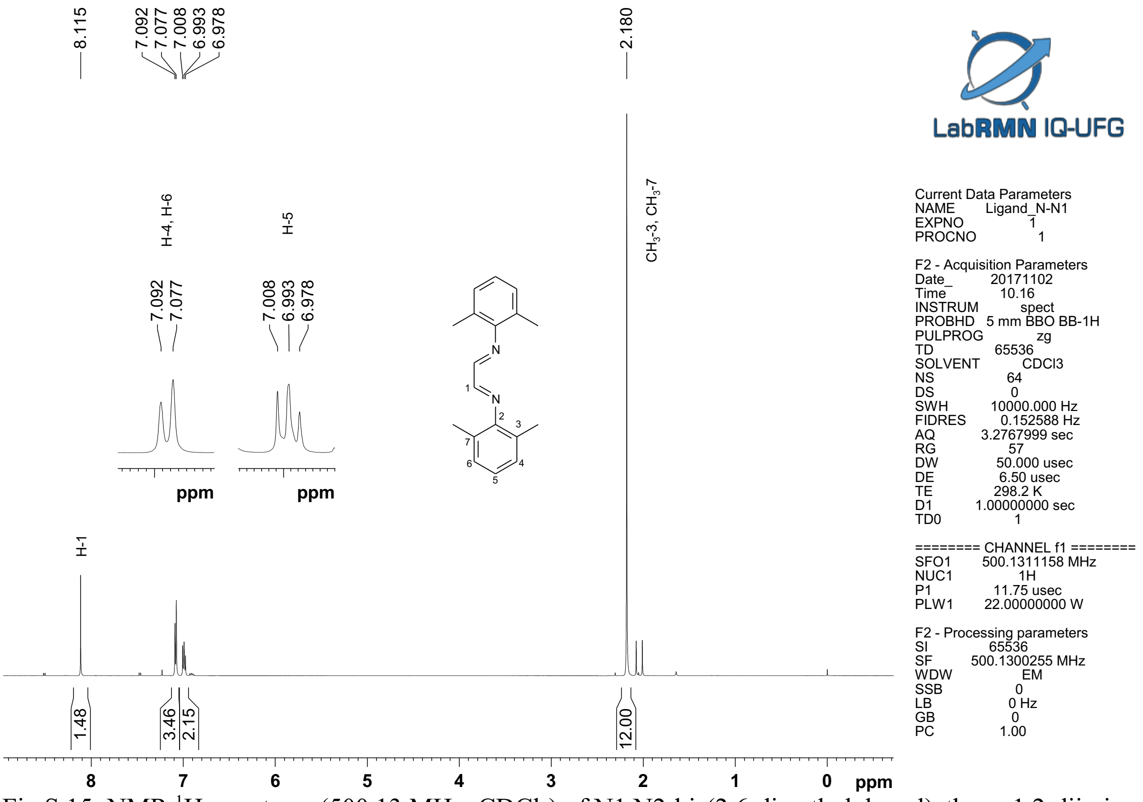

Fig.S.15: NMR ^{1}H spectrum (500.13 MHz, $CDCl_3$) of N1,N2-bis(2,6-dimethylphenyl)ethane-1,2-diimine (**N-N^{1}**).

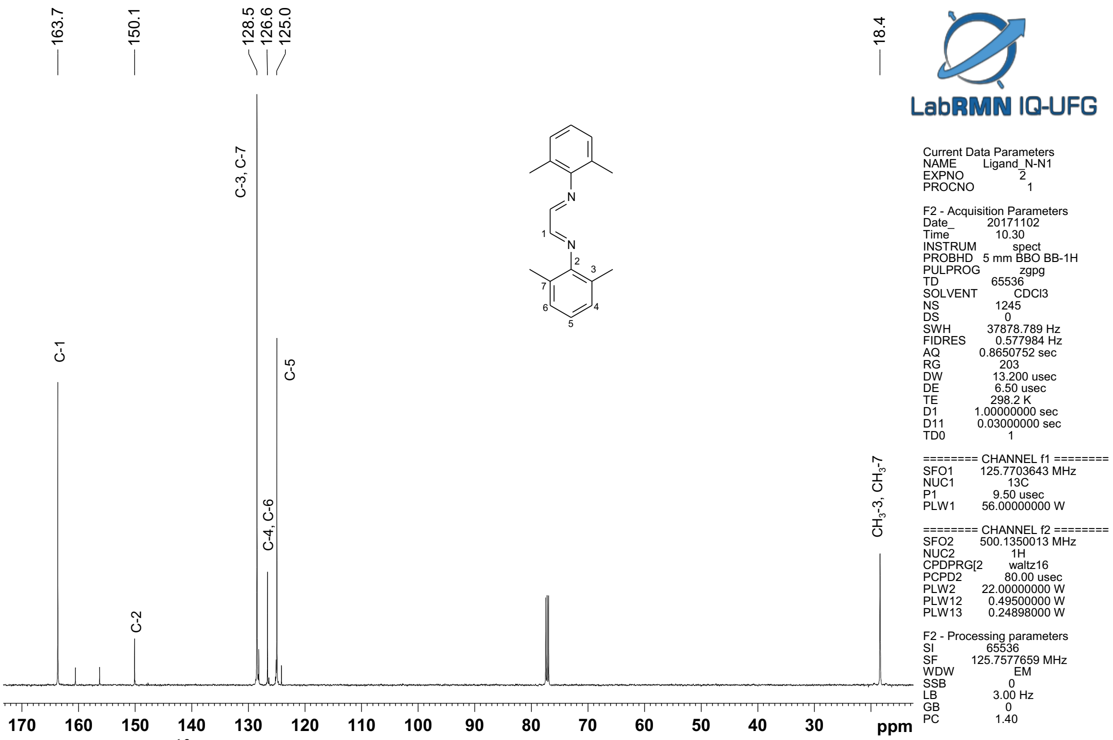

Fig.S.16: NMR ^{13}C spectrum (127.75 MHz, $CDCl_3$) of N1,N2-bis(2,6-dimethylphenyl)ethane-1,2-diimine (**N-N^{1}**).

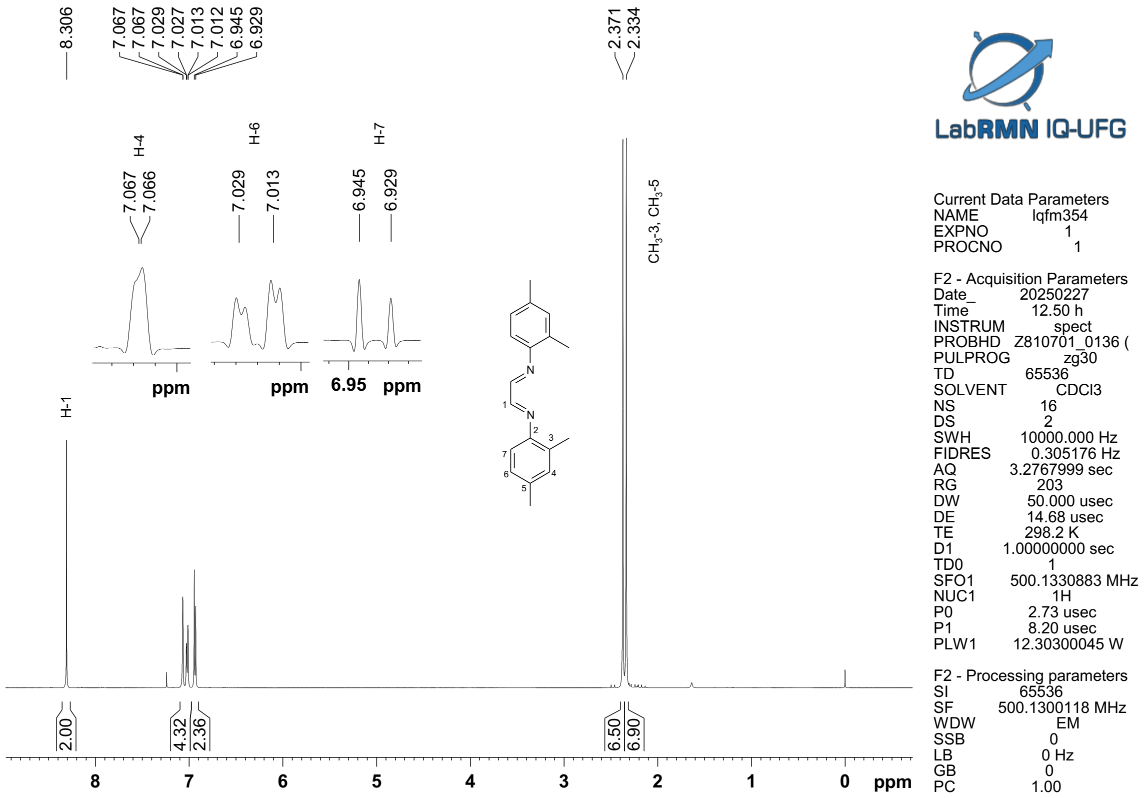

Fig.S.17: NMR ^{1}H spectrum (500.13 MHz, $CDCl_3$) of N1,N2-bis(2,4-dimethylphenyl)ethane-1,2-diimine (**N-N**^{2})

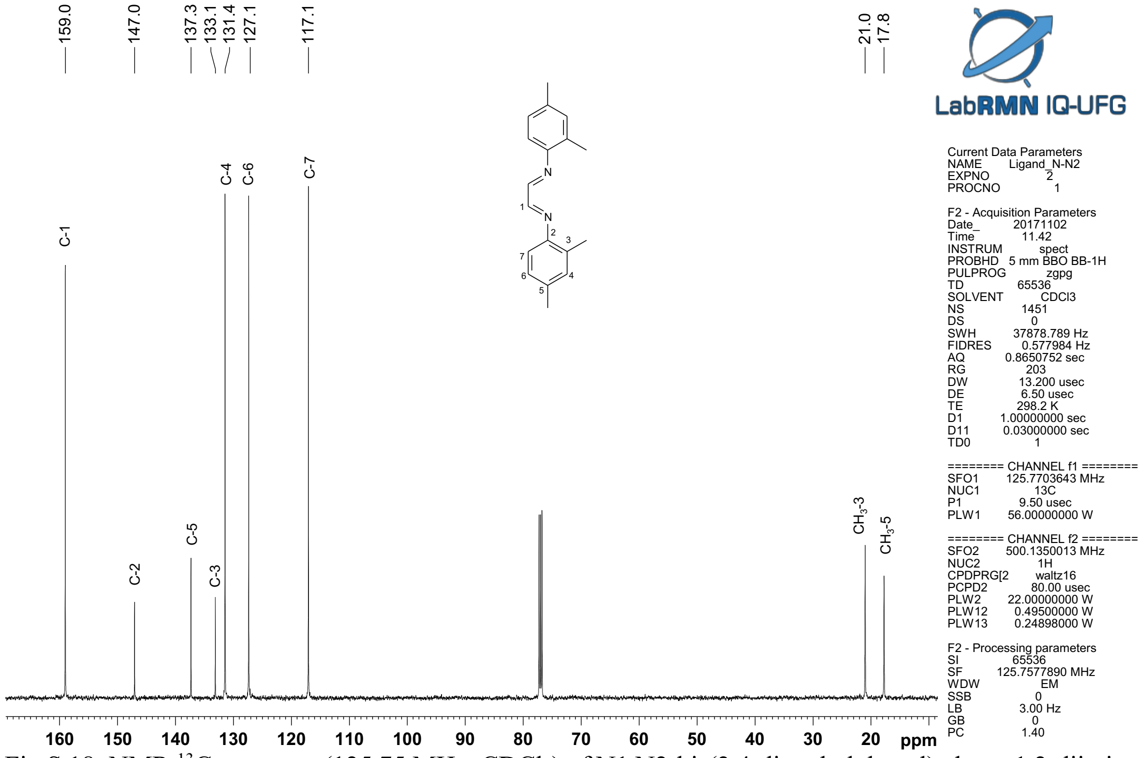

Fig.S.18: NMR ^{13}C spectrum (125.75 MHz, $CDCl_3$) of N1,N2-bis(2,4-dimethylphenyl)ethane-1,2-diimine (**N-N**^{2}).

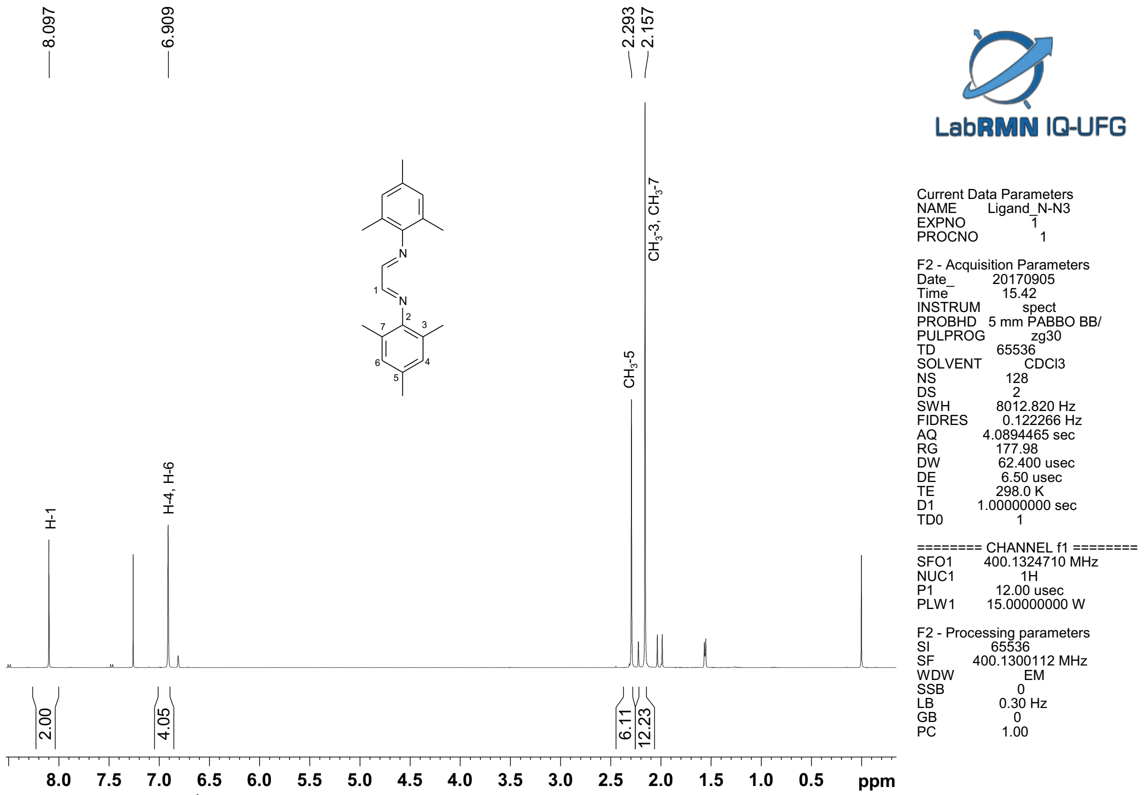

Fig.S.19: NMR ^{1}H spectrum (500.13 MHz, $CDCl_3$) of N1,N2-bis(2,4,6-trimethylphenyl)ethane-1,2-diimine (**N-N^3**).

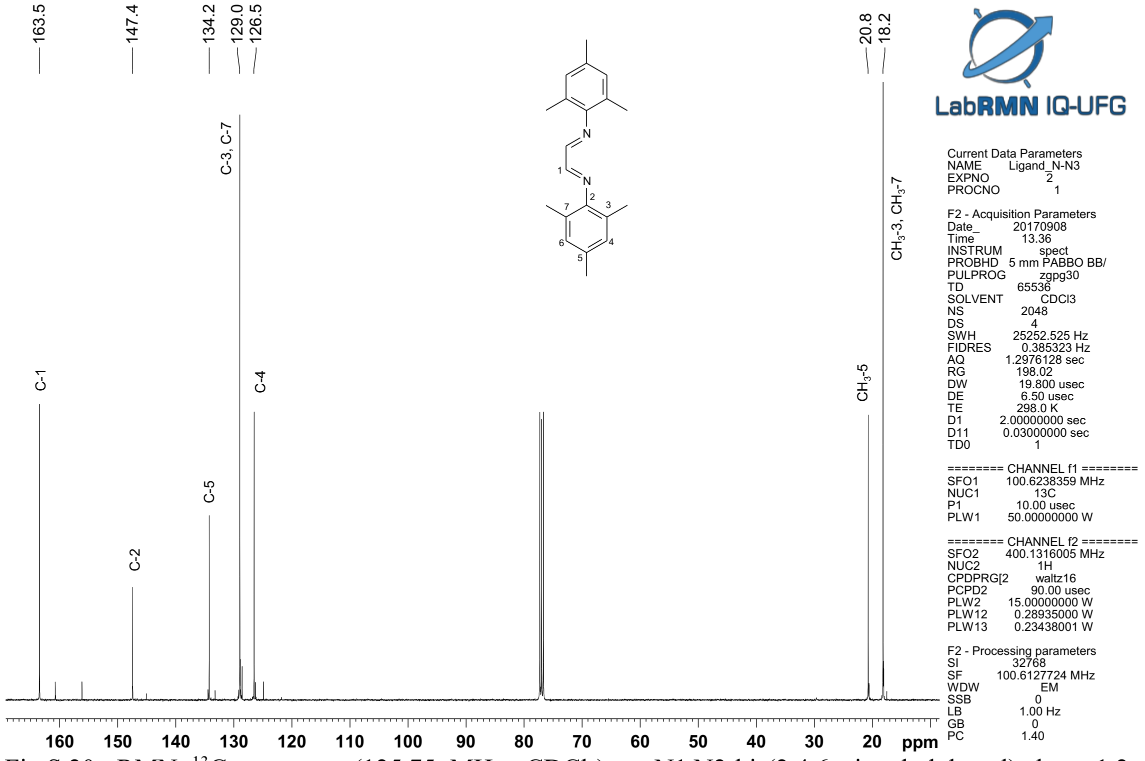

Fig.S.20: RMN ^{13}C spectrum (125.75 MHz, $CDCl_3$) to N1,N2-bis(2,4,6-trimethylphenyl)ethane-1,2-diimine (**N-N^3**).

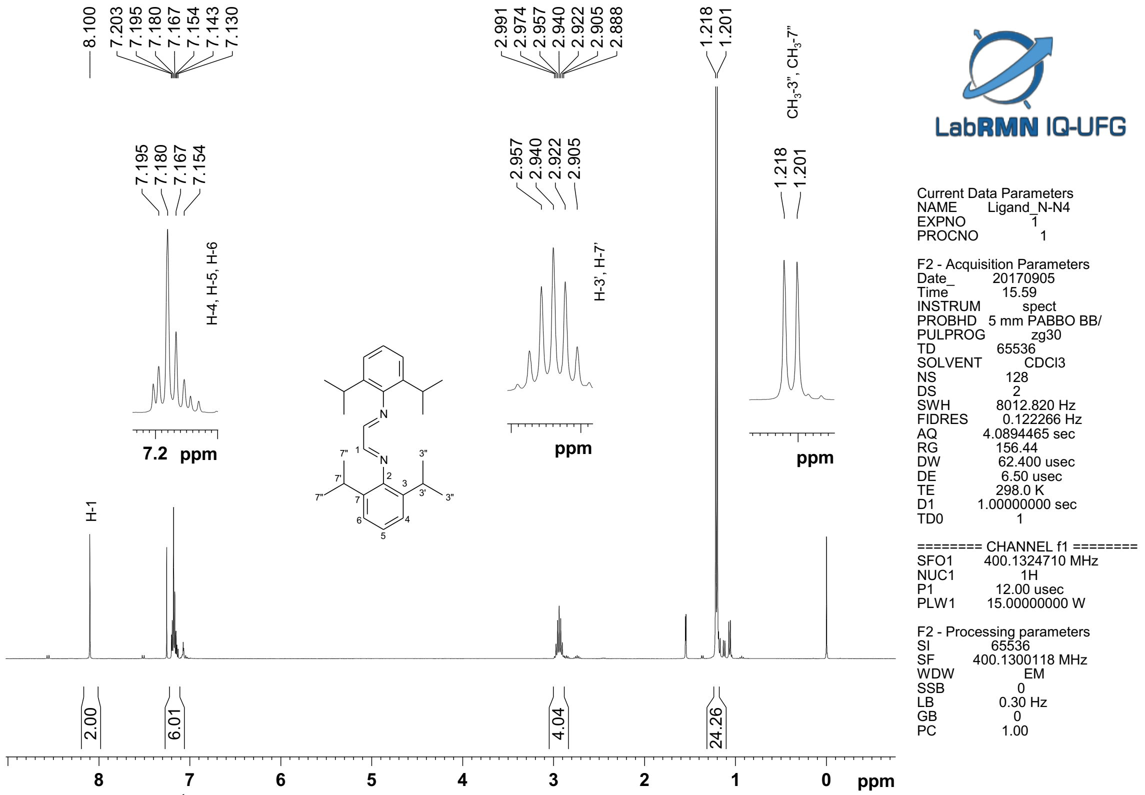

Fig.S.21: NMR ^{1}H spectrum (500.13 MHz, $CDCl_3$) of N1,N2-bis[2,6-bis(propan-2-yl)phenyl]ethane-1,2-diimine (**N-N**^{4}).

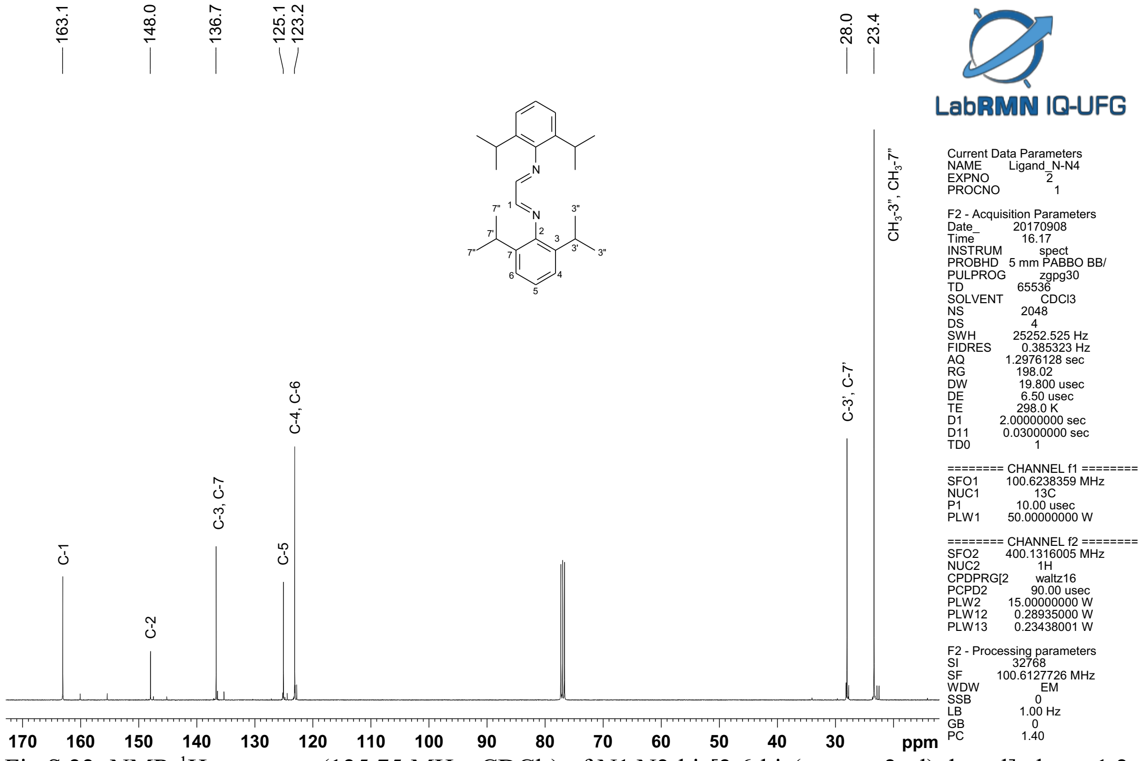

Fig.S.22: NMR ^{1}H spectrum (125.75 MHz, $CDCl_3$) of N1,N2-bis[2,6-bis(propan-2-yl)phenyl]ethane-1,2-diimine (**N-N**^{4}).

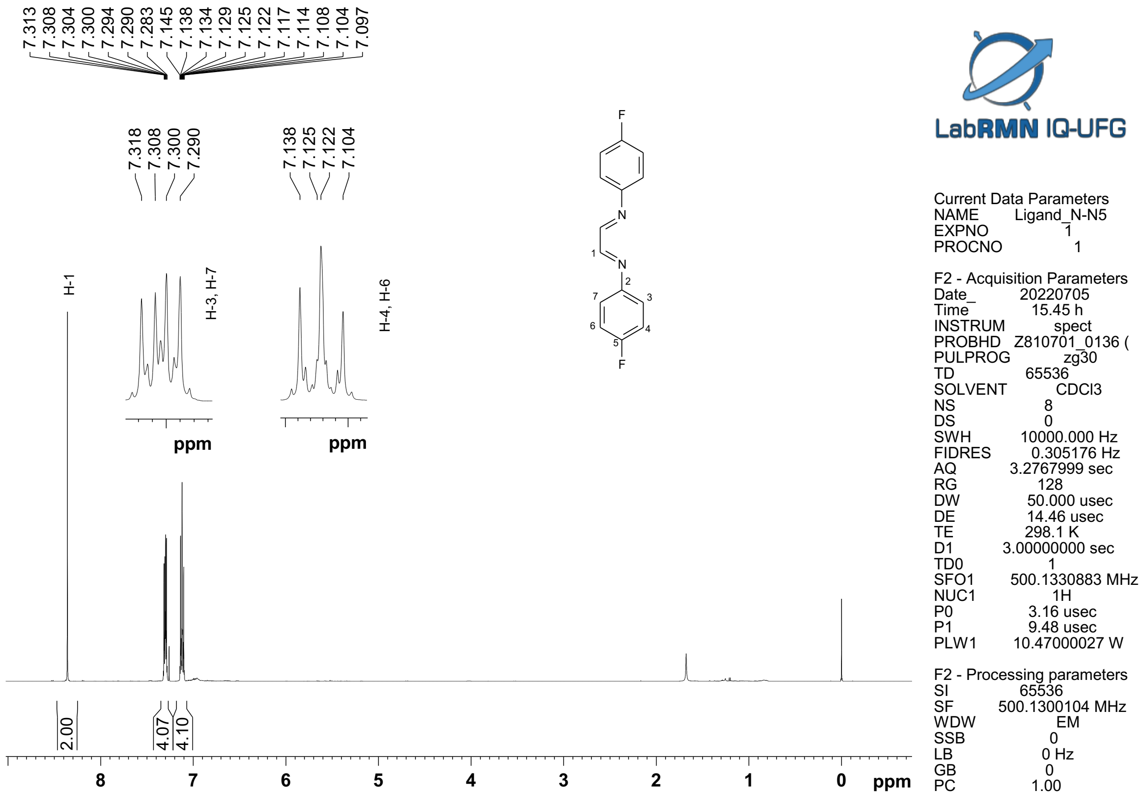

Fig.S.23: ^{1}H NMR spectrum (500.13 MHz, $CDCl_3$) of N1,N2-bis(4-fluorophenyl)ethane-1,2-diimine (**N-N^5**).

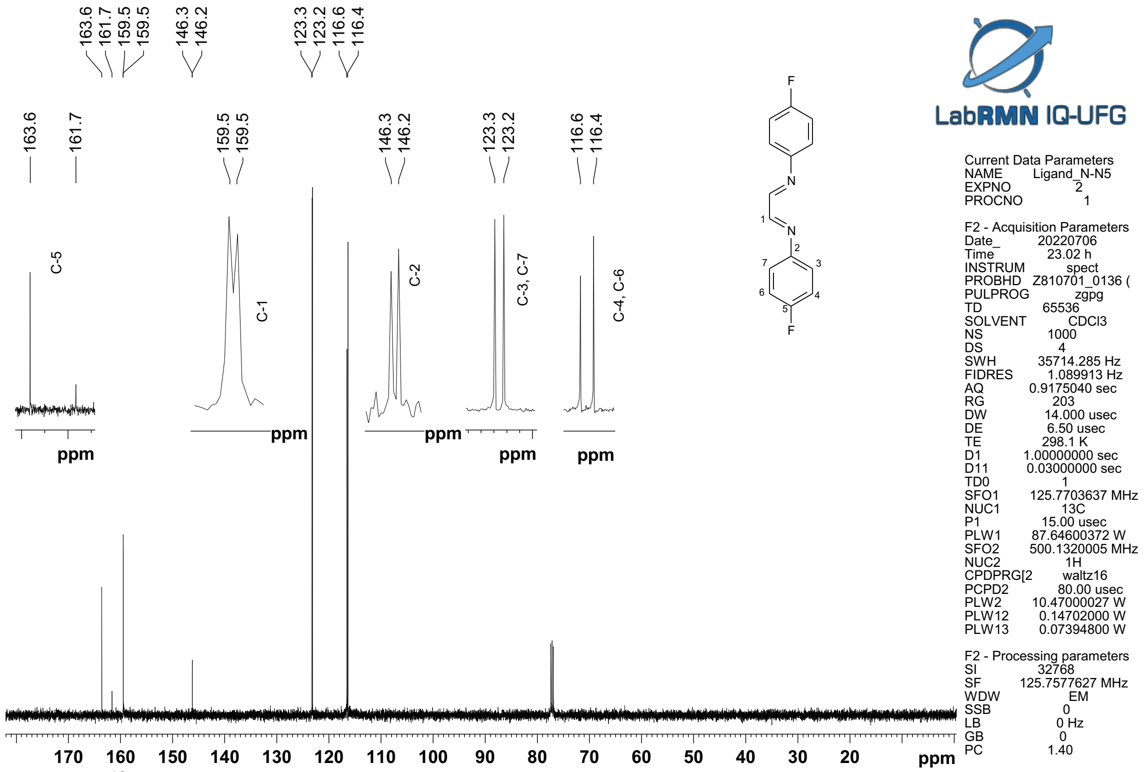

Fig.S.24: ^{13}C NMR spectrum (125.75 MHz, $CDCl_3$) of N1,N2-bis(4-fluorophenyl)ethane-1,2-diimine (**N-N^5**).

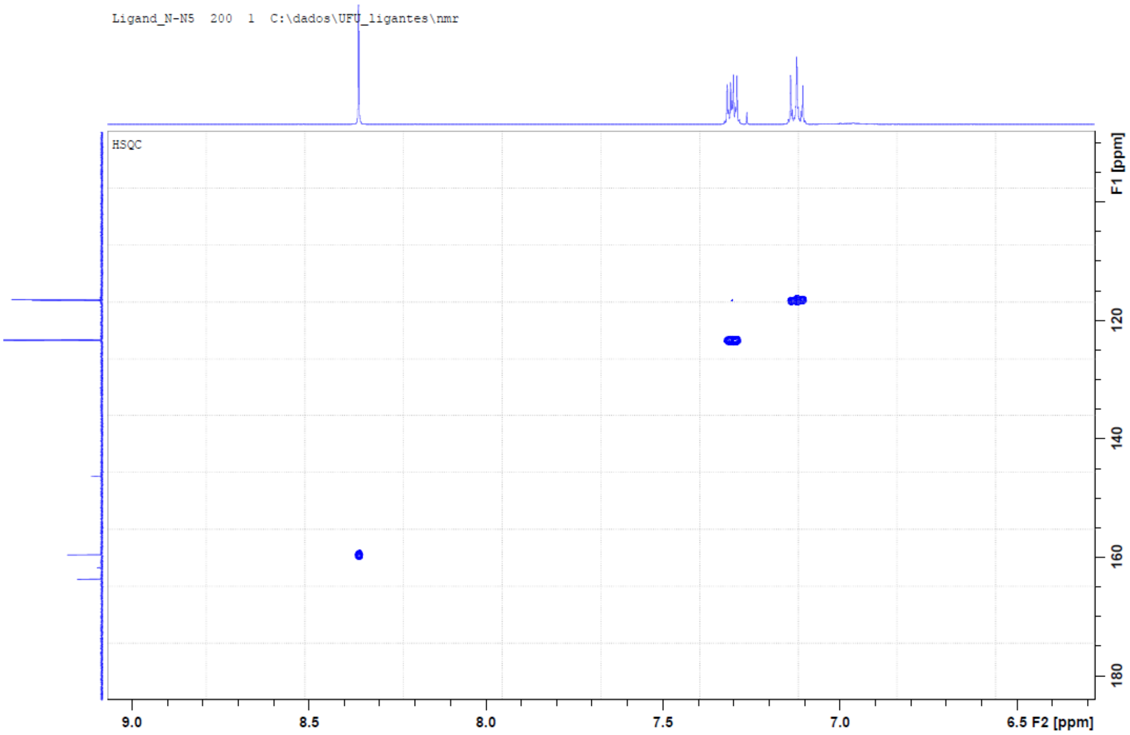

Fig. S.25: ^{1}H-^{13}C HSQC NMR contour map for N1,N2-bis(4-fluorophenyl)ethane-1,2-diimine (**N-N^5**) (500 and 125 MHz, $CDCl_3$).

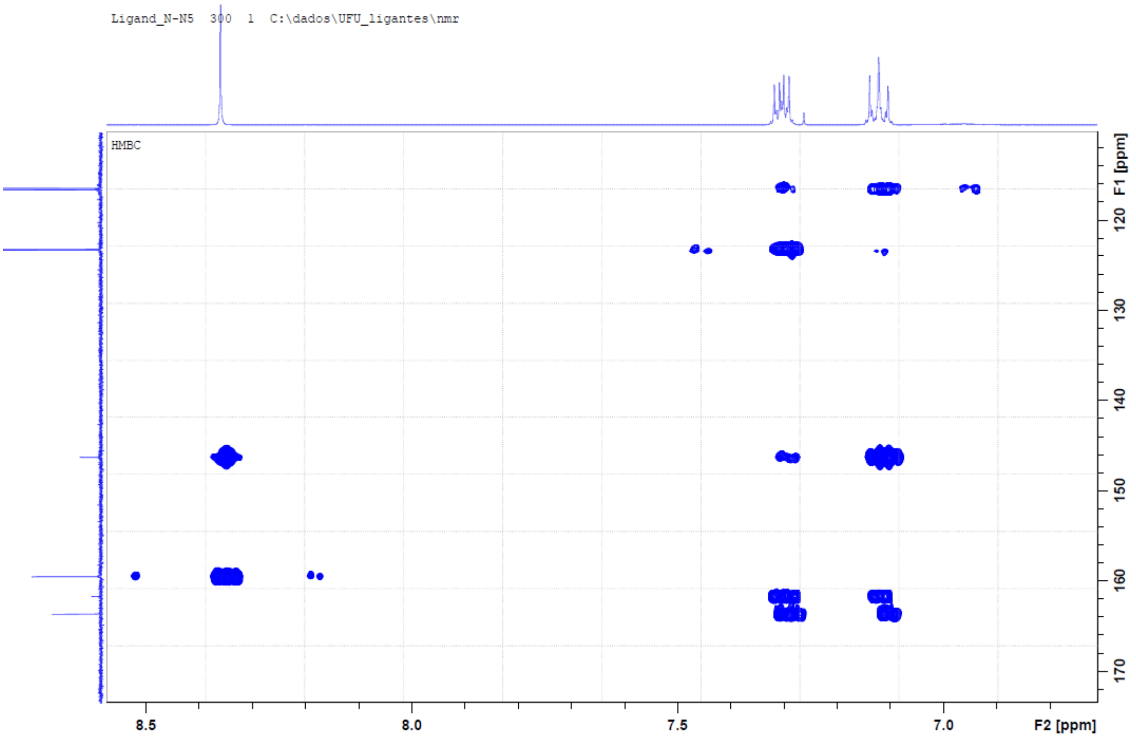

Fig. S.26: ^{1}H-^{13}C HMBC NMR contour map for N1,N2-bis(4-fluorophenyl)ethane-1,2-diimine (**N-N^5**) (500 and 125 MHz, $CDCl_3$).

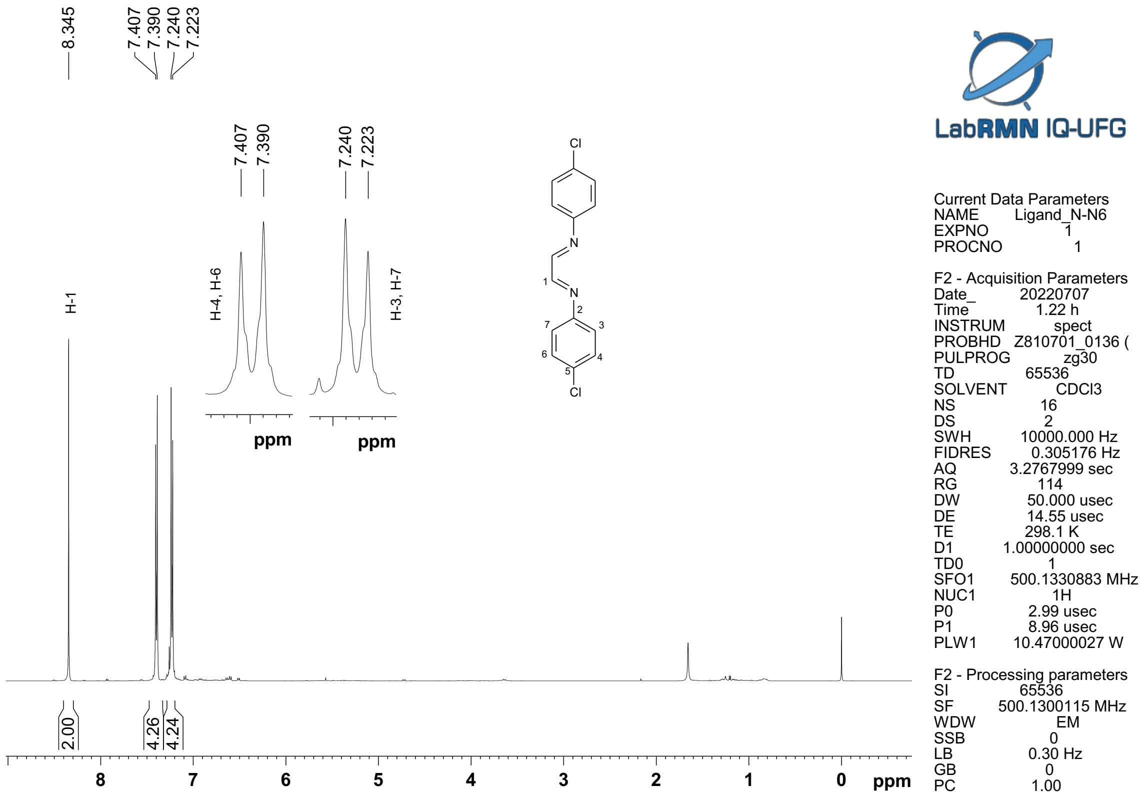

Fig.S.27: ^{1}H NMR spectrum (500.13 MHz, $CDCl_3$) of N1,N2-bis(4-chlorophenyl)ethane-1,2-diimine (**N-N^6**).

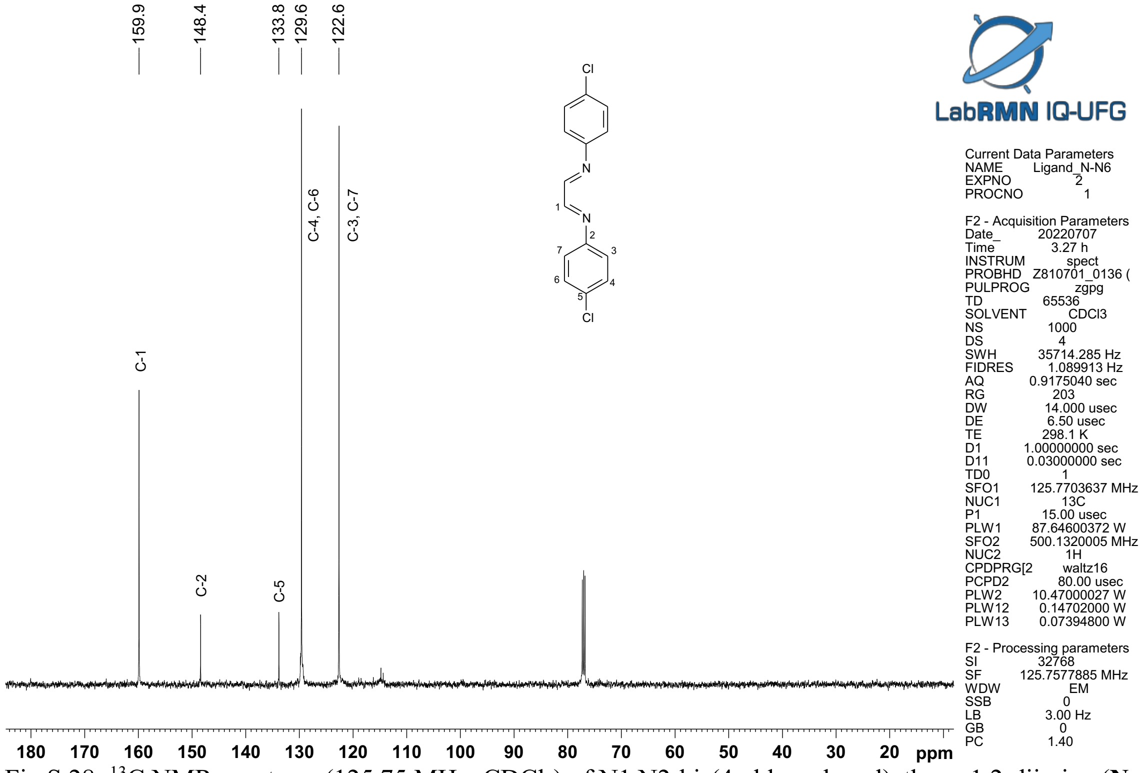

Fig.S.28: ^{13}C NMR spectrum (125.75 MHz, $CDCl_3$) of N1,N2-bis(4-chlorophenyl)ethane-1,2-diimine (**N-N^6**).

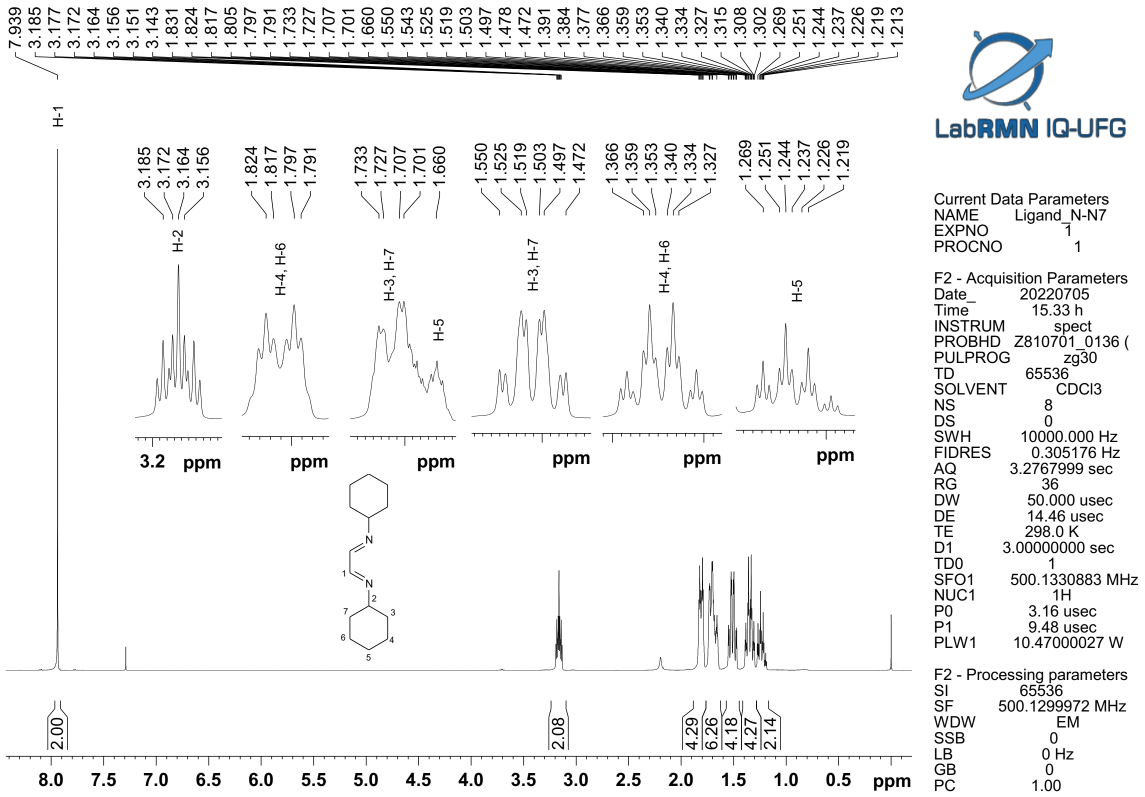

Fig.S.29: ^{1}H NMR spectrum (500.13 MHz, $CDCl_3$) of N1,N2-dicyclohexylethane-1,2-diimine (**N-N**^{7}).

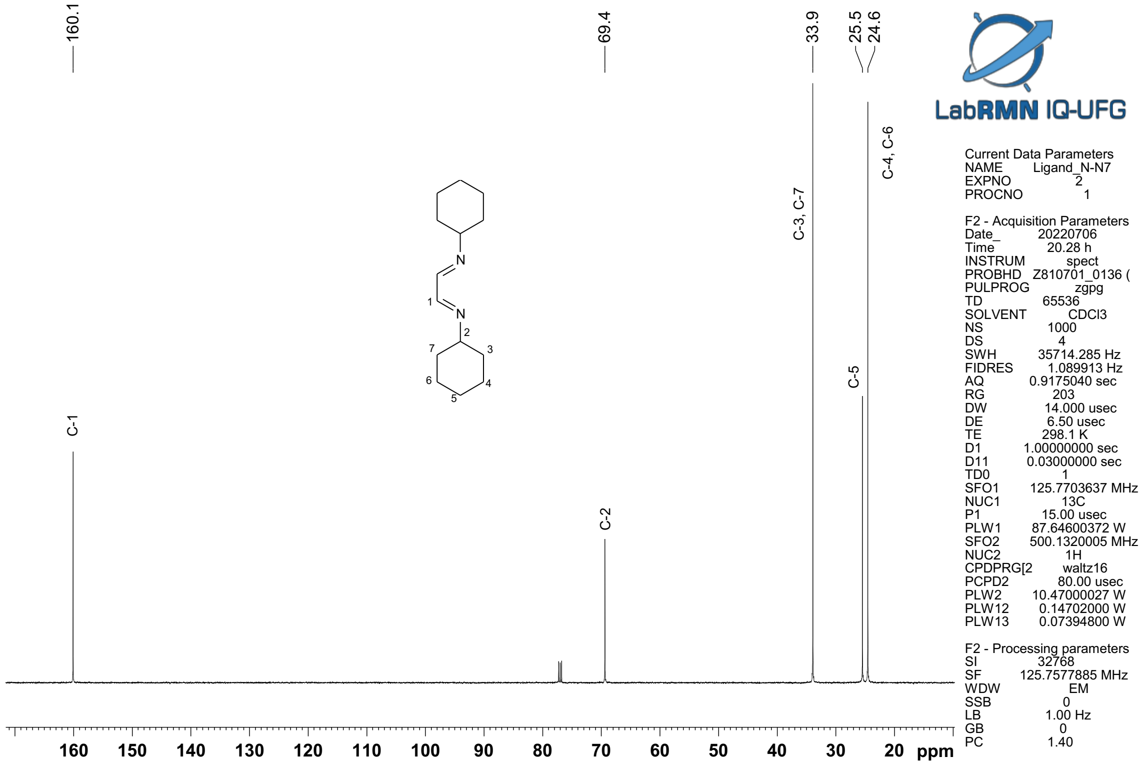

Fig.S.30: ^{13}C NMR spectrum (125.75 MHz, $CDCl_3$) of N1,N2-dicyclohexylethane-1,2-diimine (**N-N**^{7}).

Elemental Analysis of Complexes.

Table S.4: Elemental Analysis of complexes **1** – **7**.

Complex	% C found (theoretical)	% H found (theoretical)	% N found (theoretical)
**1**	49.40 (49.45)	5.23 (5.04)	4.03 (4.12)
**2**	49.58 (49.45)	4.78 (5.04)	4.62 (4.12)
**3**	49.91 (50.08)	5.41 (5.08)	3.94 (3.96)
**4**	44.99 (45.48)	4.83 (5.38)	3.99 (3.54)
**5**	43.29(43.68)	3.59 (3.67)	4.28 (4.24)
**6**	40.95 (41.01)	4.72 (4.88)	4.01 (3.99)
**7**	44.98 (45.32)	5.99 (6.02)	4.25 (4.40)

Conductivity

Table S.5: Ionic molar conductivity for **1** – **7** (concentration 1.0 × 10^{-3} mol L^{-1}).

Complex	Ionic molar conductivity at 25 °C (ohm^{-1} cm^2 mol^{-1})	
	Acetonitrile	Dichloromethane
**1**	153.9	20.4
**2**	155.6	21.5
**3**	160.1	22.4
**4**	163.2	25.2
**5**	130.7	9.3
**6**	134.4	7.8
**7**	75.3	16.1

UV/vis data of complexes

Table S.6: UV/vis data of complexes **1** – **7** and suggested band assignment

Complex	λ (nm)	log ε (L mol^{-1} cm^{-1})	Transition
**1**	346	3.23	(IL) π → π*
	435	2.99	(TCML) *d*π → π*
**2**	230	3.99	(IL) π → π*
	286	3.54	(IL) π → π*
	399	3.59	(TCML) *d*π → π*
**3**	281	4.07	(IL) π → π*
	364	3.60	(IL) π → π*
	436	3.67	(TCML) *d*π → π*
**4**	230	4.31	(IL) π → π*
	344	3.44	(IL) π → π*
	488	3.60	(TCML) *d*π → π*
**5**	285	3.82	(IL) π → π*
	358	3.76	(IL) π → π*
	441	3.43	(TCML) *d*π → π*
**6**	304	3.80	(IL) π → π*
	375	3.82	(IL) π → π*
	465	3.41	(TCML) *d*π → π*
**7**	231	4.21	(IL) π → π*
	288	3.68	(IL) π → π*
	369	3.50	(IL) π → π*
	430	3.61	(TCML) *d*π → π*

IL = Intraligand. TCML = Transfer charge metal ligand.

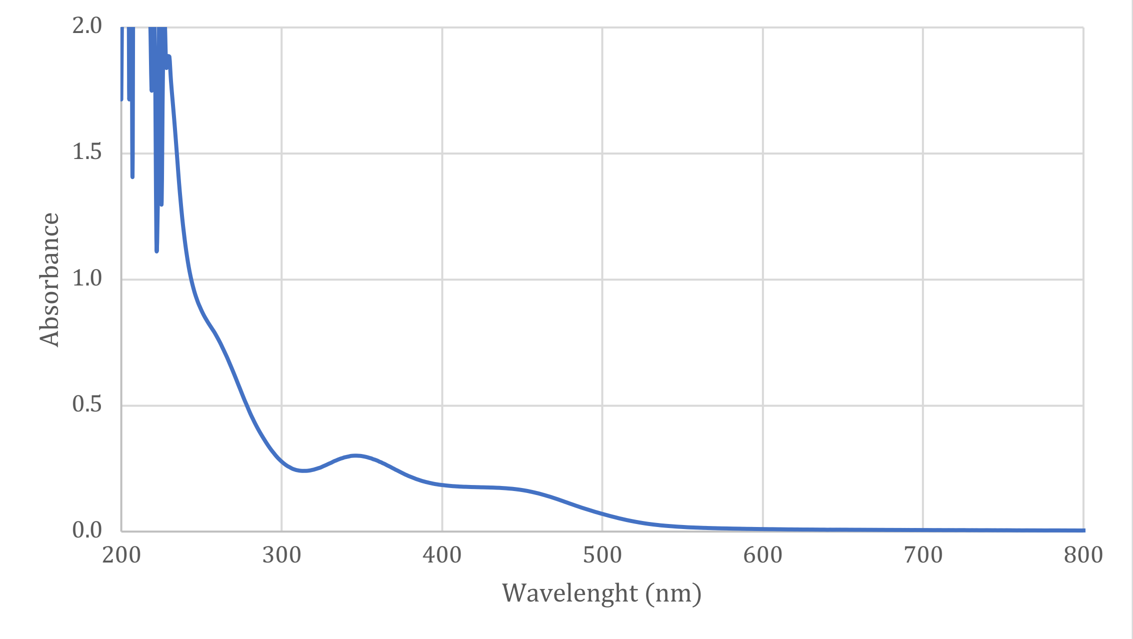

Fig.S.31: UV/vis spectrum of [RuCl(*p*-cymene)(**N-N^{1}**)]PF_6 in CH_2Cl_2 (1.79 × 10^{-4} mol L^{-1}) at 25°C.

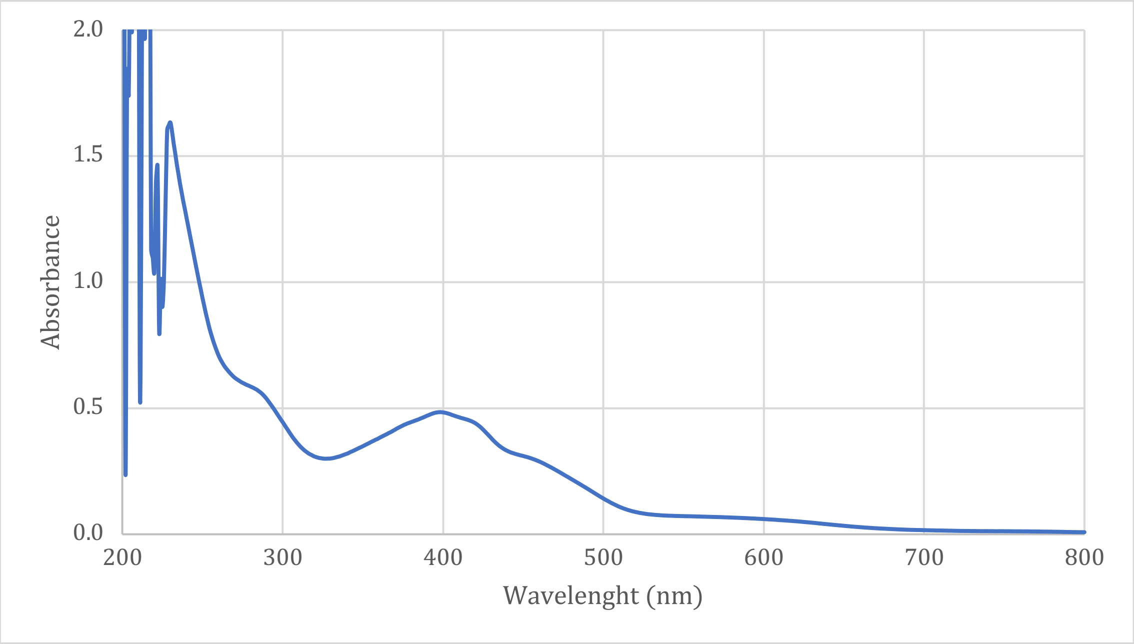

Fig.S.32: UV/vis spectrum of [RuCl(*p*-cymene)(**N-N**^{2})]PF_6 in CH_2Cl_2 (1.38 × 10^{-4} mol L^{-1}) at 25°C.

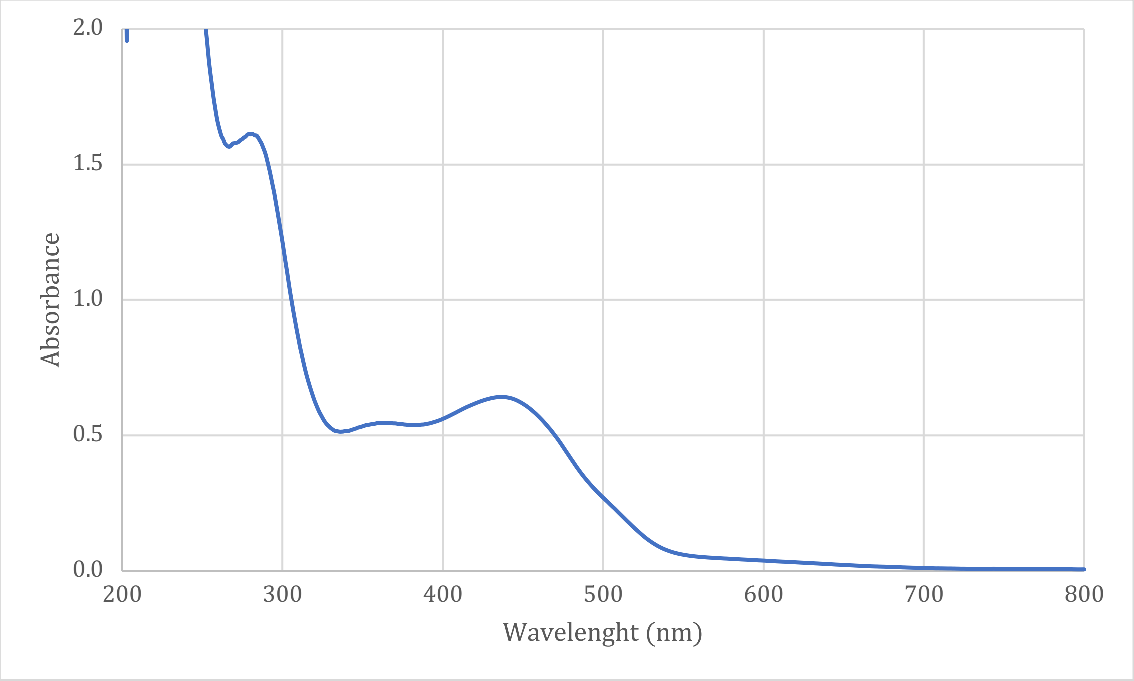

Fig.S.33: UV/vis spectrum of [RuCl(*p*-cymene)(**N-N**^{3})]PF_6 in CH_2Cl_2 (1.38 × 10^{-4} mol L^{-1}) at 25°C.

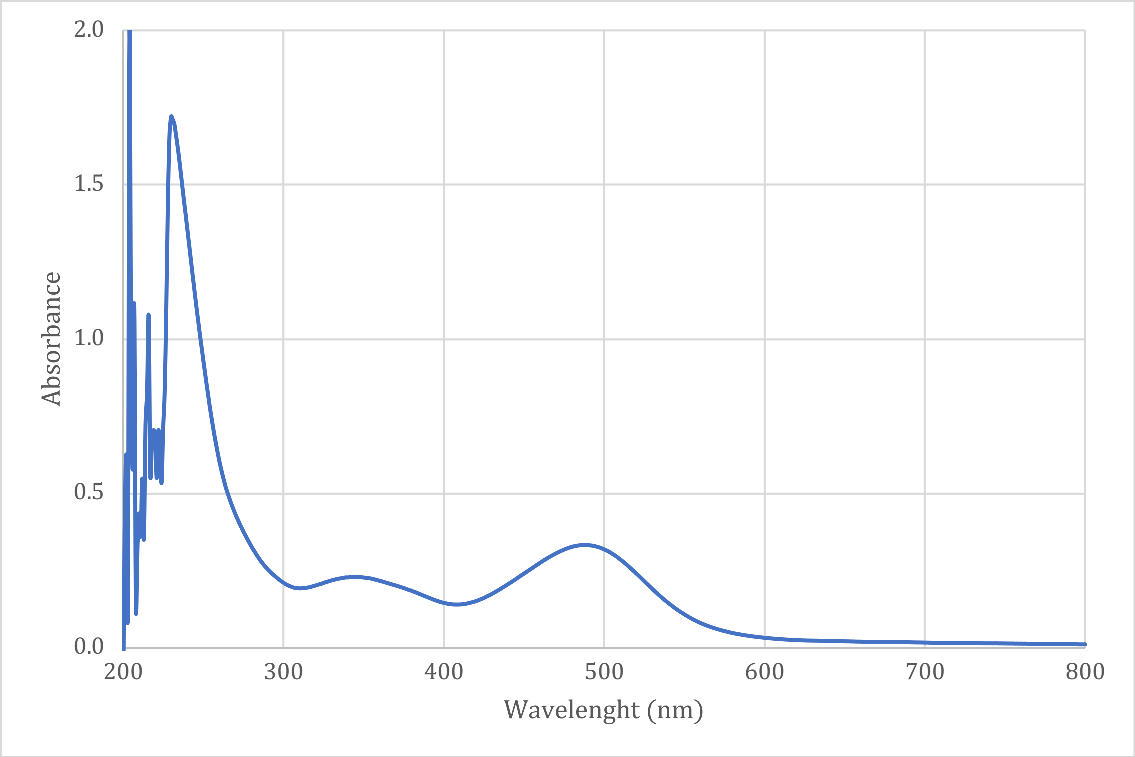

Fig.S.34: UV/vis spectrum of [RuCl(*p*-cymene)(**N-N**[4])]PF_6 in CH_2Cl_2 (8.38×10^{-5} mol L^{-1}) at 25°C.

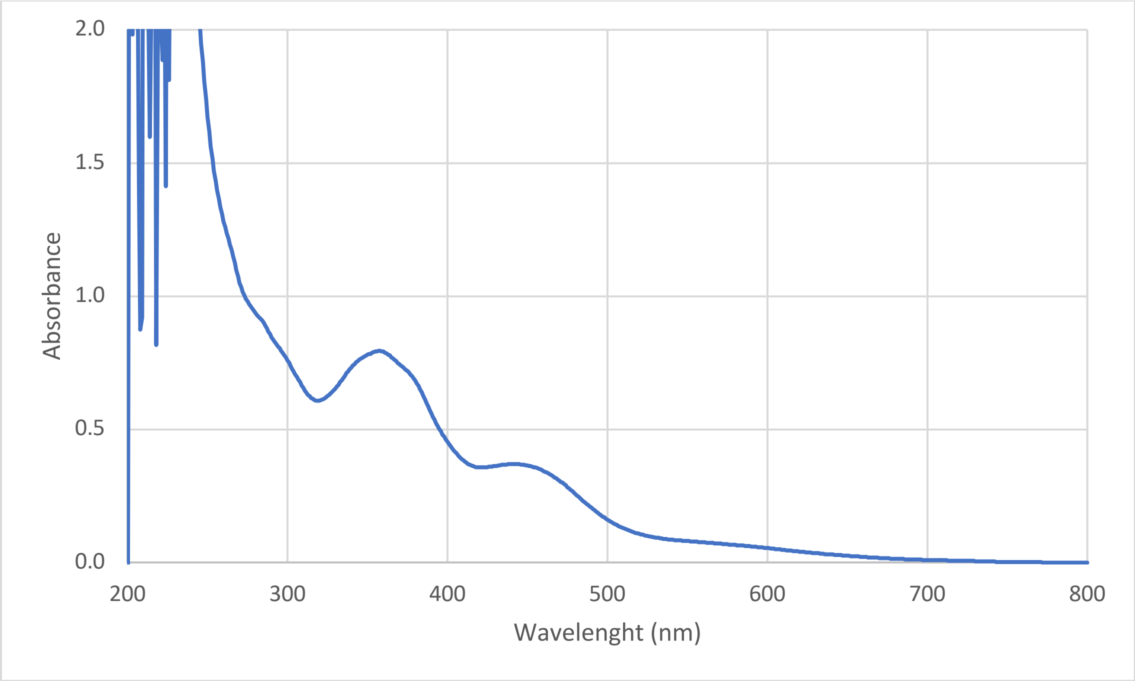

Fig.S.35: UV/vis spectrum of [RuCl(*p*-cymene)(**N-N**[5])]PF_6 in CH_2Cl_2 (1.38×10^{-4} mol L^{-1}) at 25°C.

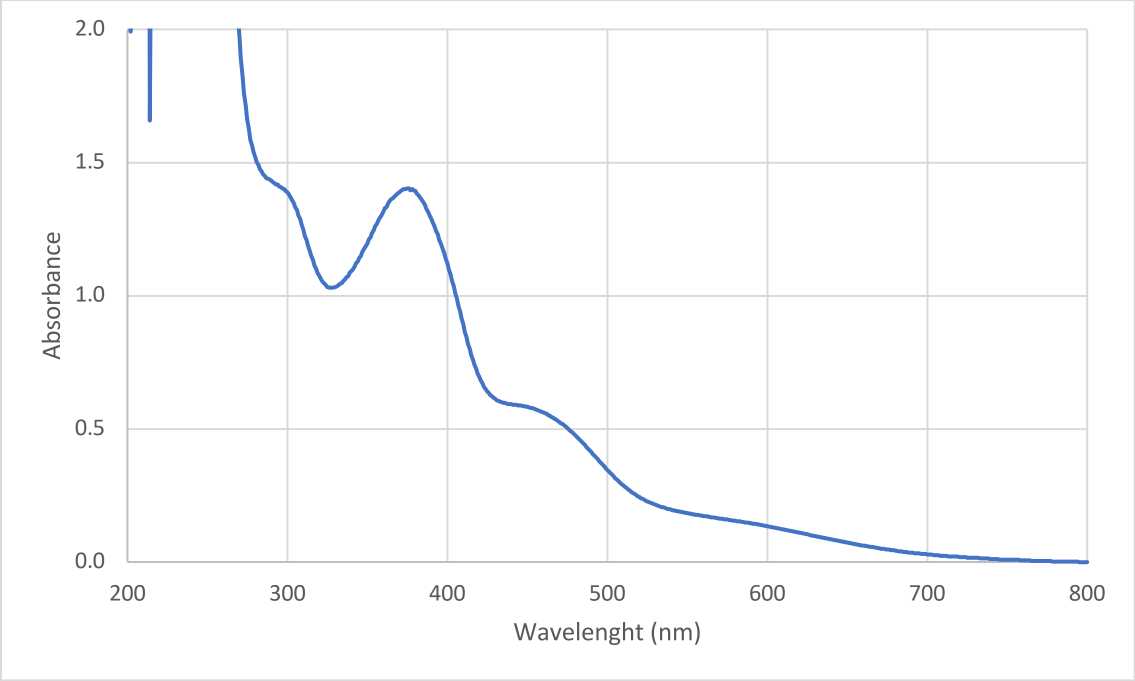

Fig.S.36: UV/vis spectrum of [RuCl(*p*-cymene)(**N-N**^{6})]PF_6 in CH_2Cl_2 (2.14×10^{-4} mol L^{-1}) at 25°C.

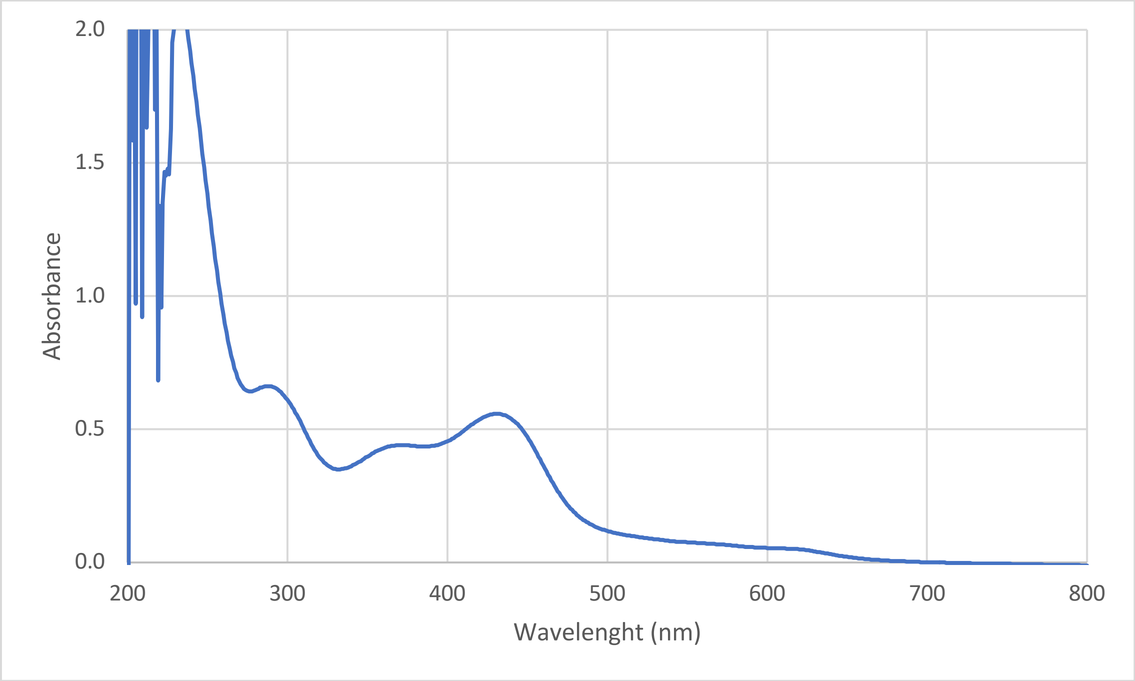

Fig.S.37: UV/vis spectrum of [RuCl(*p*-cymene)(**N-N**^{7})]PF_6 in CH_2Cl_2 (1.38×10^{-4} mol L^{-1}) at 25°C.

FTIR data of the complexes.

Table S.7: FTIR/ATR of complexes **1** – **7** and attempt to assign bands.

Complex	Wavenumber (cm^{-1})	Vibrational modes
**1**	3023, 2967, 1575, 1472, 833, 465, 274	ν_{Csp2-H}, ν_{Csp3-H}, $\nu_{C=N}$, $\nu_{C=C}$; ν_{P-F}, ν_{Ru-N}, ν_{Ru-Cl}
**2**	3049-3027, 2969-2959, 1608, 1496, 834, 449, 281	ν_{Csp2-H}, ν_{Csp3-H}, $\nu_{C=N}$, $\nu_{C=C}$, ν_{P-F}, ν_{Ru-N}, ν_{Ru-Cl}
**3**	3043, 2964, 1606, 1473-1442, 831, 439, 275	ν_{Csp2-H}, ν_{Csp3-H}, $\nu_{C=N}$, $\nu_{C=C}$, ν_{P-F}, ν_{Ru-N}, ν_{Ru-Cl}
**4**	3063, 2947, 1583, 1440, 838, 479, 306	ν_{Csp2-H}, ν_{Csp3-H}, $\nu_{C=N}$, $\nu_{C=C}$, ν_{P-F}, ν_{Ru-N}, ν_{Ru-Cl}
**5**	3145-3050, 2970, 1600, 1509,1405, 829, 557, 301	ν_{Csp2-H}, ν_{Csp3-H}, $\nu_{C=N}$, ν_{C-F}, $\nu_{C=C}$, ν_{P-F}, ν_{Ru-N}, ν_{Ru-Cl}
**6**	3104-3055, 2962, 1596, 1405, 830, 651, 557, 290	ν_{Csp2-H}, ν_{Csp3-H}, $\nu_{C=N}$, $\nu_{C=C}$, ν_{P-F}, ν_{C-Cl}, ν_{Ru-N}, ν_{Ru-Cl}
**7**	2923 - 2853,1616, 1449, 829	ν_{Csp3-H}, $\nu_{C=N}$, $\nu_{C=C}$, ν_{P-F}

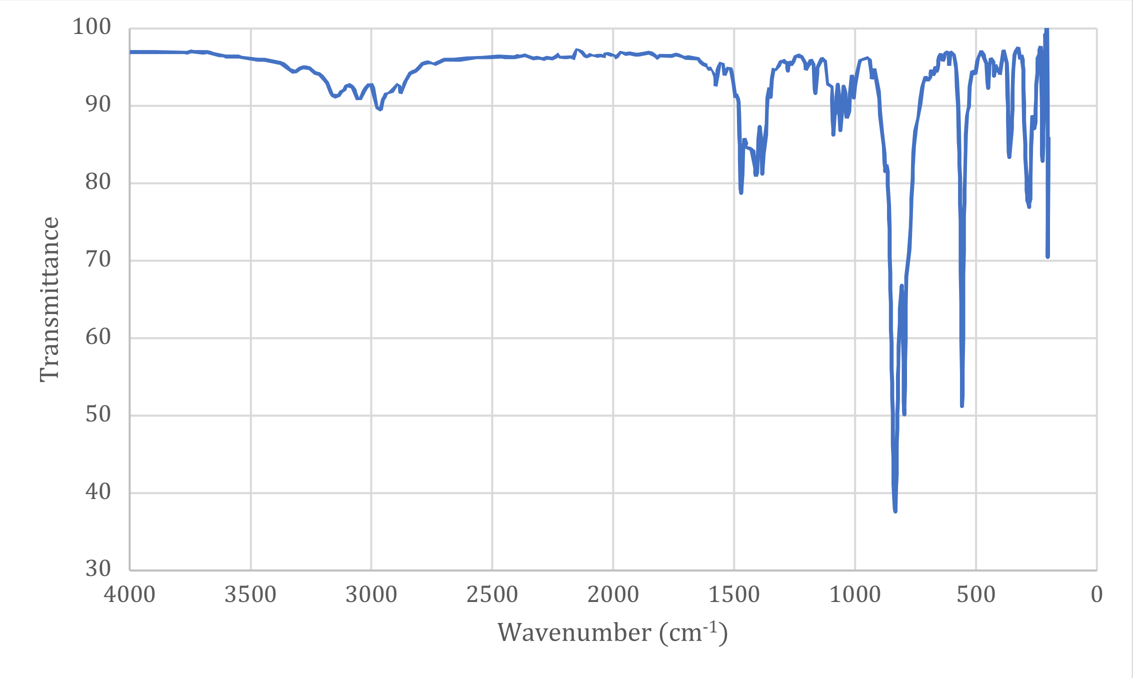

Fig.S.38: FTIR/ATR spectrum of [RuCl(*p*-cymene)(**N-N**[1])]PF_6.

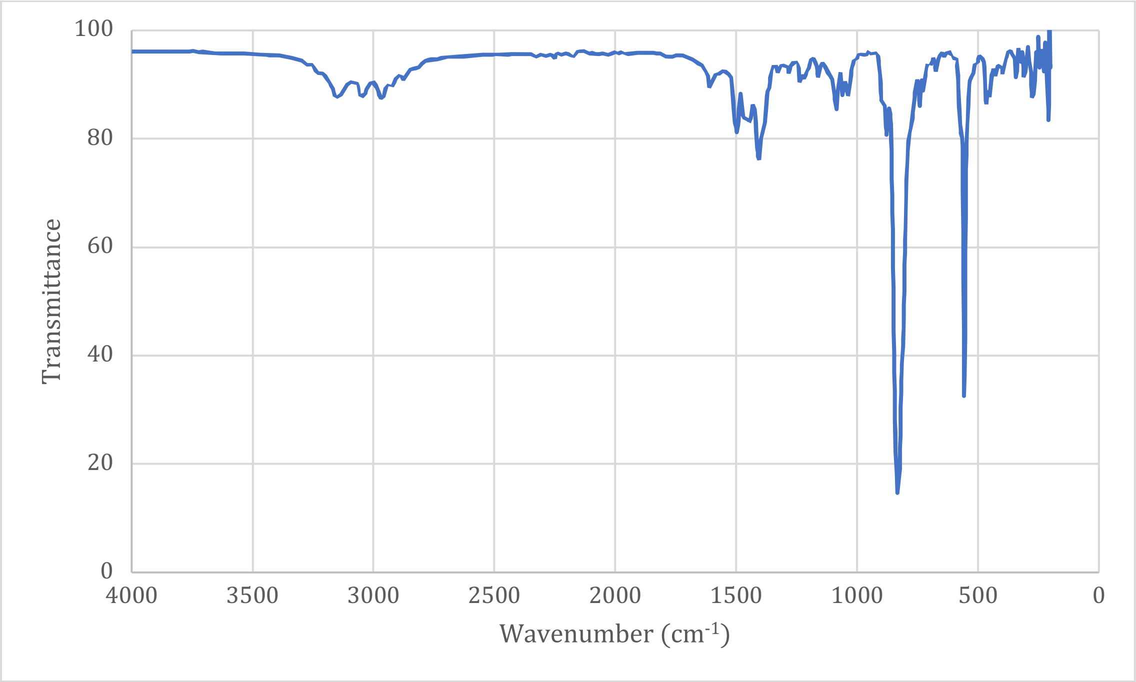

Fig.S.39: FTIR/ATR spectrum of [RuCl(*p*-cymene)(**N-N**[2])]PF_6.

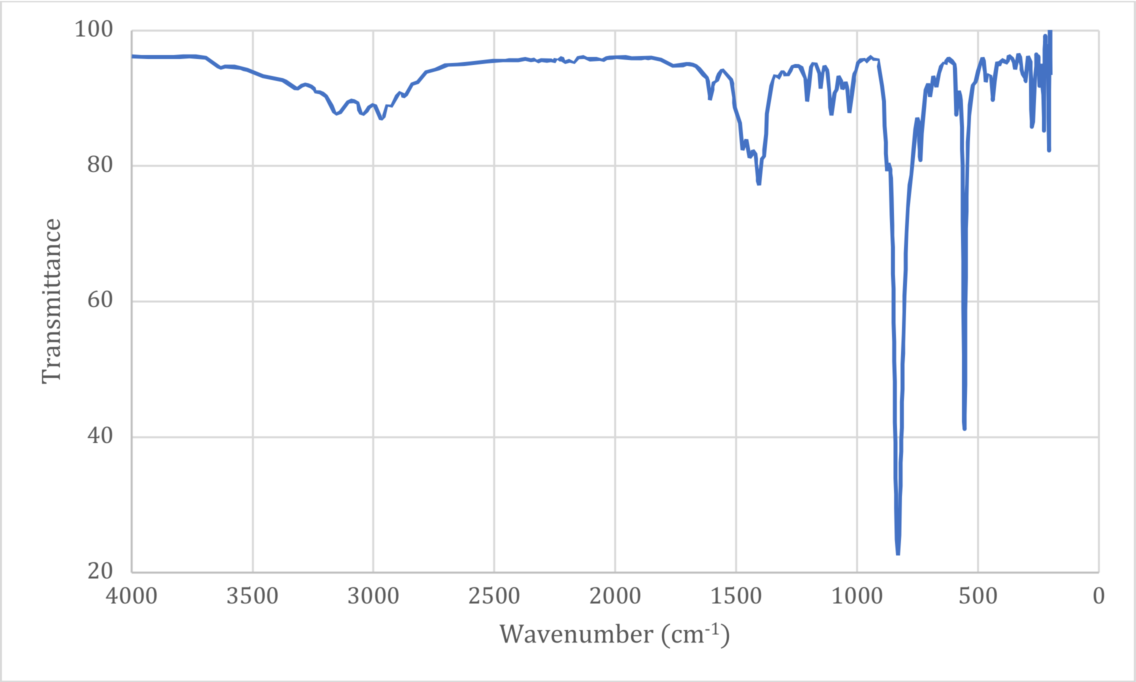

Fig.S.40: FTIR/ATR spectrum of [RuCl(*p*-cymene)(**N-N**[3])]PF_6.

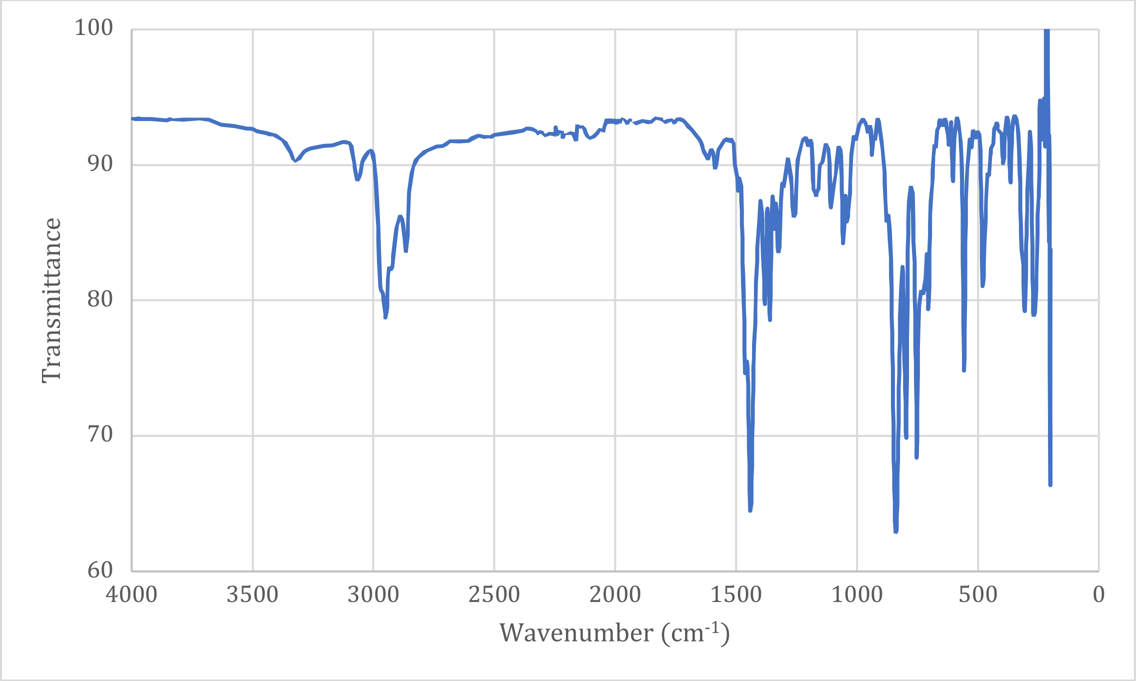

Fig.S.41: FTIR/ATR spectrum of [RuCl(*p*-cymene)(**N-N**[4])]PF_6.

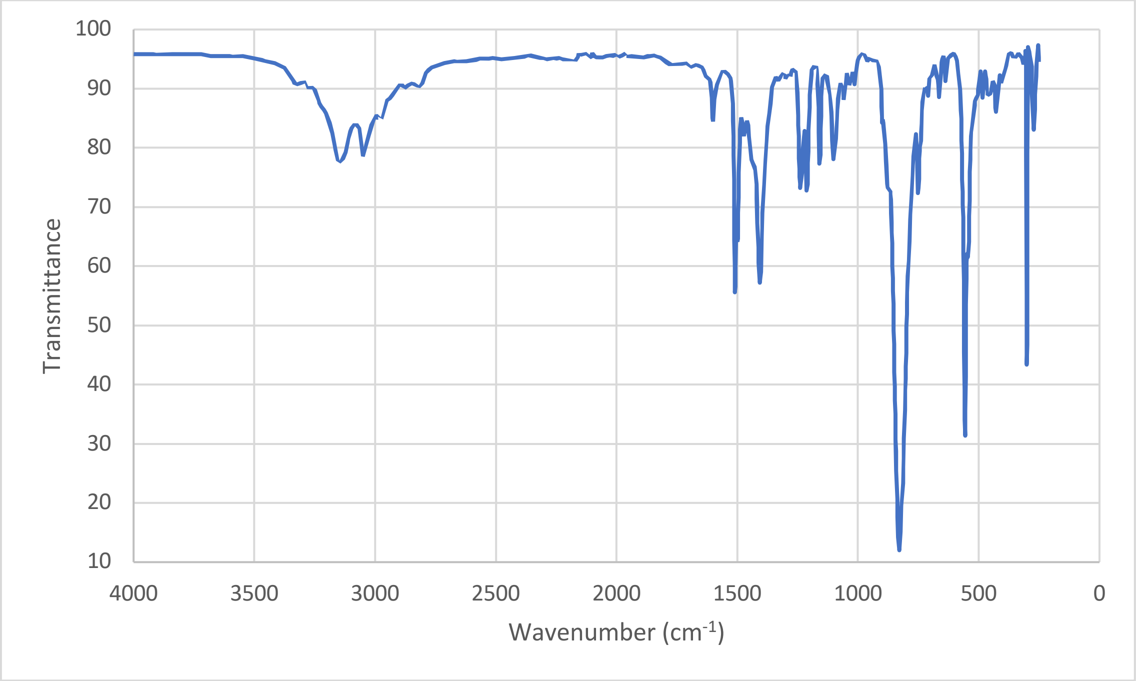

Fig.S.42: FTIR/ATR spectrum of [RuCl(*p*-cymene)(**N-N**[5])]PF_6.

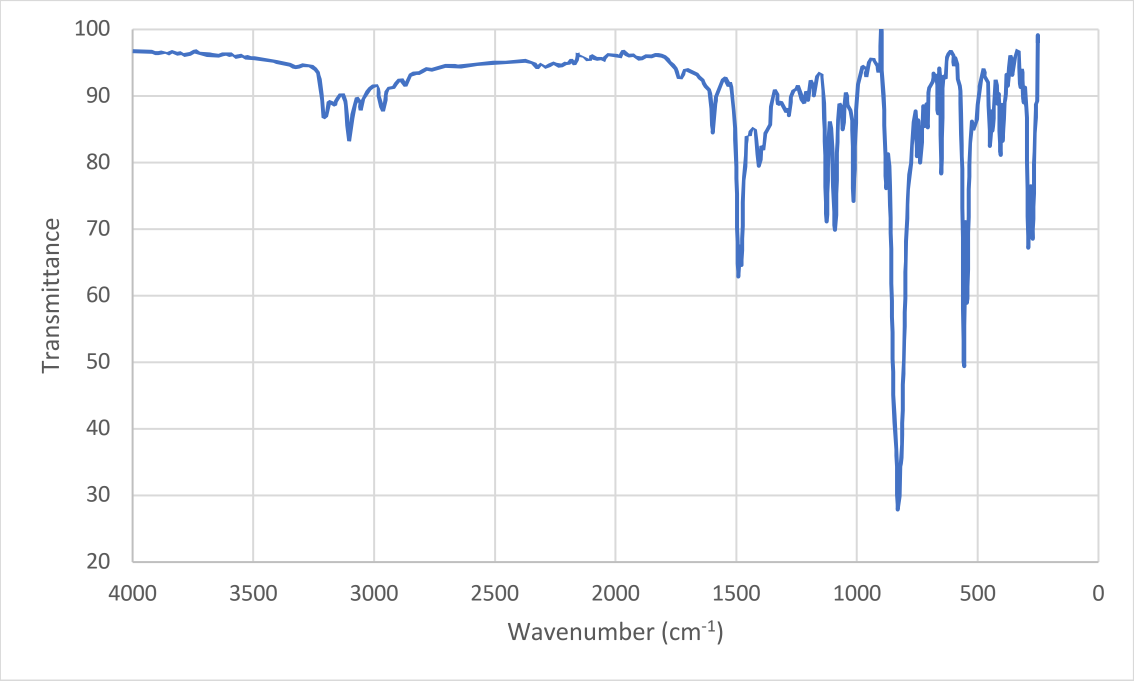

Fig.S.43: FTIR/ATR spectrum of [RuCl(*p*-cymene)(**N-N*6***)]PF_6.

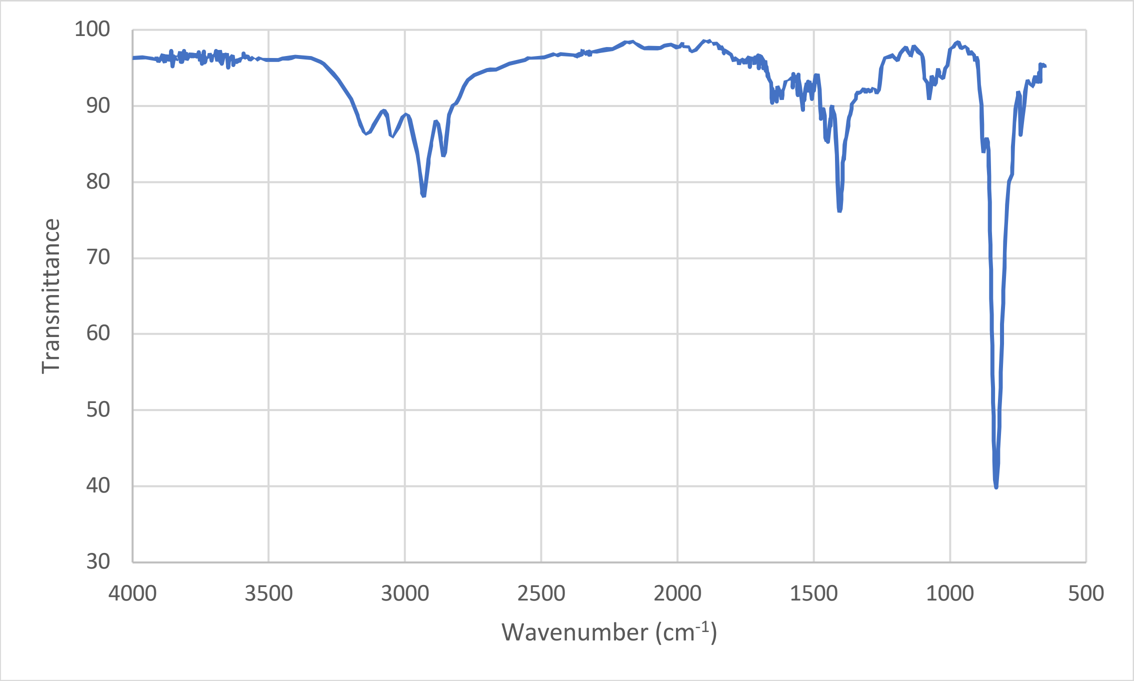

Fig.S.44: FTIR/ATR spectrum of [RuCl(*p*-cymene)(**N-N*7***)]PF_6.

NMR spectroscopy of complexes.

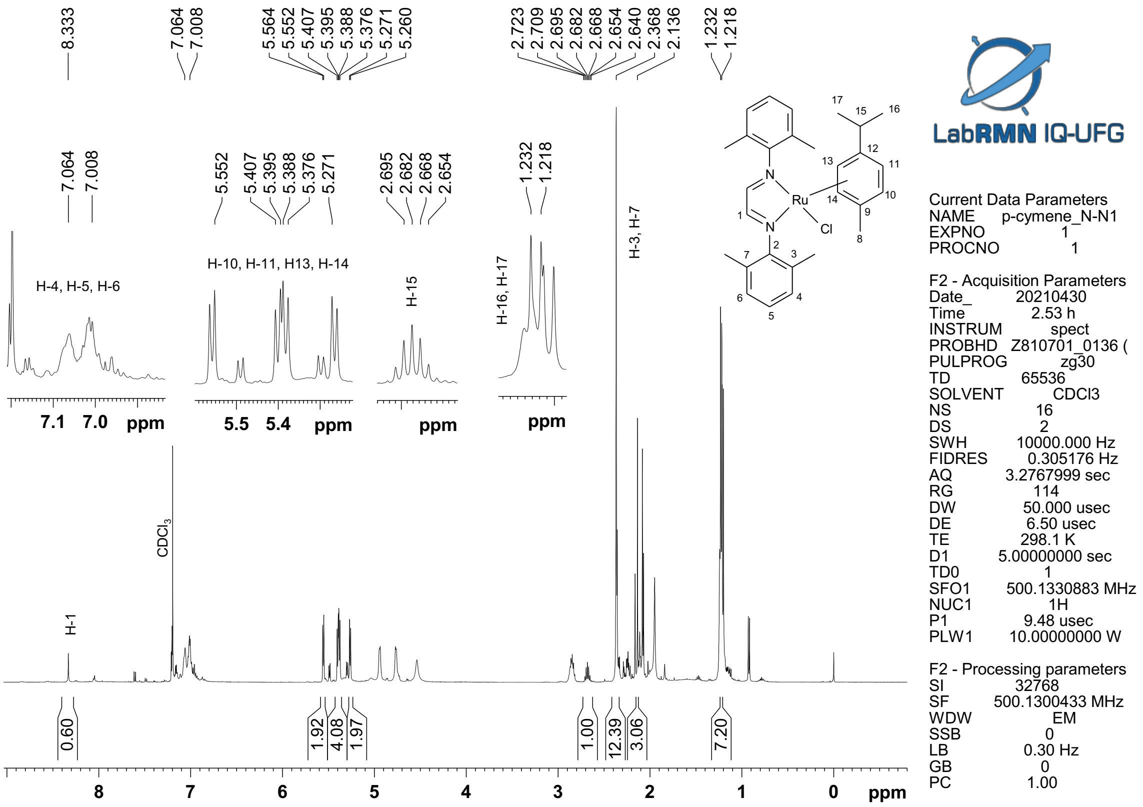

Fig.S.45: ^{1}H NMR spectrum (500.13 MHz, $CDCl_3$) of [RuCl(*p*-cymene)(**N-N^{1}**)]PF_6.

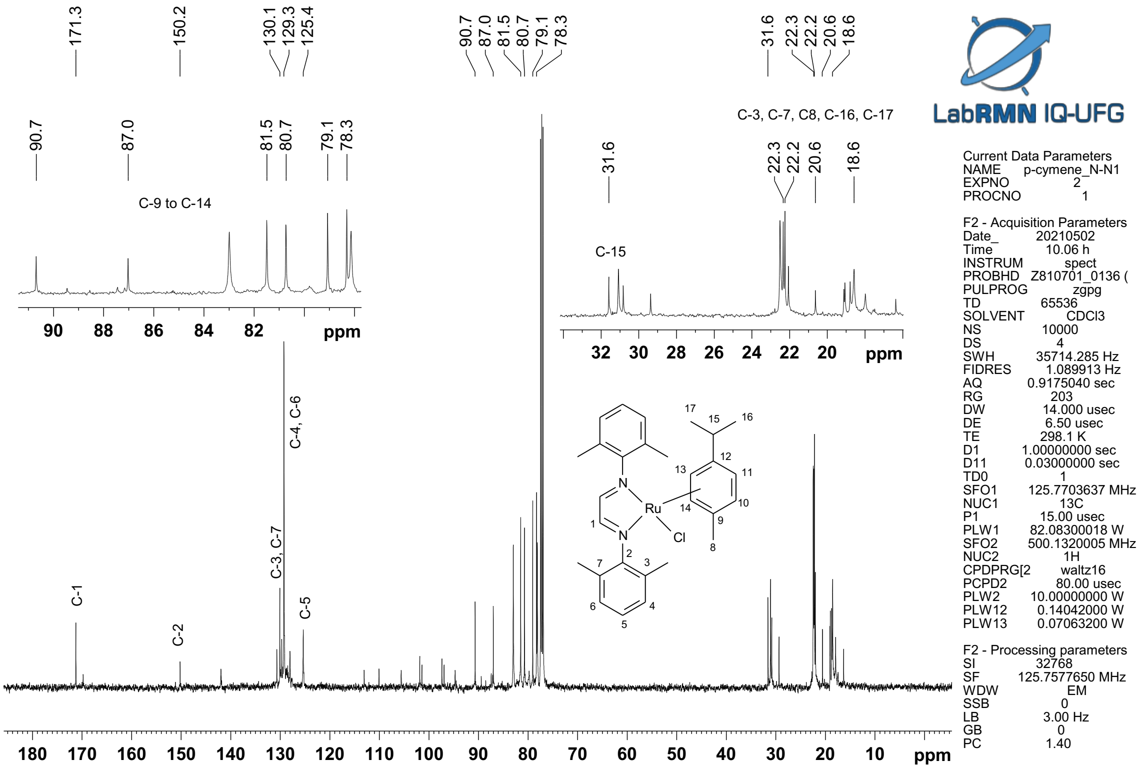

Fig.S.46: ^{13}C NMR spectrum (125.75 MHz, $CDCl_3$) of [RuCl(*p*-cymene)(**N-N^{1}**)]PF_6.

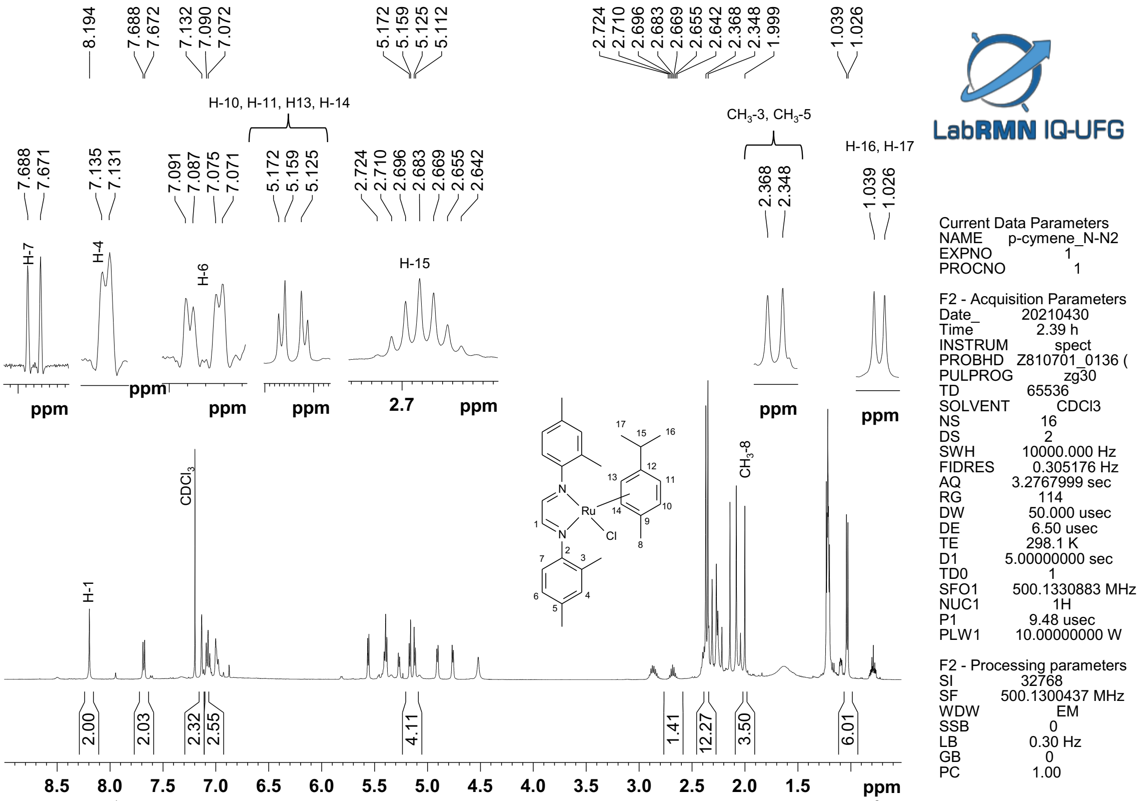

Fig.S.47: ^{1}H NMR spectrum (500.13 MHz, $CDCl_3$) of [RuCl(*p*-cymene)(**N-N^2**)]PF_6. In the expanded signals, window functions (Lorentzian and Gaussian) were used to improve the visualization of multiplicities.

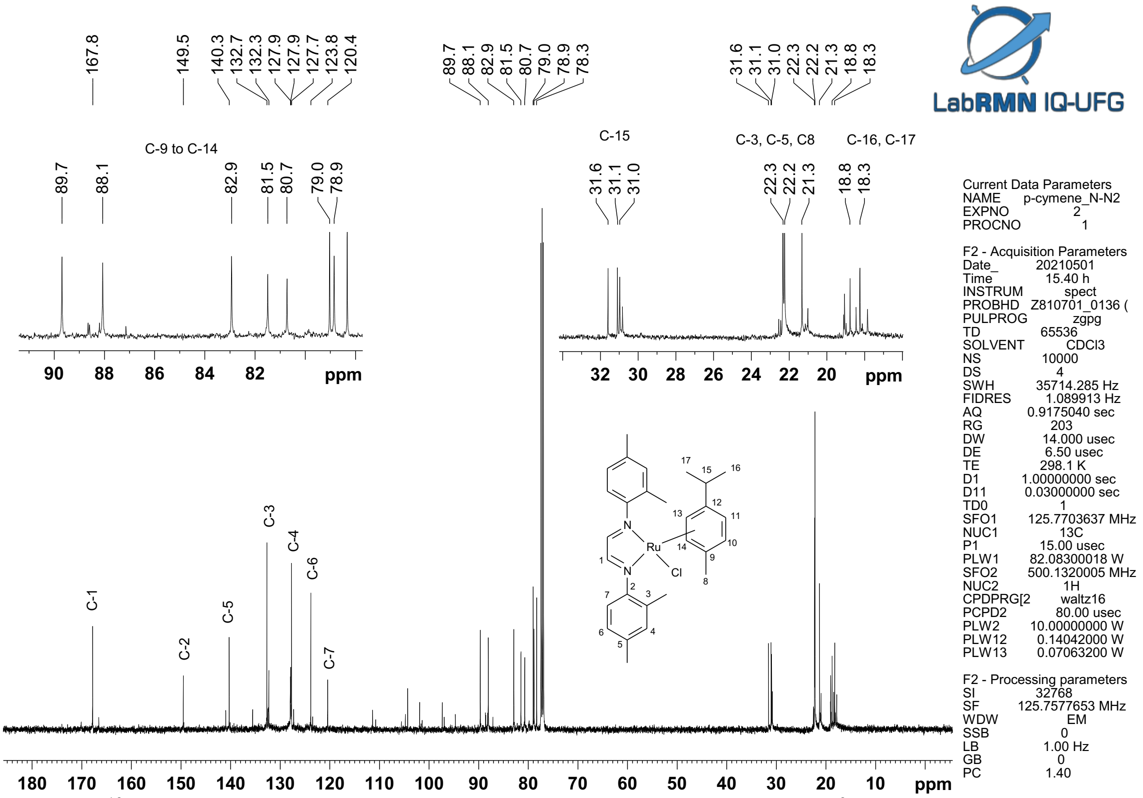

Fig.S.48: ^{13}C NMR spectrum (125.75 MHz, $CDCl_3$) of [RuCl(*p*-cymene)(**N-N^2**)]PF_6.

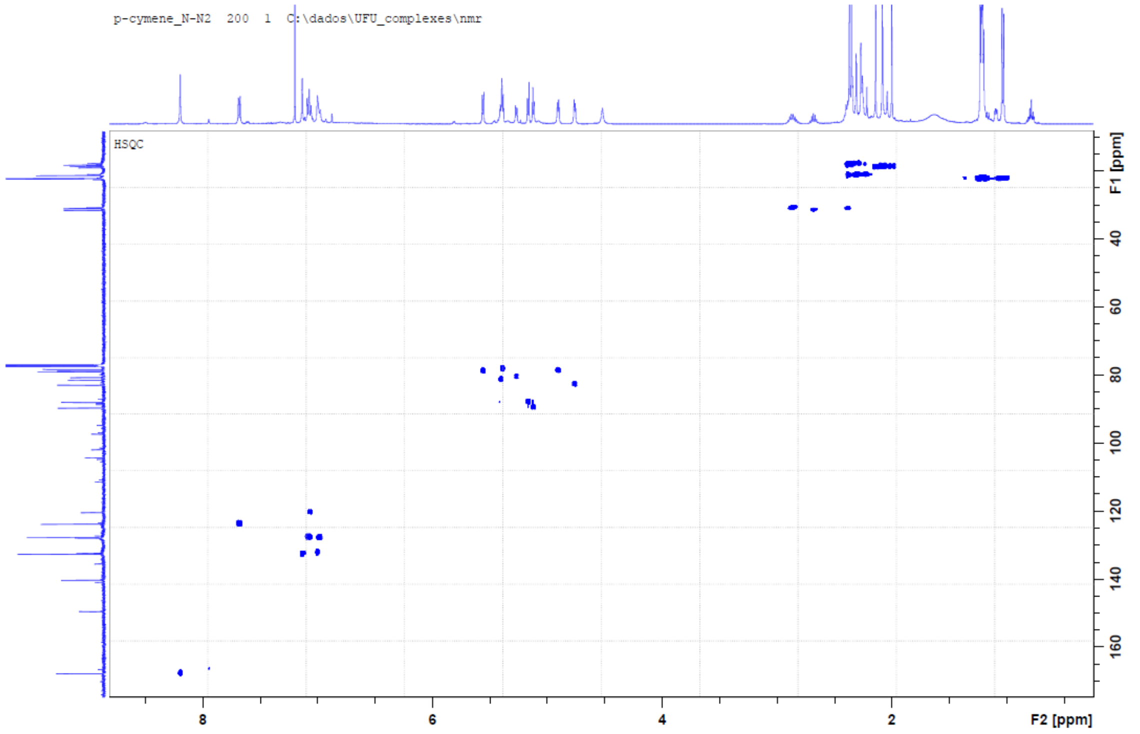

Fig. S 49: ^{1}H-^{13}C HSQC NMR contour map for [RuCl(*p*-cymene)(**N-N^{2}**)]PF_6 (500 and 125 MHz, $CDCl_3$).

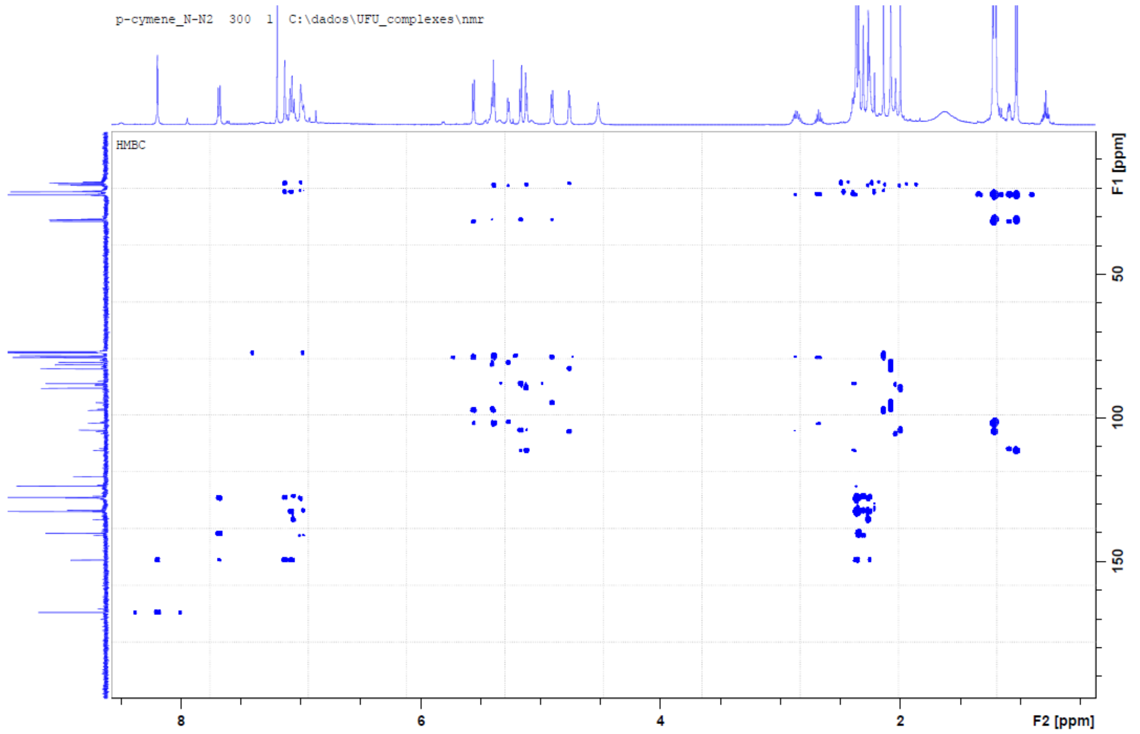

Fig. S 50: ^{1}H-^{13}C HMBC NMR contour map for [RuCl(p-cymene)(**N-N^{2}**)]PF_6 (500 and 125 MHz, $CDCl_3$).

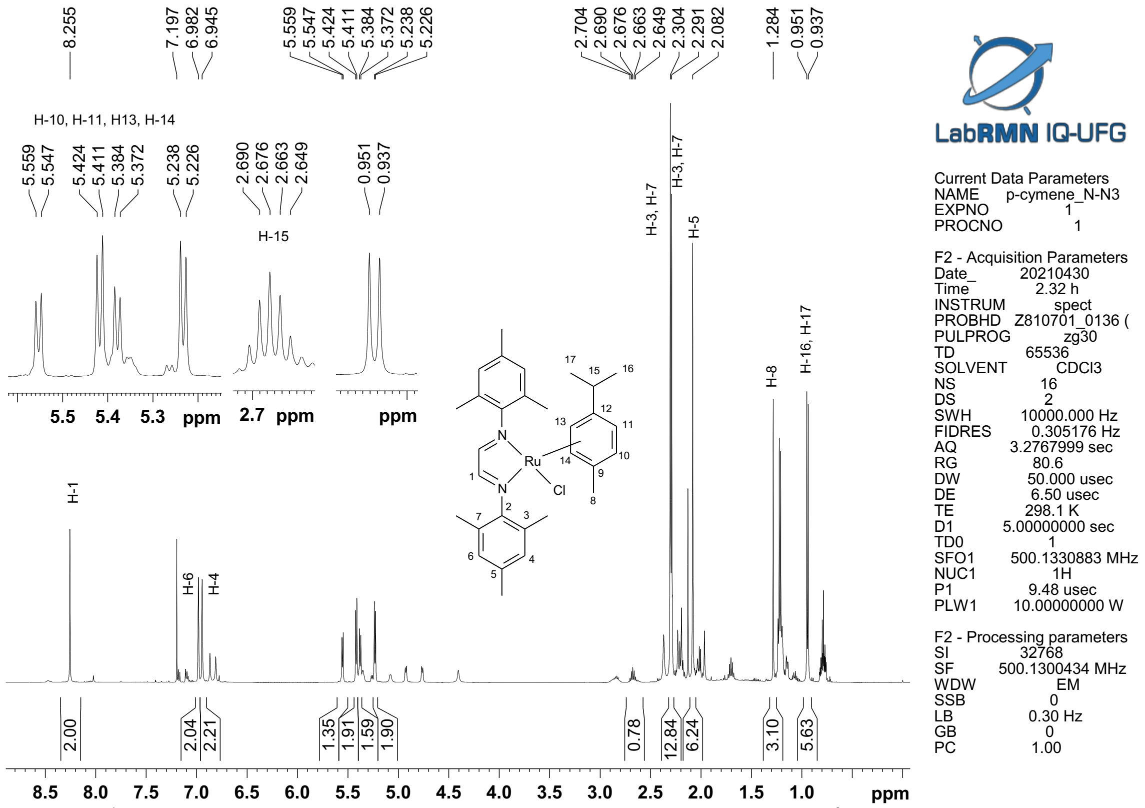

Fig.S.51: ^{1}H NMR spectrum (500.13 MHz, $CDCl_3$) of [RuCl(*p*-cymene)(**N-N**^{3})]PF_6.

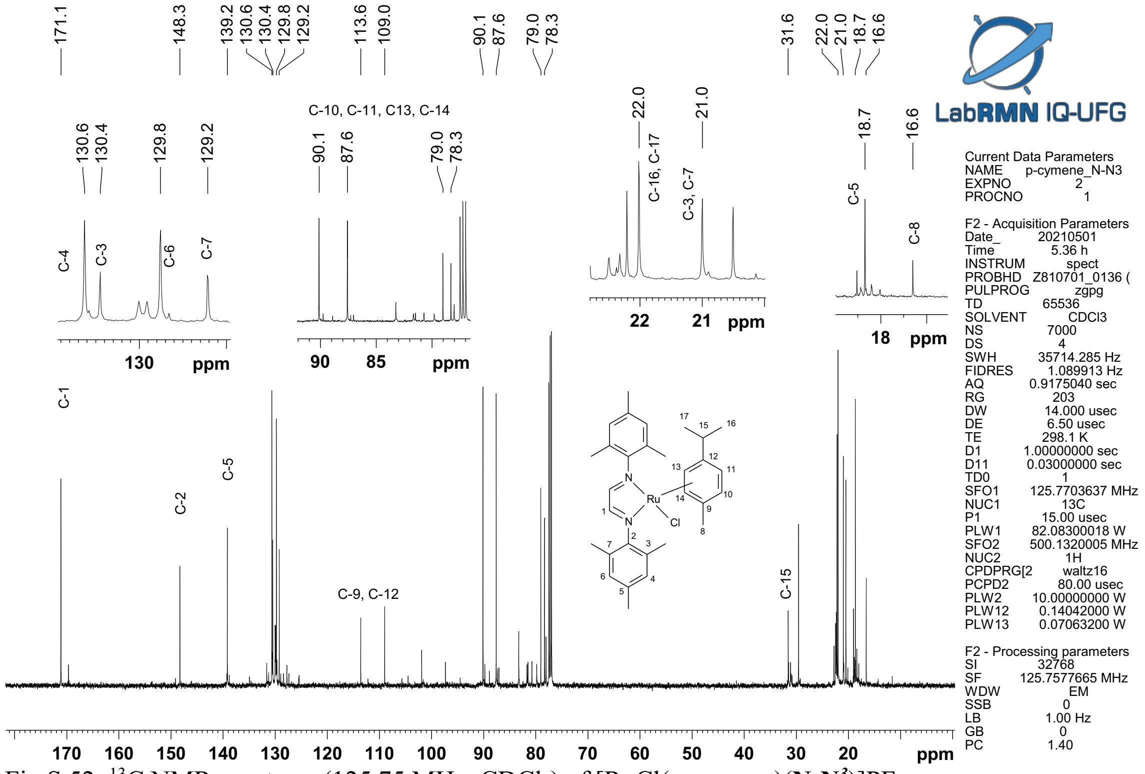

Fig.S.52: ^{13}C NMR spectrum (125.75 MHz, $CDCl_3$) of [RuCl(*p*-cymene)(**N-N**^{3})]PF_6.

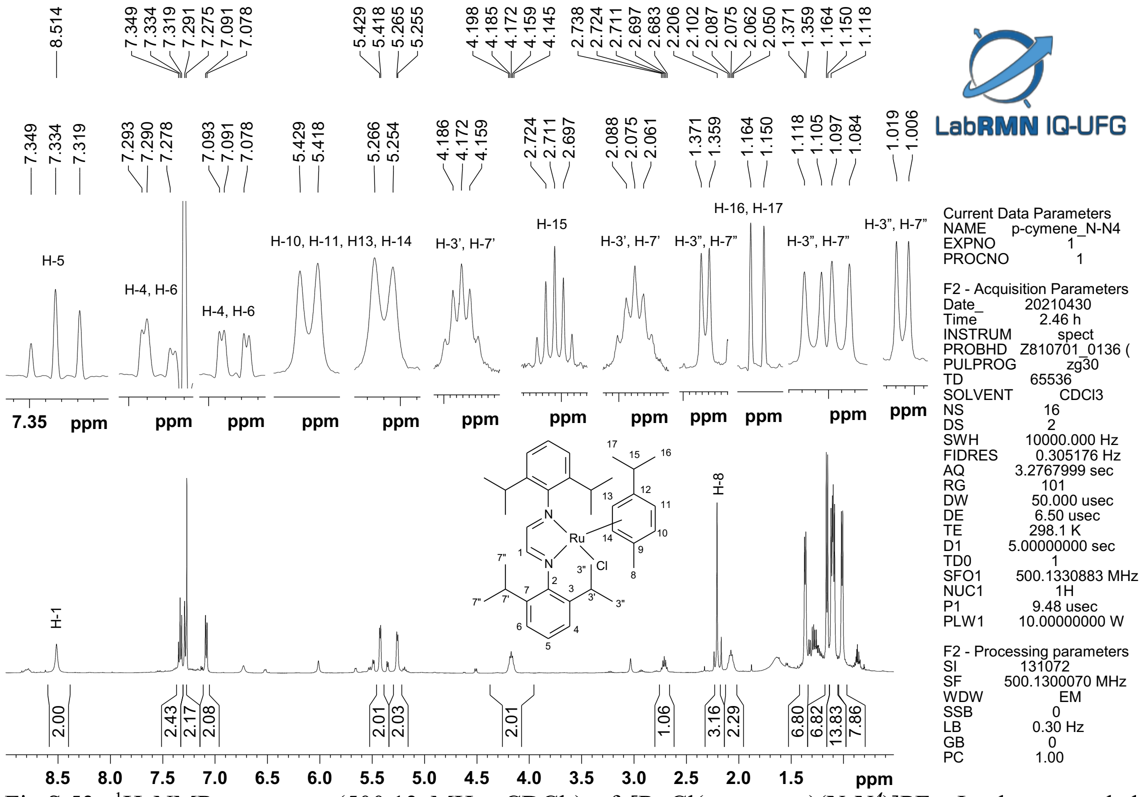

Fig.S.53: ^{1}H NMR spectrum (500.13 MHz, $CDCl_3$) of [RuCl(*p*-cymene)(**N-N**[4])]PF_6. In the expanded signals, window functions (Lorentzian and Gaussian) were used to improve the visualization of multiplicities.

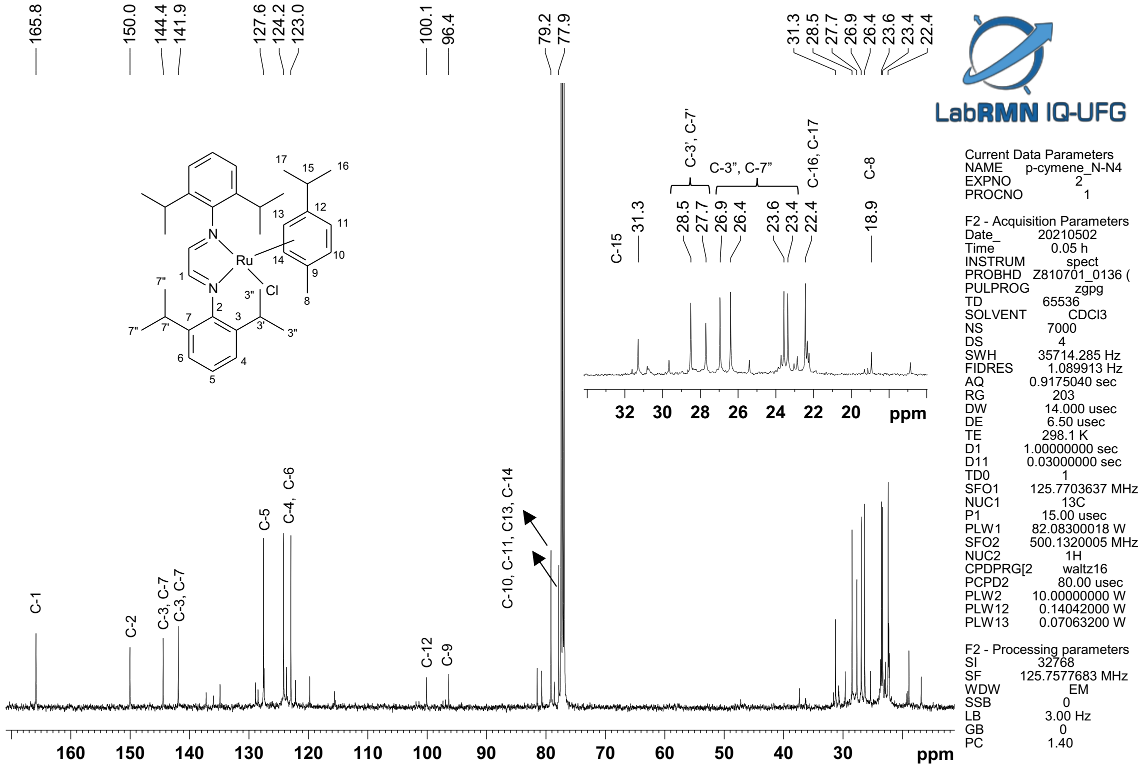

Fig.S.54: ^{13}C NMR spectrum (125.75 MHz, $CDCl_3$) of [RuCl(*p*-cymene)(**N-N**[4])]PF_6.

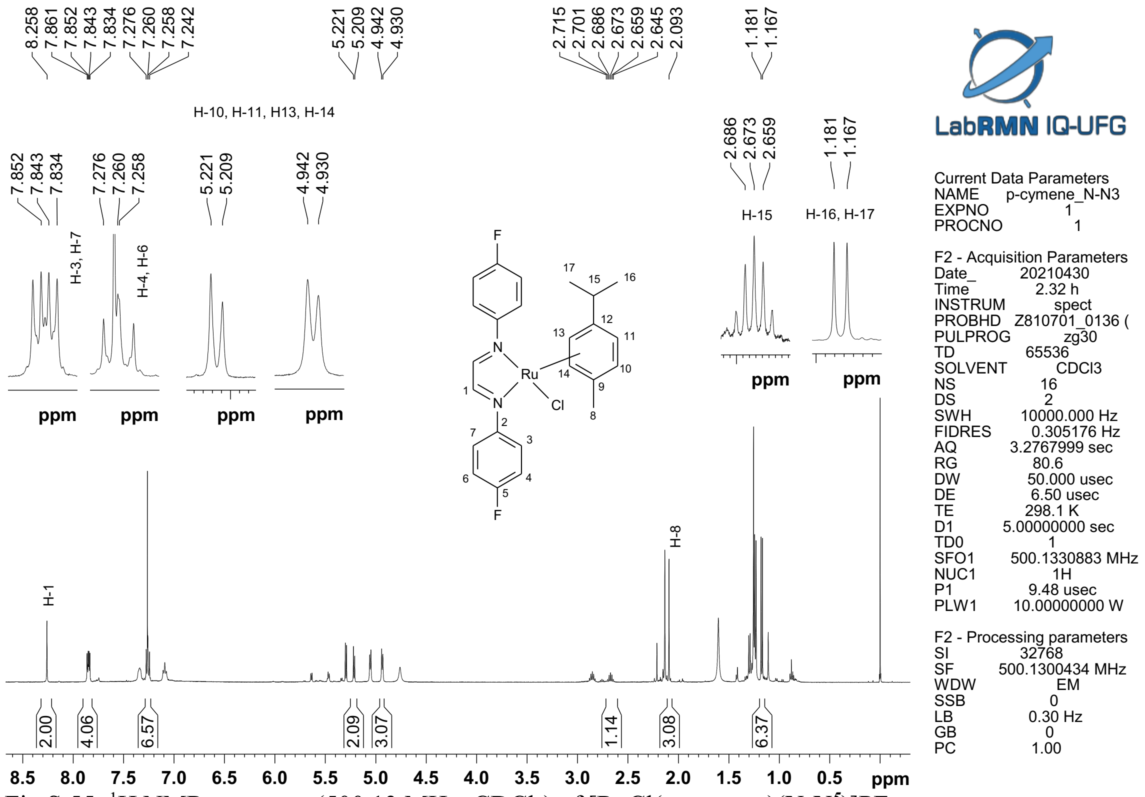

Fig.S.55: ^{1}H NMR spectrum (500.13 MHz, $CDCl_3$) of [RuCl(*p*-cymene)(**N-N^5**)]PF_6.

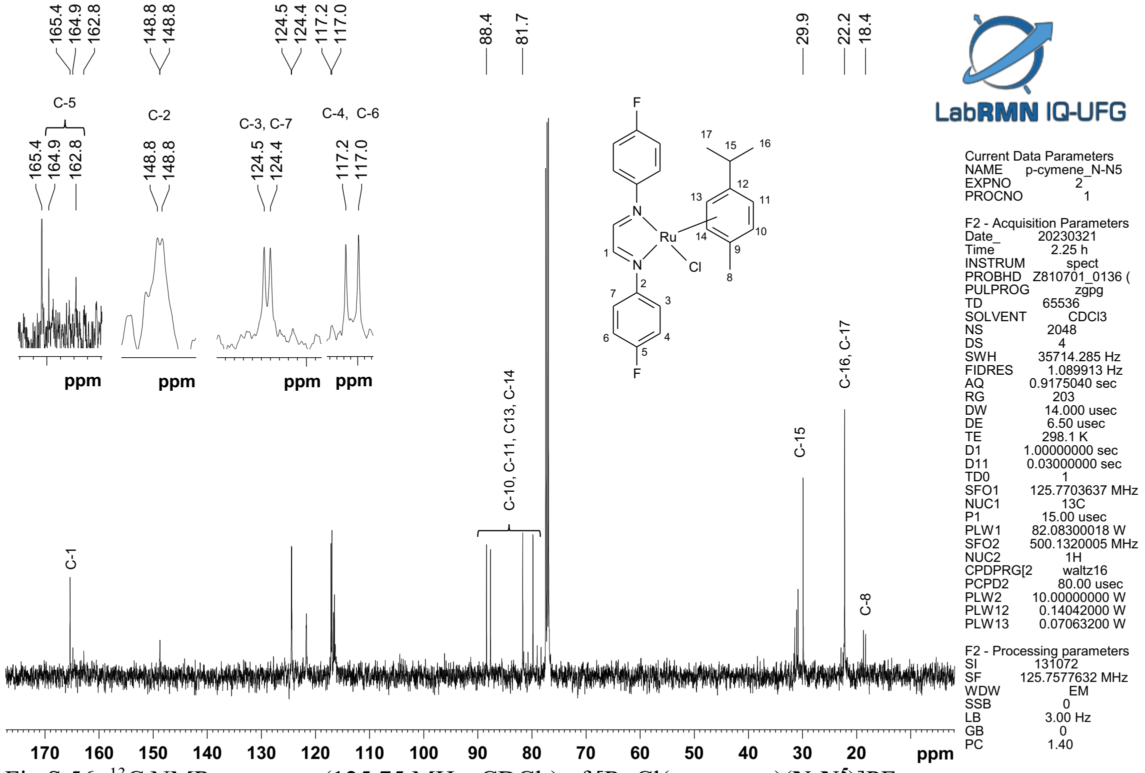

Fig.S.56: ^{13}C NMR spectrum (125.75 MHz, $CDCl_3$) of [RuCl(*p*-cymene)(**N-N^5**)]PF_6.

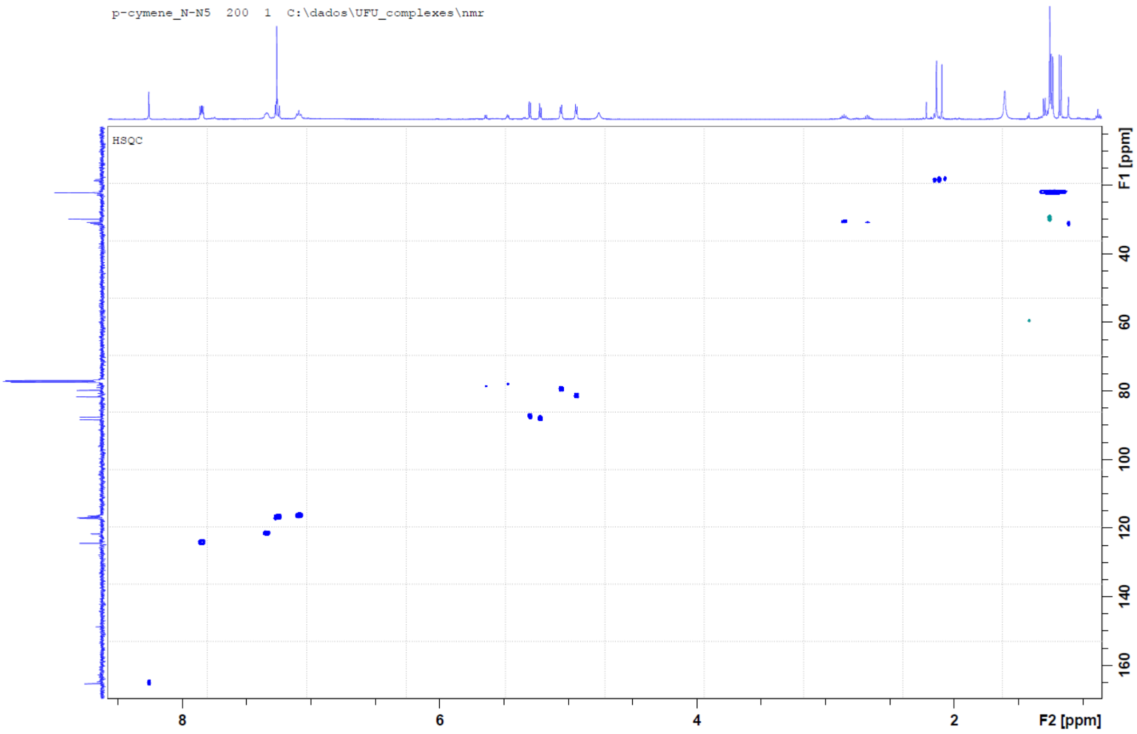

Fig. S 57: ^{1}H-^{13}C HSQC NMR contour map for [RuCl(*p*-cymene)(**N-N^5**)]PF_6 (500 and 125 MHz, $CDCl_3$).

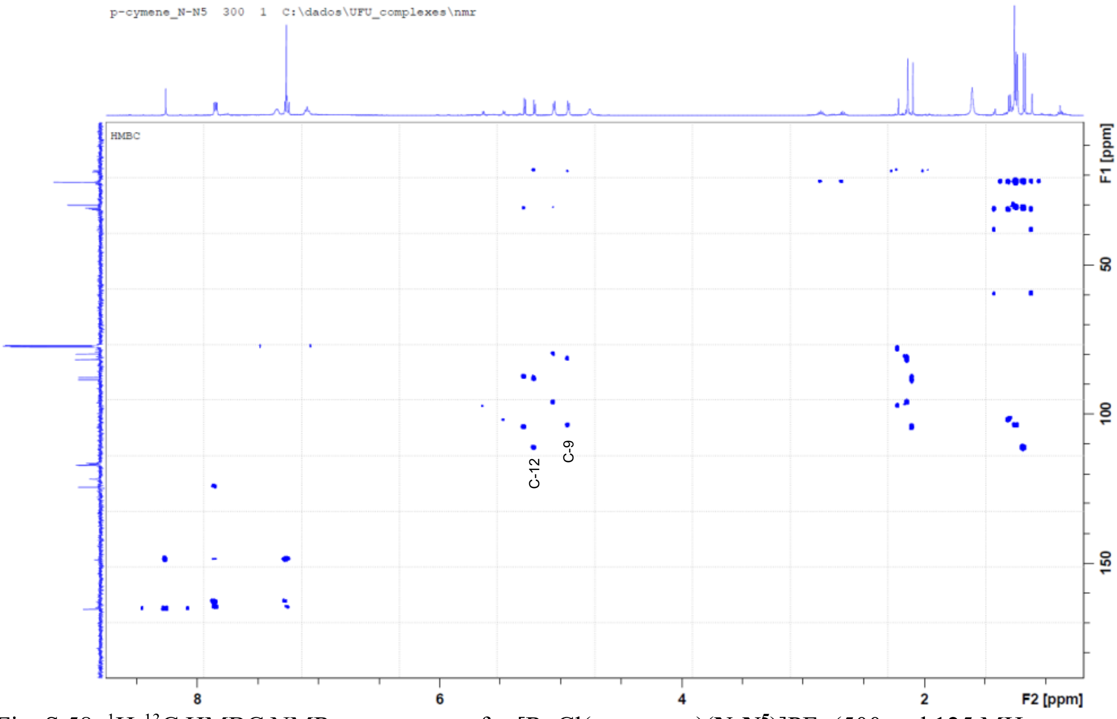

Fig. S 58: ^{1}H-^{13}C HMBC NMR contour map for [RuCl(p-cymene)(**N-N^5**)]PF_6 (500 and 125 MHz, $CDCl_3$). The C-9 and C-12 carbons were not observed in the ^{13}C NMR spectrum and are highlighted.

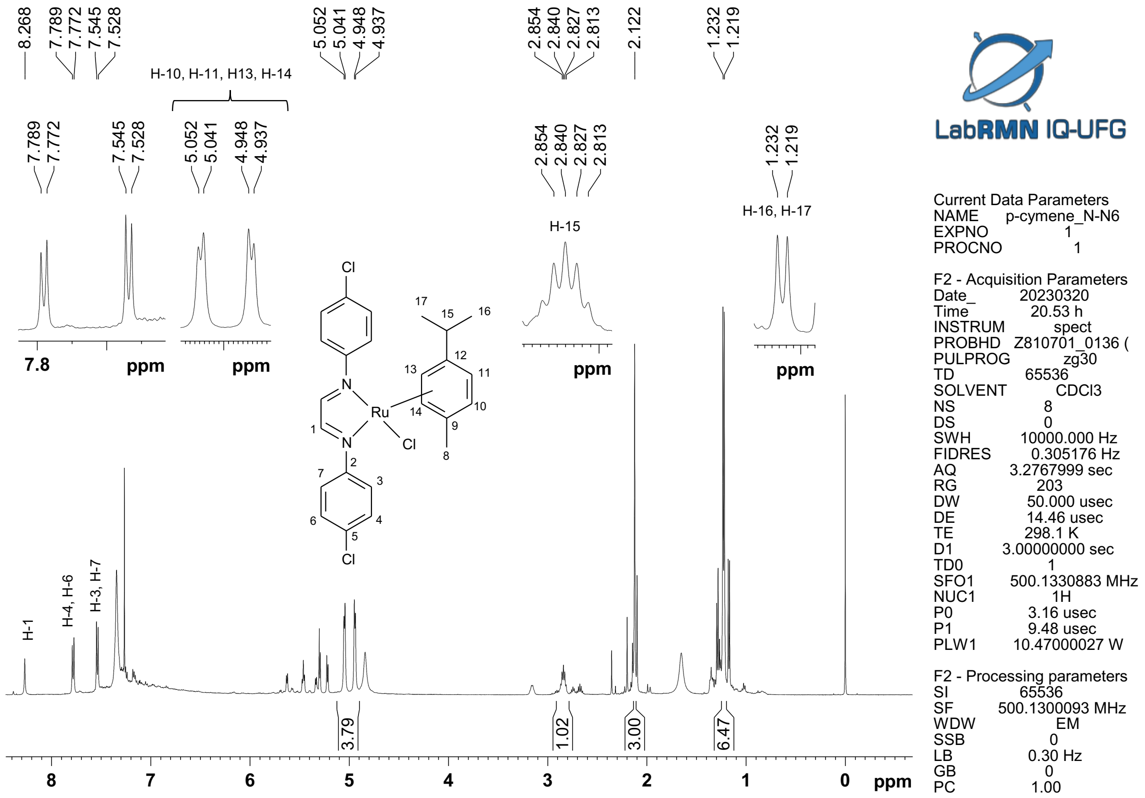

Fig.S.59: ^{1}H NMR spectrum (500.13 MHz, $CDCl_3$) of [RuCl(*p*-cymene)(**N-N^6**)]PF_6.

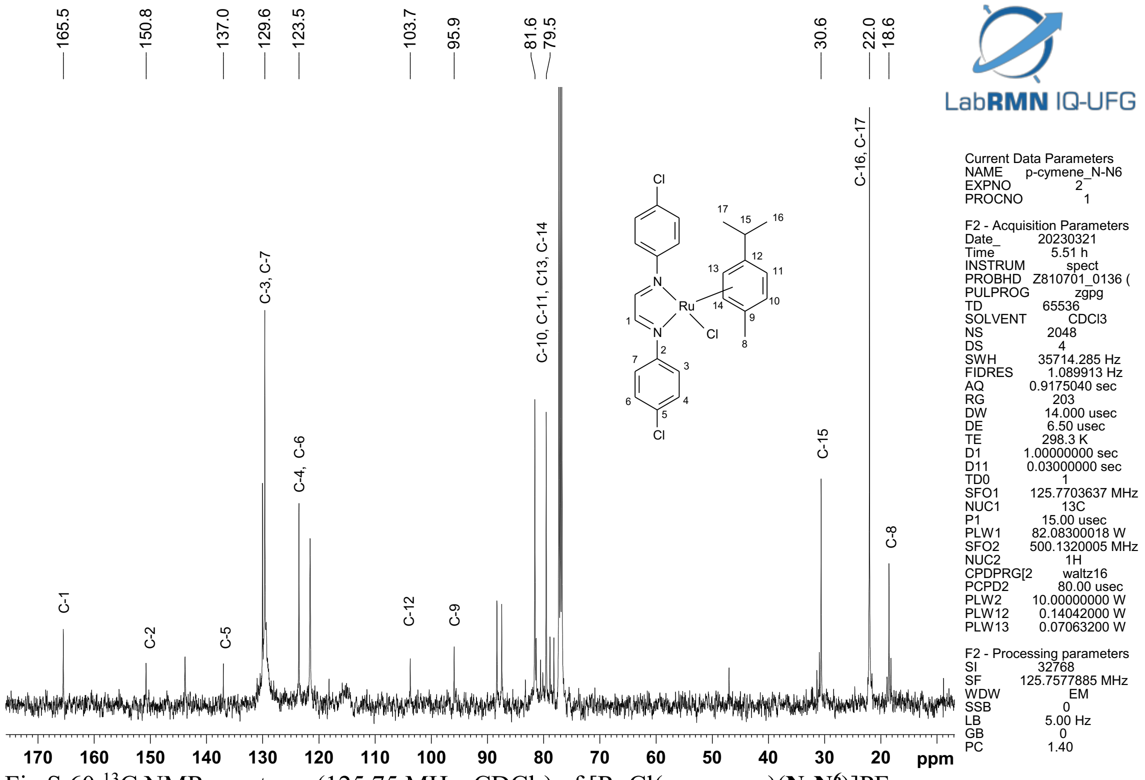

Fig.S.60: ^{13}C NMR spectrum (125.75 MHz, $CDCl_3$) of [RuCl(p-cymene)(**N-N^6**)]PF_6.

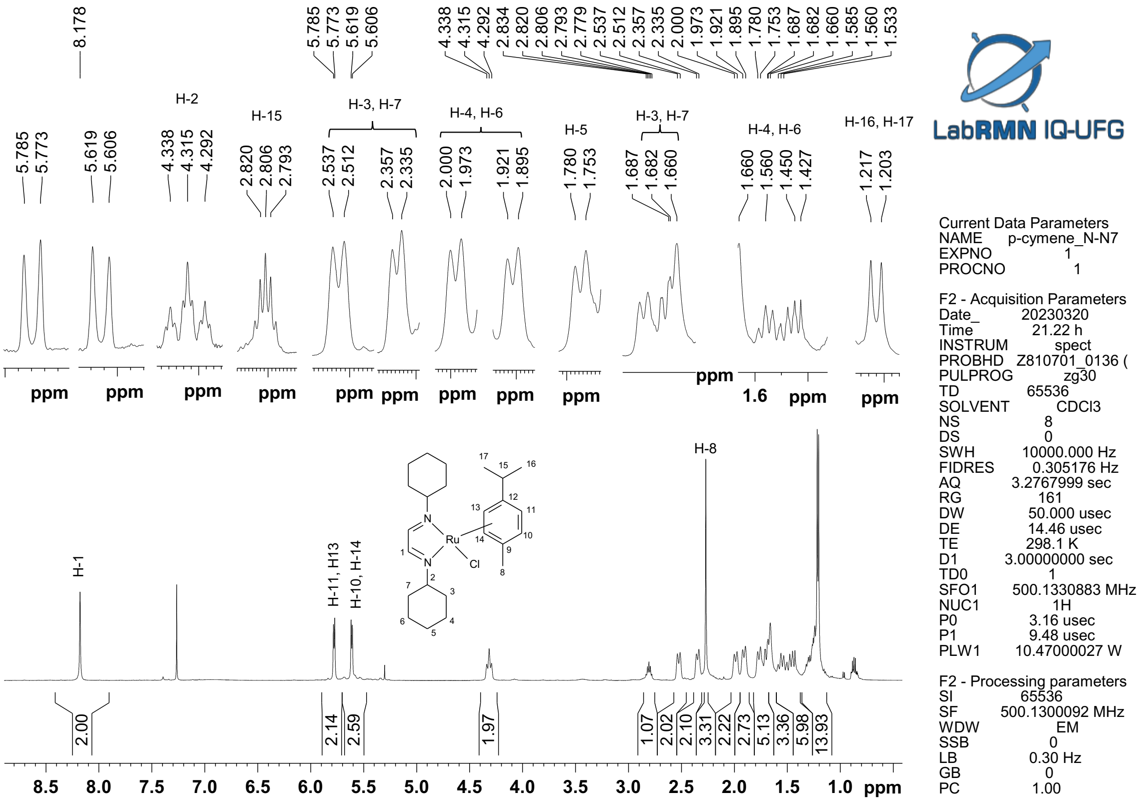

Fig.S.61: ^{1}H NMR spectrum (500.13 MHz, $CDCl_3$) of [RuCl(*p*-cymene)(**N-N**[7])]PF_6. In the expanded signals, window functions (Lorentzian and Gaussian) were used to improve the visualization of multiplicities.

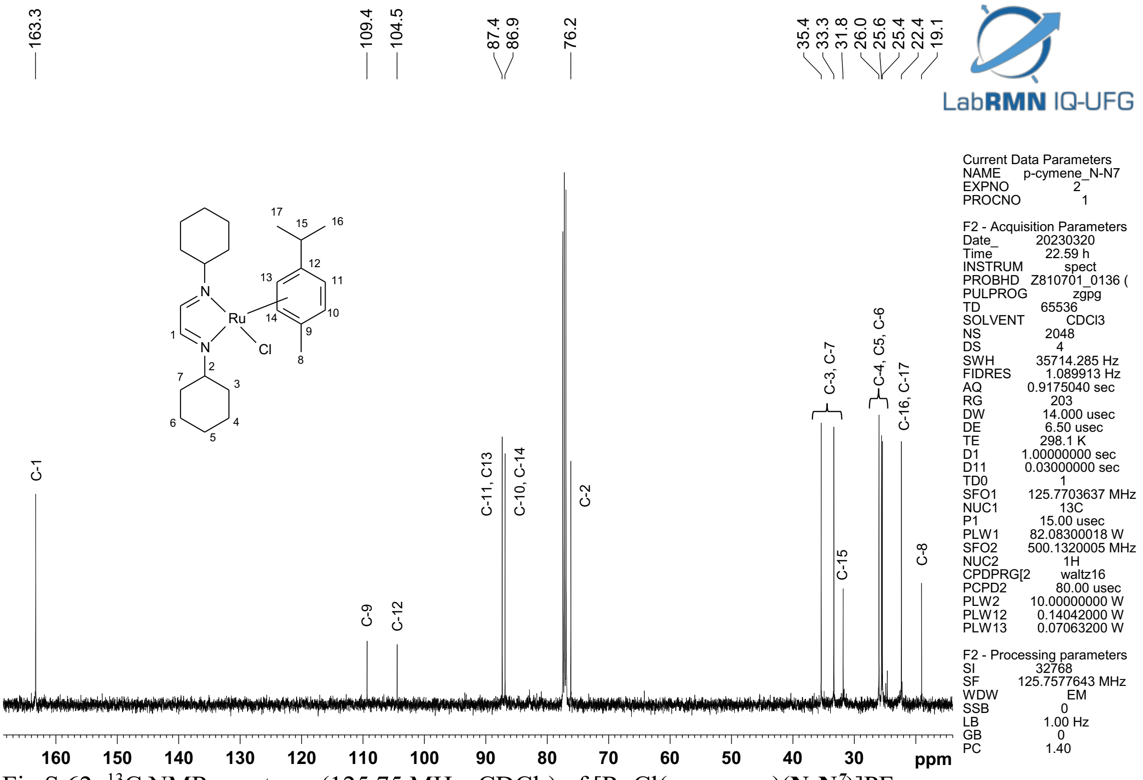

Fig.S.62: ^{13}C NMR spectrum (125.75 MHz, $CDCl_3$) of [RuCl(*p*-cymene)(**N-N**[7])]PF_6.

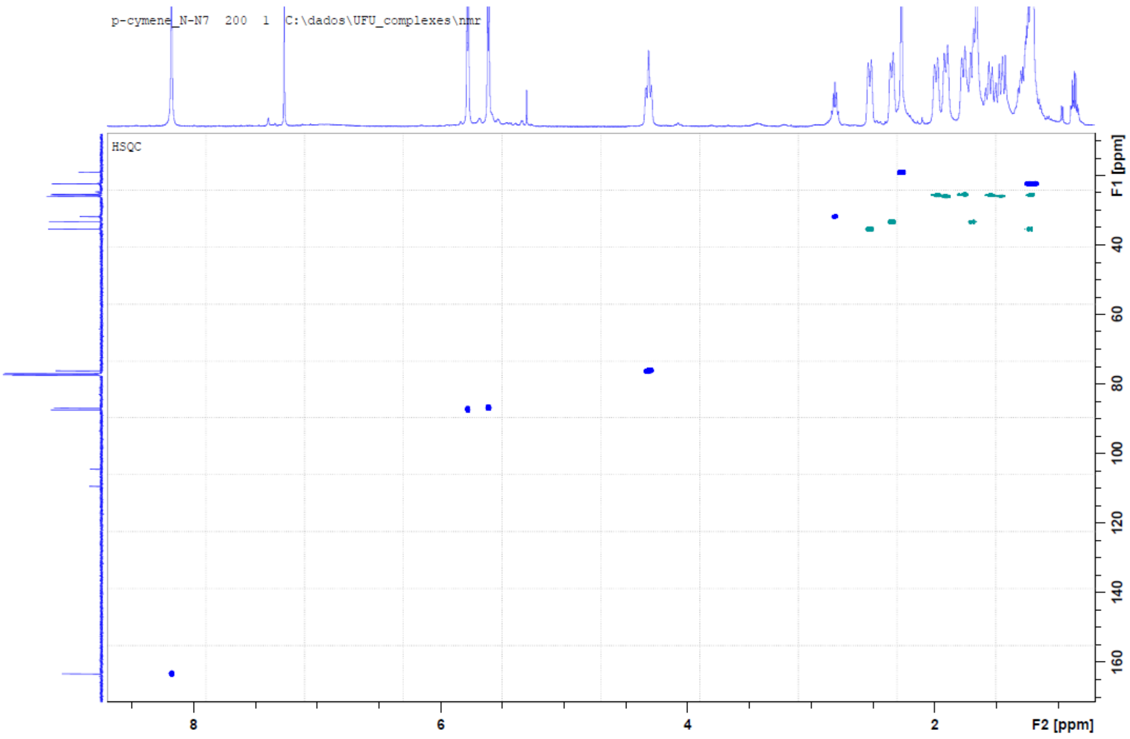

Fig. S 63: ^{1}H-^{13}C HSQC NMR contour map for [RuCl(p-cymene)(**N-N**^{7})]PF_6 (500 and 125 MHz, $CDCl_3$). The C-9 and C-12 carbons were not observed in the ^{13}C NMR spectrum and are highlighted.

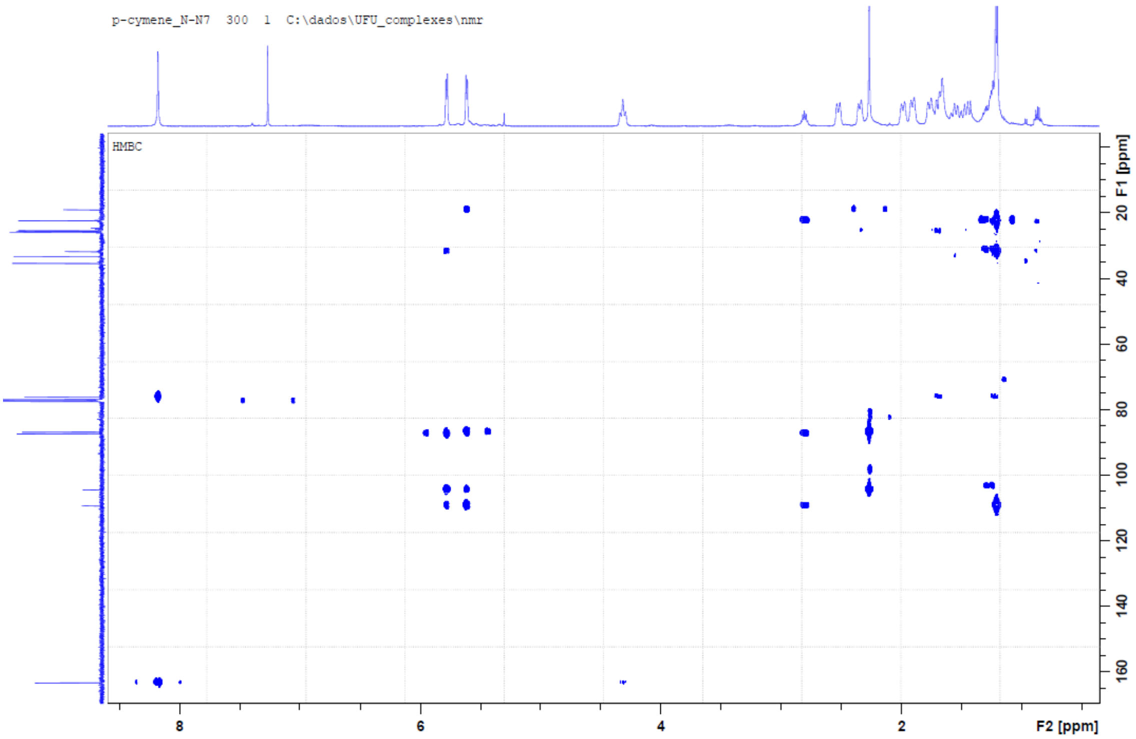

Fig. S 64: ^{1}H-^{13}C HMBC NMR contour map for [RuCl(p-cymene)(**N-N**^{7})]PF_6 (500 and 125 MHz, $CDCl_3$). The C-9 and C-12 carbons were not observed in the ^{13}C NMR spectrum and are highlighted.

X-ray data

Crystal Data and Experimental for **2**

Experimental. Single red prism-shaped crystals of **2** were obtained by recrystallisation from slow diffusion of a dichloromethane-hexamethyldisiloxane solution at low temperature (- 8 °C). A suitable crystal was selected and mounted on a suitable support on a Rigaku XtaLAB Synergy-S Dualflex, HyPix diffractometer. The crystal was kept at a steady T = 99.97(15) K during data collection. The structure was solved with the olex2.solve 1.5 (Bourhis et al., 2015) structure solution program using the Charge Flipping solution method and by using **Olex2** (Dolomanov et al., 2009) as the graphical interface. The model was refined with version 2019/2 of ShelXL 2019/2 (Sheldrick, 2015) using Least Squares minimization. The structural data were deposited at the Cambridge Structural Database, with CCDC number 2450155 for **2**.

Crystal Data. $C_{28}H_{34}ClF_6N_2PRu$, M_r = 680.06, monoclinic, $P2_1/c$ (No. 14), a = 15.08171(9) Å, b = 11.97569(8) Å, c = 15.87022(11) Å, β = 92.9253(6)°, V = 2862.65(3) Å^3, T = 99.97(15) K, Z = 4, Z' = 1, μ(Cu K$_a$)= 6.357, 38086 reflections measured, 5834 unique (R_{int} = 0.0369) which were used in all calculations. The final wR_2 was 0.0552 (all data) and R_1 was 0.0219 (I > 2σ(I)).

Data were measured using profile data from ω-scans of 0.5° per frame for 0.1/0.3 s using Cu K$_\alpha$ radiation. The diffraction pattern was indexed, and the total number of runs and images was based on the strategy calculation from the program **CrystalClear** (Rigaku) The maximum resolution that was achieved was Θ = 74.493° (0.80 Å).

The diffraction pattern was indexed, and the total number of runs and images was based on the strategy calculation from the program **CrystalClear** (Rigaku) and the unit cell was refined using **CrystalClear** (Rigaku) on 26196 reflections, 69% of the observed reflections. Data reduction, scaling and absorption corrections were performed using **CrystalClear** (Rigaku). The final completeness is 100.00 % out to 74.493° in Θ. A multi-scan absorption correction was performed using CrysAlisPro 1.171.44.91a (Rigaku Oxford Diffraction, 2025) using spherical harmonics, implemented in SCALE3 ABSPACK scaling algorithm. The absorption coefficient μ of this material is 6.357 mm^{-1} at this wavelength (λ = 1.542Å) and the minimum and maximum transmissions are 0.921 and 1.000.

The structure was solved and the space group $P2_1/c$ (No 14) determined by the olex2.solve 1.5 (Bourhis *et al.,* 2015) structure solution program using Charge Flipping and refined by Least Squares using version 2019/2 of ShelXL 2019/2 (Sheldrick, 2015). All non-hydrogen atoms were refined anisotropically. Hydrogen atom positions were calculated geometrically and refined using the riding model. Hydrogen atom positions were calculated geometrically and refined using the riding model.

Table S. 8: Fractional Atomic Coordinates (×10^4) and Equivalent Isotropic Displacement Parameters (Å^2×10^3) for **2**. U_{eq} is defined as 1/3 of the trace of the orthogonalized U_{ij}.

Atom	x	y	z	U_{eq}
Ru(5)	1761.0(2)	4846.3(2)	7011.5(2)	13.05(5)
Cl(2)	883.0(3)	4739.4(3)	8208.9(2)	20.86(9)
C5	005.3(11)	4701.2(14)	8568.7(11)	22.1(3)
C(1)	735.2(10)	2788.2(13)	6271.6(10)	16.5(3)
C(2)	4002.9(12)	6090.0(14)	9037.2(11)	24.4(3)
C(3)	2714.2(10)	5721.0(13)	6225.4(10)	17.5(3)
C(4)	4534.8(11)	3036.1(15)	7698.9(12)	25.6(4)
C(5)	-821.8(11)	2751.4(14)	5869.0(11)	20.4(3)
C(22)	2576.4(10)	2948.6(13)	7850.5(10)	18.8(3)
C(6)	4313.2(11)	4088.1(14)	8172.8(10)	19.9(3)
C(20)	1551.6(12)	6681.4(14)	6981.8(11)	22.9(3)
C(7)	3298.8(12)	5516.6(14)	8645.4(11)	22.0(3)

C(8)	4085.5(13)	6501.1(17)	5666.4(12)	30.0(4)
C(10)	1862.2(10)	2488.7(13)	7322.0(10)	18.6(3)
C(11)	-1432.1(12)	1448.5(16)	4731.7(12)	27.7(4)
C(12)	-670.0(11)	1954.7(14)	5250.8(10)	19.9(3)
C(13)	200.0(11)	1623.5(14)	5136.9(10)	21.0(3)
C(16)	919.4(11)	2028.2(13)	5636.1(10)	19.2(3)
C(14)	3896.2(12)	4412.5(16)	5702.1(12)	27.6(4)
C(21)	1129.0(11)	5461.2(14)	5809.1(10)	19.6(3)
C(19)	-91.2(12)	6380.3(17)	6616.5(13)	31.6(4)
C(1A)	3450.8(11)	4524.3(14)	8214.5(10)	18.3(3)
C(1B)	865.7(11)	6172.4(14)	6451.9(11)	21.9(3)
C(17)	5648.0(13)	6322.0(16)	9398.5(12)	28.9(4)
C(1C)	1855.3(12)	1670.4(16)	5462.3(12)	27.5(4)
F	3944.8(8)	5168.2(11)	3426.3(9)	42.0(3)
F(1)	3090.7(8)	4413.6(9)	2372.6(7)	33.1(2)
F(2)	3258.3(9)	6285.3(10)	2454.3(11)	49.8(4)
F(3)	2618.6(10)	4392.2(11)	3702.9(8)	43.3(3)
F(4)	2809.7(8)	6247.4(12)	3805.4(11)	52.4(4)
F(5)	1939.6(8)	5506.9(14)	2734.0(10)	49.0(3)
C(1D)	-127.7(11)	3169.8(13)	6378.5(10)	18.5(3)
N(4)	2710.6(9)	4012.2(11)	7767.2(8)	16.7(3)
P(4)	2944.7(3)	5344.4(4)	3077.3(3)	23.71(10)
N(5)	1434.6(8)	3195.7(11)	6836.3(8)	15.4(2)
C(1E)	2042.8(11)	5222.9(13)	5697.0(10)	17.1(3)
C(1F)	2447.7(11)	6477.7(13)	6864.4(10)	19.4(3)
C(1G)	3695.6(11)	5508.4(15)	6139.6(11)	21.8(3)
C(1H)	4868.2(12)	5693.4(14)	8998.6(11)	23.2(3)

Table S.9: Anisotropic Displacement Parameters (×10[4]) **2**. The anisotropic displacement factor exponent takes the form: $-2\pi^2[h^2a^{*2} \times U_{11} + ... + 2hk\, a^* \times b^* \times U_{12}]$

**Atom**	U_{11}	U_{22}	U_{33}	U_{23}	U_{13}	U_{12}
Ru(5)	15.94(7)	10.96(7)	12.42(7)	0.01(4)	2.40(4)	0.49(4)
Cl(2)	25.65(19)	21.82(19)	15.78(17)	-0.22(13)	7.75(14)	0.26(14)
C	21.6(8)	22.0(8)	22.5(8)	3.2(6)	-0.2(6)	-3.5(6)
C(1)	18.0(7)	13.9(7)	17.6(7)	0.9(6)	1.5(6)	-1.4(6)
C(2)	34.5(9)	17.9(8)	20.5(8)	-2.8(6)	-0.1(7)	-2.6(7)
C(3)	21.6(7)	14.5(7)	16.6(7)	6.4(6)	3.9(6)	-3.1(6)
C(4)	21.9(8)	21.9(8)	32.7(9)	-4.3(7)	-1.1(7)	1.4(6)
C(5)	17.4(7)	20.9(8)	23.1(8)	5.2(6)	1.8(6)	0.4(6)
C(22)	19.2(7)	16.2(7)	20.8(8)	3.2(6)	0.1(6)	-0.5(6)
C(6)	23.8(8)	17.3(8)	18.6(7)	1.6(6)	0.6(6)	-1.8(6)
C(20)	36.4(9)	10.6(7)	22.3(8)	2.4(6)	8.2(7)	4.3(6)
C(7)	26.9(8)	19.6(8)	19.6(8)	-0.1(6)	1.3(6)	1.0(6)
C(8)	28.9(9)	31.9(10)	29.8(9)	5.8(8)	6.9(7)	-10.1(7)
C(10)	19.2(7)	13.8(7)	23.0(8)	1.4(6)	2.3(6)	-0.6(6)

C(11)	28.8(9)	27.4(9)	26.0(9)	4.3(7)	-6.8(7)	-8.5(7)
C(12)	23.1(8)	18.3(8)	18.1(7)	5.0(6)	-1.7(6)	-4.7(6)
C(13)	27.0(8)	17.9(8)	18.1(7)	-2.3(6)	2.3(6)	-3.8(6)
C(16)	20.3(7)	16.6(7)	21.0(8)	-0.2(6)	4.9(6)	-0.7(6)
C(14)	22.1(8)	29.1(9)	32.4(9)	2.8(8)	8.8(7)	4.7(7)
C(21)	21.2(7)	20.1(8)	17.3(7)	5.8(6)	-0.7(6)	1.5(6)
C(19)	27.3(9)	32.1(10)	36.1(10)	9.5(8)	9.7(7)	13.2(7)
C(1A)	22.3(7)	16.9(7)	15.4(7)	2.3(6)	-1.4(6)	-2.5(6)
C(1B)	24.0(8)	18.0(8)	24.3(8)	8.6(6)	5.1(6)	7.4(6)
C(17)	34.4(9)	25.7(9)	26.0(9)	0.2(7)	-3.4(7)	-11.0(7)
C(1C)	22.7(8)	27.8(9)	32.5(9)	-11.1(7)	7.0(7)	0.2(7)
F	24.1(6)	55.1(8)	46.1(7)	-11.5(6)	-5.2(5)	11.6(5)
F(1)	42.7(6)	27.7(6)	30.2(6)	-7.6(5)	12.4(5)	-10.8(5)
F(2)	39.2(7)	22.0(6)	89.4(11)	19.6(6)	14.2(7)	2.9(5)
F(3)	61.3(8)	37.9(7)	32.5(6)	-4.1(5)	20.1(6)	-5.6(6)
F(4)	31.6(6)	41.5(7)	84.6(11)	-41.2(7)	8.5(6)	-1.9(5)
F(5)	19.7(5)	66.1(9)	60.4(9)	-1.3(7)	-4.6(5)	1.5(6)
C(1D)	20.3(7)	17.3(7)	18.2(7)	0.8(6)	3.2(6)	1.5(6)
N(4)	18.6(6)	16.2(6)	15.4(6)	0.4(5)	1.0(5)	0.8(5)
P(4)	18.7(2)	17.0(2)	35.4(2)	-5.49(17)	0.88(17)	-0.40(15)
N(5)	16.4(6)	13.1(6)	16.8(6)	-1.2(5)	3.4(5)	0.3(5)
C(1E)	22.2(8)	16.6(8)	12.7(7)	3.2(5)	3.1(6)	0.1(6)
C(1F)	28.7(8)	10.4(7)	19.1(7)	3.3(6)	2.1(6)	-4.8(6)
C(1G)	19.6(7)	25.1(8)	20.9(8)	4.2(7)	2.0(6)	-3.2(6)
C(1H)	29.2(8)	20.5(8)	19.5(8)	2.8(6)	-2.0(6)	-7.9(7)

Table S.10: Bond Lengths in Å for **2**.

**Atom**	**Atom**	**Length/Å**
Ru(5)	Cl(2)	2.3744(4)
Ru(5)	C(3)	2.2144(15)
Ru(5)	C(20)	2.2204(16)
Ru(5)	C(21)	2.2151(16)
Ru(5)	C(1B)	2.2387(16)
Ru(5)	N(4)	2.0765(13)
Ru(5)	N(5)	2.0526(13)
Ru(5)	C(1E)	2.1970(15)
Ru(5)	C(1F)	2.2290(15)
C	C(6)	1.399(2)
C	C(1H)	1.391(3)
C(1)	C(16)	1.397(2)
C(1)	C(1D)	1.398(2)
C(1)	N(5)	1.434(2)
C(2)	C(7)	1.385(2)
C(2)	C(1H)	1.393(3)
C(3)	C(1E)	1.413(2)

C(3)	C(1F)	1.433(2)
C(3)	C(1G)	1.515(2)
C(4)	C(6)	1.513(2)
C(5)	C(12)	1.396(2)
C(5)	C(1D)	1.384(2)
C(22)	C(10)	1.440(2)
C(22)	N(4)	1.297(2)
C(6)	C(1A)	1.406(2)
C(20)	C(1B)	1.435(3)
C(20)	C(1F)	1.395(3)
C(7)	C(1A)	1.396(2)
C(8)	C(1G)	1.539(2)
C(10)	N(5)	1.295(2)
C(11)	C(12)	1.507(2)
C(12)	C(13)	1.391(2)
C(13)	C(16)	1.397(2)
C(16)	C(1C)	1.514(2)
C(14)	C(1G)	1.522(3)
C(21)	C(1B)	1.402(2)
C(21)	C(1E)	1.427(2)
C(19)	C(1B)	1.501(2)
C(1A)	N(4)	1.430(2)
C(17)	C(1H)	1.509(2)
F	P(4)	1.5940(12)
F(1)	P(4)	1.6022(11)
F(2)	P(4)	1.5871(14)
F(3)	P(4)	1.6055(13)
F(4)	P(4)	1.6032(13)
F(5)	P(4)	1.5964(12)

Table S.11: Bond Angles in ° for **2**.

**Atom**	**Atom**	**Atom**	**Angle/°**
C(3)	Ru(5)	Cl(2)	151.76(4)
C(3)	Ru(5)	C(20)	67.39(6)
C(3)	Ru(5)	C(21)	67.70(6)
C(3)	Ru(5)	C(1B)	80.60(6)
C(3)	Ru(5)	C(1F)	37.62(6)
C(20)	Ru(5)	Cl(2)	89.19(4)
C(20)	Ru(5)	C(1B)	37.55(7)
C(20)	Ru(5)	C(1F)	36.54(6)
C(21)	Ru(5)	Cl(2)	118.32(4)
C(21)	Ru(5)	C(20)	66.31(6)
C(21)	Ru(5)	C(1B)	36.69(6)
C(21)	Ru(5)	C(1F)	78.47(6)
C(1B)	Ru(5)	Cl(2)	90.35(4)
N(4)	Ru(5)	Cl(2)	84.73(4)

N(4)	Ru(5)	C(3)	96.00(5)
N(4)	Ru(5)	C(20)	125.56(6)
N(4)	Ru(5)	C(21)	155.68(6)
N(4)	Ru(5)	C(1B)	162.67(6)
N(4)	Ru(5)	C(1E)	119.08(6)
N(4)	Ru(5)	C(1F)	99.89(6)
N(5)	Ru(5)	Cl(2)	85.23(4)
N(5)	Ru(5)	C(3)	122.50(6)
N(5)	Ru(5)	C(20)	156.55(6)
N(5)	Ru(5)	C(21)	96.53(6)
N(5)	Ru(5)	C(1B)	119.59(6)
N(5)	Ru(5)	N(4)	76.62(5)
N(5)	Ru(5)	C(1E)	97.22(5)
N(5)	Ru(5)	C(1F)	159.95(6)
C(1E)	Ru(5)	Cl(2)	156.04(4)
C(1E)	Ru(5)	C(3)	37.37(6)
C(1E)	Ru(5)	C(20)	79.10(6)
C(1E)	Ru(5)	C(21)	37.75(6)
C(1E)	Ru(5)	C(1B)	67.65(6)
C(1E)	Ru(5)	C(1F)	66.87(6)
C(1F)	Ru(5)	Cl(2)	114.34(4)
C(1F)	Ru(5)	C(1B)	67.09(6)
C(1H)	C	C(6)	122.86(16)
C(16)	C(1)	C(1D)	121.43(15)
C(16)	C(1)	N(5)	120.37(14)
C(1D)	C(1)	N(5)	118.20(14)
C(7)	C(2)	C(1H)	120.64(16)
C(1E)	C(3)	Ru(5)	70.65(9)
C(1E)	C(3)	C(1F)	117.94(15)
C(1E)	C(3)	C(1G)	123.33(15)
C(1F)	C(3)	Ru(5)	71.74(9)
C(1F)	C(3)	C(1G)	118.73(15)
C(1G)	C(3)	Ru(5)	129.54(11)
C(1D)	C(5)	C(12)	120.79(15)
N(4)	C(22)	C(10)	115.59(14)
C	C(6)	C(4)	118.80(15)
C	C(6)	C(1A)	117.04(15)
C(1A)	C(6)	C(4)	124.11(15)
C(1B)	C(20)	Ru(5)	71.92(9)
C(1F)	C(20)	Ru(5)	72.06(9)
C(1F)	C(20)	C(1B)	121.45(15)
C(2)	C(7)	C(1A)	120.04(16)
N(5)	C(10)	C(22)	115.56(14)
C(5)	C(12)	C(11)	120.76(16)
C(13)	C(12)	C(5)	118.41(15)
C(13)	C(12)	C(11)	120.82(16)
C(12)	C(13)	C(16)	122.53(15)

C(1)	C(16)	C(13)	117.26(15)
C(1)	C(16)	C(1C)	122.52(15)
C(13)	C(16)	C(1C)	120.19(15)
C(1B)	C(21)	Ru(5)	72.57(9)
C(1B)	C(21)	C(1E)	121.56(15)
C(1E)	C(21)	Ru(5)	70.44(9)
C(6)	C(1A)	N(4)	121.18(15)
C(7)	C(1A)	C(6)	120.98(15)
C(7)	C(1A)	N(4)	117.69(14)
C(20)	C(1B)	Ru(5)	70.54(9)
C(20)	C(1B)	C(19)	119.90(16)
C(21)	C(1B)	Ru(5)	70.74(9)
C(21)	C(1B)	C(20)	117.51(15)
C(21)	C(1B)	C(19)	122.56(17)
C(19)	C(1B)	Ru(5)	127.92(12)
C(5)	C(1D)	C(1)	119.48(15)
C(22)	N(4)	Ru(5)	115.12(11)
C(22)	N(4)	C(1A)	119.49(14)
C(1A)	N(4)	Ru(5)	125.33(11)
F	P(4)	F(1)	89.38(7)
F	P(4)	F(3)	90.31(8)
F	P(4)	F(4)	89.52(7)
F	P(4)	F(5)	179.28(9)
F(1)	P(4)	F(3)	89.71(6)
F(1)	P(4)	F(4)	178.14(9)
F(2)	P(4)	F	90.21(8)
F(2)	P(4)	F(1)	90.18(8)
F(2)	P(4)	F(3)	179.47(9)
F(2)	P(4)	F(4)	91.32(9)
F(2)	P(4)	F(5)	90.50(8)
F(4)	P(4)	F(3)	88.81(8)
F(5)	P(4)	F(1)	90.49(8)
F(5)	P(4)	F(3)	88.98(8)
F(5)	P(4)	F(4)	90.60(8)
C(1)	N(5)	Ru(5)	125.16(10)
C(10)	N(5)	Ru(5)	116.13(11)
C(10)	N(5)	C(1)	118.53(13)
C(3)	C(1E)	Ru(5)	71.98(9)
C(3)	C(1E)	C(21)	120.57(15)
C(21)	C(1E)	Ru(5)	71.81(9)
C(3)	C(1F)	Ru(5)	70.64(8)
C(20)	C(1F)	Ru(5)	71.39(9)
C(20)	C(1F)	C(3)	120.92(15)
C(3)	C(1G)	C(8)	108.27(14)
C(3)	C(1G)	C(14)	113.81(14)
C(14)	C(1G)	C(8)	110.71(15)
C	C(1H)	C(2)	118.42(15)

C	C(1H)	C(17)	120.03(16)
C(2)	C(1H)	C(17)	121.55(16)

Table S.12: Torsion Angles in ° for **2**.

**Atom**	**Atom**	**Atom**	**Atom**	**Angle/°**
Ru(5)	C(3)	C(1E)	C(21)	55.01(13)
Ru(5)	C(3)	C(1F)	C(20)	-52.79(14)
Ru(5)	C(3)	C(1G)	C(8)	-167.89(12)
Ru(5)	C(3)	C(1G)	C(14)	68.55(19)
Ru(5)	C(20)	C(1B)	C(21)	-54.66(13)
Ru(5)	C(20)	C(1B)	C(19)	123.28(15)
Ru(5)	C(20)	C(1F)	C(3)	52.45(13)
Ru(5)	C(21)	C(1B)	C(20)	54.55(13)
Ru(5)	C(21)	C(1B)	C(19)	-123.32(16)
Ru(5)	C(21)	C(1E)	C(3)	-55.09(13)
C	C(6)	C(1A)	C(7)	-1.0(2)
C	C(6)	C(1A)	N(4)	174.40(14)
C(2)	C(7)	C(1A)	C(6)	0.3(3)
C(2)	C(7)	C(1A)	N(4)	-175.29(15)
C(4)	C(6)	C(1A)	C(7)	-178.26(16)
C(4)	C(6)	C(1A)	N(4)	-2.8(2)
C(5)	C(12)	C(13)	C(16)	2.3(2)
C(22)	C(10)	N(5)	Ru(5)	-6.77(18)
C(22)	C(10)	N(5)	C(1)	177.84(14)
C(6)	C	C(1H)	C(2)	0.2(3)
C(6)	C	C(1H)	C(17)	-178.94(16)
C(6)	C(1A)	N(4)	Ru(5)	-131.71(14)
C(6)	C(1A)	N(4)	C(22)	51.3(2)
C(7)	C(2)	C(1H)	C	-0.9(3)
C(7)	C(2)	C(1H)	C(17)	178.15(16)
C(7)	C(1A)	N(4)	Ru(5)	43.85(19)
C(7)	C(1A)	N(4)	C(22)	-133.11(16)
C(10)	C(22)	N(4)	Ru(5)	7.87(18)
C(10)	C(22)	N(4)	C(1A)	-174.87(14)
C(11)	C(12)	C(13)	C(16)	-176.46(16)
C(12)	C(5)	C(1D)	C(1)	0.0(2)
C(12)	C(13)	C(16)	C(1)	0.4(2)
C(12)	C(13)	C(16)	C(1C)	-177.75(16)
C(16)	C(1)	C(1D)	C(5)	2.8(2)
C(16)	C(1)	N(5)	Ru(5)	122.50(14)
C(16)	C(1)	N(5)	C(10)	-62.6(2)
C(1B)	C(20)	C(1F)	Ru(5)	-54.52(14)
C(1B)	C(20)	C(1F)	C(3)	-2.1(2)
C(1B)	C(21)	C(1E)	Ru(5)	53.75(14)
C(1B)	C(21)	C(1E)	C(3)	-1.3(2)
C(1D)	C(1)	C(16)	C(13)	-3.0(2)

C(1D)	C(1)	C(16)	C(1C)	175.10(16)
C(1D)	C(1)	N(5)	Ru(5)	-57.70(18)
C(1D)	C(1)	N(5)	C(10)	117.23(17)
C(1D)	C(5)	C(12)	C(11)	176.29(15)
C(1D)	C(5)	C(12)	C(13)	-2.5(2)
N(4)	C(22)	C(10)	N(5)	-0.8(2)
N(5)	C(1)	C(16)	C(13)	176.82(14)
N(5)	C(1)	C(16)	C(1C)	-5.1(2)
N(5)	C(1)	C(1D)	C(5)	-176.96(14)
C(1E)	C(3)	C(1F)	Ru(5)	55.25(12)
C(1E)	C(3)	C(1F)	C(20)	2.5(2)
C(1E)	C(3)	C(1G)	C(8)	100.48(18)
C(1E)	C(3)	C(1G)	C(14)	-23.1(2)
C(1E)	C(21)	C(1B)	Ru(5)	-52.79(14)
C(1E)	C(21)	C(1B)	C(20)	1.8(2)
C(1E)	C(21)	C(1B)	C(19)	-176.12(15)
C(1F)	C(3)	C(1E)	Ru(5)	-55.80(12)
C(1F)	C(3)	C(1E)	C(21)	-0.8(2)
C(1F)	C(3)	C(1G)	C(8)	-78.51(18)
C(1F)	C(3)	C(1G)	C(14)	157.93(15)
C(1F)	C(20)	C(1B)	Ru(5)	54.59(14)
C(1F)	C(20)	C(1B)	C(21)	-0.1(2)
C(1F)	C(20)	C(1B)	C(19)	177.87(15)
C(1G)	C(3)	C(1E)	Ru(5)	125.21(15)
C(1G)	C(3)	C(1E)	C(21)	-179.78(14)
C(1G)	C(3)	C(1F)	Ru(5)	-125.70(14)
C(1G)	C(3)	C(1F)	C(20)	-178.50(14)
C(1H)	C	C(6)	C(4)	178.19(16)
C(1H)	C	C(6)	C(1A)	0.8(3)
C(1H)	C(2)	C(7)	C(1A)	0.7(3)

Table S.13: Hydrogen Fractional Atomic Coordinates (×10^4) and Equivalent Isotropic Displacement Parameters (Å^2×10^3) for **2**. U_{eq} is defined as 1/3 of the trace of the orthogonalized U_{ij}.

**Atom**	**x**	**y**	**z**	***U_{eq}***
H	5594.66	4428.03	8542.9	26
H(2)	3894.7	6760.32	9335.15	29
H(4A)	5142.69	3086.38	7511.64	38
H(4B)	4120.28	2952.14	7207.06	38
H(4C)	4484.77	2388.68	8071.14	38
H(5)	-1408.82	3009.52	5940.85	25
H(22)	2926.12	2502.66	8234.76	23
H(20)	1391.95	7167.75	7421.98	27
H(7)	2711.98	5798.82	8669.87	26
H(8A)	3826.07	6522.5	5087.73	45
H(8B)	4730.88	6415.19	5653.98	45

H(8C)	3947.74	7197.78	5956.11	45
H(10)	1714.63	1717.64	7329.42	22
H(11A)	-1648.84	786.44	5020.16	42
H(11B)	-1230.24	1233.66	4177.25	42
H(11C)	-1912.82	1996.22	4659.96	42
H(13)	308.98	1102.25	4702.98	25
H(14A)	3567.68	3805.63	5958.88	41
H(14B)	4534.36	4258.54	5764.26	41
H(14C)	3715.64	4467.98	5101.58	41
H(21)	688.9	5130.08	5439.32	23
H(19A)	-467.62	5883.49	6261.89	47
H(19B)	-240.97	7158.94	6483.22	47
H(19C)	-190.12	6234.24	7212.05	47
H(17A)	5916.19	5882.65	9866.02	43
H(17B)	5444.99	7040.67	9612.4	43
H(17C)	6089.32	6450.95	8976.67	43
H(1CA)	2255.46	2312.8	5527.86	41
H(1CB)	1868.47	1382.85	4884.99	41
H(1CC)	2047.91	1084	5861.67	41
H(1D)	-236.69	3712.06	6797.65	22
H(1E)	2201.1	4725.09	5263.41	21
H(1F)	2887.26	6845.7	7213.11	23
H(1G)	3987.69	5486.22	6719.37	26

Citations

- Bourhis, L. J., Dolomanov, O. V., Gildea, R. J., Howard, J. A. K. & Puschmann, H. (2015). The anatomy of a comprehensive constrained, restrained refinement program for the modern computing environment - Olex2 dissected. Acta Cryst. A71, 59 – 75.
- Sheldrick, G.M. (2015) Crystal Structure Refinement with SHELXL. Acta Crystallographica C, C71, 3-8.
- O.V. Dolomanov and L.J. Bourhis and R.J. Gildea and J.A.K. Howard and H. Puschmann, Olex2: A complete structure solution, refinement and analysis program, *J. Appl. Cryst.*, (2009), **42**, 339-341.
- Oxford Diffraction, CrysAlis PRO, Oxford Diffraction Ltd, Yarnton, England, 2025.

Crystal Data and Experimental for 5

Experimental. Single yellow block-shaped crystals of **5** were obtained by recrystallisation from slow diffusion of a dichloromethane-hexamethyldisiloxane solution at low temperature (- 8 °C). A suitable crystal 0.17×0.17×0.10 mm^3 was selected and mounted on a suitable support on a Rigaku XtaLAB Synergy-S Dualflex, HyPix diffractometer. The crystal was kept at a steady T = 100.01(18) K during data collection. The structure was solved with the ShelXT (Sheldrick, 2015) structure solution program using the dual solution method and by using **Olex2** (Dolomanov et al., 2009) as the graphical interface. The model was refined with version 2019/2 of ShelXL 2019/2 (Sheldrick, 2015) using Least Squares minimization. The structure was deposited in the Cambridge Structural Database, with CCDC number 2450156 for **5**.

Crystal Data. $C_{24}H_{24}ClF_8N_2PRu$, M_r = 659.94, monoclinic, $P2_1/c$ (No. 14), a = 10.42187(11) Å, b = 20.3425(2) Å, c = 12.84344(15) Å, β = 112.1083(13)°, V = 2522.69(5) Å^3, T = 100.01(18) K, Z = 4, Z' = 1, μ(Cu K_α) = 7.313, 30268 reflections measured, 5151 unique (R_{int} = 0.0436) which were used in all calculations. The final wR_2 was 0.0737 (all data) and R_1 was 0.0272 (I > 2σ(I)).

Data were measured using profile data from ω-scans of 0.5° per frame for 0.9/0.2 s using Cu K_a radiation. The diffraction pattern was indexed and the total number of and images was based on the strategy calculation from the program **CrystalClear** (Rigaku) The maximum resolution that was achieved was Θ = 74.498° (0.80 Å). The diffraction pattern was indexed, and the total number of runs and images was based on the strategy calculation from the program **CrystalClear** (Rigaku) and the unit cell was refined using **CrystalClear** (Rigaku) on 20220 reflections, 67% of the observed reflections.

Data reduction, scaling and absorption corrections were performed using **CrystalClear** (Rigaku). The final completeness is 100.00 % out to 74.498° in Θ. A multi-

scan absorption correction was performed using CrysAlisPro 1.171.44.91a (Rigaku Oxford Diffraction, 2025) using spherical harmonics,implemented in SCALE3 ABSPACK scaling algorithm. The absorption coefficient μ of this material is 7.313 mm^{-1} at this wavelength (λ = 1.542Å) and the minimum and maximum transmissions are 0.693 and 1.000.

The structure was solved and the space group $P2_1/c$ (No 14) determined by the ShelXT (Sheldrick, 2015) structure solution program using dual and refined by Least Squares using version 2019/2 of ShelXL 2019/2 (Sheldrick, 2015). All non-hydrogen atoms were refined anisotropically. Hydrogen atom positions were calculated geometrically and refined using the riding model. Hydrogen atom positions were calculated geometrically and refined using the riding model.

Table S.14: Fractional Atomic Coordinates (×10^4) and Equivalent Isotropic Displacement Parameters ($Å^2$×10^3) for **5**. U_{eq} is defined as 1/3 of the trace of the orthogonalised U_{ij}.

**Atom**	**x**	**y**	**z**	U_{eq}
Ru(1)	2581.9(2)	6124.7(2)	2803.6(2)	15.95(7)
Cl(2)	792.3(5)	6242.2(3)	1012.1(4)	23.47(12)
P(3)	7262.5(6)	4436.0(3)	2406.6(5)	22.66(13)
F(7)	6939.9(14)	4855.9(7)	3340.4(12)	28.5(3)
F(5)	5858.7(14)	4019.9(7)	2172.2(12)	28.9(3)
F(4)	7566.5(16)	4004.7(7)	1482.3(13)	32.7(3)
F(9)	8085.5(16)	3910.8(7)	3353.3(14)	35.2(3)
F(26)	-2241.9(16)	6557.3(8)	5220.2(16)	41.0(4)
F(6)	8647.8(15)	4844.9(8)	2643.2(16)	38.3(4)
F(8)	6402.3(16)	4951.8(8)	1471.2(14)	35.5(3)
F(27)	7380.4(17)	6920.9(8)	491.7(17)	43.3(4)
N(19)	1635.8(18)	6900.0(9)	3298.9(16)	20.5(4)
N(16)	3466.8(18)	6955.2(9)	2416.3(16)	19.5(3)
C(32)	2645(2)	5506.3(11)	4222.6(19)	20.7(4)
C(34)	1875(2)	4636.7(11)	1398.8(19)	22.2(4)
C(29)	3950(2)	5314.5(10)	2678.1(18)	18.8(4)
C(31)	3985(2)	5786.8(10)	4477.0(18)	20.5(4)
C(10)	4498(2)	6946.5(11)	1932.3(19)	21.1(4)
C(33)	1979(2)	5145.8(10)	3230.7(19)	20.5(4)
C(20)	616(2)	6824.7(11)	3782(2)	21.4(4)
C(21)	585(2)	7253.2(11)	4619(2)	24.3(4)
C(17)	3040(2)	7516.6(11)	2636(2)	24.8(5)

C(14)	6675(2)	7315.1(12)	1910(2)	27.8(5)
C(30)	4608(2)	5689.5(10)	3676.7(19)	20.1(4)
C(18)	1997(2)	7485.0(11)	3116(2)	25.1(5)
C(28)	2633(2)	5037.1(10)	2444.9(19)	19.0(4)
C(13)	6427(3)	6933.1(12)	972(2)	29.8(5)
C(11)	4283(2)	6555.8(12)	996(2)	26.1(5)
C(25)	-332(2)	6308.2(12)	3426(2)	26.1(5)
C(15)	5692(2)	7327.6(11)	2399(2)	25.0(4)
C(37)	4665(3)	6198.6(12)	5505(2)	26.5(5)
C(36)	2378(2)	4749.0(12)	443(2)	27.2(5)
C(23)	-1298(2)	6650.5(12)	4739(2)	28.3(5)
C(12)	5258(3)	6551.0(12)	501(2)	29.4(5)
C(24)	-1300(3)	6221.8(12)	3905(3)	30.4(5)
C(22)	-386(2)	7168.8(12)	5104(2)	26.6(5)
C(35)	2011(3)	3911.0(11)	1748(2)	29.9(5)

Table S.15: Anisotropic Displacement Parameters (×10^4) **5**. The anisotropic displacement factor exponent takes the form: $-2\pi^2[h^2a^{*2} \times U_{11} + ... + 2hka^* \times b^* \times U_{12}]$.

**Atom**	***U*₁₁**	***U*₂₂**	***U*₃₃**	***U*₂₃**	***U*₁₃**	***U*₁₂**
Ru(1)	16.50(9)	12.31(10)	19.9(1)	-0.13(5)	7.83(7)	0.00(5)
Cl(2)	23.4(2)	18.7(2)	24.2(3)	1.68(19)	4.29(19)	1.18(18)
P(3)	24.0(3)	16.9(3)	29.3(3)	-0.3(2)	12.6(2)	-0.6(2)
F(7)	30.3(7)	24.0(7)	34.6(7)	-4.4(6)	16.0(6)	-3.0(5)
F(5)	27.2(7)	27.3(7)	33.0(7)	-2.9(6)	12.2(6)	-5.9(5)
F(4)	38.7(8)	26.5(7)	39.4(8)	-4.6(6)	22.1(7)	0.6(6)
F(9)	34.2(8)	28.8(8)	36.0(8)	4.0(6)	5.7(7)	6.3(6)
F(26)	39.1(8)	38.0(9)	62.4(11)	-13.5(8)	37.8(8)	-10.6(6)
F(6)	31.4(7)	29.2(8)	63.0(11)	-11.6(7)	27.6(7)	-9.6(6)
F(8)	43.7(8)	29.6(8)	39.3(8)	11.3(6)	22.7(7)	10.1(6)
F(27)	43.7(9)	38.1(9)	66.7(11)	-11.0(8)	41.8(9)	-9.7(7)
N(19)	19.9(8)	15.7(8)	26.9(9)	-1.7(7)	10.1(7)	1.8(6)
N(16)	21.6(8)	14.2(8)	23.6(9)	1.0(7)	9.7(7)	-1.3(6)
C(32)	24.8(10)	17.2(10)	21.7(10)	6.4(8)	10.5(8)	2.7(8)
C(34)	23.4(10)	16.4(10)	24.9(11)	0.0(8)	6.8(8)	-0.9(8)
C(29)	19.6(9)	14.3(9)	24.0(10)	1.0(8)	10.0(8)	6.8(7)
C(31)	21.1(9)	18.0(10)	19.6(10)	3.5(8)	4.4(8)	3.0(8)
C(10)	23.7(10)	16.4(10)	25.5(11)	3.0(8)	11.9(9)	-0.7(8)
C(33)	20.7(9)	13.6(9)	27.8(11)	3.9(8)	9.8(8)	-1.3(7)
C(20)	21.4(9)	15.8(10)	29.4(11)	-0.5(8)	12.4(9)	2.2(8)
C(21)	22.6(10)	17.8(10)	33.6(12)	-1.9(9)	11.6(9)	-0.4(8)
C(17)	28.6(11)	17.1(11)	32.0(12)	0.7(9)	15.3(10)	-1.6(8)
C(14)	25.7(11)	21.1(11)	39.6(14)	-0.3(9)	15.9(10)	-3.8(9)
C(30)	14.7(9)	16.8(10)	26.5(11)	2.9(8)	5.3(8)	3.6(7)
C(18)	29.4(11)	15.3(10)	34.6(12)	-1.2(9)	16.8(10)	0.5(8)
C(28)	23.2(10)	9.4(9)	22.9(10)	2.2(8)	6.8(8)	2.6(7)
C(13)	31.0(12)	23.6(12)	43.9(14)	0.9(10)	24.5(11)	-2.0(9)

C(11)	26.7(10)	23.8(11)	30.2(12)	-2.4(9)	13.5(9)	-2.9(9)
C(25)	25.5(11)	20.3(11)	34.7(13)	-5.0(9)	14.0(10)	0.1(8)
C(15)	29.1(11)	17.2(10)	30.2(12)	-1.7(9)	12.8(9)	-2.7(8)
C(37)	28.7(11)	26.5(12)	22.8(11)	-3.3(9)	8.0(9)	-4.3(9)
C(36)	29.4(11)	26.1(12)	25.1(11)	-4.9(9)	9.1(9)	-1.8(9)
C(23)	26.5(11)	24.0(11)	40.9(13)	-1.7(10)	20.1(10)	-0.9(9)
C(12)	34.4(12)	25.4(12)	35.2(13)	-4.3(10)	20.6(11)	-4.2(9)
C(24)	27.4(11)	22.3(11)	46.0(15)	-7.9(10)	19.0(11)	-4.1(9)
C(22)	24.8(10)	24.6(11)	32.3(12)	-5.8(9)	12.8(9)	0.5(9)
C(35)	38.5(13)	15.2(11)	27.5(12)	-1.0(8)	2.7(10)	-1.5(9)

Table S.16: Bond Lengths in Å for **5**.

**Atom**	**Atom**	**Length/Å**
Ru(1)	Cl(2)	2.3663(5)
Ru(1)	N(19)	2.0824(18)
Ru(1)	N(16)	2.0729(17)
Ru(1)	C(32)	2.195(2)
Ru(1)	C(29)	2.2241(19)
Ru(1)	C(31)	2.206(2)
Ru(1)	C(33)	2.218(2)
Ru(1)	C(30)	2.172(2)
Ru(1)	C(28)	2.265(2)
P(3)	F(7)	1.6076(15)
P(3)	F(5)	1.6174(14)
P(3)	F(4)	1.6008(15)
P(3)	F(9)	1.6033(16)
P(3)	F(6)	1.5933(15)
P(3)	F(8)	1.5928(16)
F(26)	C(23)	1.358(3)
F(27)	C(13)	1.353(3)
N(19)	C(20)	1.426(3)
N(19)	C(18)	1.296(3)
N(16)	C(10)	1.429(3)
N(16)	C(17)	1.295(3)
C(32)	C(31)	1.429(3)
C(32)	C(33)	1.406(3)
C(34)	C(28)	1.515(3)
C(34)	C(36)	1.523(3)
C(34)	C(35)	1.534(3)
C(29)	C(30)	1.427(3)
C(29)	C(28)	1.408(3)
C(31)	C(30)	1.421(3)
C(31)	C(37)	1.497(3)
C(10)	C(11)	1.388(3)
C(10)	C(15)	1.396(3)
C(33)	C(28)	1.431(3)

C(20)	C(21)	1.394(3)
C(20)	C(25)	1.396(3)
C(21)	C(22)	1.384(3)
C(17)	C(18)	1.439(3)
C(14)	C(13)	1.374(4)
C(14)	C(15)	1.389(3)
C(13)	C(12)	1.378(4)
C(11)	C(12)	1.386(3)
C(25)	C(24)	1.377(3)
C(23)	C(24)	1.380(4)
C(23)	C(22)	1.378(3)

Table S. 17: Bond Angles in ° for **5.**

**Atom**	**Atom**	**Atom**	**Angle/°**
N(19)	Ru(1)	Cl(2)	85.99(5)
N(19)	Ru(1)	C(32)	92.86(8)
N(19)	Ru(1)	C(29)	166.75(8)
N(19)	Ru(1)	C(31)	98.91(8)
N(19)	Ru(1)	C(33)	113.17(7)
N(19)	Ru(1)	C(30)	129.13(8)
N(19)	Ru(1)	C(28)	148.43(7)
N(16)	Ru(1)	Cl(2)	86.75(5)
N(16)	Ru(1)	N(19)	76.15(7)
N(16)	Ru(1)	C(32)	142.12(8)
N(16)	Ru(1)	C(29)	103.38(7)
N(16)	Ru(1)	C(31)	107.22(8)
N(16)	Ru(1)	C(33)	169.72(7)
N(16)	Ru(1)	C(30)	91.28(8)
N(16)	Ru(1)	C(28)	134.50(7)
C(32)	Ru(1)	Cl(2)	129.16(6)
C(32)	Ru(1)	C(29)	79.39(8)
C(32)	Ru(1)	C(31)	37.89(8)
C(32)	Ru(1)	C(33)	37.15(9)
C(32)	Ru(1)	C(28)	67.25(8)
C(29)	Ru(1)	Cl(2)	107.25(6)
C(29)	Ru(1)	C(28)	36.55(8)
C(31)	Ru(1)	Cl(2)	165.91(6)
C(31)	Ru(1)	C(29)	68.38(8)
C(31)	Ru(1)	C(33)	67.98(8)
C(31)	Ru(1)	C(28)	80.56(8)
C(33)	Ru(1)	Cl(2)	97.94(6)
C(33)	Ru(1)	C(29)	66.52(8)
C(33)	Ru(1)	C(28)	37.23(8)
C(30)	Ru(1)	Cl(2)	143.18(6)
C(30)	Ru(1)	C(32)	67.56(8)
C(30)	Ru(1)	C(29)	37.85(8)

C(30)	Ru(1)	C(31)	37.87(8)
C(30)	Ru(1)	C(33)	79.47(8)
C(30)	Ru(1)	C(28)	67.31(8)
C(28)	Ru(1)	Cl(2)	88.34(6)
F(7)	P(3)	F(5)	89.34(7)
F(4)	P(3)	F(7)	178.76(8)
F(4)	P(3)	F(5)	89.58(8)
F(4)	P(3)	F(9)	89.91(9)
F(9)	P(3)	F(7)	89.47(8)
F(9)	P(3)	F(5)	88.89(8)
F(6)	P(3)	F(7)	90.53(8)
F(6)	P(3)	F(5)	179.74(10)
F(6)	P(3)	F(4)	90.55(8)
F(6)	P(3)	F(9)	90.88(9)
F(8)	P(3)	F(7)	89.79(8)
F(8)	P(3)	F(5)	89.51(8)
F(8)	P(3)	F(4)	90.81(9)
F(8)	P(3)	F(9)	178.25(9)
F(8)	P(3)	F(6)	90.71(9)
C(20)	N(19)	Ru(1)	124.59(14)
C(18)	N(19)	Ru(1)	115.91(15)
C(18)	N(19)	C(20)	119.48(18)
C(10)	N(16)	Ru(1)	124.70(14)
C(17)	N(16)	Ru(1)	116.49(15)
C(17)	N(16)	C(10)	118.81(18)
C(31)	C(32)	Ru(1)	71.45(12)
C(33)	C(32)	Ru(1)	72.32(12)
C(33)	C(32)	C(31)	121.48(19)
C(28)	C(34)	C(36)	114.30(18)
C(28)	C(34)	C(35)	107.36(18)
C(36)	C(34)	C(35)	111.0(2)
C(30)	C(29)	Ru(1)	69.10(11)
C(28)	C(29)	Ru(1)	73.29(11)
C(28)	C(29)	C(30)	120.40(19)
C(32)	C(31)	Ru(1)	70.66(12)
C(32)	C(31)	C(37)	121.5(2)
C(30)	C(31)	Ru(1)	69.78(12)
C(30)	C(31)	C(32)	116.9(2)
C(30)	C(31)	C(37)	121.51(19)
C(37)	C(31)	Ru(1)	127.21(16)
C(11)	C(10)	N(16)	118.80(19)
C(11)	C(10)	C(15)	121.1(2)
C(15)	C(10)	N(16)	120.1(2)
C(32)	C(33)	Ru(1)	70.53(12)
C(32)	C(33)	C(28)	121.05(19)
C(28)	C(33)	Ru(1)	73.14(11)
C(21)	C(20)	N(19)	120.4(2)

C(21)	C(20)	C(25)	120.3(2)
C(25)	C(20)	N(19)	119.3(2)
C(22)	C(21)	C(20)	120.2(2)
N(16)	C(17)	C(18)	115.55(19)
C(13)	C(14)	C(15)	118.7(2)
C(29)	C(30)	Ru(1)	73.06(11)
C(31)	C(30)	Ru(1)	72.35(11)
C(31)	C(30)	C(29)	121.92(19)
N(19)	C(18)	C(17)	115.87(19)
C(34)	C(28)	Ru(1)	131.42(15)
C(29)	C(28)	Ru(1)	70.17(11)
C(29)	C(28)	C(34)	123.06(19)
C(29)	C(28)	C(33)	118.2(2)
C(33)	C(28)	Ru(1)	69.64(12)
C(33)	C(28)	C(34)	118.73(18)
F(27)	C(13)	C(14)	118.8(2)
F(27)	C(13)	C(12)	118.0(2)
C(14)	C(13)	C(12)	123.2(2)
C(12)	C(11)	C(10)	119.6(2)
C(24)	C(25)	C(20)	119.8(2)
C(14)	C(15)	C(10)	119.0(2)
F(26)	C(23)	C(24)	118.4(2)
F(26)	C(23)	C(22)	118.5(2)
C(22)	C(23)	C(24)	123.1(2)
C(13)	C(12)	C(11)	118.3(2)
C(25)	C(24)	C(23)	118.6(2)
C(23)	C(22)	C(21)	118.0(2)

Table S.18: Torsion Angles in ° for **5**.

**Atom**	**Atom**	**Atom**	**Atom**	**Angle/°**
Ru(1)	N(19)	C(20)	C(21)	143.83(18)
Ru(1)	N(19)	C(20)	C(25)	-34.7(3)
Ru(1)	N(19)	C(18)	C(17)	-2.3(3)
Ru(1)	N(16)	C(10)	C(11)	48.8(3)
Ru(1)	N(16)	C(10)	C(15)	-131.31(19)
Ru(1)	N(16)	C(17)	C(18)	-0.4(3)
Ru(1)	C(32)	C(31)	C(30)	-53.93(16)
Ru(1)	C(32)	C(31)	C(37)	122.5(2)
Ru(1)	C(32)	C(33)	C(28)	55.11(18)
Ru(1)	C(29)	C(30)	C(31)	55.72(18)
Ru(1)	C(29)	C(28)	C(34)	127.18(19)
Ru(1)	C(29)	C(28)	C(33)	-52.18(17)
Ru(1)	C(31)	C(30)	C(29)	-56.04(18)
Ru(1)	C(33)	C(28)	C(34)	-126.96(18)
Ru(1)	C(33)	C(28)	C(29)	52.43(17)
F(26)	C(23)	C(24)	C(25)	-179.1(2)

F(26)	C(23)	C(22)	C(21)	179.1(2)
F(27)	C(13)	C(12)	C(11)	179.3(2)
N(19)	C(20)	C(21)	C(22)	-178.4(2)
N(19)	C(20)	C(25)	C(24)	178.5(2)
N(16)	C(10)	C(11)	C(12)	178.7(2)
N(16)	C(10)	C(15)	C(14)	-179.5(2)
N(16)	C(17)	C(18)	N(19)	1.8(3)
C(32)	C(31)	C(30)	Ru(1)	54.36(16)
C(32)	C(31)	C(30)	C(29)	-1.7(3)
C(32)	C(33)	C(28)	Ru(1)	-53.92(18)
C(32)	C(33)	C(28)	C(34)	179.12(19)
C(32)	C(33)	C(28)	C(29)	-1.5(3)
C(31)	C(32)	C(33)	Ru(1)	-54.08(18)
C(31)	C(32)	C(33)	C(28)	1.0(3)
C(10)	N(16)	C(17)	C(18)	179.7(2)
C(10)	C(11)	C(12)	C(13)	0.8(4)
C(33)	C(32)	C(31)	Ru(1)	54.47(18)
C(33)	C(32)	C(31)	C(30)	0.5(3)
C(33)	C(32)	C(31)	C(37)	176.9(2)
C(20)	N(19)	C(18)	C(17)	179.5(2)
C(20)	C(21)	C(22)	C(23)	0.5(4)
C(20)	C(25)	C(24)	C(23)	-0.5(4)
C(21)	C(20)	C(25)	C(24)	-0.1(4)
C(17)	N(16)	C(10)	C(11)	-131.2(2)
C(17)	N(16)	C(10)	C(15)	48.7(3)
C(14)	C(13)	C(12)	C(11)	0.4(4)
C(30)	C(29)	C(28)	Ru(1)	52.55(17)
C(30)	C(29)	C(28)	C(34)	179.73(19)
C(30)	C(29)	C(28)	C(33)	0.4(3)
C(18)	N(19)	C(20)	C(21)	-38.1(3)
C(18)	N(19)	C(20)	C(25)	143.3(2)
C(28)	C(29)	C(30)	Ru(1)	-54.48(17)
C(28)	C(29)	C(30)	C(31)	1.2(3)
C(13)	C(14)	C(15)	C(10)	0.8(4)
C(11)	C(10)	C(15)	C(14)	0.3(3)
C(25)	C(20)	C(21)	C(22)	0.1(4)
C(15)	C(10)	C(11)	C(12)	-1.1(4)
C(15)	C(14)	C(13)	F(27)	179.9(2)
C(15)	C(14)	C(13)	C(12)	-1.2(4)
C(37)	C(31)	C(30)	Ru(1)	-122.0(2)
C(37)	C(31)	C(30)	C(29)	-178.1(2)
C(36)	C(34)	C(28)	Ru(1)	71.8(2)
C(36)	C(34)	C(28)	C(29)	-20.1(3)
C(36)	C(34)	C(28)	C(33)	159.21(19)
C(24)	C(23)	C(22)	C(21)	-1.1(4)
C(22)	C(23)	C(24)	C(25)	1.1(4)
C(35)	C(34)	C(28)	Ru(1)	-164.65(17)

C(35)	C(34)	C(28)	C(29)	103.4(2)
C(35)	C(34)	C(28)	C(33)	-77.3(2)

Table S.19: Hydrogen Fractional Atomic Coordinates (×10[4]) and Equivalent Isotropic Displacement Parameters (Å[2]×10[3]) for **5**. U_{eq} is defined as 1/3 of the trace of the orthogonalized U_{ij}.

**Atom**	**x**	**y**	**z**	***U_{eq}***
H(32)	2192.96	5564.61	4735.22	25
H(34)	871.38	4757.3	1122.92	27
H(29)	4402.47	5251.6	2167.33	23
H(33)	1080.88	4971.51	3079.54	25
H(21)	1231.45	7604.19	4857.07	29
H(17)	3391.91	7922.28	2490.62	30
H(14)	7501.91	7565.55	2217.47	33
H(30)	5488.52	5879.61	3810.49	24
H(18)	1597.14	7869.3	3287.92	30
H(11)	3472.8	6293.04	695.29	31
H(25)	-309.09	6017.13	2854.6	31
H(15)	5830.36	7591.82	3042.56	30
H(37A)	5326.11	6499.81	5379.26	40
H(37B)	5153.87	5912.93	6146.28	40
H(37C)	3958.26	6452.46	5661.14	40
H(36A)	2268.32	5213.92	227.35	41
H(36B)	1832.62	4478.64	-204.04	41
H(36C)	3357.37	4626.62	690.19	41
H(12)	5123.57	6290.9	-145.86	35
H(24)	-1956.37	5874.58	3666.99	36
H(22)	-422.38	7459.36	5670.94	32
H(35A)	2987.79	3781.25	2020.44	45
H(35B)	1476.84	3639.58	1098.64	45
H(35C)	1655.38	3848.61	2346.88	45

Citations

- Bourhis, L. J., Dolomanov, O. V., Gildea, R. J., Howard, J. A. K. & Puschmann, H. (2015). The anatomy of a comprehensive constrained, restrained refinement program for the modern computing environment - Olex2 dissected. Acta Cryst. A71, 59 – 75.
- Sheldrick, G.M. (2015) Crystal Structure Refinement with SHELXL. Acta Crystallographica C, C71, 3-8.
- O.V. Dolomanov and L.J. Bourhis and R.J. Gildea and J.A.K. Howard and H. Puschmann, Olex2: A complete structure solution, refinement and analysis program, *J. Appl. Cryst.*, (2009), **42**, 339-341.
- Oxford Diffraction, CrysAlis PRO, Oxford Diffraction Ltd, Yarnton, England, 2025.

Crystal Data and Experimental for **6.CH_2Cl_2.**

Experimental. Single crystals of **6.CH_2Cl_2** were obtained by recrystallisation from slow diffusion of a dichloromethane-hexamethyldisiloxane solution at low temperature (- 8 °C). A suitable crystal was selected and mounted on a suitable support on an XtaLAB Synergy-S Dualflex, HyPix diffractometer. The crystal was kept at a steady T = 100.00(16) K during data collection. The structure was solved with the ShelXT (Sheldrick, 2015) structure solution program using the dual solution method and by using **Olex2** (Dolomanov et al., 2009) as the graphical interface. The model was refined with version 2019/2 of ShelXL 2019/2 (Sheldrick, 2015) using Least Squares minimization. The structure was deposited in the Cambridge Structural Database, with CCDC number 2450157 for **6.CH_2Cl**.

Crystal Data. $C_{49}H_{48}Cl_8F_{12}N_4P_2Ru_2$, M_r = 1468.59, monoclinic, $I2/a$ (No. 15), a = 16.6760(2) Å, b = 15.4841(2) Å, c = 22.2792(3) Å, β = 99.9480(10)°, V = 5666.28(13) Å^3, T = 100.00(16) K, Z = 4, Z' = 0.5, μ(Cu K_α) = 9.021, 28751 reflections measured, 6058 unique (R_{int} = 0.0434) which were used in all calculations. The final wR_2 was 0.0767 (all data) and R_1 was 0.0289 (I >σ2(I)).

Experimental Extended. A crystal with was mounted on a suitable support. Data were collected using an XtaLAB Synergy, Dualflex, HyPix diffractometer operating at T = 100.00(16) K. Data were measured using w scans of 0.5° per frame for 0.7/0.2 s using Cu K_a radiation. The diffraction pattern was indexed, and the total number of runs and images was based on the strategy calculation from the program CrysAlisPro (Rigaku, V1.171.44.91a, 2025) The maximum resolution that was achieved was Θ = 79.520° (0.78 Å).

The diffraction pattern was indexed and the total number of runs and images was based on the strategy calculation from the program CrysAlisPro (Rigaku, V1.171.44.91a, 2025) and the unit cell was refined using CrysAlisPro (Rigaku, V1.171.44.91a, 2025) on 19324 reflections, 67% of the observed reflections.

Data reduction, scaling and absorption corrections were performed using CrysAlisPro (Rigaku, V1.171.44.91a, 2025). The final completeness is 99.80 % out to 79.520° in Θ. A multi-scan absorption correction was performed using CrysAlisPro 1.171.44.91a (Rigaku Oxford Diffraction, 2025) using spherical harmonics, implemented in SCALE3 ABSPACK scaling algorithm. The absorption coefficient μ of this material is 9.021 mm^{-1} at this wavelength (λ = 1.542Å) and the minimum and maximum transmissions are 0.424 and 1.000.

The structure was solved and the space group *I*2/*a* (# 15) determined by the ShelXT (Sheldrick, 2015) structure solution program using dual and refined by Least Squares using version 2019/2 of ShelXL 2019/2 (Sheldrick, 2015). All non-hydrogen atoms were refined anisotropically. Hydrogen atom positions were calculated geometrically and refined using the riding model. Hydrogen atom positions were calculated geometrically and refined using the riding model.

Table S.20: Fractional Atomic Coordinates (×10^4) and Equivalent Isotropic Displacement Parameters (Å^2×10^3) for **6.CH$_2$Cl$_2$**. U_{eq} is defined as 1/3 of the trace of the orthogonalised U_{ij}.

**Atom**	**x**	**y**	**z**	***U**$_{eq}$*
Ru1	5403.2(2)	5963.3(2)	6462.1(2)	17.82(7)
Cl2	4232.8(4)	5905.3(4)	5683.7(3)	22.93(13)
Cl1	96824.1(5)	1648.3(5)	5737.3(4)	31.60(16)
Cl2	04705.4(6)	10596.5(5)	6211.0(4)	39.00(19)
Cl3	06968.9(10)	9459.7(9)	5449.2(7)	80.1(4)
N9	5732.5(14)	6974.4(15)	5940.9(11)	21.6(5)
N12	6011.8(14)	5360.7(15)	5838.9(10)	19.8(4)
C3	35377.3(18)	6722.9(19)	7301.2(12)	23.5(6)
C14	5632.1(17)	3849.3(19)	5900.9(12)	22.1(5)
C32	4637.5(17)	6278.2(19)	7160.3(12)	21.1(5)

C3	5504.5(18)	7860.6(18)	5998.3(13)	24.0(6)
C15	5812.6(18)	2980.7(19)	5874.9(13)	24.1(6)
C17	7182.7(17)	3347.0(19)	5730.2(13)	23.0(5)
C38	3816.8(18)	6722.2(19)	7095.9(13)	24.9(6)
C11	6204.3(17)	5849.1(18)	5410.3(13)	22.7(5)
C37	4670.8(18)	5364.5(18)	7076.9(12)	22.5(5)
C34	6136.4(18)	6285(2)	7351.4(13)	26.7(6)
C8	4883(2)	8211(2)	5574.0(14)	30.0(6)
C10	6025.3(17)	6755.3(18)	5458.2(13)	22.9(5)
C18	6996.3(17)	4214.4(19)	5755.9(13)	22.7(5)
C16	6591.5(19)	2741.2(19)	5785.5(13)	24.7(6)
C35	6161.8(18)	5383(2)	7281.3(13)	26.7(6)
C13	6220.1(17)	4465.3(17)	5833.1(12)	20.3(5)
C40	3538(2)	6682(2)	7713.9(15)	33.6(7)
C4	5896.0(19)	8351.8(19)	6483.9(14)	26.5(6)
C7	4639(2)	9061(2)	5632.8(16)	33.6(7)
C36	5414.5(19)	4924.0(19)	7146.5(13)	25.7(6)
C5	5656(2)	9199(2)	6545.4(15)	29.2(6)
C6	5027(2)	9539(2)	6122.4(15)	31.6(7)
C39	3807(2)	7640(2)	6856.5(17)	35.7(7)
C41	6963(2)	4910(3)	7356.7(16)	37.7(8)
C31	7500	8862(4)	5000	98(4)

Table S: 21: Anisotropic Displacement Parameters (×10^4) **6.CH_2Cl_2**. The anisotropic displacement factor exponent takes the form: $-2\pi^2[h^2a^{*2} \times U_{11} + ... + 2hka^* \times b^* \times U_{12}]$

**Atom**	***U_{11}***	***U_{22}***	***U_{33}***	***U_{23}***	***U_{13}***	***U_{12}***
Ru1	19.17(11)	17.57(11)	16.13(11)	-1.79(7)	1.40(7)	-0.23(7)
Cl2	20.5(3)	27.4(3)	19.6(3)	-2.7(2)	-0.2(2)	0.3(2)
Cl19	39.0(4)	22.3(3)	32.5(4)	-3.6(3)	3.4(3)	4.2(3)
Cl20	54.5(5)	22.4(3)	38.7(4)	-6.0(3)	3.9(4)	7.2(3)
Cl30	102.8(10)	71.4(8)	80.1(9)	20.9(7)	55.1(8)	36.2(7)
N9	24.7(11)	18.2(11)	21.4(11)	0.3(9)	2.6(9)	-3.0(9)
N12	17.8(10)	21.2(11)	19.8(11)	-2.5(9)	1.9(8)	0.1(9)
C33	28.9(14)	25.0(14)	16.3(12)	-7.0(10)	3.3(10)	-2.8(11)
C14	20.2(12)	28.3(14)	17.6(12)	0.2(10)	2.6(10)	-0.4(11)
C32	22.2(13)	27.2(14)	14.3(12)	-2.0(10)	4.1(10)	0.4(11)
C3	29.4(14)	18.4(13)	24.1(14)	0.1(10)	4.2(11)	-1.0(11)
C15	26.8(14)	22.7(13)	22.0(13)	-0.4(11)	2.3(11)	-2.7(11)
C17	21.6(13)	27.2(14)	19.7(12)	-4.0(11)	2.1(10)	3.9(11)
C38	24.9(14)	25.6(14)	23.4(14)	-3.5(11)	2.2(11)	1.3(11)
C11	23.0(13)	24.8(14)	20.5(13)	0.1(10)	4.6(10)	-0.6(11)
C37	26.3(14)	22.8(13)	18.6(12)	-0.2(10)	4.6(10)	-2.1(11)
C34	23.4(14)	37.1(16)	18.1(13)	-2.1(11)	-0.8(11)	-1.6(12)
C8	39.3(17)	24.1(14)	24.1(14)	-3.6(11)	-2.0(12)	0.3(13)
C10	24.0(13)	23.1(13)	21.9(13)	0.8(10)	4.7(11)	-1.5(11)

C18	22.1(13)	26.4(14)	19.0(13)	-0.7(10)	2.5(10)	-0.6(11)
C16	31.5(15)	22.8(13)	19.3(13)	-2.7(10)	2.5(11)	3.3(11)
C35	26.5(14)	35.7(16)	17.7(13)	3.8(11)	2.9(11)	5.7(12)
C13	22.1(13)	19.6(13)	18.9(12)	-2.7(10)	2.6(10)	0.6(10)
C40	31.7(16)	40.3(18)	30.7(16)	-1.2(13)	10.6(13)	6.4(14)
C4	29.9(15)	21.8(14)	26.4(14)	-1.1(11)	1.1(12)	-3.7(11)
C7	43.2(19)	25.5(15)	28.5(16)	-0.9(12)	-3.5(14)	4.3(13)
C36	36.7(16)	21.8(13)	19.4(13)	5.0(10)	7.3(12)	3.2(12)
C5	31.9(16)	24.6(14)	30.3(15)	-5.2(12)	2.8(12)	-4.8(12)
C6	43.5(18)	20.3(14)	31.3(16)	-1.8(12)	7.3(14)	1.9(13)
C39	32.5(16)	30.3(16)	46(2)	4.7(14)	11.2(14)	7.3(13)
C41	34.9(17)	50(2)	27.5(16)	7.3(14)	2.9(13)	14.3(15)
C31	164(10)	27(3)	133(8)	0	109(8)	0

Table S: 22: Bond Lengths in Å for **CH_2Cl_2**.

**Atom**	**Atom**	**Length/Å**
Ru1	Cl2	2.3781(7)
Ru1	N9	2.079(2)
Ru1	N12	2.077(2)
Ru1	C33	2.215(3)
Ru1	C32	2.230(3)
Ru1	C37	2.192(3)
Ru1	C34	2.198(3)
Ru1	C35	2.223(3)
Ru1	C36	2.215(3)
Cl19	C16	1.744(3)
Cl20	C6	1.745(3)
Cl30	C31	1.717(4)
N9	C3	1.435(4)
N9	C10	1.301(4)
N12	C11	1.301(4)
N12	C13	1.430(3)
C33	C32	1.400(4)
C33	C34	1.423(4)
C14	C15	1.382(4)
C14	C13	1.395(4)
C32	C38	1.516(4)
C32	C37	1.429(4)
C3	C8	1.387(4)
C3	C4	1.390(4)
C15	C16	1.398(4)
C17	C18	1.382(4)
C17	C16	1.382(4)
C38	C40	1.529(4)
C38	C39	1.516(4)
C11	C10	1.442(4)

C37	C36	1.400(4)
C34	C35	1.406(5)
C8	C7	1.391(4)
C18	C13	1.390(4)
C35	C36	1.421(4)
C35	C41	1.508(4)
C4	C5	1.386(4)
C7	C6	1.383(5)
C5	C6	1.386(5)

Table S.23: Bond Angles in ° for **6.CH$_2$Cl$_2$**.

**Atom**	**Atom**	**Atom**	**Angle/°**
N9	Ru1	Cl2	83.33(7)
N9	Ru1	C33	96.72(10)
N9	Ru1	C32	117.60(10)
N9	Ru1	C37	153.78(10)
N9	Ru1	C34	99.99(11)
N9	Ru1	C35	126.52(11)
N9	Ru1	C36	163.77(11)
N12	Ru1	Cl2	85.97(6)
N12	Ru1	N9	76.01(9)
N12	Ru1	C33	152.31(10)
N12	Ru1	C32	165.71(10)
N12	Ru1	C37	128.09(10)
N12	Ru1	C34	116.38(10)
N12	Ru1	C35	95.33(10)
N12	Ru1	C36	100.76(10)
C33	Ru1	Cl2	120.15(8)
C33	Ru1	C32	36.72(10)
C33	Ru1	C35	67.22(11)
C32	Ru1	Cl2	91.16(7)
C37	Ru1	Cl2	87.94(8)
C37	Ru1	C33	66.62(11)
C37	Ru1	C32	37.70(10)
C37	Ru1	C34	79.39(11)
C37	Ru1	C35	67.39(11)
C37	Ru1	C36	37.05(11)
C34	Ru1	Cl2	157.60(8)
C34	Ru1	C33	37.63(11)
C34	Ru1	C32	67.55(11)
C34	Ru1	C35	37.09(12)
C34	Ru1	C36	66.84(12)
C35	Ru1	Cl2	149.61(9)
C35	Ru1	C32	80.14(10)
C36	Ru1	Cl2	112.52(8)
C36	Ru1	C33	78.69(11)

C36	Ru1	C32	67.45(11)
C36	Ru1	C35	37.36(12)
C3	N9	Ru1	124.79(19)
C10	N9	Ru1	116.01(19)
C10	N9	C3	117.8(2)
C11	N12	Ru1	116.13(19)
C11	N12	C13	117.8(2)
C13	N12	Ru1	126.09(18)
C32	C33	Ru1	72.22(16)
C32	C33	C34	121.4(3)
C34	C33	Ru1	70.53(16)
C15	C14	C13	119.9(3)
C33	C32	Ru1	71.06(15)
C33	C32	C38	123.0(3)
C33	C32	C37	117.6(3)
C38	C32	Ru1	130.50(19)
C37	C32	Ru1	69.70(15)
C37	C32	C38	119.4(3)
C8	C3	N9	119.4(3)
C8	C3	C4	120.8(3)
C4	C3	N9	119.8(3)
C14	C15	C16	118.6(3)
C16	C17	C18	119.1(3)
C32	C38	C40	107.9(2)
C32	C38	C39	114.4(3)
C39	C38	C40	111.6(3)
N12	C11	C10	115.5(3)
C32	C37	Ru1	72.59(16)
C36	C37	Ru1	72.36(16)
C36	C37	C32	121.4(3)
C33	C34	Ru1	71.84(16)
C35	C34	Ru1	72.40(17)
C35	C34	C33	120.5(3)
C3	C8	C7	120.2(3)
N9	C10	C11	115.3(3)
C17	C18	C13	119.8(3)
C15	C16	Cl19	119.2(2)
C17	C16	Cl19	118.9(2)
C17	C16	C15	121.8(3)
C34	C35	Ru1	70.52(16)
C34	C35	C36	118.6(3)
C34	C35	C41	120.9(3)
C36	C35	Ru1	71.02(16)
C36	C35	C41	120.5(3)
C41	C35	Ru1	131.0(2)
C14	C13	N12	119.1(2)
C18	C13	N12	120.3(2)

C18	C13	C14	120.6(3)
C5	C4	C3	119.4(3)
C6	C7	C8	118.4(3)
C37	C36	Ru1	70.59(16)
C37	C36	C35	120.5(3)
C35	C36	Ru1	71.62(16)
C4	C5	C6	119.3(3)
C7	C6	Cl20	118.7(3)
C7	C6	C5	121.9(3)
C5	C6	Cl20	119.4(2)
Cl30[1]	C31	Cl30	114.8(4)

[1]3/2-x,+y,1-z

Table S.24: Torsion Angles in ° for **6.CH_2Cl_2**.

**Atom**	**Atom**	**Atom**	**Atom**	**Angle/°**
Ru1	N9	C3	C8	-102.7(3)
Ru1	N9	C3	C4	76.0(3)
Ru1	N9	C10	C11	-9.9(3)
Ru1	N12	C11	C10	4.8(3)
Ru1	N12	C13	C14	46.6(3)
Ru1	N12	C13	C18	-133.9(2)
Ru1	C33	C32	C38	-126.6(3)
Ru1	C33	C32	C37	53.6(2)
Ru1	C33	C34	C35	-55.8(2)
Ru1	C32	C38	C40	174.2(2)
Ru1	C32	C38	C39	-61.0(4)
Ru1	C32	C37	C36	55.7(2)
Ru1	C37	C36	C35	53.5(2)
Ru1	C34	C35	C36	-54.2(2)
Ru1	C34	C35	C41	126.8(3)
Ru1	C35	C36	C37	-53.0(2)
N9	C3	C8	C7	178.1(3)
N9	C3	C4	C5	-178.1(3)
N12	C11	C10	N9	3.4(4)
C33	C32	C38	C40	-93.2(3)
C33	C32	C38	C39	31.6(4)
C33	C32	C37	Ru1	-54.3(2)
C33	C32	C37	C36	1.4(4)
C33	C34	C35	Ru1	55.5(2)
C33	C34	C35	C36	1.3(4)
C33	C34	C35	C41	-177.7(3)
C14	C15	C16	Cl19	-178.8(2)
C14	C15	C16	C17	0.8(4)
C32	C33	C34	Ru1	53.5(2)
C32	C33	C34	C35	-2.2(4)

C32	C37	C36	Ru1	-55.8(2)
C32	C37	C36	C35	-2.3(4)
C3	N9	C10	C11	-176.9(2)
C3	C8	C7	C6	-0.2(5)
C3	C4	C5	C6	0.2(5)
C15	C14	C13	N12	177.7(3)
C15	C14	C13	C18	-1.8(4)
C17	C18	C13	N12	-177.8(3)
C17	C18	C13	C14	1.7(4)
C38	C32	C37	Ru1	125.9(2)
C38	C32	C37	C36	-178.4(3)
C11	N12	C13	C14	-132.0(3)
C11	N12	C13	C18	47.4(4)
C37	C32	C38	C40	86.6(3)
C37	C32	C38	C39	-148.6(3)
C34	C33	C32	Ru1	-52.7(2)
C34	C33	C32	C38	-179.3(3)
C34	C33	C32	C37	0.9(4)
C34	C35	C36	Ru1	53.9(2)
C34	C35	C36	C37	0.9(4)
C8	C3	C4	C5	0.5(5)
C8	C7	C6	Cl20	-178.2(3)
C8	C7	C6	C5	1.0(5)
C10	N9	C3	C8	63.2(4)
C10	N9	C3	C4	-118.1(3)
C18	C17	C16	Cl19	178.7(2)
C18	C17	C16	C15	-0.9(4)
C16	C17	C18	C13	-0.4(4)
C13	N12	C11	C10	-176.4(2)
C13	C14	C15	C16	0.6(4)
C4	C3	C8	C7	-0.5(5)
C4	C5	C6	Cl20	178.2(3)
C4	C5	C6	C7	-1.0(5)
C41	C35	C36	Ru1	-127.0(3)
C41	C35	C36	C37	180.0(3)

Table S.25: Hydrogen Fractional Atomic Coordinates ($\times10^4$) and Equivalent Isotropic Displacement Parameters ($Å^2\times10^3$) for **6.CH₂Cl₂**. U_{eq} is defined as 1/3 of the trace of the orthogonalized U_{ij}.

**Atom**	**x**	**y**	**z**	***U_eq***
H33	5372.37	7329.46	7364.46	28
H14	5108.04	4026.82	5964.87	26
H15	5415.68	2555.2	5916.92	29
H17	7710.54	3169.61	5675.23	28
H38	3421.84	6378.15	6800.92	30
H11	6446.57	5623.95	5087.44	27
H37	4177.82	5049.18	6971.9	27
H34	6629.68	6604.21	7433.08	32
H8	4623.94	7869.51	5242.27	36
H10	6114.99	7163.35	5158.29	27
H18	7397.54	4638.19	5720.84	27
H40A	3543.06	6080.7	7851.76	50
H40B	2984.32	6913.86	7674.7	50
H40C	3906.99	7025.68	8011.28	50
H4	6324.25	8109	6770.94	32
H7	4215.15	9307.93	5343.67	40
H36	5419.63	4313.76	7103.41	31
H5	5918.8	9543.64	6874.22	35
H39A	4156.84	8003.77	7152.33	54
H39B	3248.58	7863.05	6794.01	54
H39C	4007.95	7644.81	6468.49	54
H41A	6908.35	4403.45	7088.72	57
H41B	7119.26	4723.04	7781.16	57
H41C	7382.97	5294.7	7249.3	57
H31A	7887.86	8484.62	5266.32	118
H31B	7112.15	8484.59	4733.69	118

Table S.26: Atomic Occupancies for all atoms that are not fully occupied in **6.CH₂Cl₂**.

**Atom**	**Occupancy**
H31A	0.5
H31B	0.5

Table S.27: Solvent masking (Olex2) information for **6.CH_2Cl_2**.

No	x	y	z	V	e	Content
1	0.250	0.250	-0.158	425.0	287.4	4PF6
2	-0.250	0.750	-0.207	425.0	287.4	4PF6
3	0.172	0.556	0.871	8.5	0.0	?
4	0.172	0.944	0.371	8.5	0.0	?
5	0.328	0.556	0.129	8.5	0.0	?
6	0.328	0.944	0.629	8.5	0.0	?
7	0.672	0.056	0.371	8.5	0.0	?
8	0.672	0.444	0.871	8.5	0.0	?
9	0.828	0.056	0.629	8.5	0.0	?
10	0.828	0.444	0.129	8.5	0.0	?

Citations

- Bourhis, L. J., Dolomanov, O. V., Gildea, R. J., Howard, J. A. K. & Puschmann, H. (2015). The anatomy of a comprehensive constrained, restrained refinement program for the modern computing environment - Olex2 dissected. Acta Cryst. A71, 59 – 75.
- Sheldrick, G.M. (2015) Crystal Structure Refinement with SHELXL. Acta Crystallographica C, C71, 3-8.
- O.V. Dolomanov and L.J. Bourhis and R.J. Gildea and J.A.K. Howard and H. Puschmann, Olex2: A complete structure solution, refinement and analysis program, *J. Appl. Cryst.*, (2009), **42**, 339-341.
- Oxford Diffraction, CrysAlis PRO, Oxford Diffraction Ltd, Yarnton, England, 2025.

DFT calculation

Complex **1**

1-a **1-b**

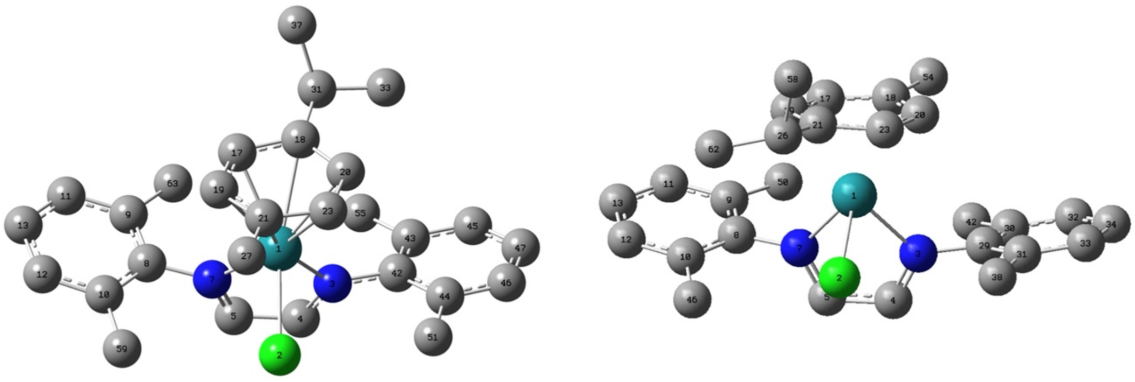

Fig.S.65: Atomic numbering for optimized structures **1-a** and **1-b** obtained by DFT calculation.

Table S.28: Bond length structural values for optimized structures **1-a** and **1-b** were calculated using DFT B3LYP/Lanl2dz (Ru) and 6-31G* (for all other atoms), with 6-31+G(d) applied to the *p*-cymene ligand in dichloromethane.

Bond	Structure **1-a** Length(Å)	Structure **1-b** Length (Å)
Ru-N (1)	2.0936	2.0761
N-C (2)	1.4459	1.4449
N=C (3)	1.3029	1.3022
C-C (4)	1.4445	1.4456
Ru-Cl (5)	2.4316	2.4347
Ru-C (6)	1.8008	1.8178

Table S.29: Percentual composition of the HOMO and LUMO orbitals for structures **1-a** and **1-b**.

Orbital	% Ru	% Cl	% *p*-cymene	% 2,6-dimetil
HOMO (**1-a**)	40.60	26.67	12.94	19.79
LUMO (**1-a**)	8.93	3.59	5.35	82.13
HOMO (**1-b**)	39.03	26.22	12.39	22.36
LUMO (**1-b**)	9.18	3.70	5.37	81.75

Structure **1-a** | Structure **1-b**

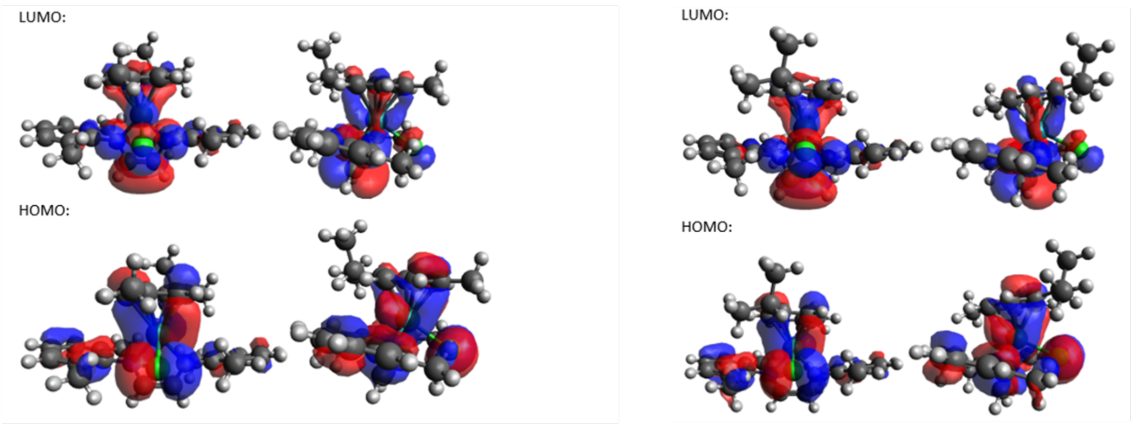

HOMO: -0.24617
LUMO : -0.13346
GAP HOMO-LUMO: 3.066 eV

HOMO: -0.24583
LUMO : -0.13407
GAP HOMO-LUMO: 3.041 eV

Relative stabilization energy +1.89 kcal mol^{-1}

B3LYP/LanI2dz (Ru), 6-31G* (other atoms) and 6-33+G(d) for *p*-cymene (in CH_2Cl_2).

Fig.S.66: Representation of HOMO and LUMO orbitals for optimized structures **1-a** and **1-b**.

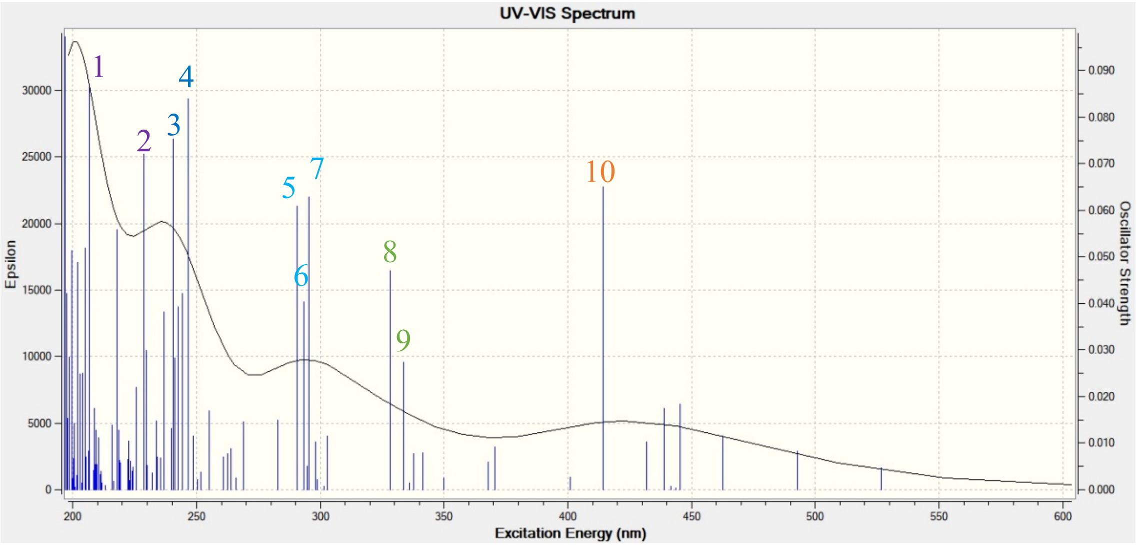

Fig.S67: UV/vis of optimized structure **1-a** calculated by DTDFT calculation.

Natural Transition Orbitals to describe the observed excited states for structure **1-a**.

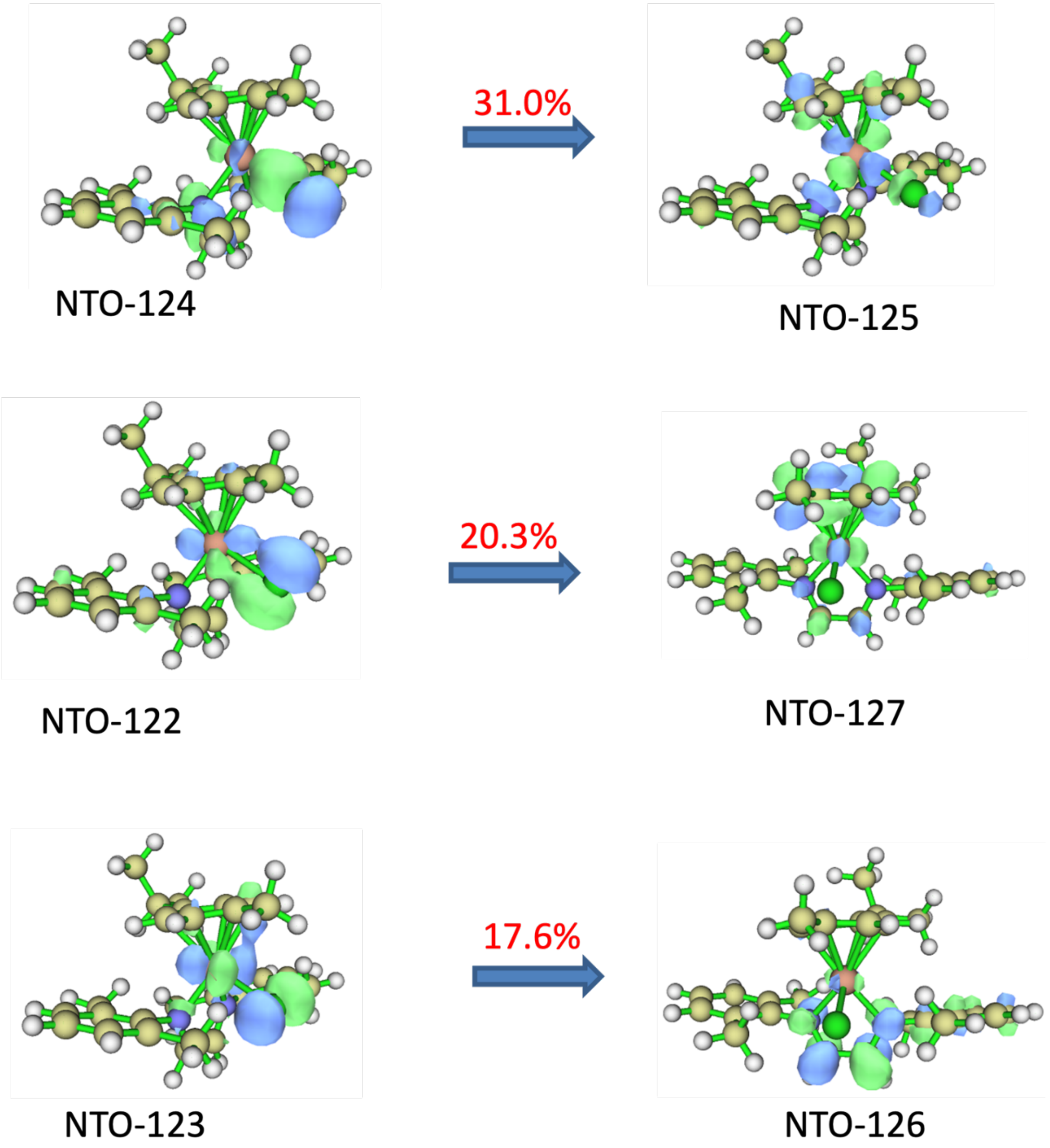

Scheme S.1: 1 Excited state 76 (λ = 206.49 nm, f = 0.0870).

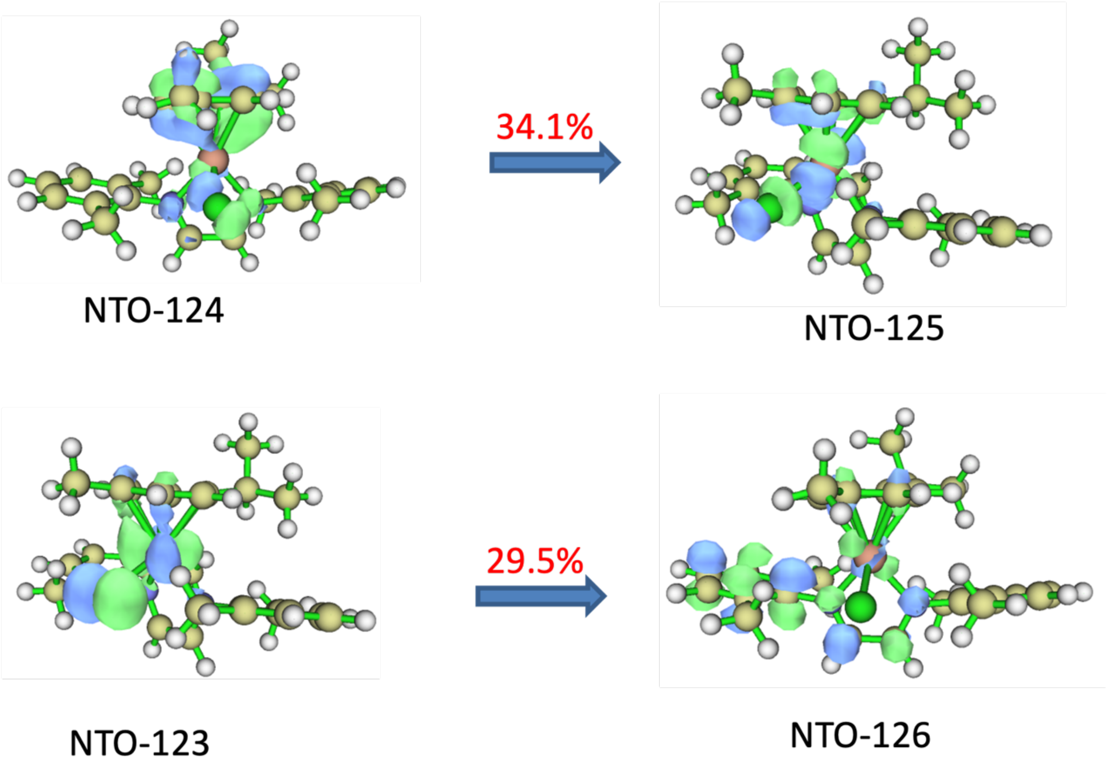

Scheme S.2: 2 Excited state 51 (λ = 228.81 nm, f = 0.0721).

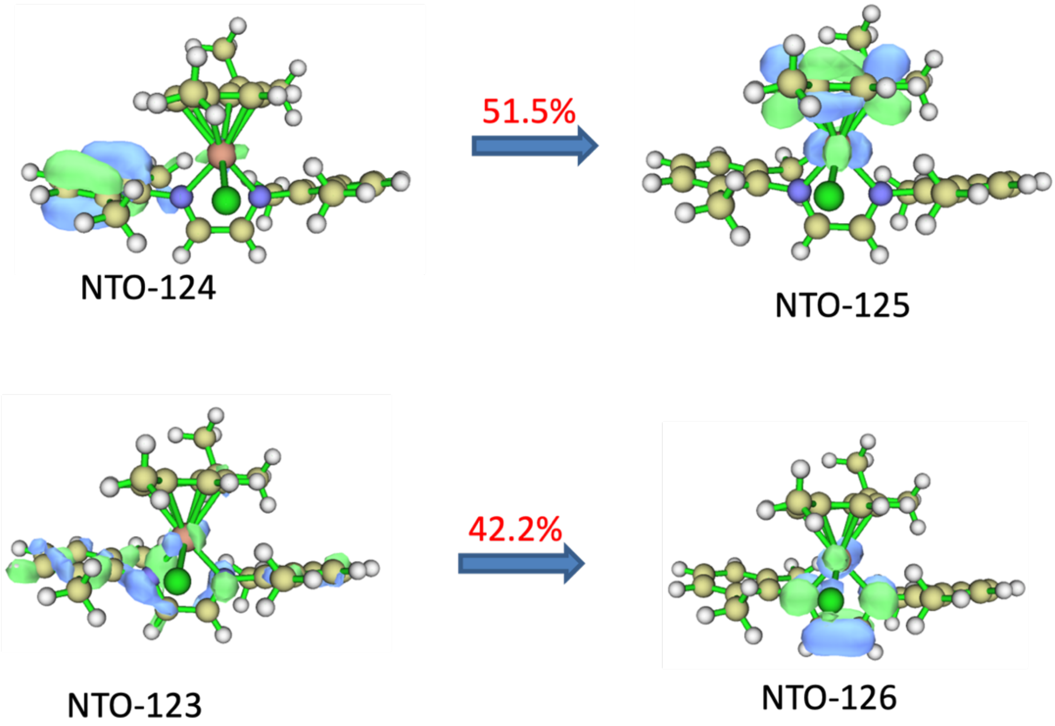

Scheme S.3: 3 Excited state 42 (λ = 240.51 nm, f = 0.0753).

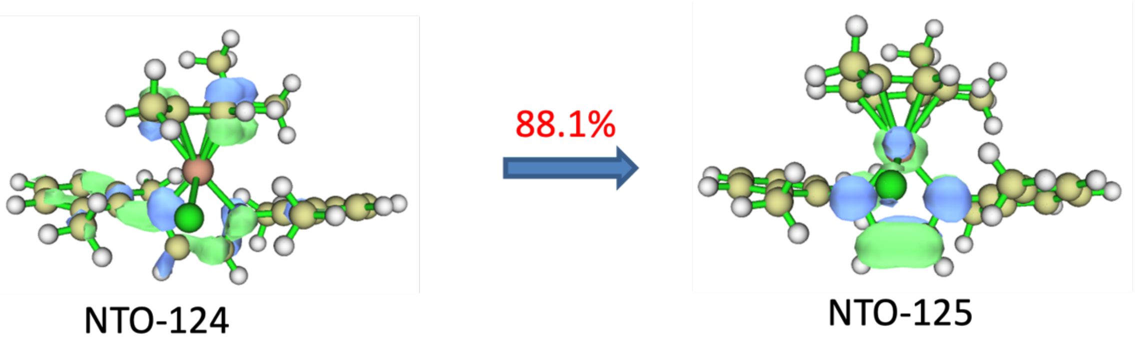

Scheme S.4: 4 Excited state 38 (λ = 246.71 nm, f = 0.0840).

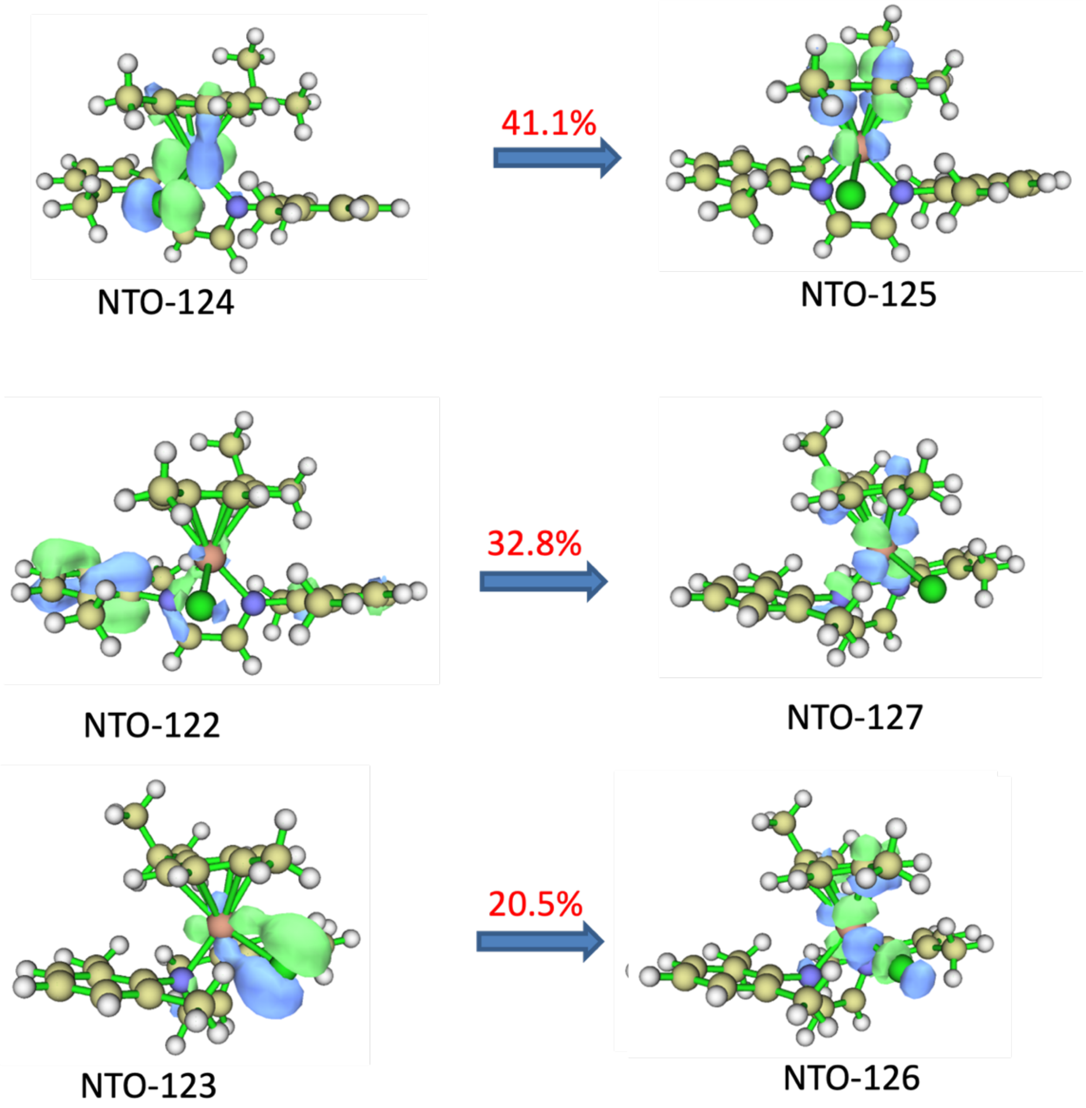

Scheme S.5: 5 Excited state 27 (λ = 290.76 nm f = 0.0609).

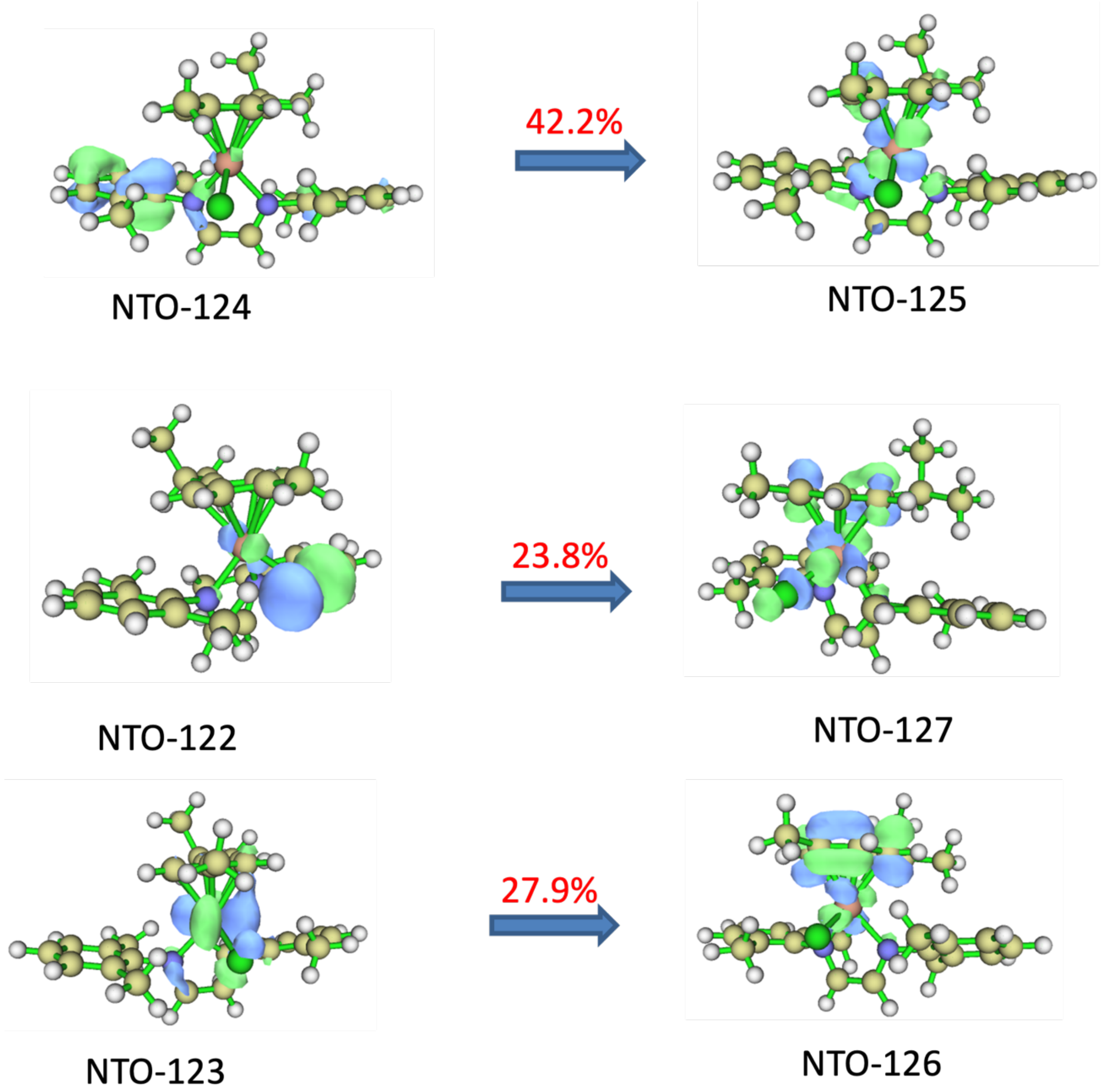

Scheme S.6: 6 Excited state 26 (λ = 293.26 nm, f = 0.0403).

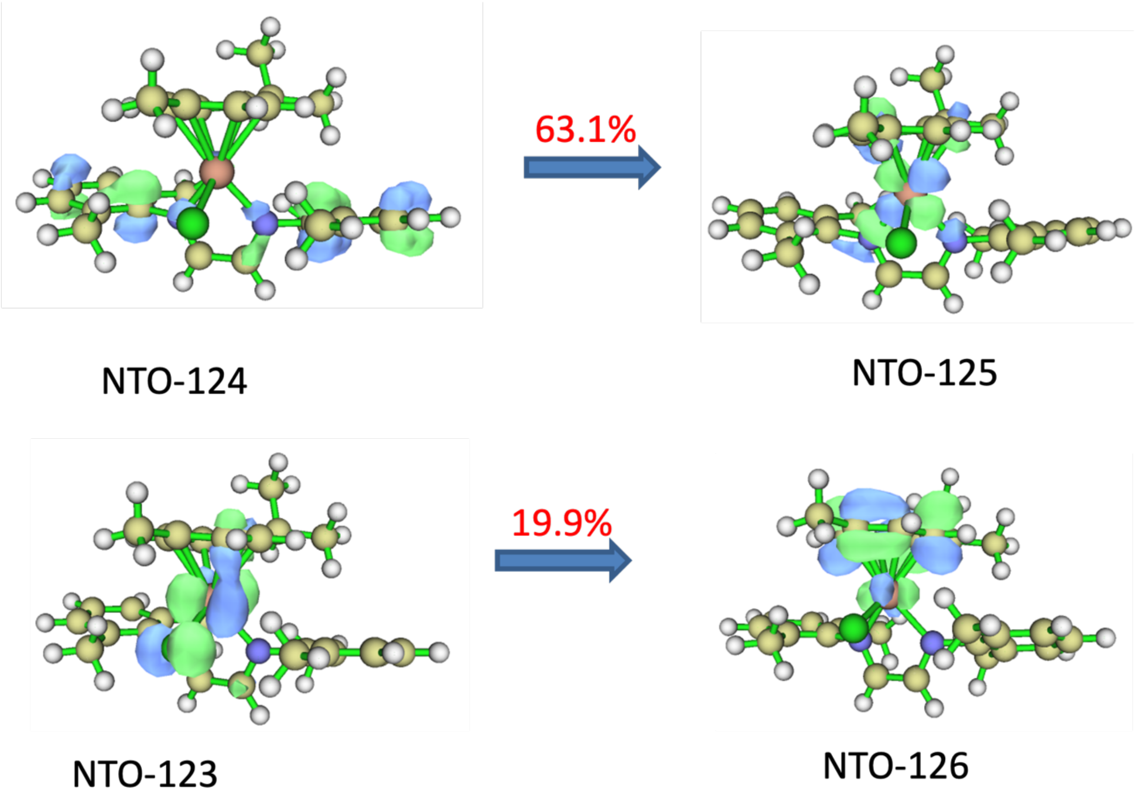

Scheme S.7: 7 Excited state 24 (λ = 295.40 nm f = 0.0628).

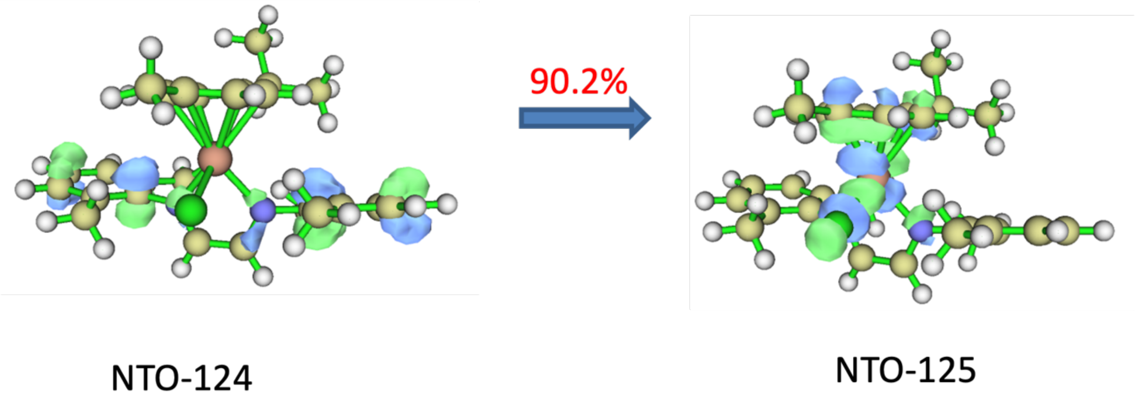

Scheme S.8: 8 Excited state19 (λ = 328.24 nm, f = 0.0470).

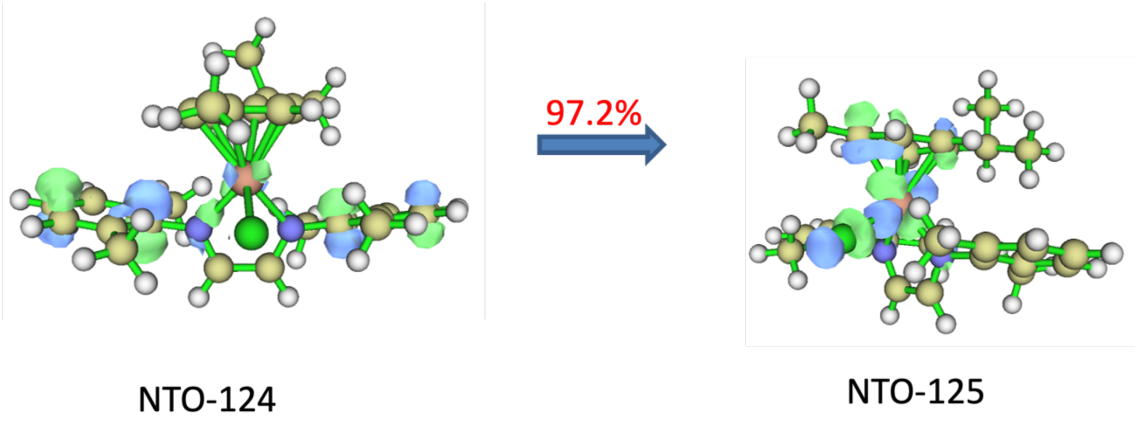

Scheme S.9: 9 Excited state 18 (λ = 333.66 nm, f = 0.0273).

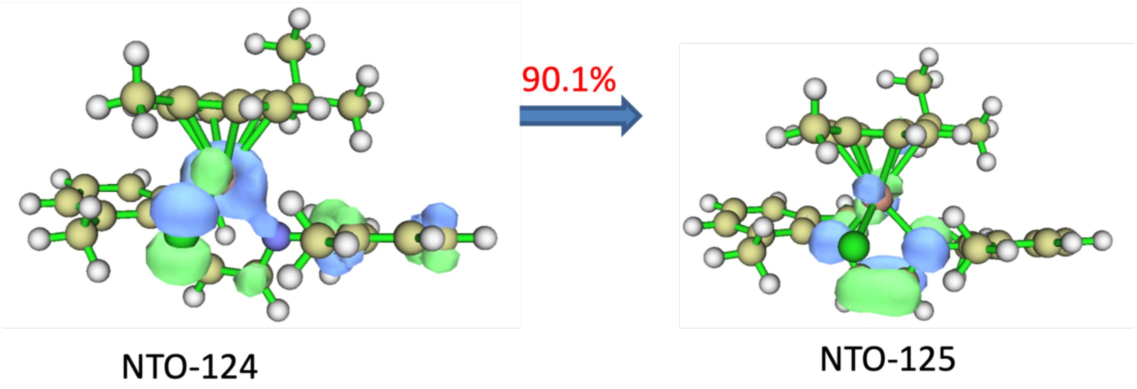

Scheme S.10: 10 Excited state 10 (λ = 414.08 nm, f = 0.0650).

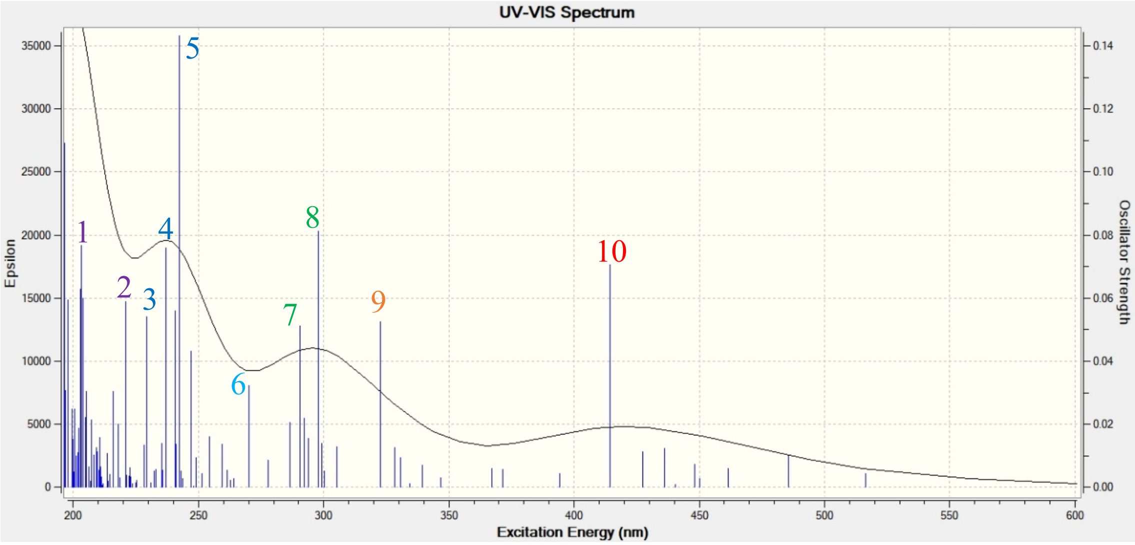

Fig.S.68: UV/vis of optimized structure **1-b** calculated by DTDFT calculation

Natural Transition Orbitals to describe the observed excited states for structure **1-b**.

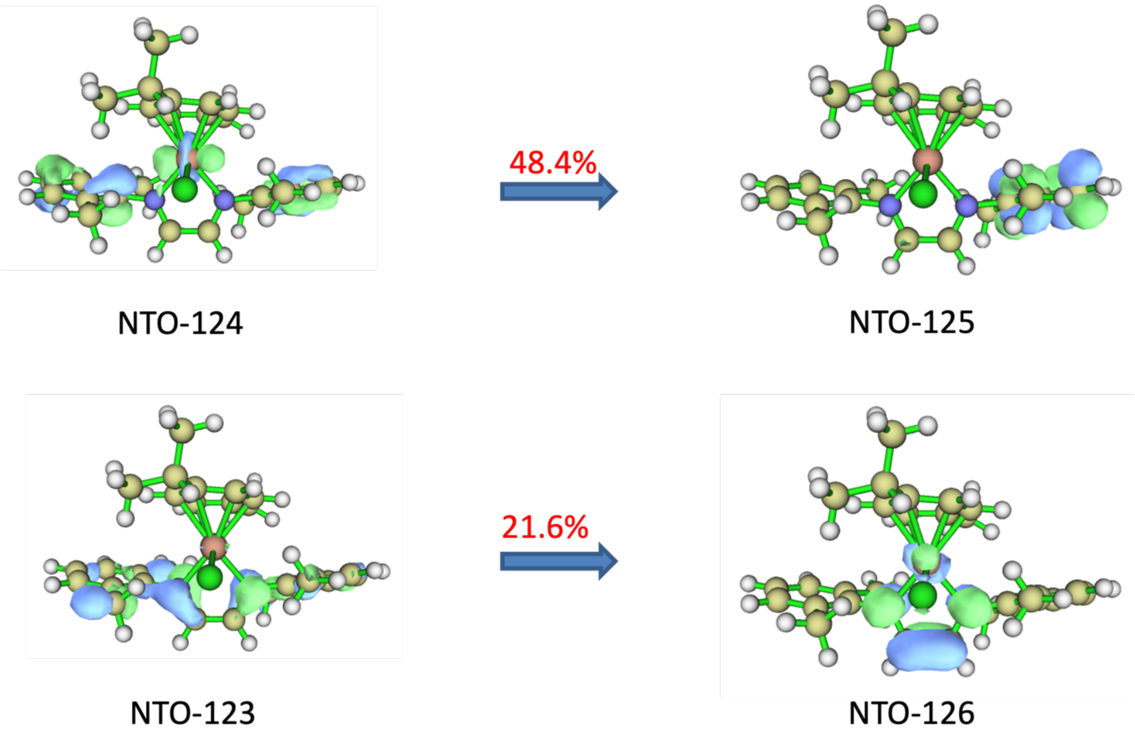

Scheme S. 11: 1 Excited state 82 (λ = 203.22 nm, f = 0.0767).

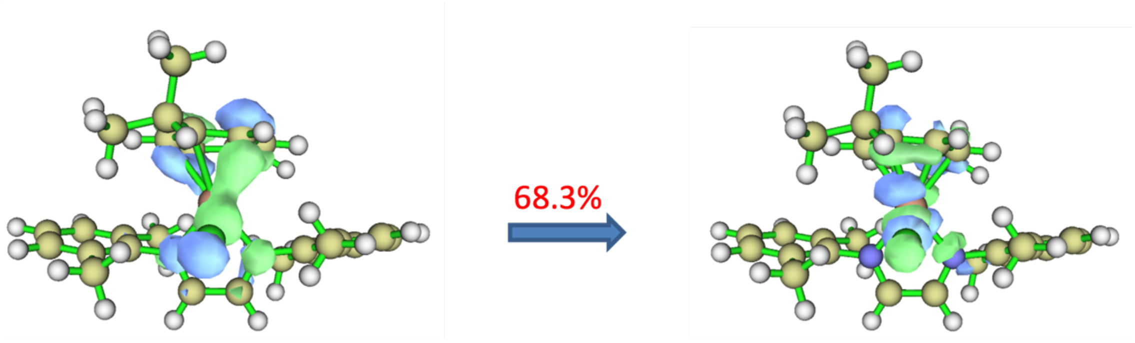

NTO-124 NTO-125

Scheme S.12: 2 Estado Excitado 59 (λ = 220.81 nm, f = 0.0590).

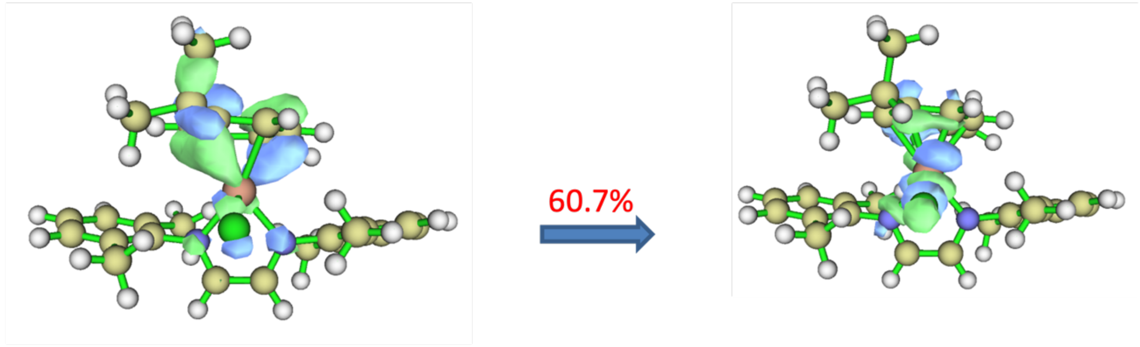

NTO-124 NTO-125

Scheme S. 13: 3 Excited state 50 (λ = 229.38 nm f = 0.0540).

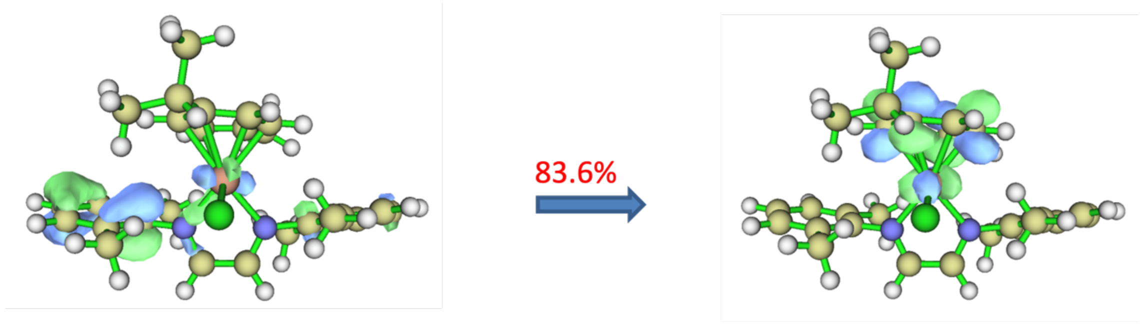

NTO-124 NTO-125

Scheme S.14: 4 Excited state 44 (λ = 237.08 nm, f = 0.0758).

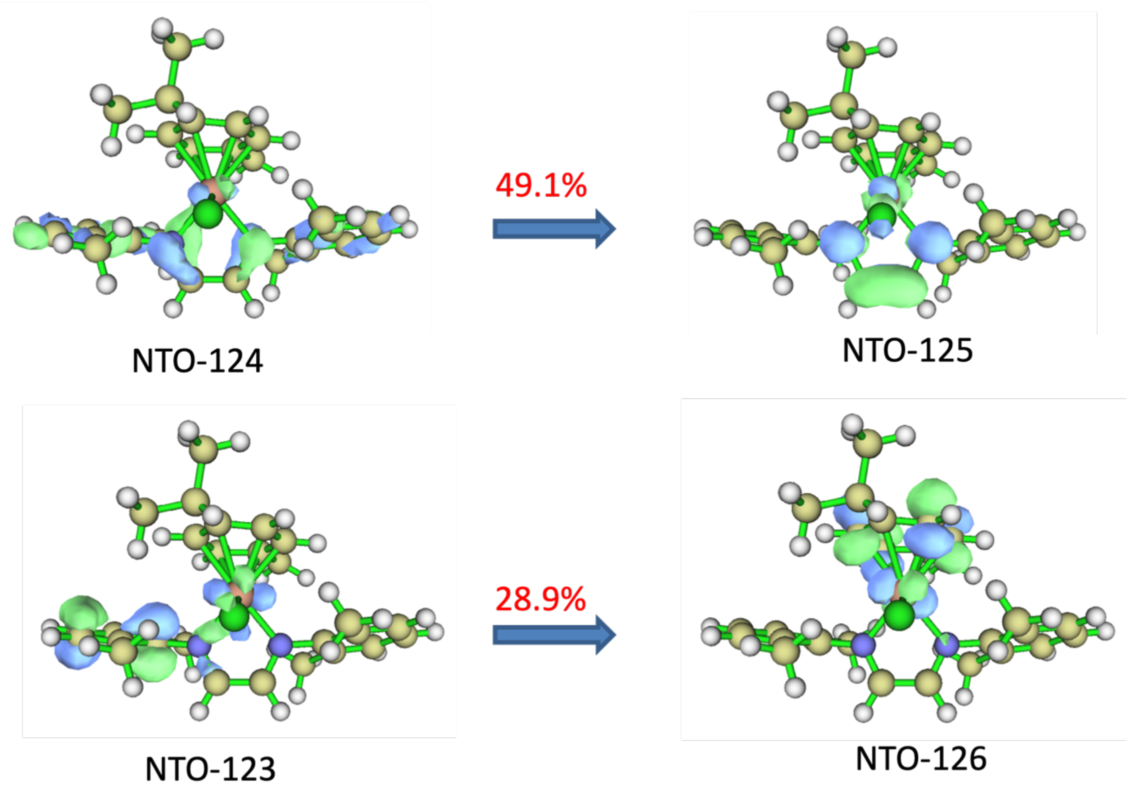

Scheme S.15: 5 Excited state 41 (λ = 242.29 nm, f = 0.1432).

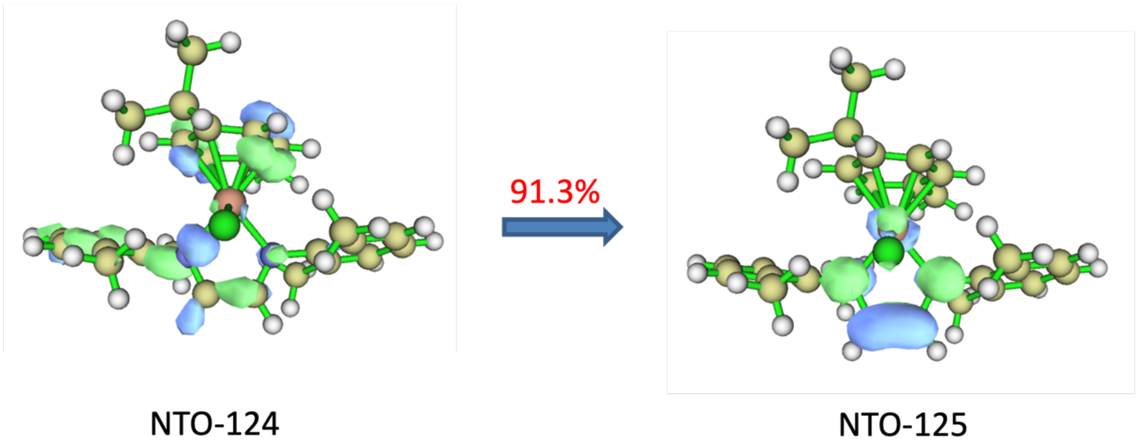

Scheme S.16: 6 Excited state 38 (λ = 247.11 nm, f = 0.0432).

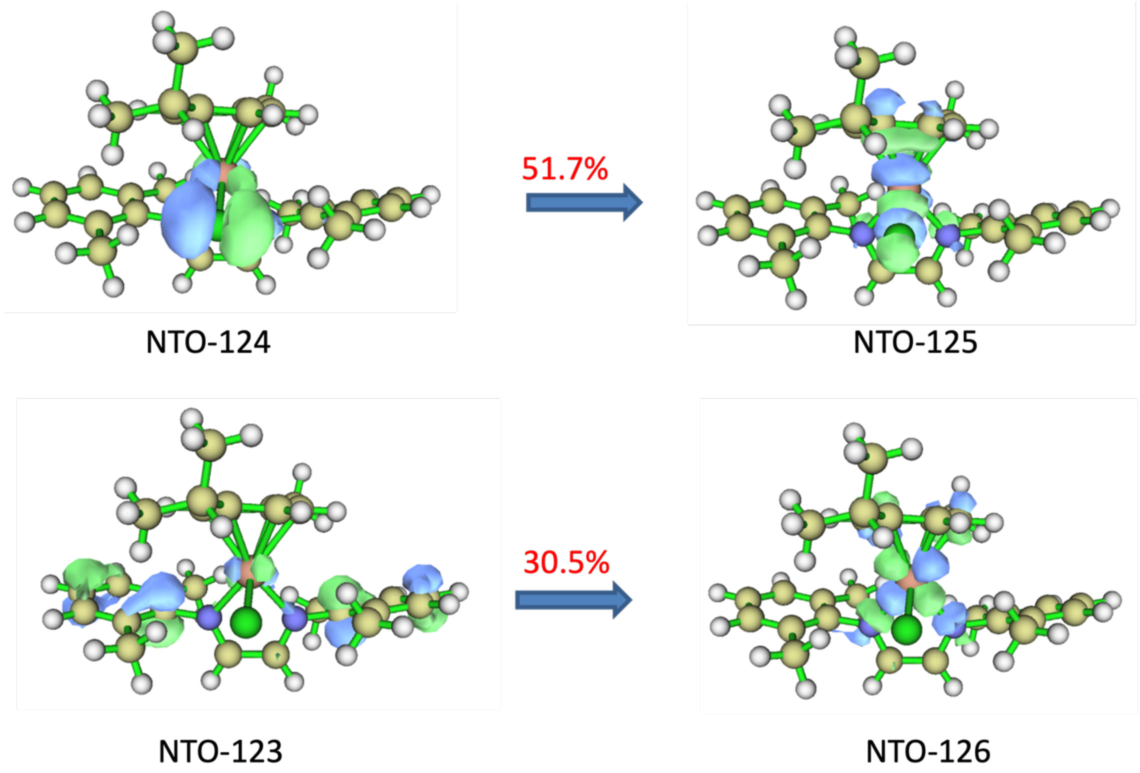

Scheme S.17: 7 Excited state 26 (λ = 290.42 nm f = 0.0511).

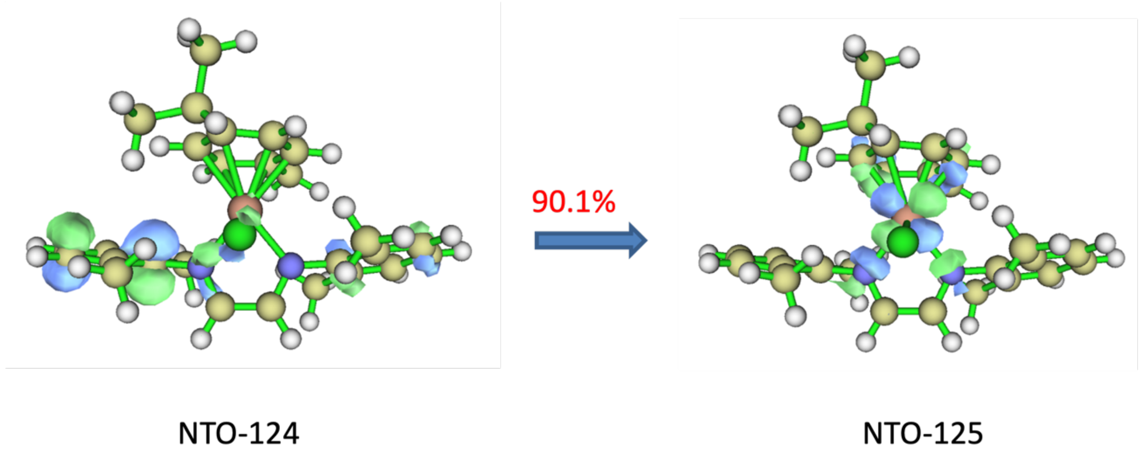

Scheme S.18: 8 Excited state 23 (λ = 297.82 nm, f = 0.0813).

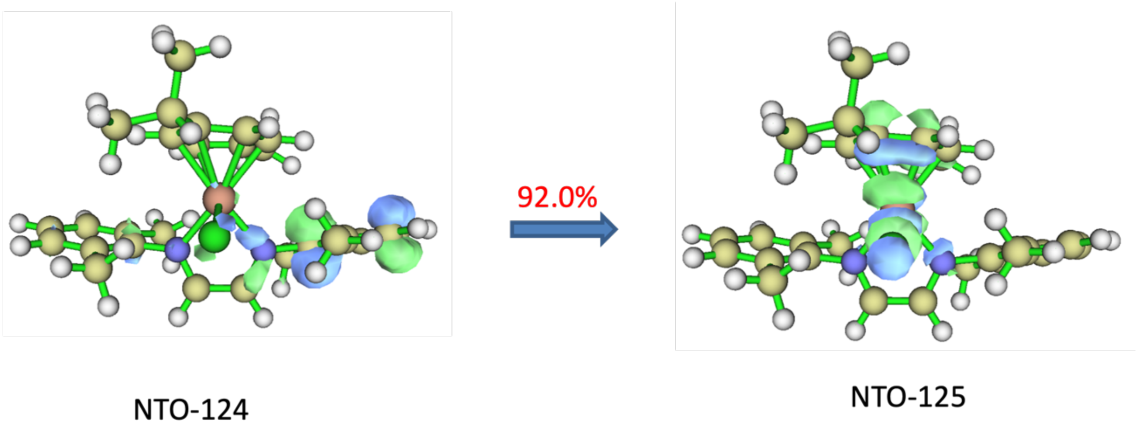

Scheme S.19: 9 Excited state 19 (λ = 322.61 nm, f = 0.0524).

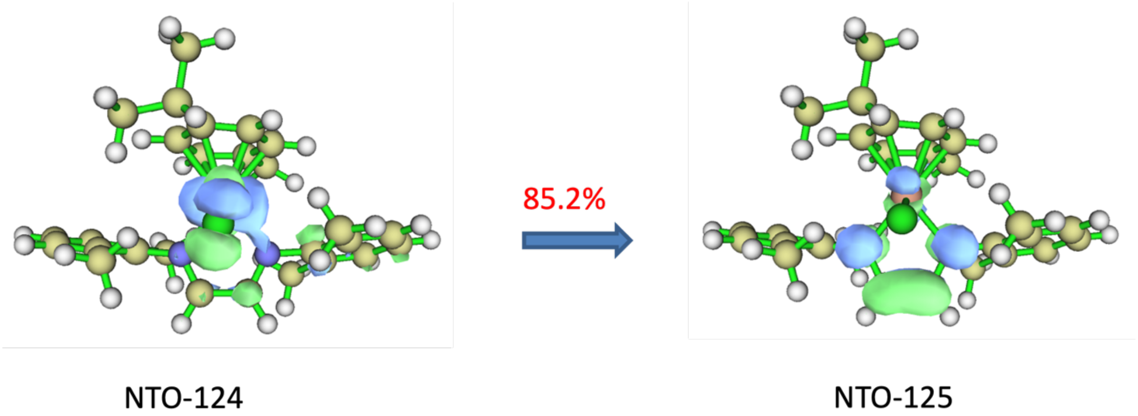

Scheme S.20: 10 Excited state 10 (λ = 414.48 nm, f = 0.0705)

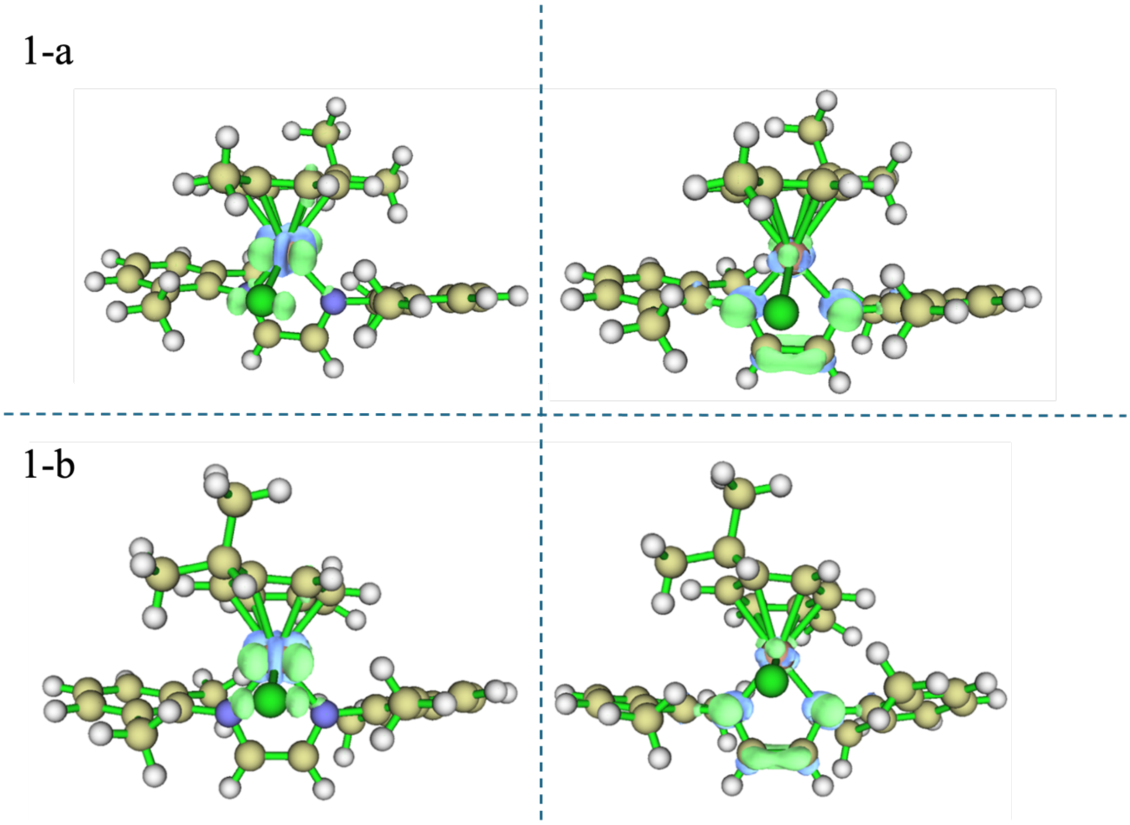

Fig.S.69: Fukui function for the optimized structures **1-a** and **1-b**, *f*- (left) represents an electrophilic attack region and *f*+ (right) represents a nucleophilic attack region.

Table S.30: Atomic dipole moment corrected Hirshfeld atomic charges , values of *f*+ and *f*- of structure **1-a**

		N (neutral)	N-1 (cation)	N+1 (anion)	*f*-	*f*+
1	Ru	0.356383	0.458478	0.335902	0.10210	0.020481
2	Cl	-0.32273	-0.16316	-0.39077	0.15957	0.068040
3	N	-0.0535	-0.03728	-0.12625	0.01622	0.072750
4	C	0.075329	0.112064	-0.0145	0.03674	0.089829
5	C	0.078849	0.113789	-0.01176	0.03494	0.090609
7	N	-0.05186	-0.03437	-0.12298	0.01749	0.071120
8	C	0.030114	0.031147	0.033837	0.00103	-0.003723
9	C	0.001185	0.007987	-0.00566	0.00680	0.006845
10	C	0.006399	0.015946	-0.0012	0.00955	0.007599
11	C	-0.04469	-0.02992	-0.05826	0.01477	0.013570
12	C	-0.0434	-0.0316	-0.05687	0.01180	0.013470
13	C	-0.03608	-0.01471	-0.05425	0.02137	0.018170

17	C	-0.01938	0.001348	-0.03494	0.02073	0.015560
18	C	0.025598	0.048494	0.007214	0.02290	0.018384
19	C	-0.0248	0.009597	-0.04653	0.03440	0.021730
20	C	-0.01008	0.012865	-0.02973	0.02295	0.019650
21	C	0.044916	0.067349	0.025955	0.02243	0.018961
23	C	-0.02511	0.018323	-0.04605	0.04343	0.020940
27	C	-0.06567	-0.05577	-0.0732	0.00990	0.007530
31	C	-0.00106	0.002737	-0.00347	0.00380	0.002410
33	C	-0.08179	-0.07812	-0.08465	0.00367	0.002860
37	C	-0.07648	-0.07001	-0.08161	0.00647	0.005130
42	C	0.032105	0.031757	0.034214	-0.00035	-0.002109
43	C	0.005456	0.014005	-0.00323	0.00855	0.008686
44	C	0.00811	0.013857	-0.00205	0.00575	0.010160
45	C	-0.04144	-0.02977	-0.05584	0.01167	0.014400
46	C	-0.04245	-0.03034	-0.05713	0.01211	0.014680
47	C	-0.03288	-0.01274	-0.05485	0.02014	0.021970
51	C	-0.08742	-0.08279	-0.09184	0.00463	0.004420
55	C	-0.08158	-0.07793	-0.08542	0.00365	0.003840
59	C	-0.08755	-0.0822	-0.09118	0.00535	0.003630
63	C	-0.0816	-0.07786	-0.08489	0.00374	0.003290

Table S.: 31: Atomic dipole moment corrected Hirshfeld atomic charges , values of *f*+ and *f*- of structure **1-b**

		N (neutral)	N-1 (cation)	N+1 (anion)	*f*-	*f*+
1	Ru	0.356543	0.457723	0.336143	0.101180	0.020400
2	Cl	-0.32318	-0.1619	-0.392686	0.161280	0.069506
3	N	-0.05188	-0.03678	-0.123278	0.015100	0.071398
4	C	0.076539	0.112915	-0.014727	0.036376	0.091266
5	C	0.080365	0.115396	-0.011299	0.035031	0.091664
7	N	-0.05153	-0.03493	-0.123194	0.016600	0.071664
8	C	0.029823	0.032269	0.033202	0.002446	-0.00338
9	C	0.000969	0.007462	-0.005884	0.006493	0.006853
10	C	0.006067	0.018276	-0.001670	0.012209	0.007737
11	C	-0.04516	-0.02769	-0.058624	0.017470	0.013464
12	C	-0.04379	-0.03121	-0.057100	0.012580	0.01331

13	C	-0.03656	-0.01526	-0.054446	0.021300	0.017886
17	C	-0.01962	0.004049	-0.037289	0.023669	0.017669
18	C	0.025831	0.047138	0.009138	0.021307	0.016693
19	C	-0.02047	0.01133	-0.042420	0.031800	0.02195
20	C	-0.01182	0.012741	-0.033047	0.024561	0.021227
21	C	0.037444	0.061327	0.020115	0.023883	0.017329
23	C	-0.01934	0.016991	-0.040136	0.036331	0.020796
26	C	-0.003	0.000452	-0.005583	0.003452	0.002583
29	C	0.031083	0.029755	0.033976	-0.001328	-0.00289
30	C	0.004637	0.011811	-0.003589	0.007174	0.008226
31	C	0.007762	0.013402	-0.001591	0.005640	0.009353
32	C	-0.04247	-0.03056	-0.057224	0.011910	0.014754
33	C	-0.0418	-0.03034	-0.056575	0.011460	0.014775
34	C	-0.03305	-0.01366	-0.054584	0.019390	0.021534
38	C	-0.08681	-0.08263	-0.091048	0.004180	0.004238
42	C	-0.0821	-0.0788	-0.085761	0.003300	0.003661
46	C	-0.08924	-0.08125	-0.093104	0.007990	0.003864
50	C	-0.08172	-0.07769	-0.084989	0.004030	0.003269
54	C	-0.06542	-0.05634	-0.071789	0.009080	0.006369
58	C	-0.07599	-0.06827	-0.081185	0.007720	0.005195
62	C	-0.07971	-0.07585	-0.082602	0.003860	0.002892

Complex 2

2-a **2-b**

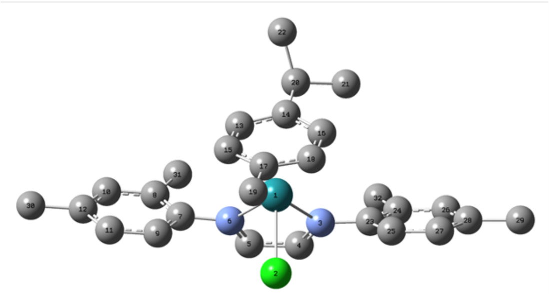

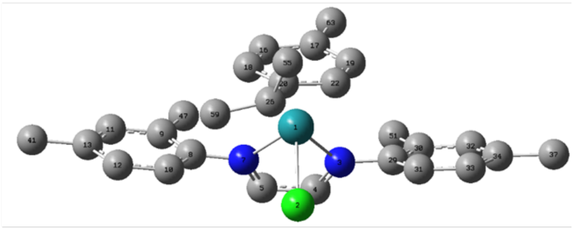

Fig.S.70: Representative atomic numbering for optimized structures **2-2b** obtained by DFT calculation.

Table S.32: Bond length structural values for optimized structures **2-1a**, **2-2a**, **2-3a** and **2-4a** were calculated using DFT B3LYP/Lanl2dz (Ru) and 6-31G* (for all other atoms), with 6-31+G(d) applied to the *p*-cymene ligand in dichloromethane.

Bond	Structure **2-1a** (Å)	Structure **2-2a** (Å)	Structure **2-3a** (Å)	Structure **2-4a** (Å)
Ru-N (1)	2.0865	2.0906	2.0839	2.0962
N-C (2)	1.4381	1.4273	1.4379	1.4273
N=C (3)	1.3007	1.3041	1.3013	1.3029
C-C (4)	1.4416	1.4367	1.4399	1.4390
Ru-Cl (5)	2.4380	2.4389	2.4440	2.4379
Ru-C (6)	1.8135	1.7967	1.8896	1.7897

Table S.33: Bond length structural values for optimized structures **2-1b**, **2-2b**, **2-3b** and **2-4b** were calculated using DFT B3LYP/Lanl2dz (Ru) and 6-31G* (for all other atoms), with 6-31+G(d) applied to the *p*-cymene ligand in dichloromethane.

Bond	Structure **2-1b** (Å)	Structure **2-2b** (Å)	Structure **2-3b** (Å)	Structure **2-4b** (Å)
Ru-N (1)	2.0837	2.0746	2.0838	2.0723
N-C (2)	1.4371	1.4291	1.4365	1.4307
N=C (3)	1.3031	1.3028	1.3036	1.3025
C-C (4)	1.4388	1.4380	1.4398	1.4379
Ru-Cl (5)	2.4442	2.4411	2.4409	2.4475
Ru-C (6)	1.8047	1.8031	1.8062	1.8189

Table S.34:Percentual composition of the HOMO and LUMO orbitals for all optimized structures of complex **2**.

Orbital	% Ru	% Cl	% *p*-cymene	% **N-N**^{2}
HOMO **2-1a**	36.82	21.71	13.02	28.45
LUMO **2-1a**	8.14	3.25	4.75	83.86
HOMO **2-1b**	44.58	24.96	13.00	17.46
LUMO **2-1b**	7.26	2.94	4.34	85.46
HOMO **2-2a**	29.52	12.25	12.10	46.13
LUMO **2-2a**	7.71	2.51	3.64	86.14
HOMO **2-2b**	31.21	13.01	12.91	42.87
LUMO **2-2b**	8.01	2.65	3.79	85.55
HOMO **2-3a**	33.77	16.69	12.36	37.18
LUMO **2-3a**	7.98	2.87	4.09	85.06
HOMO **2-3b**	34.30	16.79	13.66	35.25
LUMO **2-3b**	8.57	3.12	4.19	84.12
HOMO **2-4a**	30.85	16.06	12.65	40.44
LUMO **2-4a**	7.78	2.85	4.20	85.17
HOMO **2-4b**	41.65	20.06	12.96	25.33
LUMO **2-4b**	7.47	2.77	4.17	85.59

Structure **2-1a**

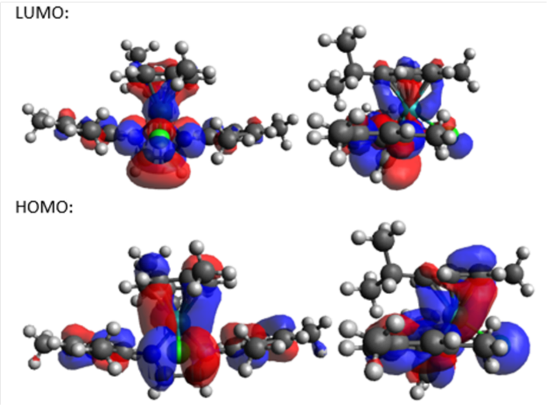

HOMO: -0.24255
LUMO : -0.13403 } GAP HOMO-LUMO: 2.952 eV

Relative stabilization energy +4.85 kcal mol^{-1}

Structure **2-2a**

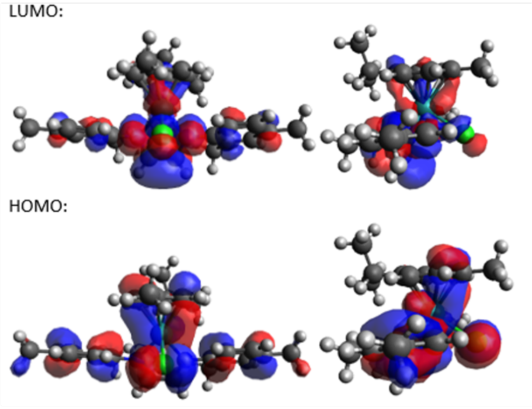

HOMO: -0.24031
LUMO : -0.13273 } GAP HOMO-LUMO: 2.927eV

Relative stabilization energy +1.05 kcal mol^{-1}

Structure **2-3a**

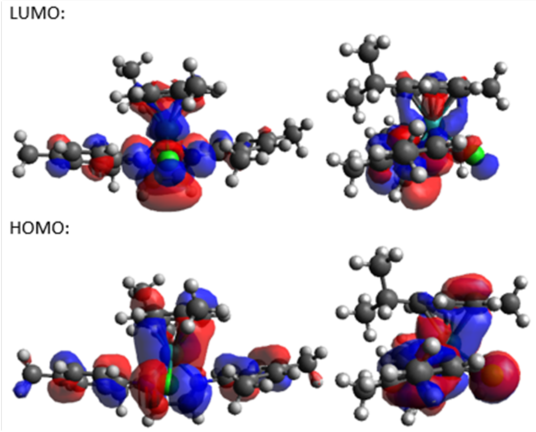

HOMO: -0.24088
LUMO : -0.13301 } GAP HOMO-LUMO: 2.935 eV

Relative stabilization energy +1.69 kcal mol^{-1}

Structure **2-4a**

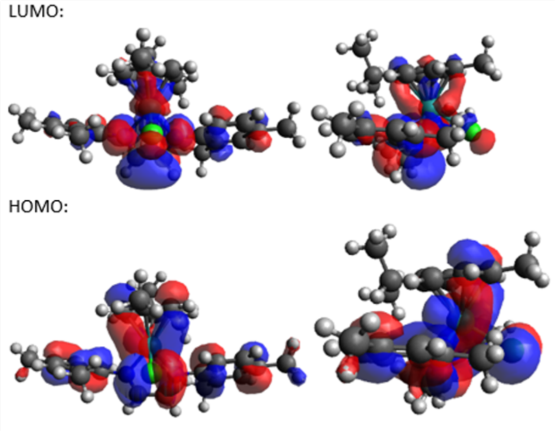

HOMO: -0.24175
LUMO : -0.13337 } GAP HOMO-LUMO: 2.949 eV

Relative stabilization energy +3.27 kcal mol^{-1}

B3LYP/LanI2dz (Ru), 6-31G* (other atoms) and 6-33+G(d) for *p*-cymene (in CH_2Cl_2).

Fig.S71: Representation of HOMO and LUMO orbitals for optimized structures **2-1a, 2-2a, 2-3a and 2-4a**.

Structure **2-1b**

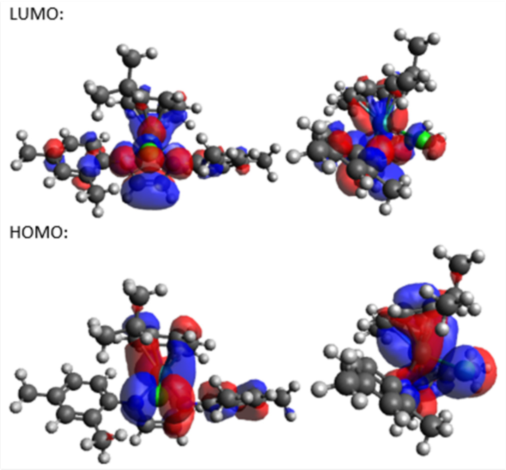

HOMO: -0.24588
LUMO : -0.13359

GAP HOMO-LUMO: 3.055 eV

Relative stabilization energy +4.18 kcal mol^{-1}

Structure **2-2b**

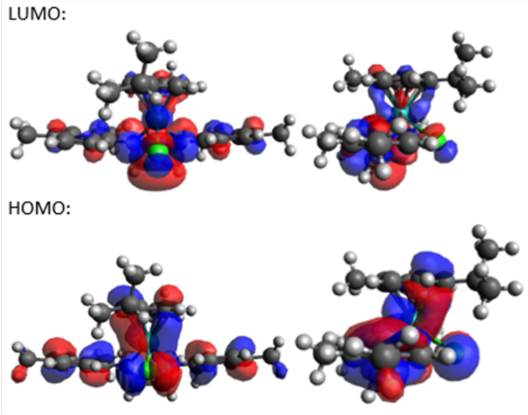

HOMO: -0.24019
LUMO : -0.13271

GAP HOMO-LUMO: 2.924 eV

Relative stabilization energy +0.00 kcal mol^{-1}

Structure **2-3b**

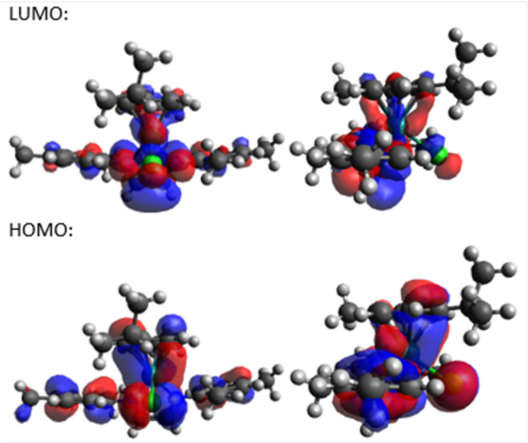

HOMO: -0.24184
LUMO : -0.13345

GAP HOMO-LUMO: 2.949 eV

Relative stabilization energy +2.41 kcal mol^{-1}

Structure **2-4b**

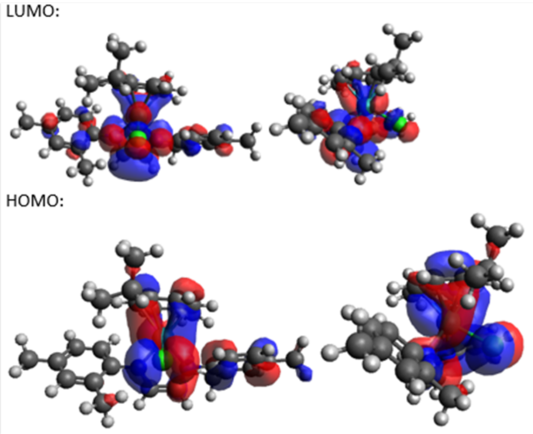

HOMO: -0.24460
LUMO : -0.13338

GAP HOMO-LUMO: 3.026 eV

Relative stabilization energy +1.63 kcal mol^{-1}

B3LYP/LanI2dz (Ru), 6-31G* (other atoms) and 6-33+G(d) for *p*-cymene (in CH_2Cl_2).

Fig.S.72: Representation of HOMO and LUMO orbitals for optimized structures **2-1b, 2-2b, 2-3b and 2-4b**.

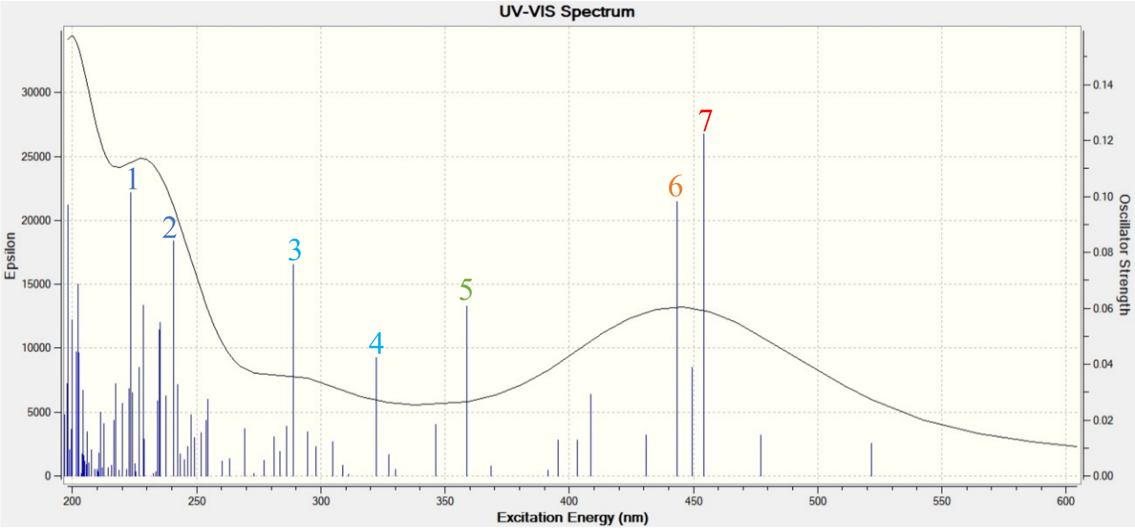

Fig. S.73: UV/vis of optimized structure **2-2a** calculated by DTDFT calculation.

Natural Transition Orbitals to describe the observed excited states for structure **2-2a**.

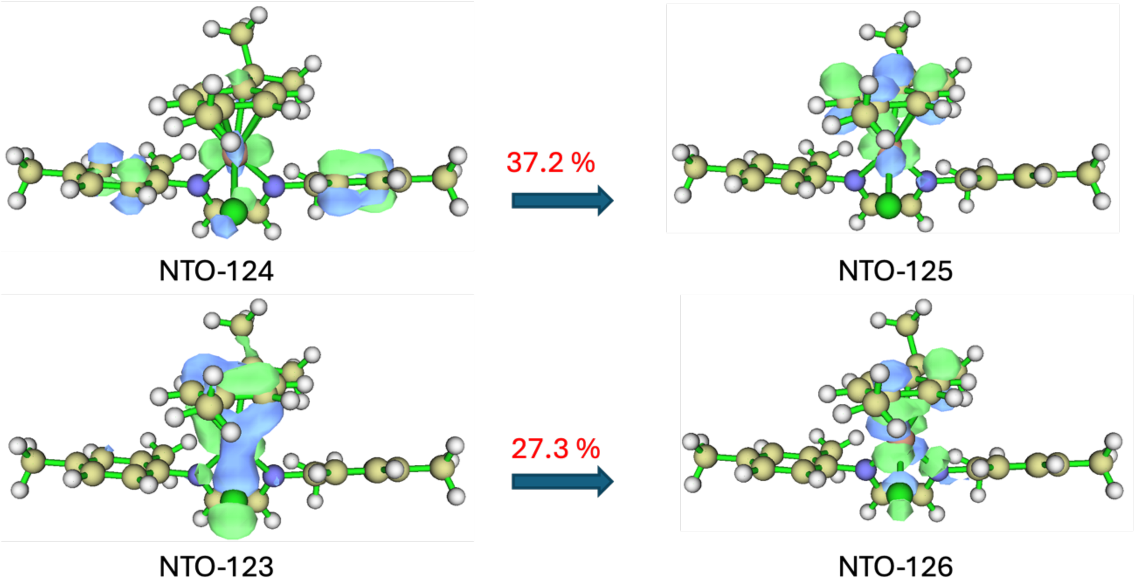

Scheme S.21: 1 Excited state 54 (223.33 nm, f = 0.1013).

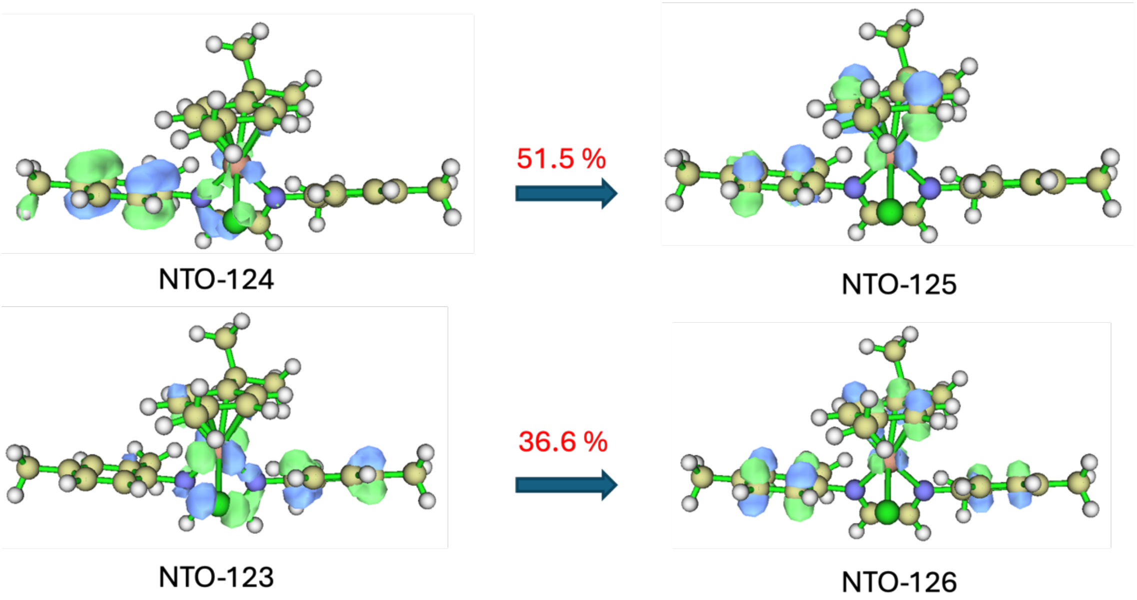

Scheme S. 22: 2 Excited state 41 (240.58 nm, f = 0.0840).

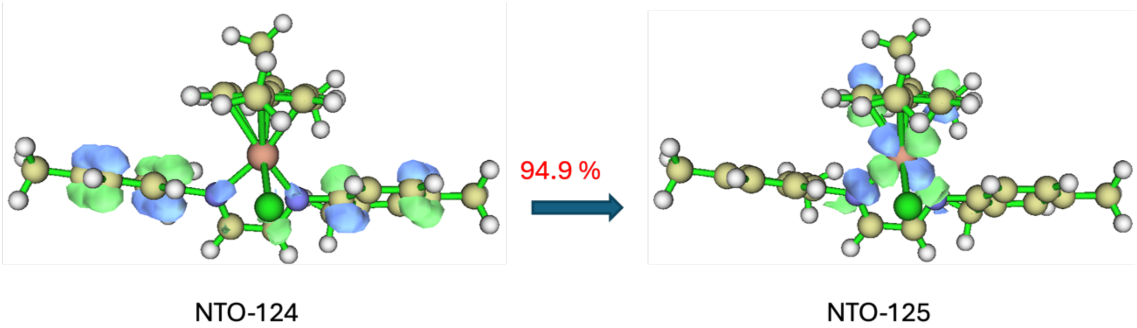

Scheme S. 23: 3 Excited state 23 (288.75 nm, f = 0.0757).

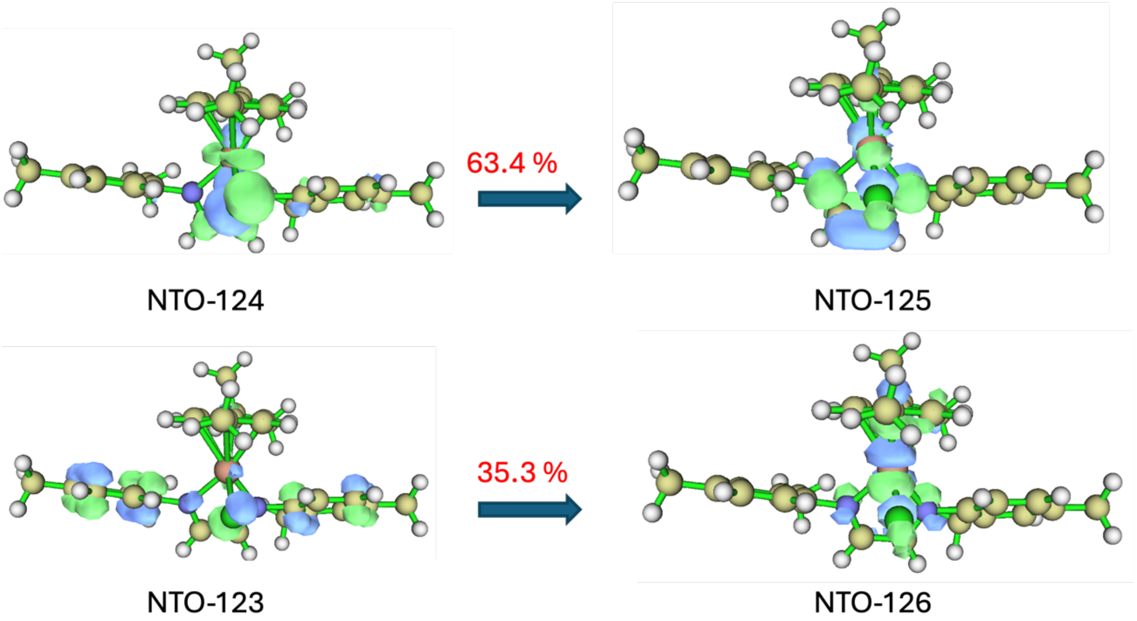

Scheme S.24: 4 Excited state 17 (322.36 nm, f = 0.0422).

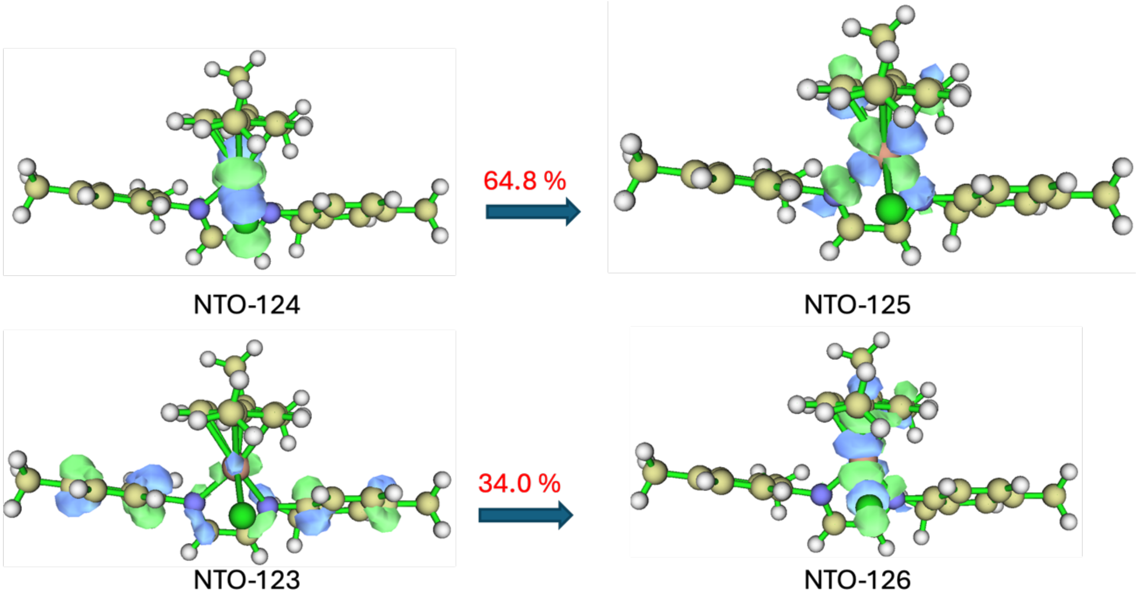

Scheme S. 25: 5 Excited state 13 (358.75 nm, f = 0.0608).

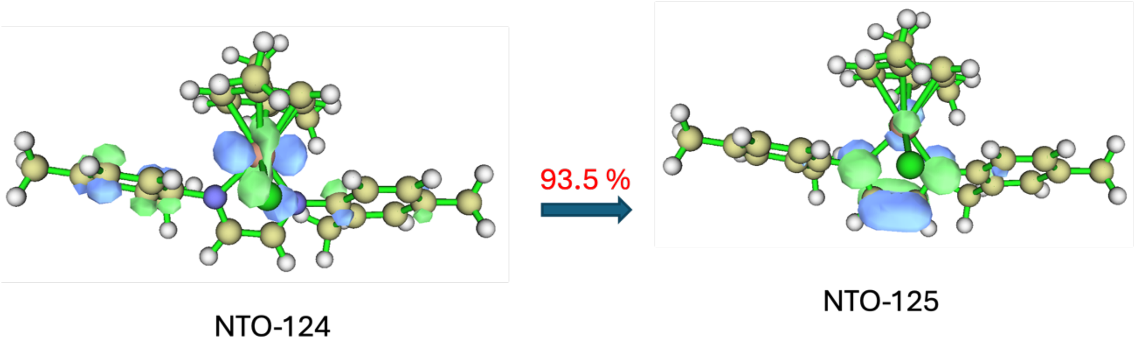

Scheme S.26: 6 Excited state 6 (443.56 nm, f = 0.0981).

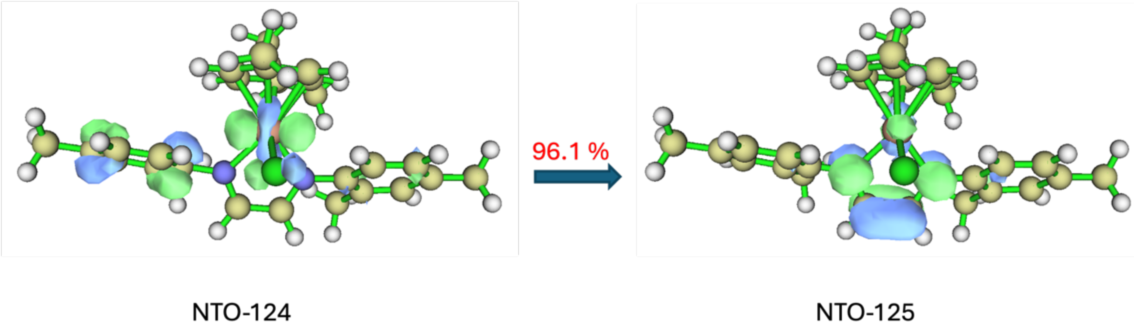

Scheme S.27: 7 Excited state 4 (454.24 nm, f = 0.1224).

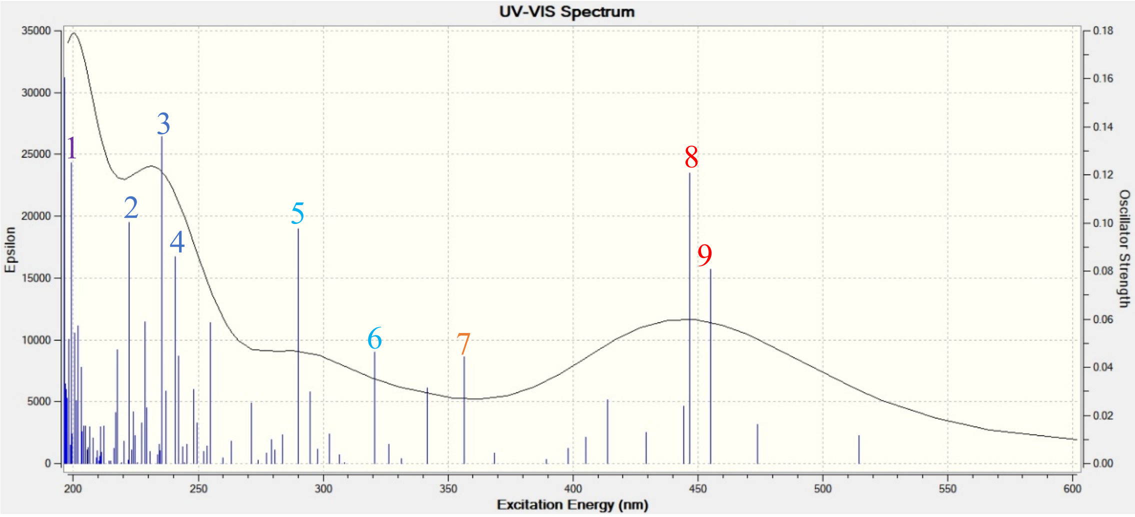

Fig.S.74: UV/vis of optimized structure **2-2b** calculated by DTDFT calculation

Natural Transition Orbitals to describe the observed excited states for structure **2-2b**.

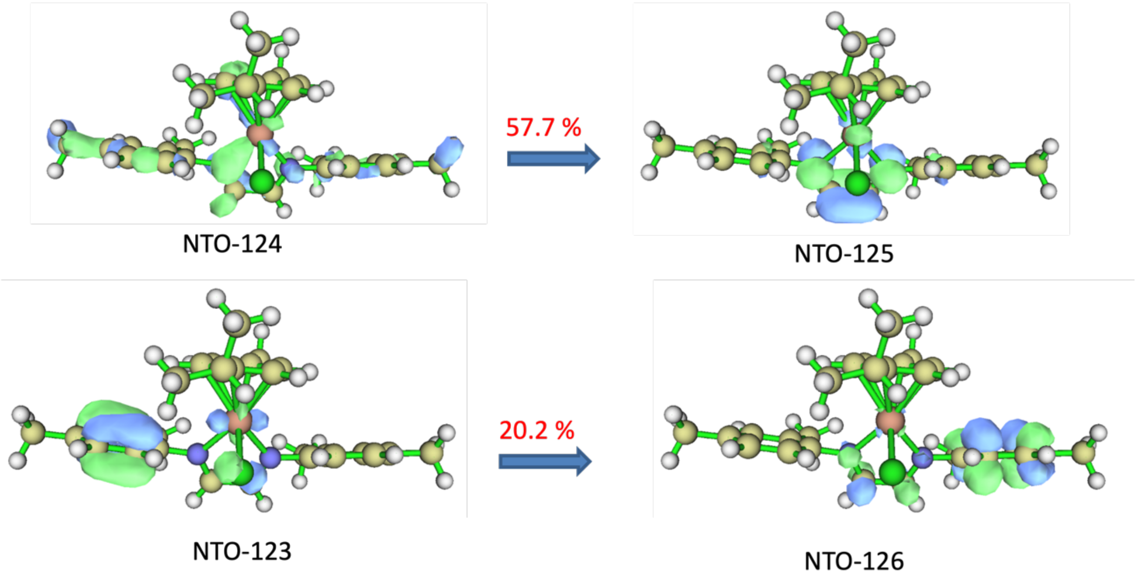

Scheme S.28:1 Excited state 88 (199.27 nm, f = 0.1251)

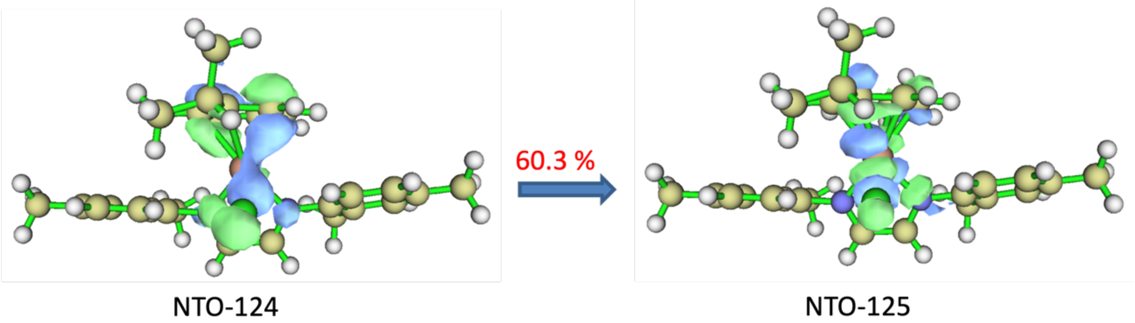

Scheme S. 29: 2 Excited state 55 (222.38 nm, f = 0.1003).

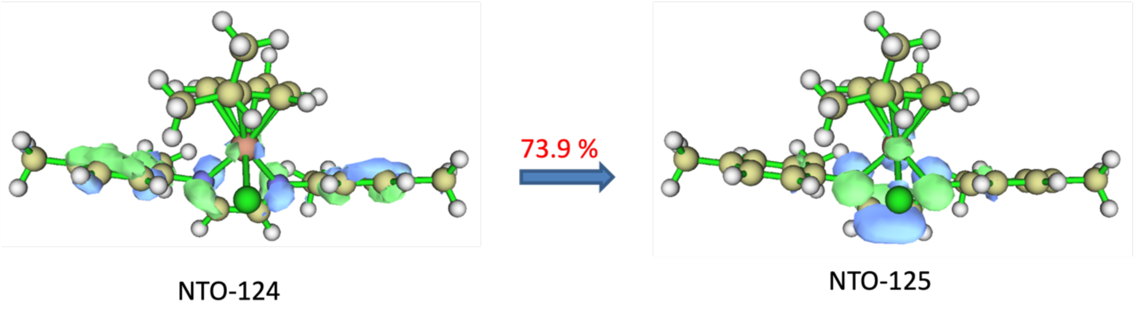

Scheme S.30: 3 Excited state 43 (235.30 nm, f = 0,1360).

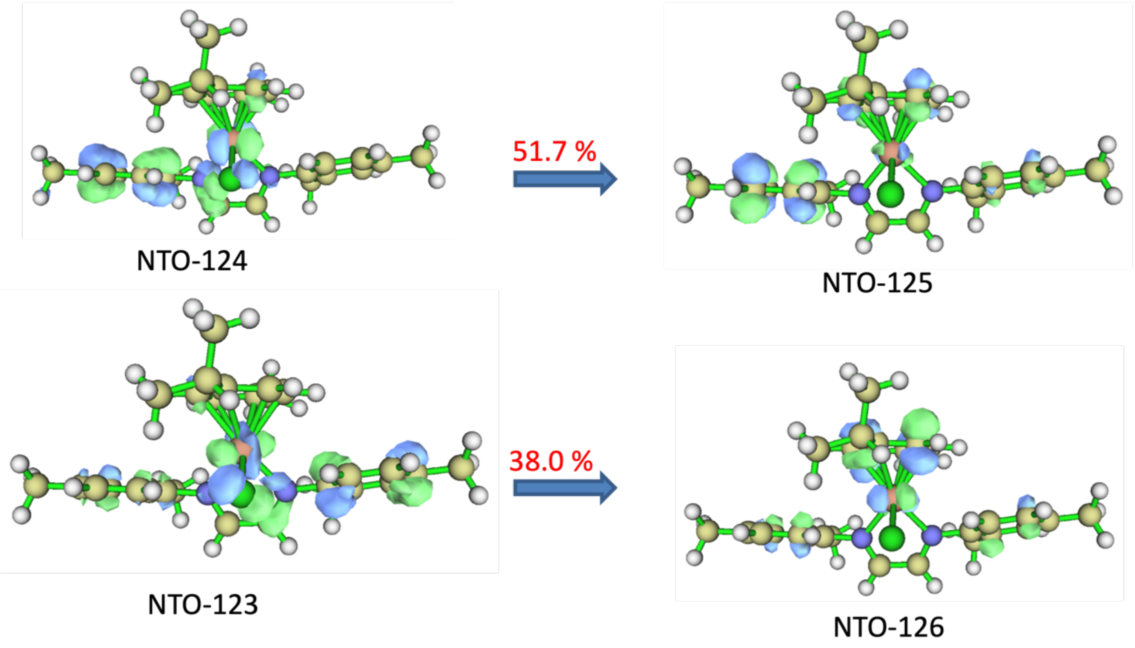

Scheme S. 31: 4 Excited state 41 (240.66 nm, f = 0.0862).

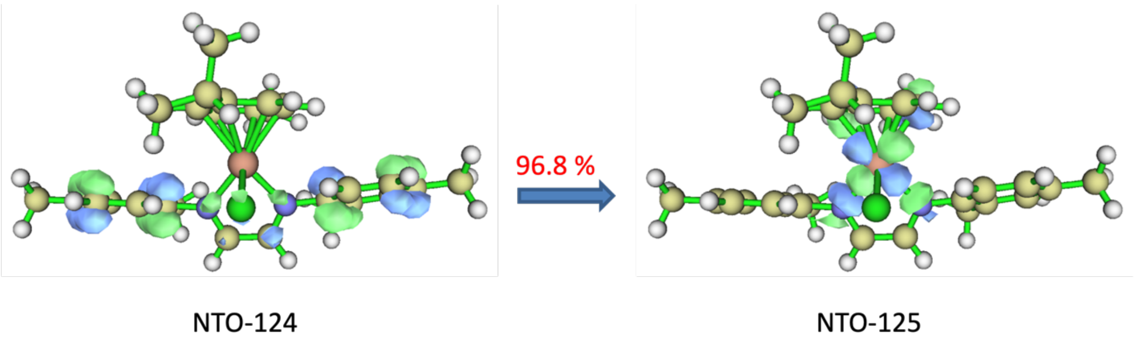

Scheme S.32: 5 Excited state 23 (290.06 nm, f = 0.0976)

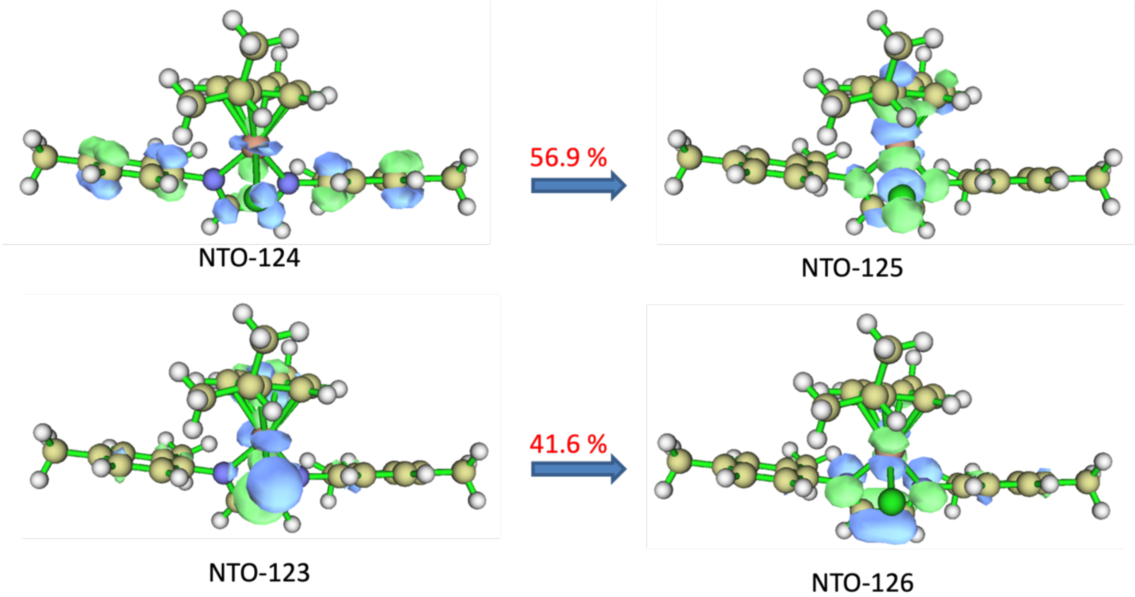

Scheme S.33: 6 Excited state 17 (320.74 nm, f = 0.0462).

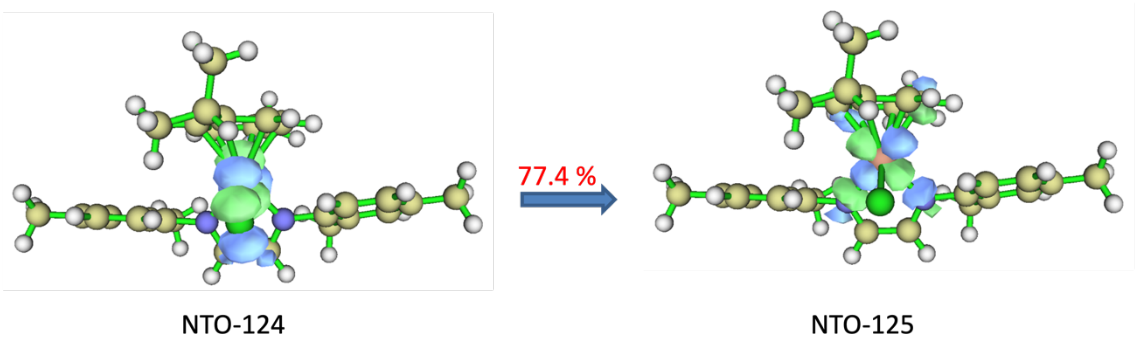

Scheme S.34: 7 Excited state 13 (356.46 nm, f = 0.0445).

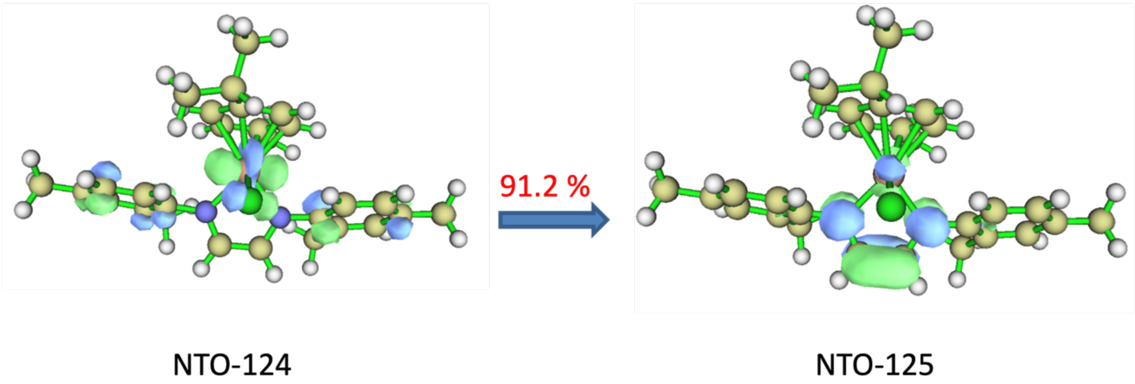

Scheme S.35: 8 Excited state 5 (446.69 nm, f = 0.1207).

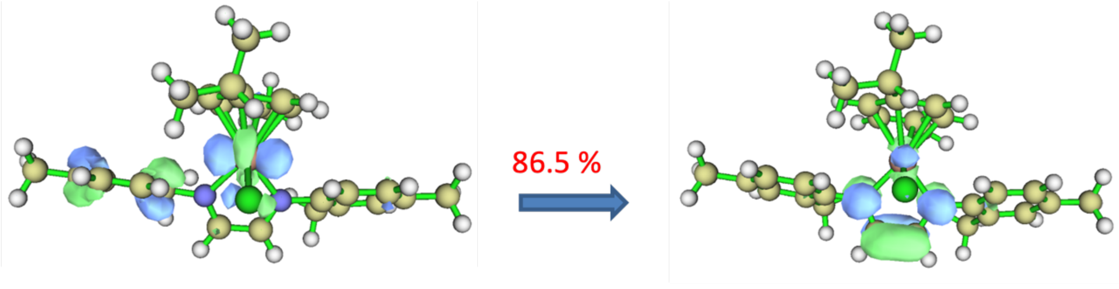

NTO-124 NTO-125

Scheme S. 36: 9 Excited state 4 (455.13 nm, f = 0.0806).

Table S: 35: Atomic dipole moment corrected Hirshfeld atomic charges, values of *f*+ and *f*- of structure **2-2a**.

		N	N-1 (cation)	N+1 (anion)	f^-	f^+
1	Ru	0.144266	0.181761	0.154777	0.037495	-0.010511
2	Cl	-0.390967	-0.242589	-0.468315	0.148378	0.077348
3	N	-0.045046	-0.023142	-0.133987	0.021904	0.088941
4	C	-0.058382	-0.068908	-0.038003	-0.010526	-0.020379
5	C	-0.025208	-0.055359	0.001877	-0.030151	-0.027085
6	N	-0.081042	-0.033061	-0.201739	0.047981	0.120697
7	C	0.090233	0.210222	0.005535	0.119989	0.084698
8	C	0.009843	0.021409	0.010481	0.011566	-0.000638
9	C	-0.249929	-0.589077	0.041355	-0.339148	-0.291284
10	C	-0.078451	-0.055522	-0.092602	0.022929	0.014151
11	C	-0.075137	0.047460	-0.181317	0.122597	0.106180
12	C	-0.019784	-0.000682	-0.034254	0.019102	0.014470
13	C	-0.670124	-0.734695	-0.601662	-0.064571	-0.068462
14	C	0.007614	0.012026	-0.004854	0.004412	0.012468
15	C	0,556389	0,724803	0.437780	0.168414	0.118609
16	C	0.512732	0.635437	0.405845	0.122705	0.106887
17	C	-0.056536	-0.056932	-0.060055	-0.000396	0.003519
18	C	-0.435054	-0.470924	-0.393292	-0.035870	-0.041762
19	C	-0.241559	-0.243423	-0.240757	-0.001864	-0.000802
20	C	-0.061597	-0.065422	-0.057935	-0,003825	-0.003662
21	C	-0.253810	-0.254859	-0.252331	-0.001049	-0.001479
22	C	-0.255942	-0.254346	-0.257782	0.001596	0.001840
23	C	0.036448	0.111942	-0.040545	0.075494	0.076993
24	C	0.005845	0.027924	-0.007721	0.022079	0.013566
25	C	-0.101582	-0.336218	0.175184	-0.234636	-0.276766
26	C	-0.108695	-0.068726	-0.109210	0.039969	0.000515
27	C	-0.107494	-0.027875	-0.202841	0.079619	0.095347
28	C	-0.021876	0.001433	-0.051325	0.023309	0.029449
29	C	-0.237419	-0.231915	-0.239119	0.005504	0.001700
30	C	-0.238377	-0.235043	-0.240234	0.003334	0.001857
31	C	-0.256306	-0.258559	-0.253473	-0.002253	-0.002833
32	C	-0.280414	-0.258586	-0.251818	0.021828	-0.028596

Table S: 36: Atomic dipole moment corrected Hirshfeld atomic charges, values of *f*+ and *f*- of structure **2-2b**.

		N (neutral)	N-1 (cation)	N+1 (anion)	*f*-	*f*+
1	Ru	0.356494	0.43622	0.3387	0.07973	0.017794
2	Cl	-0.374267	-0.244547	-0.436863	0.12972	0.062596
3	N	-0.052392	-0.037152	-0.122147	0.01524	0.069755
4	C	0.069682	0.101731	-0.016665	0.03205	0.086347
5	C	0.072421	0.104887	-0.014747	0.03247	0.087168
7	N	-0.054044	-0.037806	-0.124185	0.01624	0.070141
8	C	0.030127	0.042532	0.030737	0.01241	-0.000610
9	C	0.004706	0.019176	-0.007899	0.01447	0.012605
10	C	-0.040786	-0.02764	-0.053675	0.01315	0.012889
11	C	-0.044691	-0.026931	-0.058348	0.01776	0.013657
12	C	-0.039857	-0.024205	-0.054532	0.01565	0.014675
13	C	0.013533	0.040331	-0.006314	0.02680	0.019847
16	C	-0.02001	0.000603	-0.0359	0.02061	0.015890
17	C	0.02003	0.038662	0.004184	0.01863	0.015846
18	C	-0.02594	0.006513	-0.044202	0.03245	0.018262
19	C	-0.011807	0.009897	-0.029132	0.02170	0.017325
20	C	0.038429	0.059631	0.023015	0.02120	0.015414
22	C	-0.025773	0.011453	-0.044517	0.03723	0.018744
26	C	-0.002546	0.000519	-0.004918	0.00307	0.002372
29	C	0.031529	0.042367	0.030894	0.01084	0.000635
30	C	0.007343	0.02114	-0.008675	0.01380	0.016018
31	C	-0.03919	-0.027544	-0.055565	0.01165	0.016375
32	C	-0.043296	-0.026159	-0.058646	0.01714	0.015350
33	C	-0.038652	-0.023517	-0.055193	0.01514	0.016541
34	C	0.016874	0.042272	-0.007631	0.02540	0.024505
37	C	-0.075247	-0.067937	-0.082182	0.00731	0.006935
41	C	-0.076037	-0.068387	-0.081875	0.00765	0.005838
47	C	-0.080302	-0.075121	-0.084631	0.00518	0.004329
51	C	-0.079499	-0.07444	-0.084777	0.00506	0.005278
55	C	-0.076031	-0.069374	-0.080692	0.00666	0.004661
59	C	-0.080068	-0.076625	-0.082665	0.00344	0.002597
63	C	-0.064918	-0.056803	-0.070862	0.00812	0.005944

Complex 3

3-a 3-b

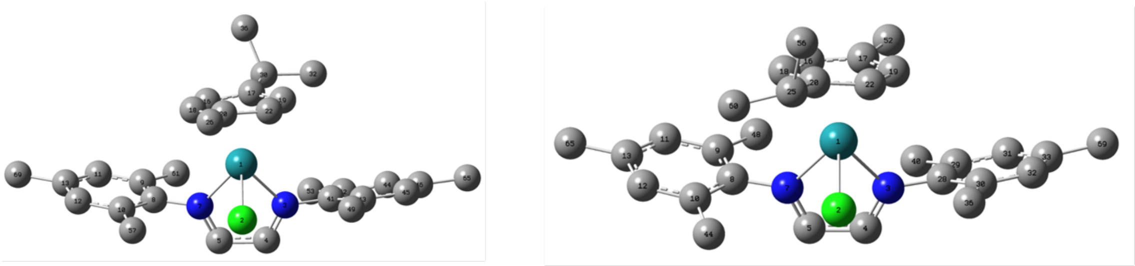

Fig. S 75: Atomic numbering for optimized structures **3-a** and **3-b** obtained by DFT calculation.

Table S.37: Bond length structural values for optimized structures **3-a** and **3-b** were calculated using DFT B3LYP/Lanl2dz (Ru) and 6-31G* (for all other atoms), with 6-31+G(d) applied to the p-cymene ligand in dichloromethane.

Bond	Complex **3-a** (Å)	Complex **3-b** (Å)
Ru-N (1)	2.0901	2.0762
N-C (2)	1.4443	1.4439
N=C (3)	1.3033	1.3025
C-C (4)	1.4440	1.4451
Ru-Cl (5)	2.4325	2.4361
Ru-C (6)	1.7978	1.8206

Table S.38: Percentual composition of the HOMO and LUMO orbitals for structures **3-a** and **3-b**.

Orbital	% Ru	% Cl	% *p*-cymene	% **N-N**[3]
HOMO **3-a**	33.12	21.06	12.36	33.46
LUMO **3-a**	8.95	3.62	5.38	82.05
HOMO **3-b**	33.71	21.75	11.81	32.73
LUMO **3-b**	9.03	3.67	5.39	81.91

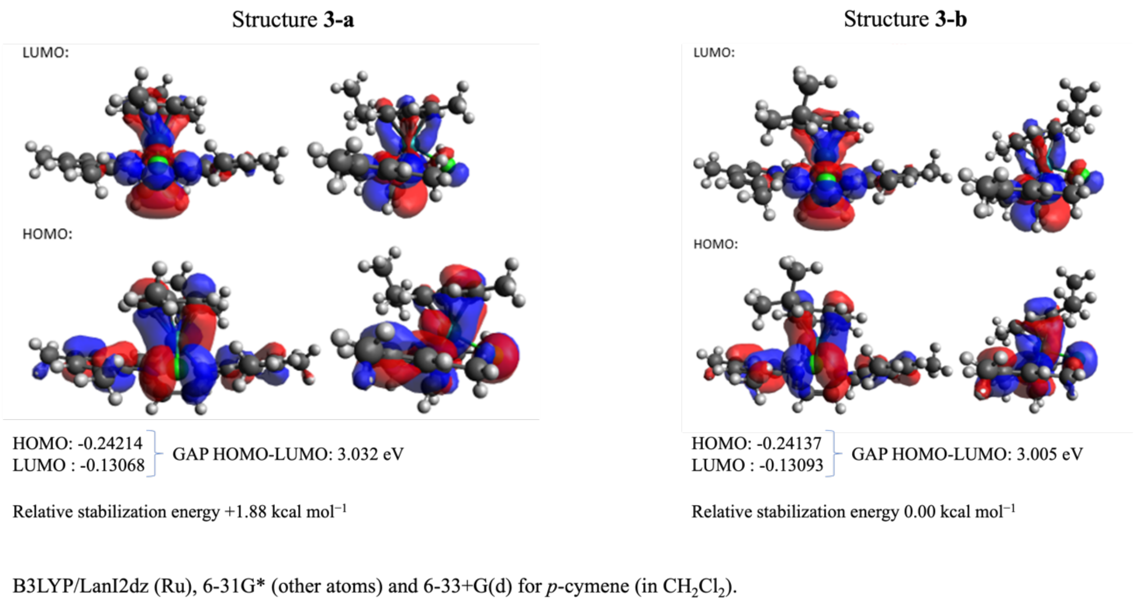

Fig. S 76: Representation of HOMO and LUMO orbitals for optimized structures **3-a** and **3-b**.

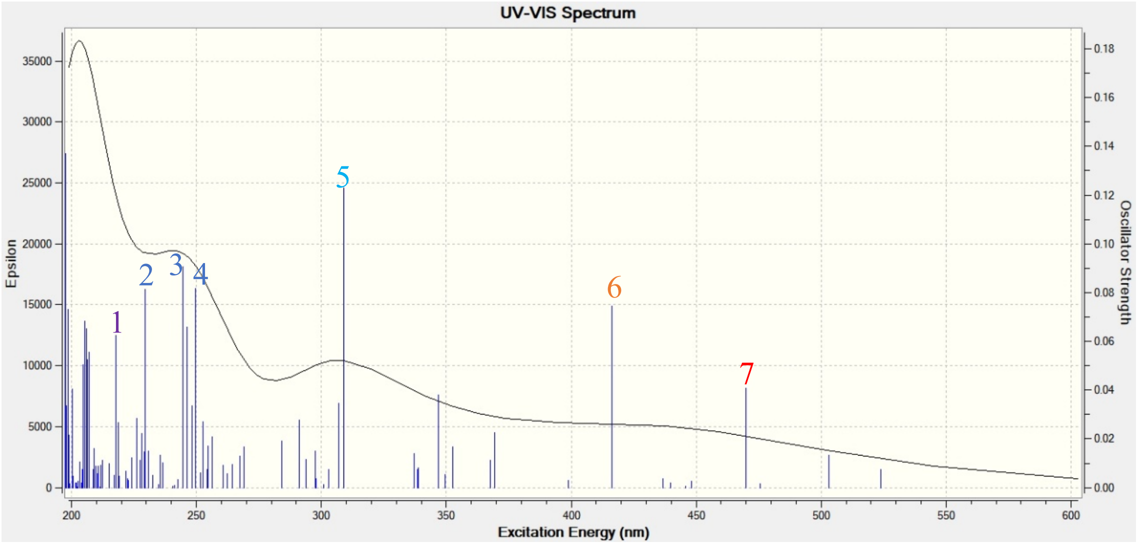

Fig. S. 77: UV/vis of optimized structure **3-a** calculated by DTDFT calculation.

Natural Transition Orbitals to describe the observed excited states for structure **3-a**.

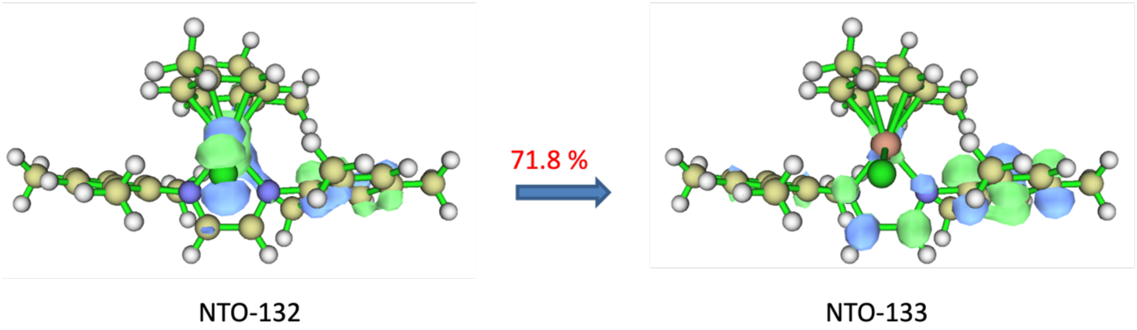

Scheme S. 37: 1 Excited state 62 (217.81 nm, f = 0.0626).

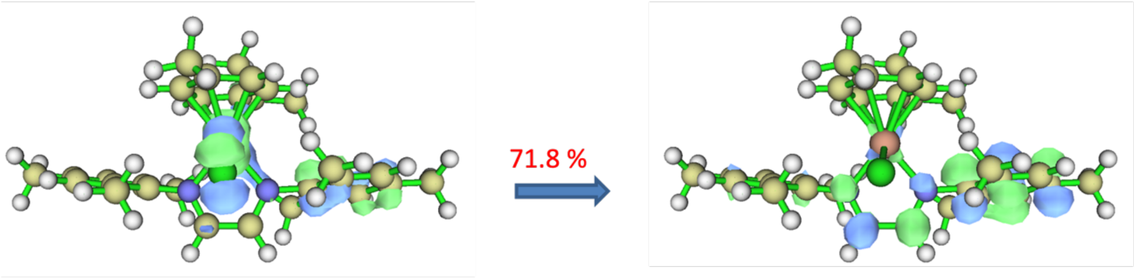

NTO-132 NTO-133

Scheme S. 38: 2 Excited state 51 (229.40 nm, f = 0.0813)

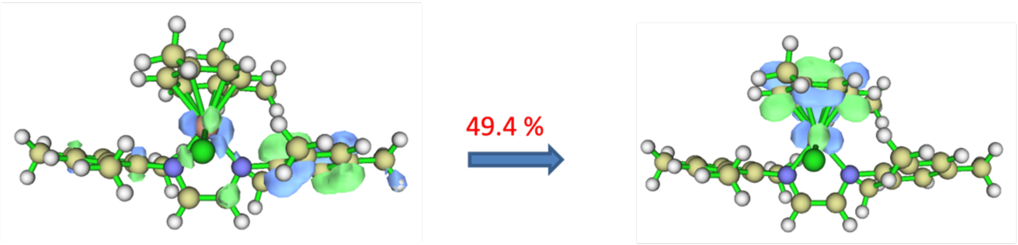

NTO-132 NTO-133

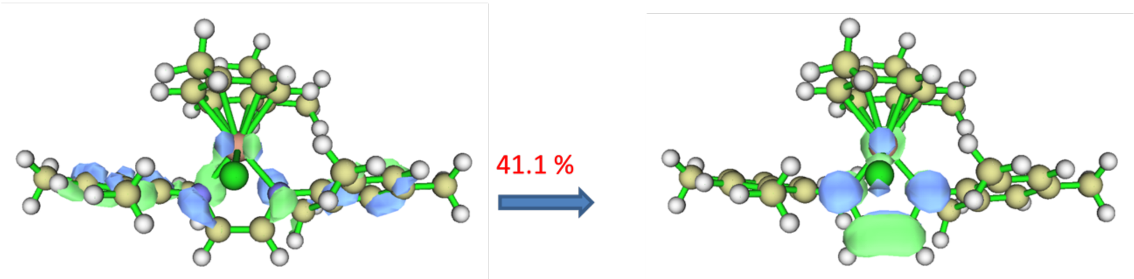

NTO-131 NTO-134

Scheme S. 39: 3 Excited state 42 (244.58 nm, f = 0.0906)

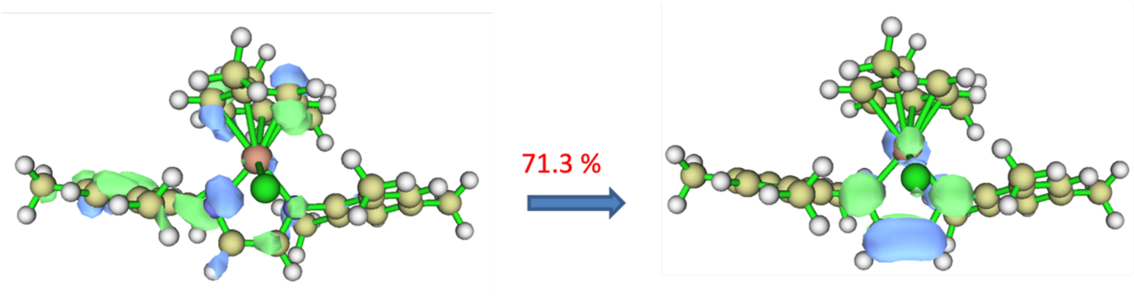

NTO-132 NTO-133

Scheme S.40: 4 Excited state 39 (249.55 nm, f = 0.0818)

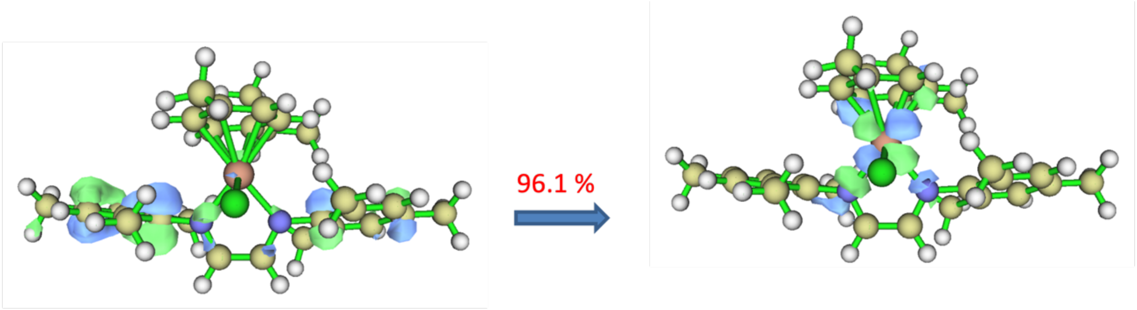

NTO-132 NTO-133

Scheme S. 41: 5 Excited state 20 (309.03 nm, f = 0.1228).

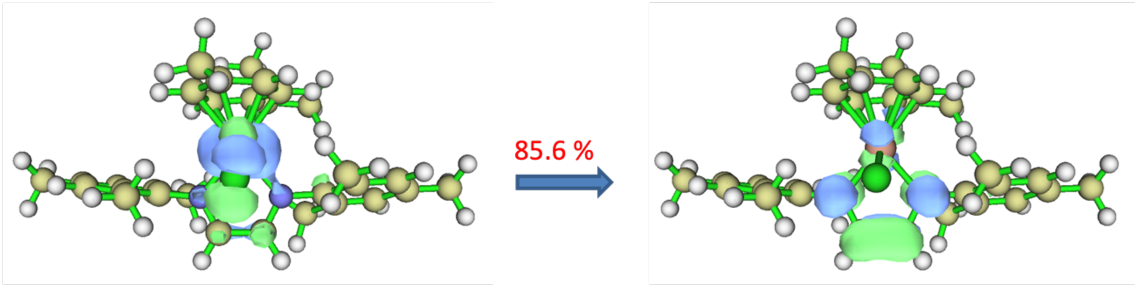

NTO-132 NTO-133

Scheme S. 42: 6 Excited state 10 (416.37 nm, f = 0.0746).

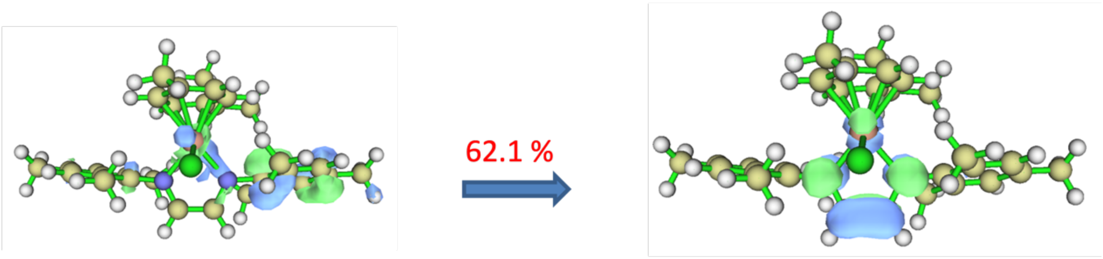

NTO-132 NTO-133

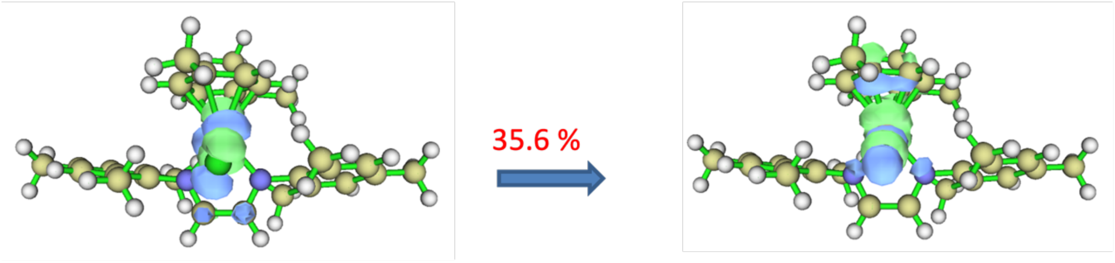

NTO-131 NTO-134

Scheme S.43: 7 Excited state 5 (469.93 nm, f = 0.0407)

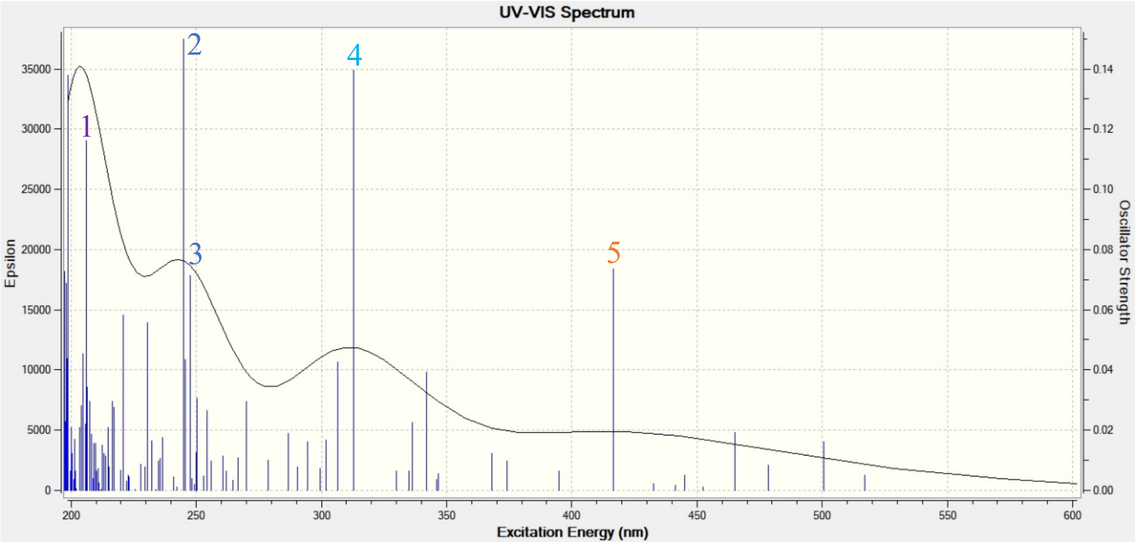

Fig. S 78: UV/vis of optimized structure **3-b** calculated by DTDFT calculation.

Natural Transition Orbitals to describe the observed excited states for structure **3-b**.

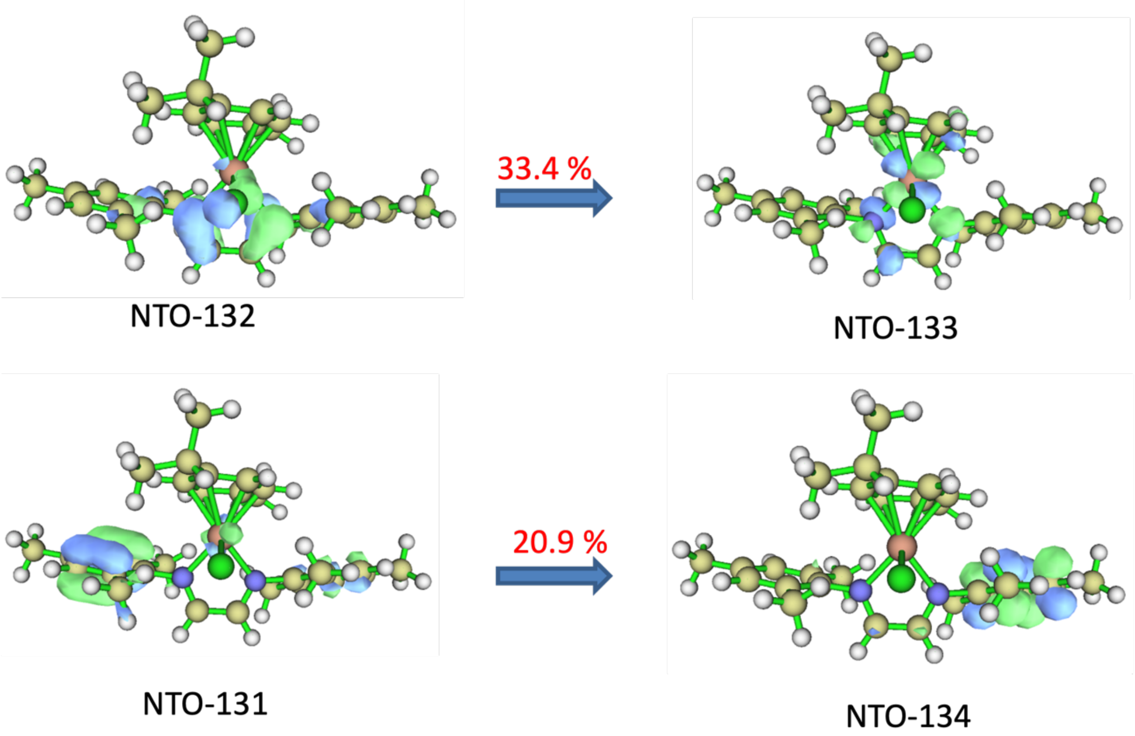

Scheme S.44:1 Excited State 81 (206.26 nm, f = 0,1163).

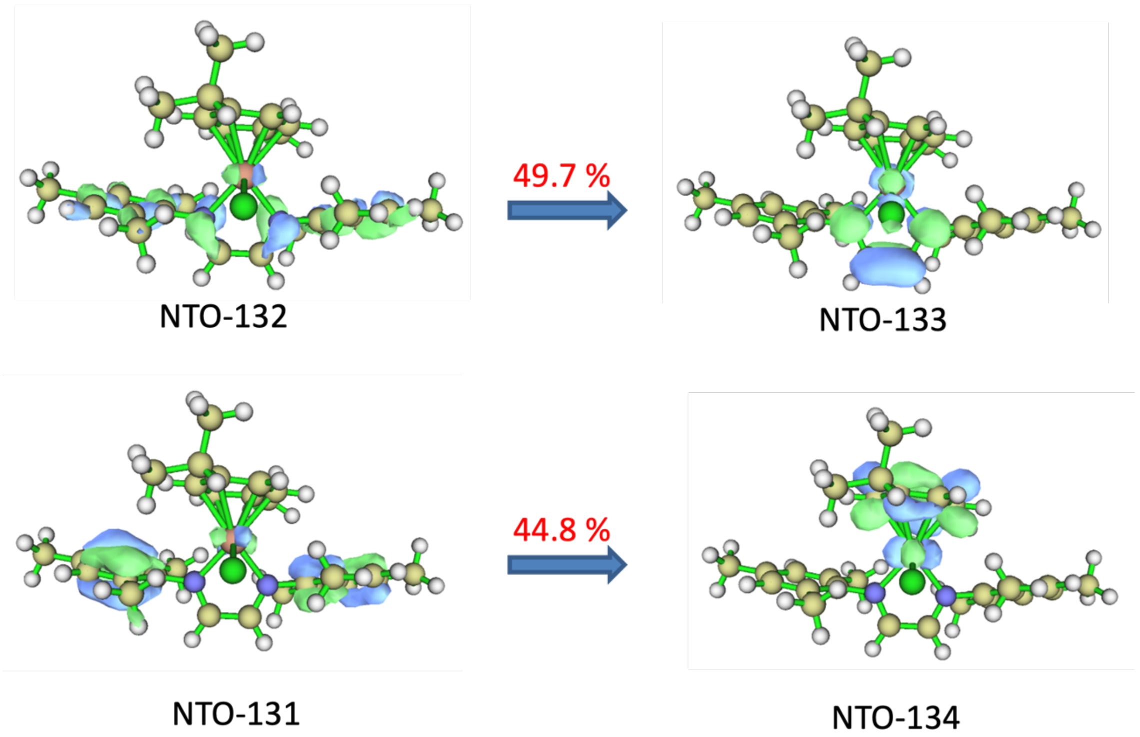

Scheme S. 45: 2 Excited state 43 (245.02 nm, f = 0.1499).

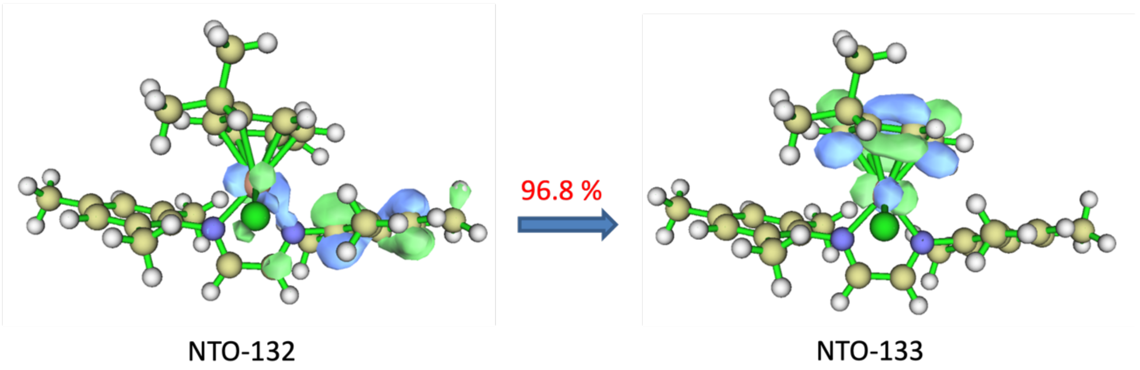

Scheme S.46: 3 Excited state 41 (247.70 nm, f = 0,0712).

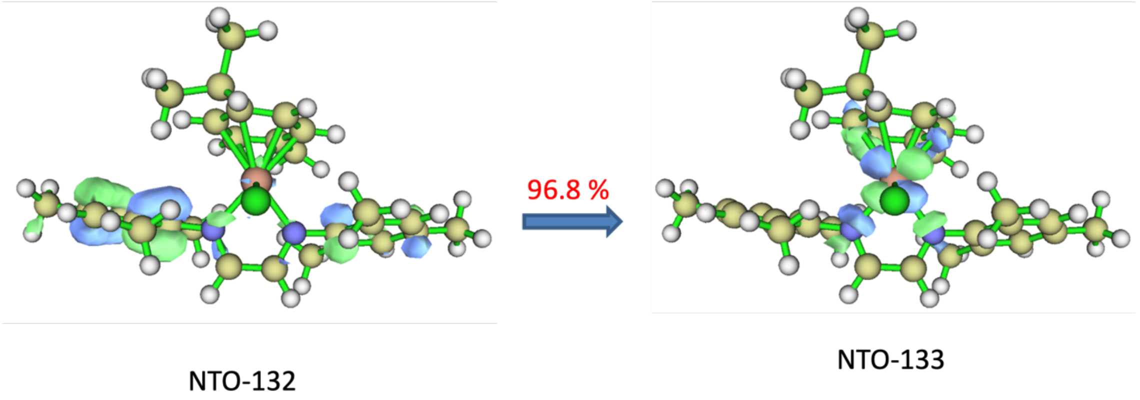

Scheme S. 47:4 Excited state 20 (312.92 nm, f = 0.1396).

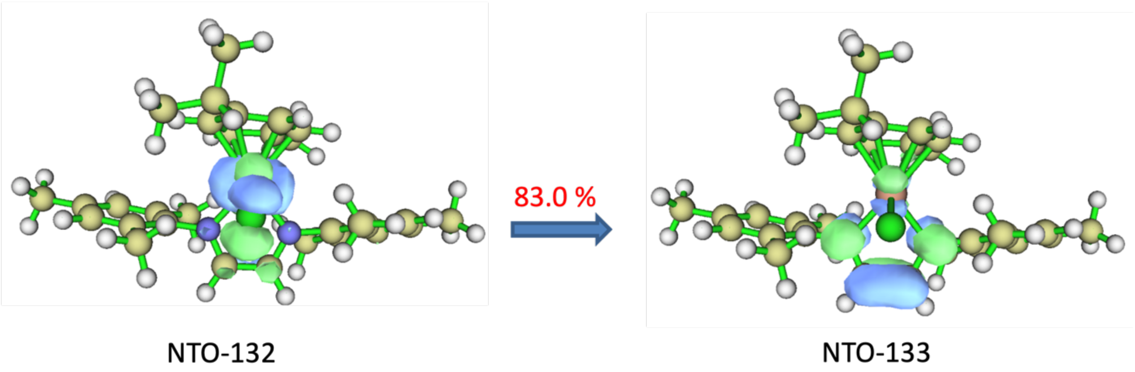

Scheme S. 48: 5 Excited state 10 (416.63 nm, f = 0.0736)

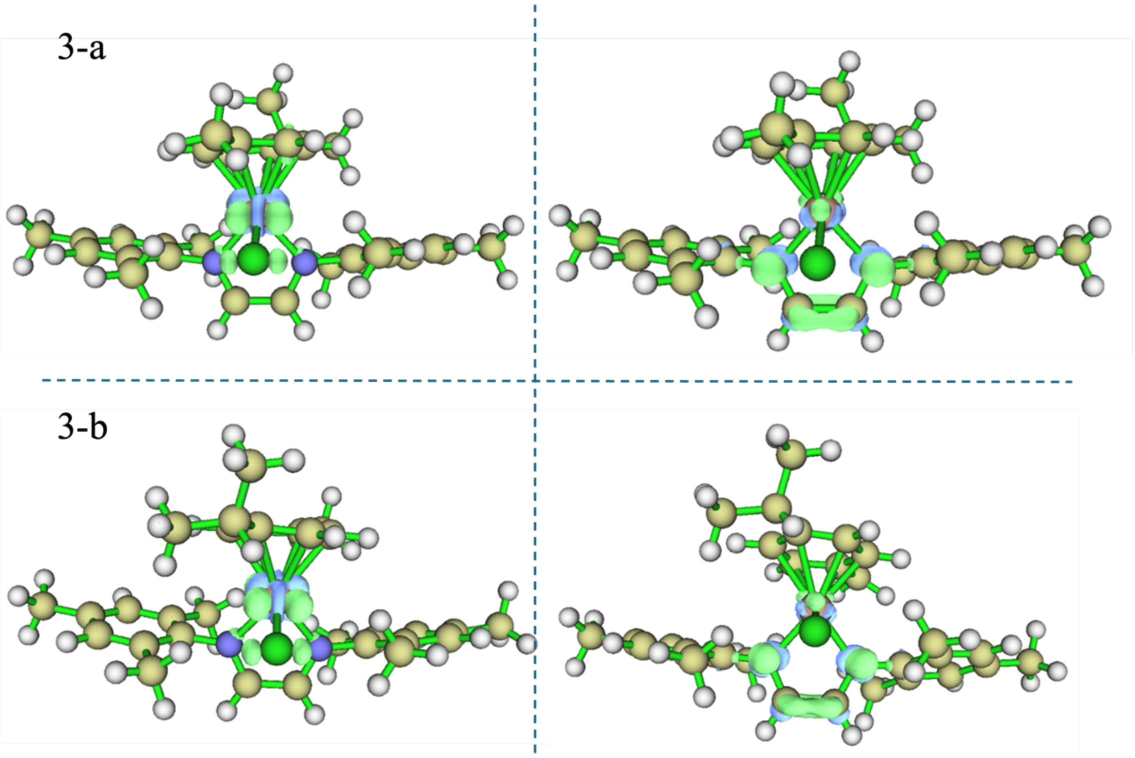

Fig. S.79: Fukui function for the optimized structures **3-a** and **3-b**, *f*- (left) represents an electrophilic attack region and *f*+ (right) represents a nucleophilic attack region.

Scheme S. 49: Atomic dipole moment corrected Hirshfeld atomic charges, values of *f*+ and *f*- of structure **3-a.**

		N (neutral)	N-1 (cation)	N+1 (anion)	*f*-	*f*+
1	Ru	0.355387	0.438286	0.337081	0.082899	0.018306
2	Cl	-0.32606	-0.18143	-0.39433	0.144630	0.068270
3	N	-0.05364	-0.03818	-0.12589	0.015460	0.072250
4	C	0.073923	0.107651	-0.01504	0.033728	0.088963
5	C	0.077688	0.110815	-0.01246	0.033127	0.090148
7	N	-0.05226	-0.03654	-0.12255	0.015720	0.070290
8	C	0.025264	0.033638	0.029439	0.008374	-0.004175
9	C	-0.00013	0.00917	-0.00698	0.009300	0.006850
10	C	0.00502	0.016728	-0.00215	0.011708	0.007170
11	C	-0.05102	-0.03306	-0.06347	0.017960	0.012450
12	C	-0.04939	-0.03622	-0.06183	0.013170	0.012440
13	C	0.008369	0.031909	-0.00638	0.023540	0.014749
16	C	-0.01977	-0.00176	-0.03515	0.018010	0.015380
17	C	0.024788	0.044597	0.006477	0.019809	0.018311
18	C	-0.02563	0.006837	-0.04704	0.032467	0.021410
19	C	-0.01038	0.010497	-0.03	0.020877	0.019620
20	C	0.044478	0.065086	0.025293	0.020608	0.019185
22	C	-0.02609	0.014856	-0.04712	0.040946	0.021030
26	C	-0.06589	-0.05678	-0.07348	0.009110	0.007590
30	C	-0.00115	0.002197	-0.00355	0.003347	0.002400
32	C	-0.08187	-0.07846	-0.08469	0.003410	0.002820
36	C	-0.07657	-0.07076	-0.08171	0.005810	0.005140
41	C	0.027493	0.032114	0.02984	0.004621	-0.002347
42	C	0.00441	0.014616	-0.00435	0.010206	0.008760
43	C	0.006969	0.014277	-0.00306	0.007308	0.010029
44	C	-0.04757	-0.035	-0.06087	0.012570	0.013300
45	C	-0.04855	-0.03488	-0.06209	0.013670	0.013540
46	C	0.011527	0.032046	-0.00672	0.020519	0.018247
49	C	-0.08762	-0.0829	-0.09201	0.004720	0.004390
53	C	-0.08181	-0.07769	-0.08563	0.004120	0.003820
57	C	-0.08801	-0.0821	-0.09154	0.005910	0.003530
61	C	-0.0819	-0.07744	-0.08519	0.004460	0.003290

65	C	-0.07596	-0.06994	-0.08151	0.006020	0.005550
69	C	-0.07671	-0.06986	-0.08144	0.006850	0.004730

Table S: 39: Atomic dipole moment corrected Hirshfeld atomic charges, values of *f*+ and *f*- of structure **3-b.**

		N (neutral)	N-1 (cation)	N+1 (anion)	*f*-	*f*+
1	Ru	0,35552	0,446605	0,335204	0,091085	0,020316
2	Cl	-0,3271	-0,177251	-0,396039	0,149849	0,068939
3	N	-0,0517	-0,036831	-0,123467	0,014869	0,071767
4	C	0,074975	0,109581	-0,015648	0,034606	0,090623
5	C	0,079434	0,112962	-0,011970	0,033528	0,091404
7	N	-0,05158	-0,035828	-0,123260	0,015752	0,071680
8	C	0,025026	0,033270	0,028778	0,008244	-0,003752
9	C	-0,00029	0,007678	-0,006953	0,007968	0,006663
10	C	0,004741	0,018570	-0,002686	0,013829	0,007427
11	C	-0,05159	-0,031919	-0,063765	0,019671	0,012175
12	C	-0,05007	-0,037921	-0,062087	0,012149	0,012017
13	C	0,007779	0,029494	-0,006430	0,021715	0,014209
16	C	-0,02097	0,000954	-0,038990	0,021924	0,018020
17	C	0,026487	0,045944	0,010225	0,019457	0,016262
18	C	-0,01947	0,009648	-0,041049	0,029118	0,021579
19	C	-0,01363	0,010200	-0,034928	0,023830	0,021298
20	C	0,034999	0,058954	0,017915	0,023955	0,017084
22	C	-0,01818	0,015057	-0,038900	0,033237	0,020720
25	C	-0,00329	0,000268	-0,005947	0,003558	0,002657
28	C	0,026857	0,028516	0,029881	0,001659	-0,003024
29	C	0,003759	0,011874	-0,004581	0,008115	0,008340
30	C	0,00658	0,012897	-0,002661	0,006317	0,009241
31	C	-0,0486	-0,036583	-0,062077	0,012017	0,013477
32	C	-0,0479	-0,036128	-0,061510	0,011772	0,013610
33	C	0,011377	0,029467	-0,006414	0,018090	0,017791
36	C	-0,08692	-0,082742	-0,091087	0,004178	0,004167
40	C	-0,08222	-0,078690	-0,085855	0,003530	0,003635
44	C	-0,08965	-0,081553	-0,093376	0,008097	0,003726
48	C	-0,08217	-0,077753	-0,085377	0,004417	0,003207

52	C	-0,0656	-0,057091	-0,071934	0,008509	0,006334
56	C	-0,07645	-0,069247	-0,081487	0,007203	0,005037
60	C	-0,07952	-0,075900	-0,082346	0,003620	0,002826
65	C	-0,07689	-0,070438	-0,081484	0,006452	0,004594
69	C	-0,07594	-0,070599	-0,081407	0,005341	0,005467

Complex **4**

4-a **4-b**

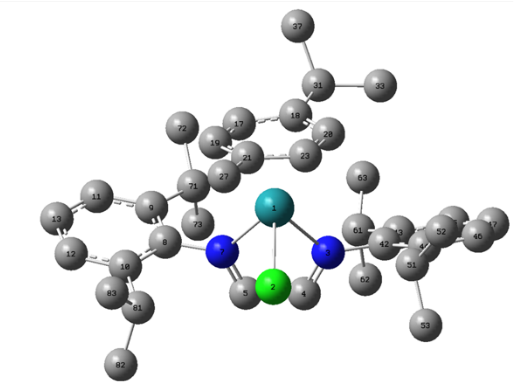

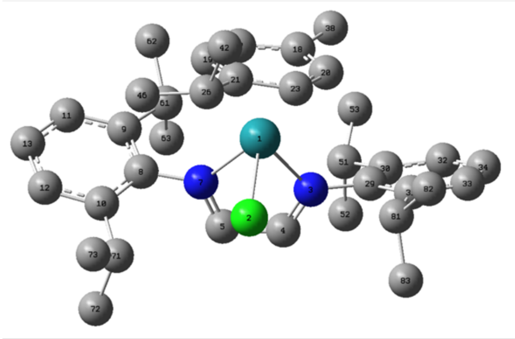

Table S: 40: Atomic numbering for optimized structures **4-a** and **4-b** obtained by DFT calculation.

Table S: 41: Bond length structural values for optimized structures **4-a** and **4-b** were calculated using DFT B3LYP/Lanl2dz (Ru) and 6-31G* (for all other atoms), with 6-31+G(d) applied to the p-cymene ligand in dichloromethane.

Bond	Complex **4-a** (Aº)	Complex **4-b** (Aº)
Ru-N (1)	2.0987	2.0871
N-C (2)	1.4485	1.4503
N=C (3)	1.3044	1.3028
C-C (4)	1.4452	1.4439
Ru-Cl (5)	2.4390	2.4405
Ru-C (6)	1.8010	1.8031

Table S.42: Percentual composition of the HOMO and LUMO orbitals for structures **4-a** and **4-b**

Orbital	% Ru	% Cl	% *p*-cimeno	% 2,6-diisopropil
HOMO **4-a**	39.67	23.27	12.00	25.06
LUMO **4-a**	9.10	3.85	5.32	81.73
HOMO **4-b**	42.53	25.18	12.43	19.86
LUMO **4-b**	8.30	3.50	5.10	83.10

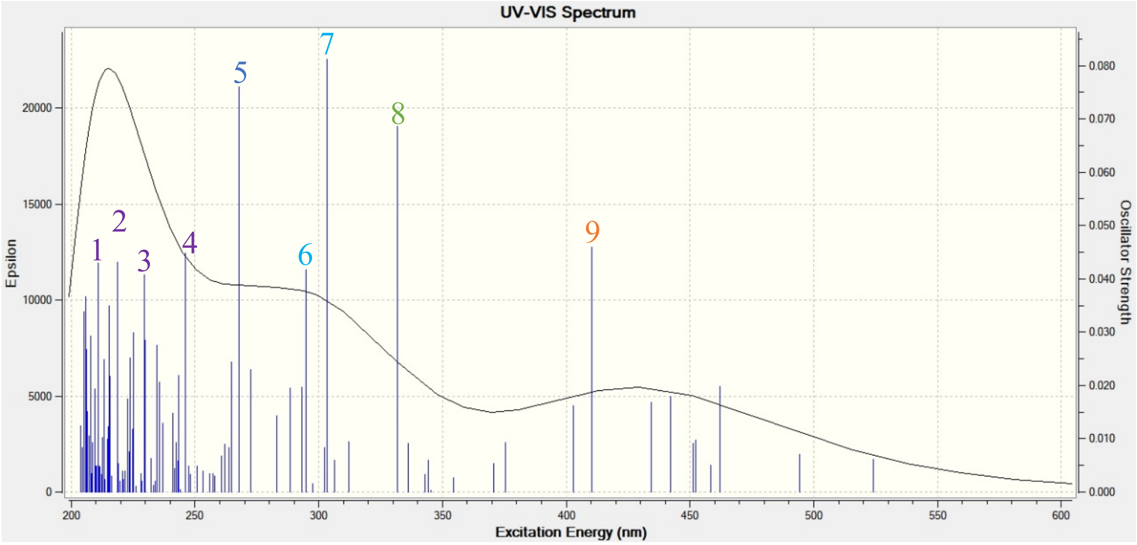

Fig. S.80: UV/vis of optimized structure **4-a** calculated by DTDFT calculation.

Natural Transition Orbitals to describe the observed excited states for structure **4-a**.

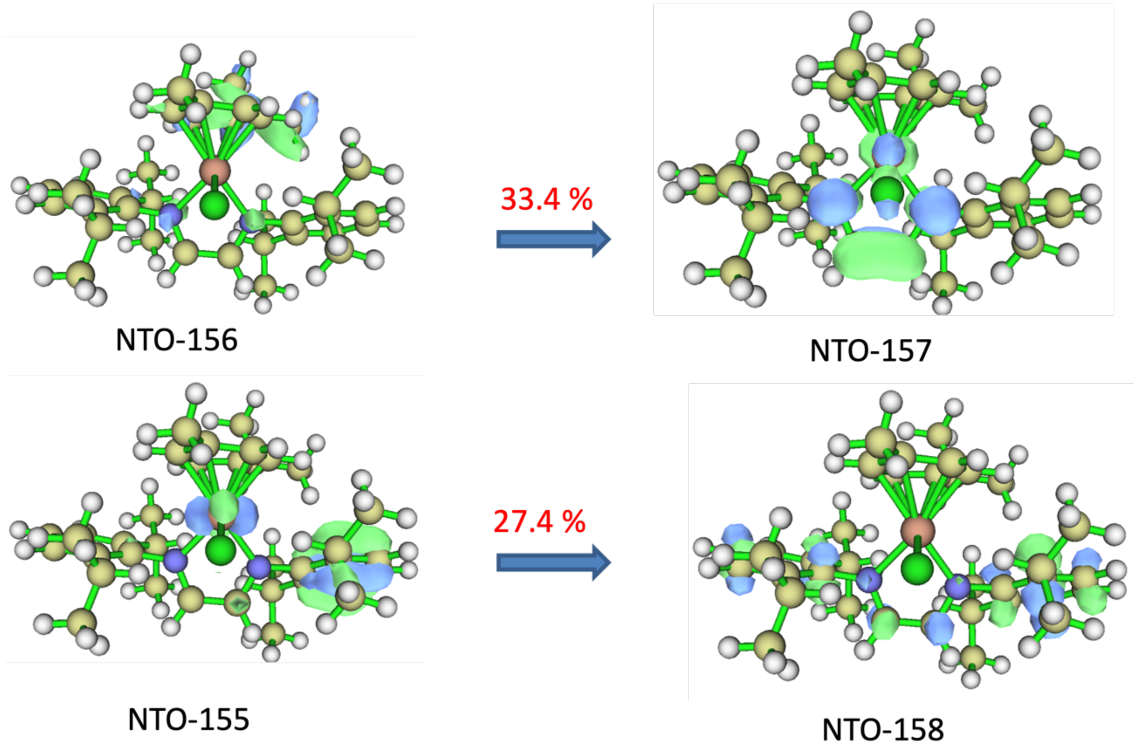

Scheme S.50:1 Excited state 84 (210.79 nm, f = 0.0429).

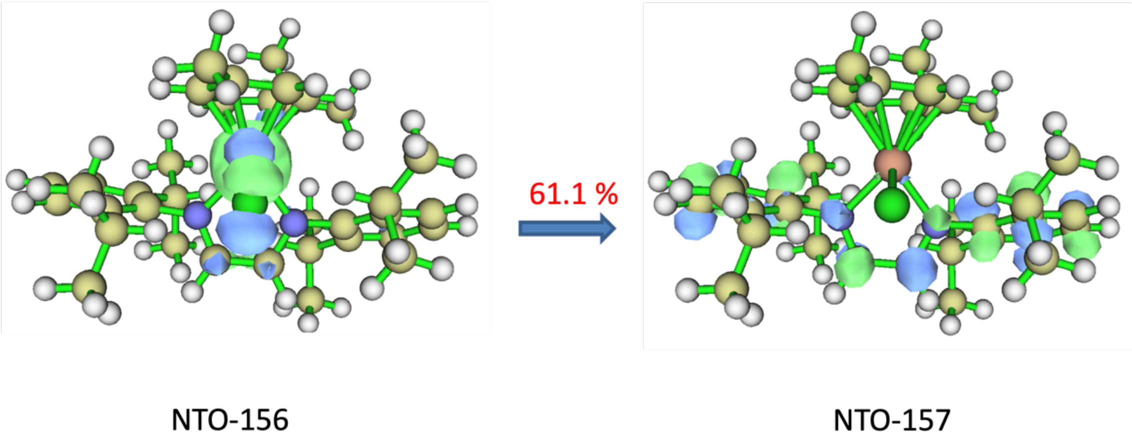

Scheme S.51: 2 Excited state 71 (218.71 nm, f = 0.0431).

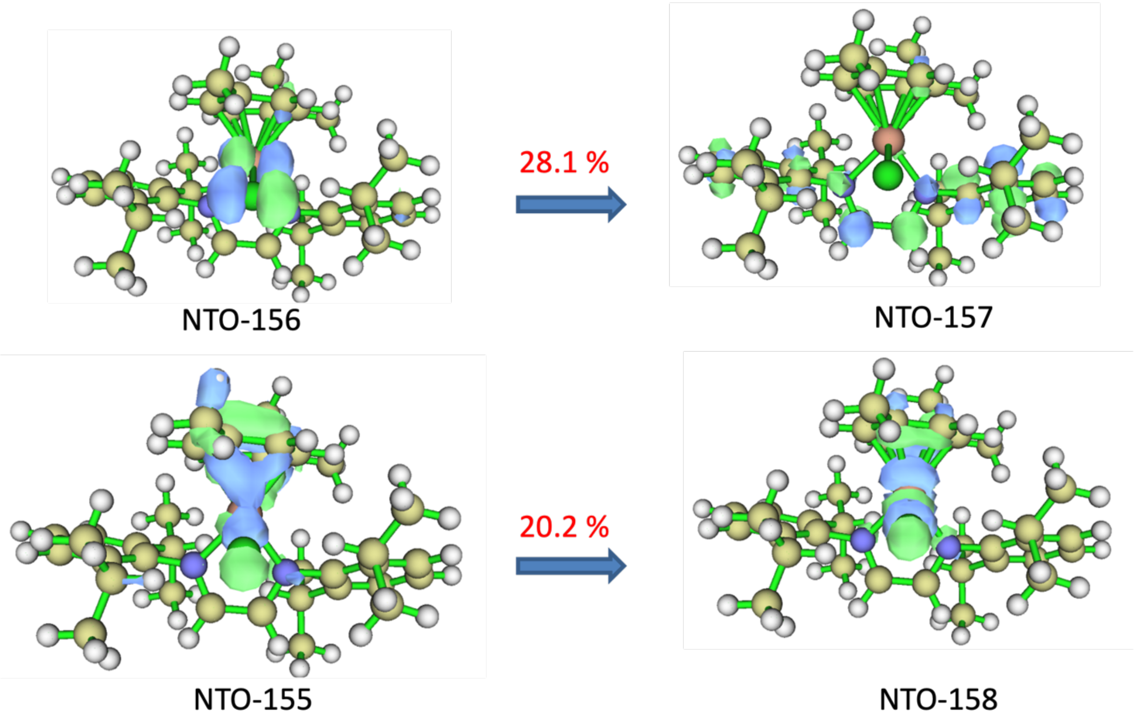

Scheme S.52: 3 Excited state 57 (229.62 nm, f = 0.0408).

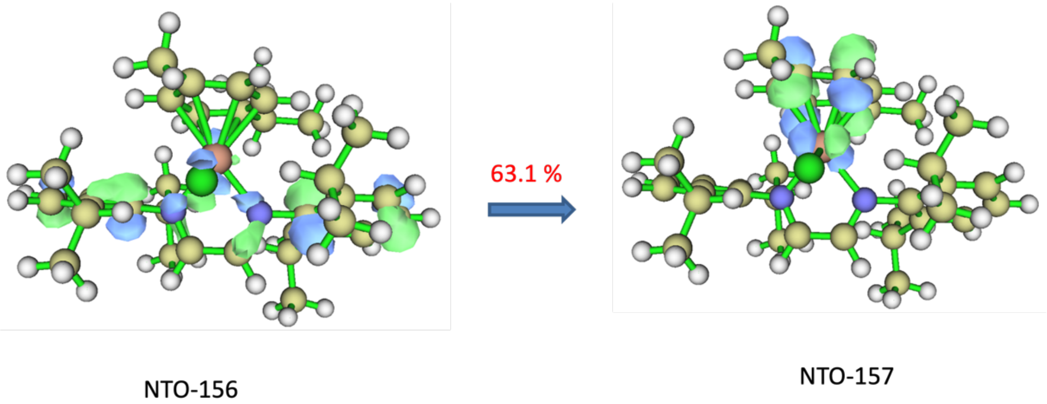

Scheme S. 53: 4 Excited state 43 (246.25 nm, f = 0.0448).

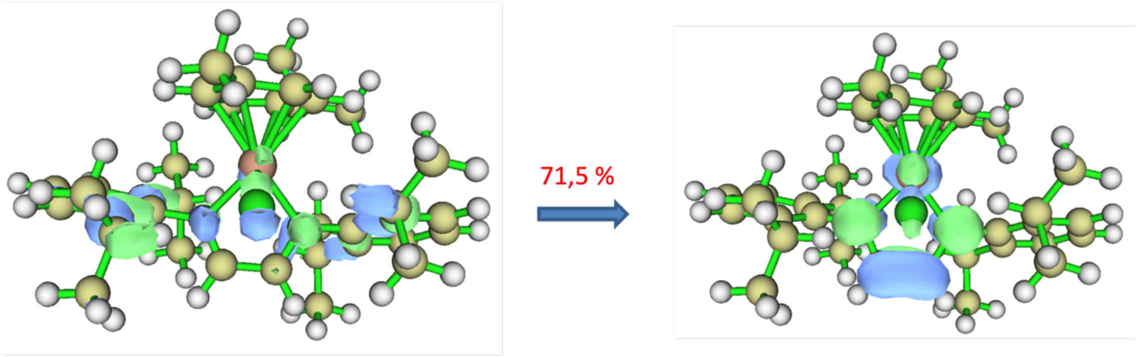

NTO-156 NTO-157

Scheme S. 54: 5 Excited state 31 (267.64 nm, f = 0.0760).

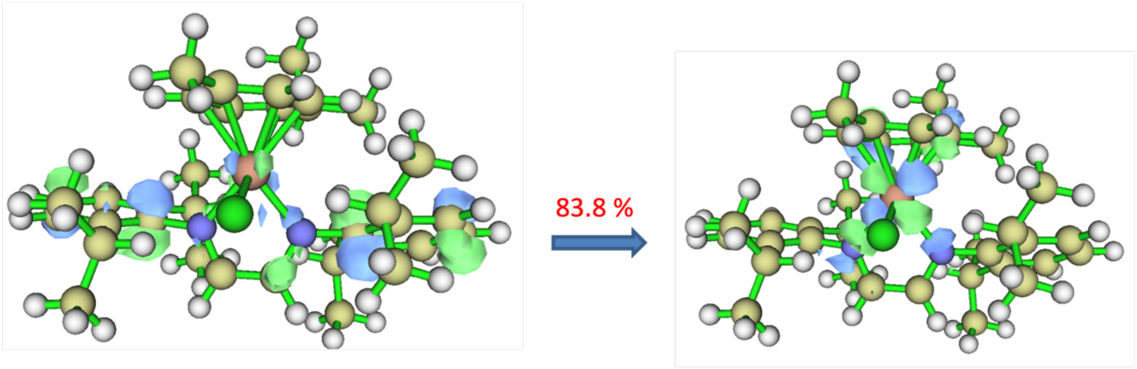

NTO-156 NTO-157

Scheme S.55: 6 Excited state 25 (294.94 nm, f = 0.0417).

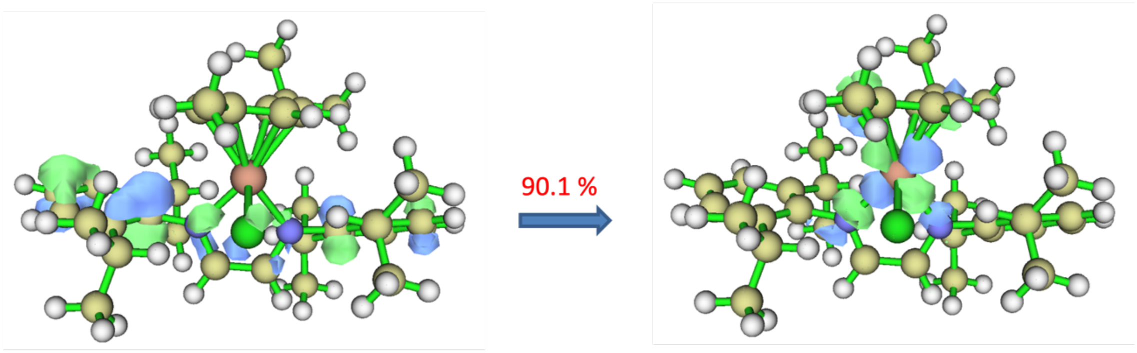

NTO-156 NTO-157

Scheme S. 56: 7 Excited state 22 (303.18 nm, f = 0.0812).

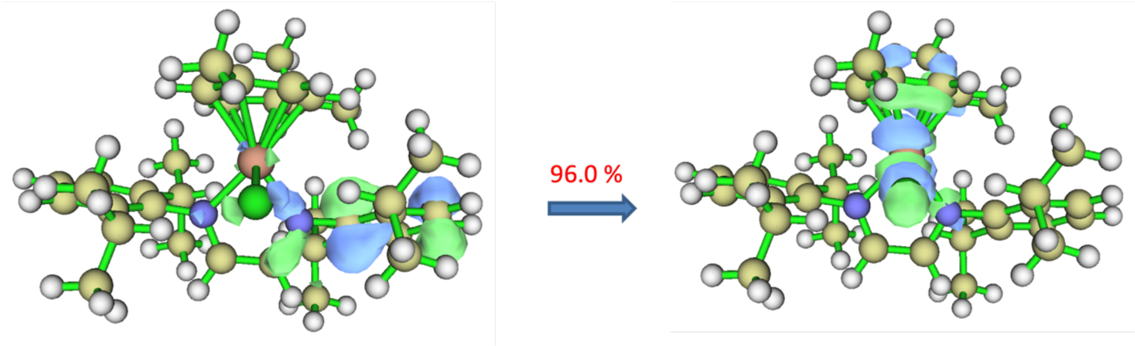

NTO-156 NTO-157

Scheme S. 57: 8 Excited state 19 (331.88 nm, f = 0.0686).

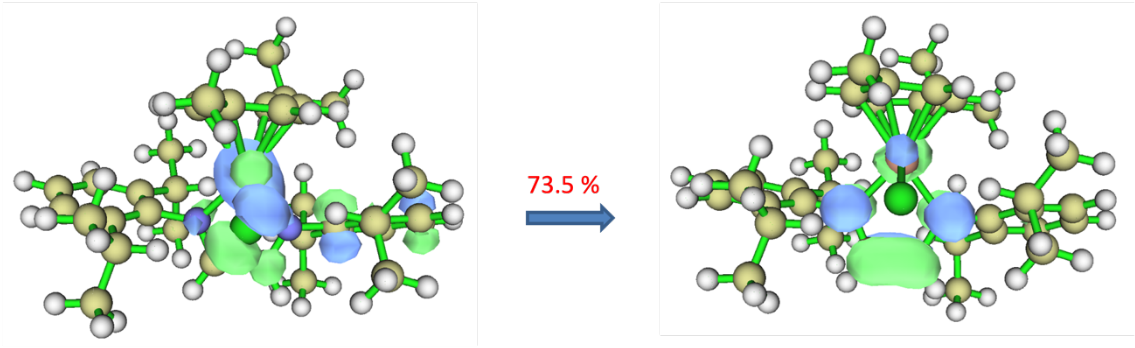

NTO-156 NTO-157

Scheme S. 58: 9 Excited state 10 (410.36 nm, f = 0.0460).

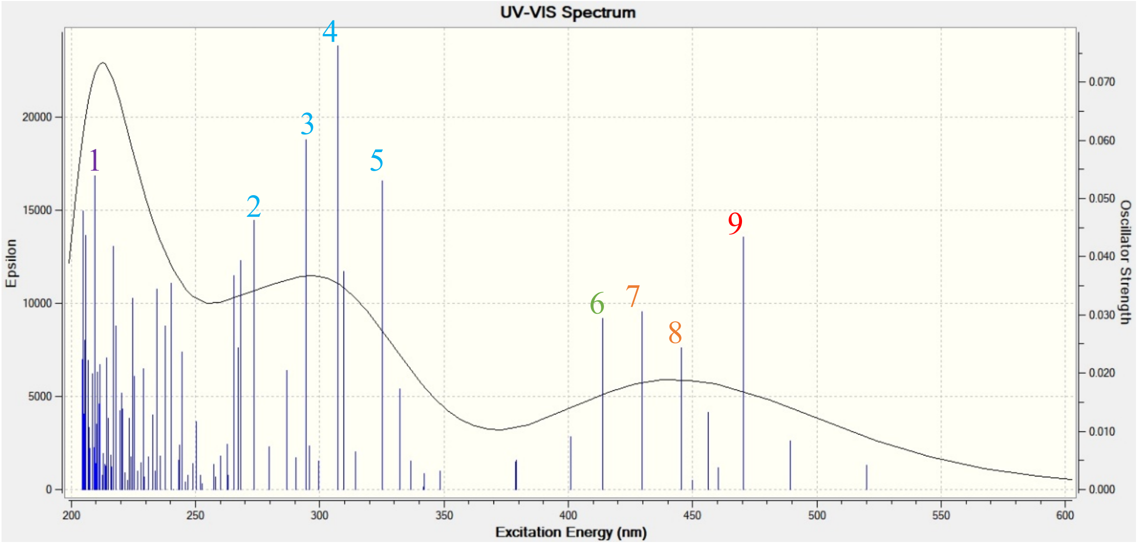

Fig. S 81: UV/vis of optimized structure **4-b** calculated by DTDFT calculation.

Natural Transition Orbitals to describe the observed excited states for structure **4-b**.

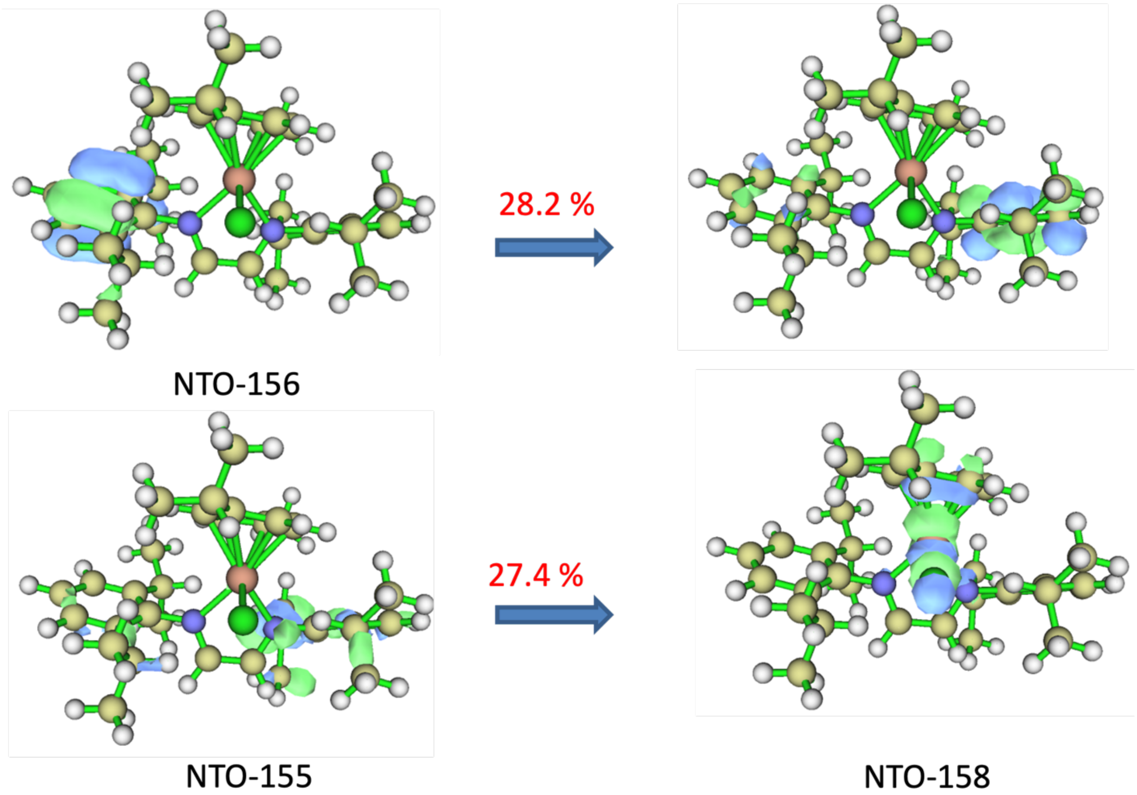

Scheme S.59: 1 Excited state 87 (209.55 nm, f = 0.0539).

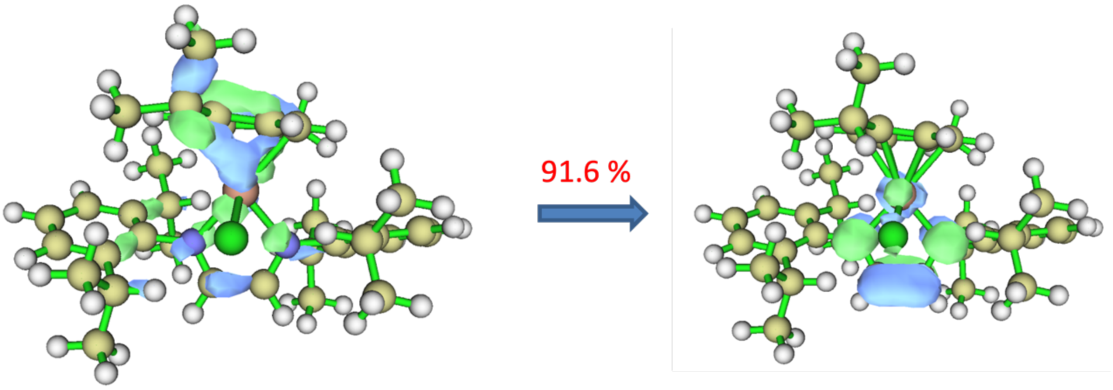

NTO-156 NTO-157

Scheme S.60: 2 Excited state 29 (273.62 nm, f = 0.0462).

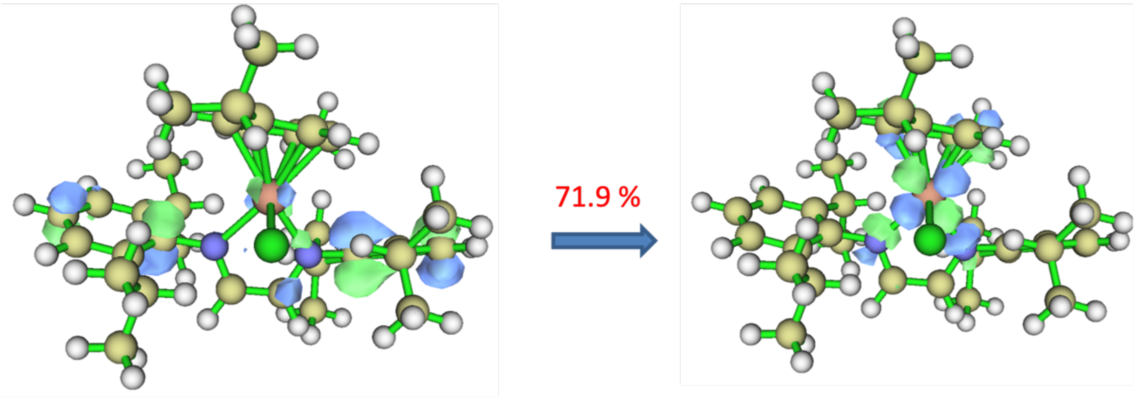

NTO-156 NTO-157

Scheme S.61: 3 Excited state 25 (294.38 nm, f = 0.0601).

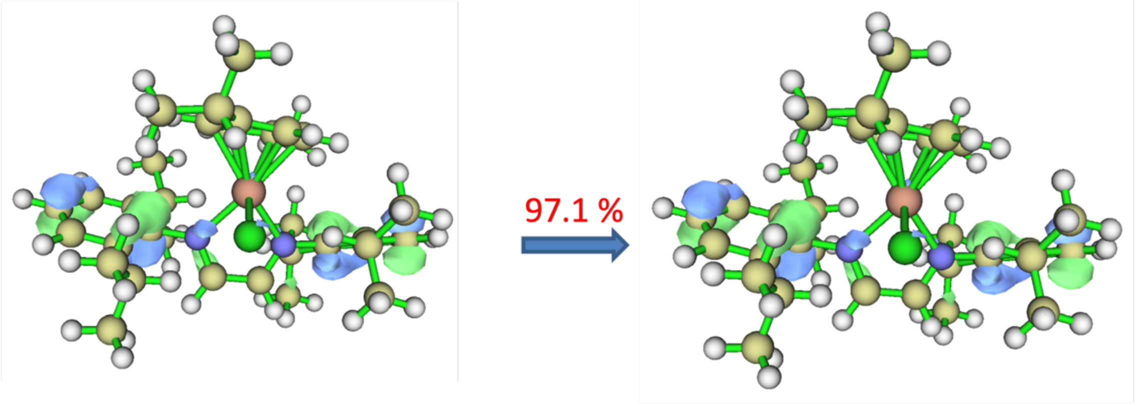

NTO-156 NTO-157

Scheme S.62: 4 Excited state 22(307.43 nm, f = 0.0763).

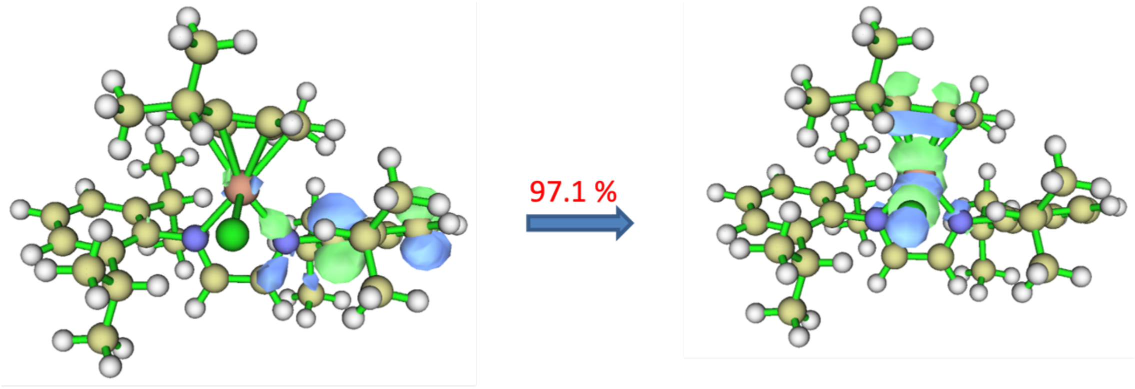

NTO-156 NTO-157

Scheme S. 63: 5 Excited state 19 (324.99 nm, f = 0.0530).

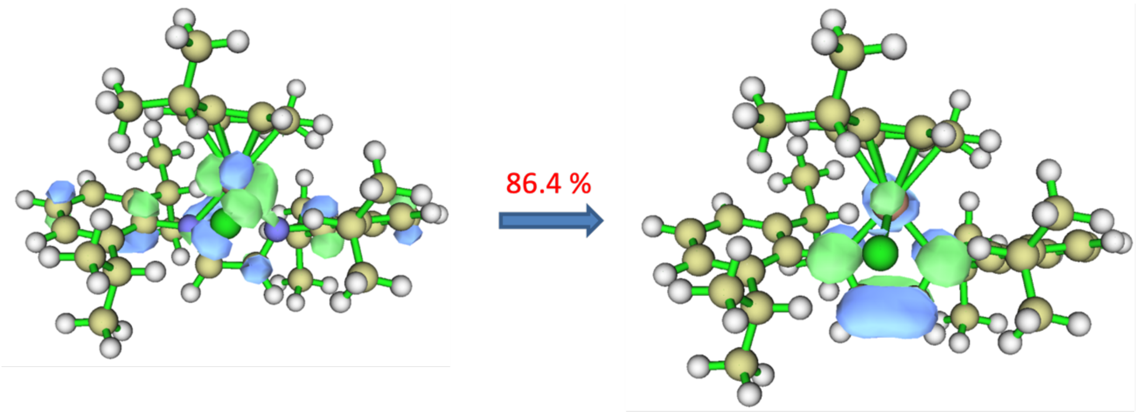

NTO-156 NTO-157

Scheme S.64: 6 Excited state 10 (413.74 nm, f = 0.0294).

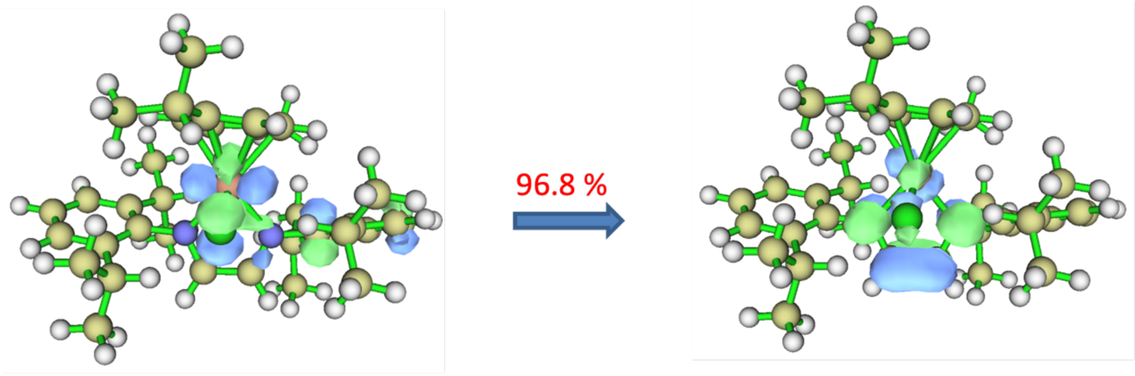

NTO-156 NTO-157

Scheme S.65: 7 Excited state 9 (429.77 nm, f = 0.0305).

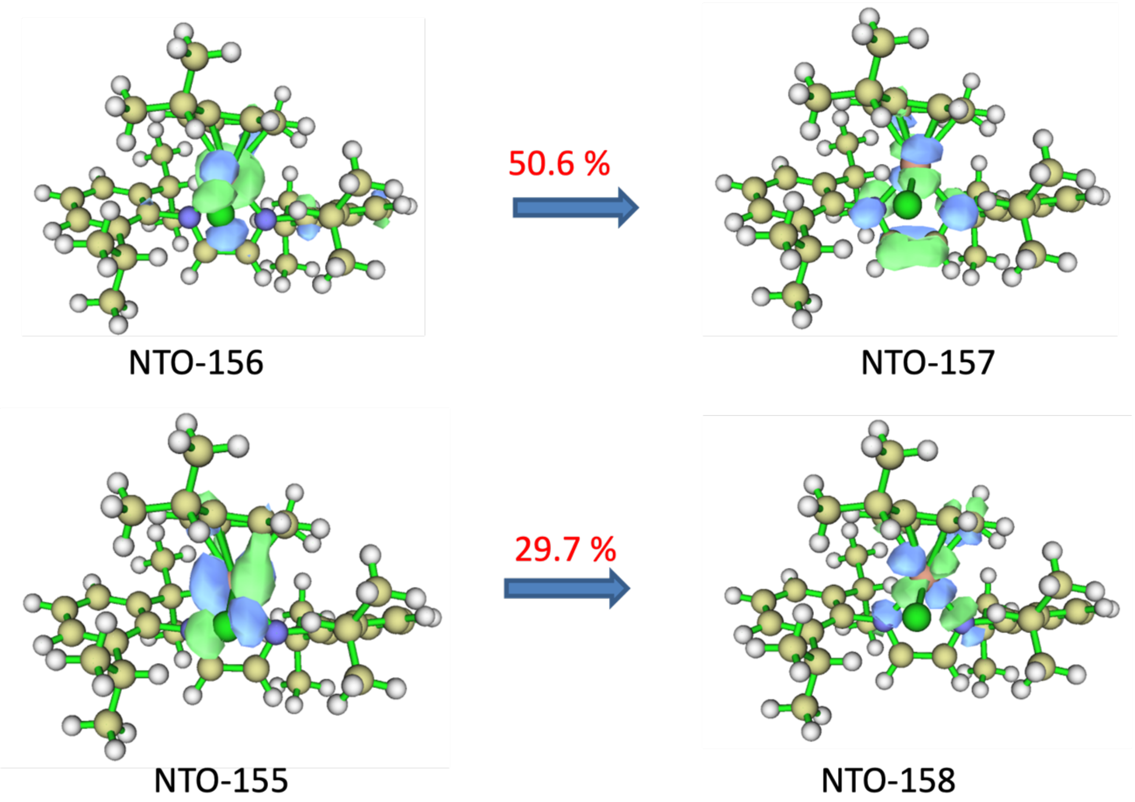

Scheme S. 66: 8 Excited state 8 (445.50 nm, f = 0.0244).

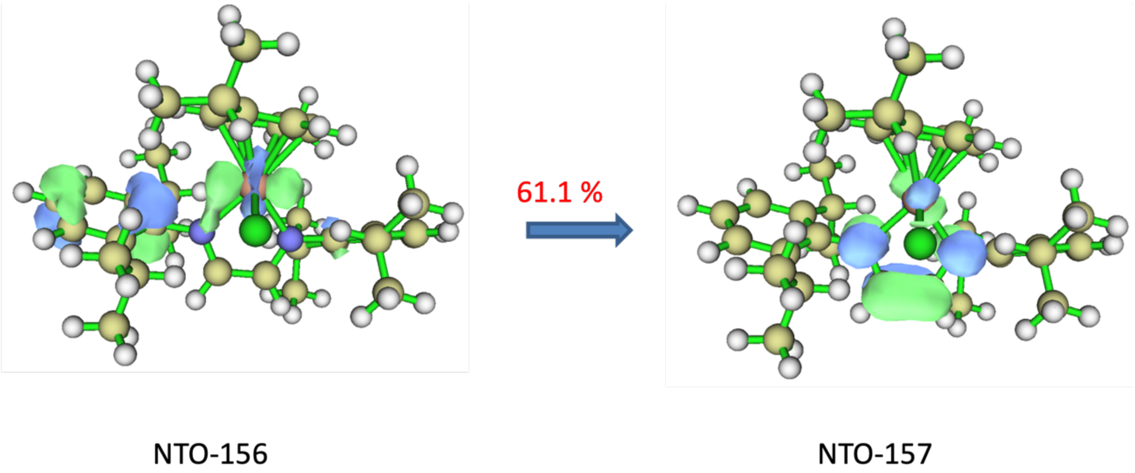

Scheme S.67: 9 Excited state 4 (470.50 nm, f = 0.0434).

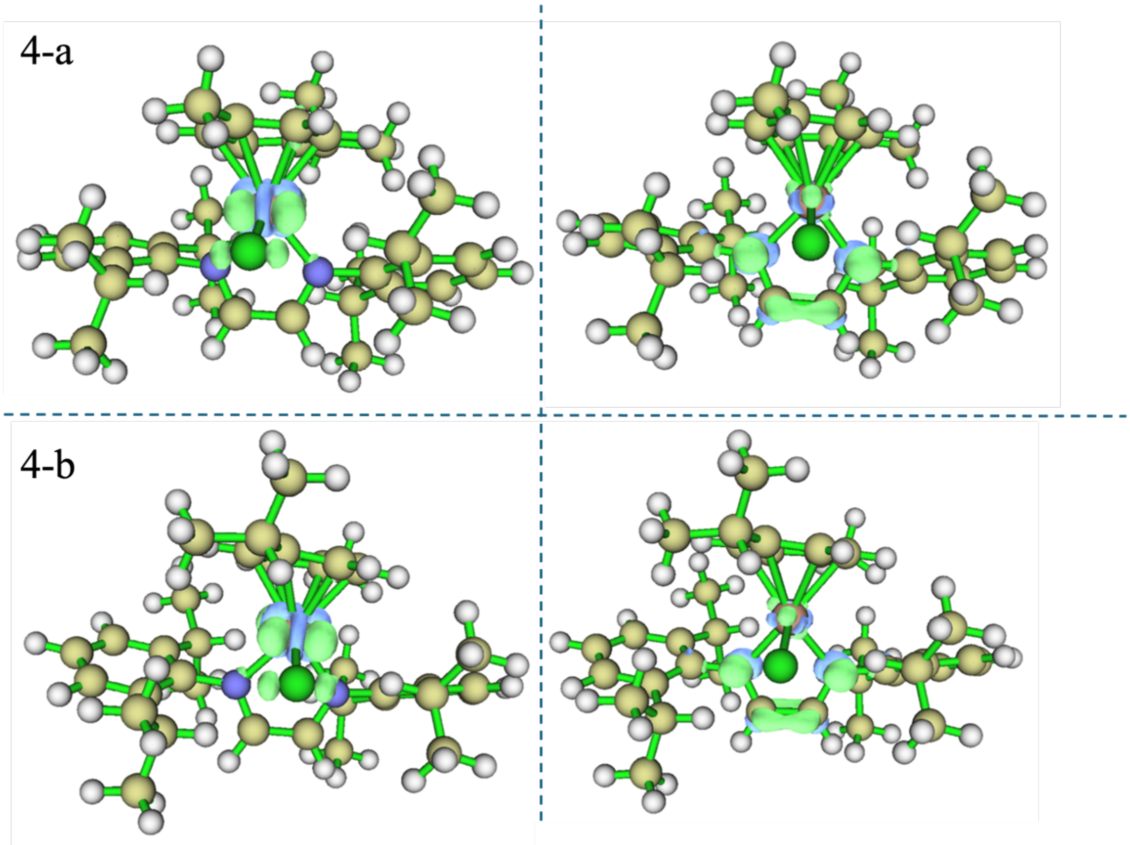

Fig. S.82: Fukui function for the optimized structures **4-a** and **4-b**, *f-* (left) represents an electrophilic attack region and *f+* (right) represents a nucleophilic attack region.

Table S.43: Atomic dipole moment corrected Hirshfeld atomic charges, values of *f+* and *f-* of structure **4-a**

		N (neutral)	N-1 (cation)	N+1 (anion)	*f-*	*f+*
1	Ru	0.357154	0.459961	0.337005	0.10281	0.020149
2	Cl	-0.300689	-0.153920	-0.368923	0.14677	0.068234
3	N	-0.055415	-0.039608	-0.125965	0.01581	0.070550
4	C	0.072389	0.106382	-0.011190	0.03399	0.083579
5	C	0.078532	0.109897	-0.006596	0.03137	0.085128
7	N	-0.051978	-0.034738	-0.121065	0.01724	0.069087
8	C	0.031252	0.030629	0.035766	-0.00062	-0.004514
9	C	-0.000201	0.004032	-0.005145	0.00423	0.004944
10	C	0.006069	0.015633	-0.000357	0.00956	0.006426
11	C	-0.043795	-0.029100	-0.056258	0.01470	0.012463
12	C	-0.041068	-0.030187	-0.053394	0.01088	0.012326
13	C	-0.036406	-0.017054	-0.054155	0.01935	0.017749
17	C	-0.020225	0.001336	-0.036475	0.02156	0.016250

18	C	0.027891	0.049378	0.011780	0.02149	0.016111
19	C	-0.020357	0.011756	-0.042432	0.03211	0.022075
20	C	-0.012861	0.009593	-0.033491	0.02245	0.020630
21	C	0.042080	0.067336	0.022799	0.02526	0.019281
23	C	-0.020921	0.017396	-0.041951	0.03832	0.021030
27	C	-0.066048	-0.055607	-0.073717	0.01044	0.007669
31	C	-0.001265	0.002147	-0.003210	0.00341	0,001945
33	C	-0.081980	-0.078657	-0.084563	0.00332	0.002583
37	C	-0.076382	-0.069955	-0.081312	0.00643	0.004930
42	C	0.035145	0.036427	0.037377	0.00128	-0.002232
43	C	0.006687	0.013765	-0.001615	0.00708	0.008302
44	C	0.006989	0.013329	-0.001516	0.00634	0.008505
45	C	-0.038841	-0.025739	-0.052537	0.01310	0.013696
46	C	-0.039562	-0.028627	-0.053543	0.01094	0.013981
47	C	-0.030604	-0.009347	-0.053423	0.02126	0.022819
51	C	-0.020907	-0.019816	-0.021454	0.00109	0.000547
52	C	-0.082893	-0.080253	-0.086286	0.00264	0.003393
53	C	-0.084927	-0.080978	-0.088367	0.00395	0.003440
61	C	-0.012951	-0.012222	-0.013475	0.00073	0.000524
62	C	-0.083169	-0.080304	-0.086654	0.00287	0.003485
63	C	-0.081793	-0.078795	-0.085788	0.00300	0.003995
71	C	-0.014736	-0.015016	-0.013584	-0.00028	-0.001152
72	C	-0.081533	-0.078996	-0.085149	0.00254	0.003616
73	C	-0.083034	-0.080093	-0.085927	0.00294	0.002893
81	C	-0.020365	-0.016408	-0.020921	0.00396	0.000556
82	C	-0.084596	-0.080121	-0.087606	0.00448	0.003010
83	C	-0.084563	-0.081390	-0.087876	0.00317	0.003313

Table S.44: Atomic dipole moment corrected Hirshfeld atomic charges, values of *f*+ and *f*- of structure **4-b**

		N (neutral)	N-1 (cation)	N+1 (anion)	*f*-	*f*+
1	Ru	0.355704	0.465562	0.337090	0.10986	0.018614
2	Cl	-0.303938	-0.152218	-0.368466	0.15172	0.064528
3	N	-0.052681	-0.038372	-0.123293	0.01431	0.070612
4	C	0.07359	0.109211	-0.012030	0.03562	0.085620
5	C	0.07961	0.108165	-0.005698	0.02856	0.085308
7	N	-0.051473	-0.034602	-0.122244	0.01687	0.070771
8	C	0.031864	0.030357	0.035297	-0.00151	-0.003433
9	C	0.000728	0.004865	-0.006426	0.00414	0.007154
10	C	0.007122	0.014527	-0.000748	0.00741	0.007870
11	C	-0.043728	-0.031713	-0.057049	0.01202	0.013321
12	C	-0.041034	-0.031145	-0.053826	0.00989	0.012792
13	C	-0.035672	-0.017648	-0.055823	0.01802	0.020151
17	C	-0.019454	0.007156	-0.037564	0.02661	0.018110
18	C	0.027776	0.049884	0.012048	0.02211	0.015728
19	C	-0.018567	0.012957	-0.039170	0.03152	0.020603
20	C	-0.014943	0.009662	-0.036468	0.02461	0.021525
21	C	0.036264	0.063522	0.019256	0.02726	0.017008
23	C	-0.018194	0.014602	-0.037750	0.03280	0.019556
26	C	-0.003474	0.000730	-0.006193	0.00420	0.002719
29	C	0.033875	0.033596	0.036865	-0.00028	-0.002990
30	C	0.005588	0.011907	-0.002053	0.00632	0.007641
31	C	0.00598	0.011678	-0.001601	0.00570	0.007581
32	C	-0.040808	-0.027702	-0.054490	0.01311	0.013682
33	C	-0.040127	-0.029300	-0.053931	0.01083	0.013804
34	C	-0.03228	-0.012003	-0.053990	0.02028	0.021710
38	C	-0.064786	-0.055460	-0.070892	0.00933	0.006106
42	C	-0.076116	-0.068069	-0.081102	0.00805	0.004986
46	C	-0.079139	-0.075267	-0.081894	0.00387	0.002755
51	C	-0.012793	-0.012606	-0.012599	0.00019	-0.000194
52	C	-0.083254	-0.080364	-0.086695	0.00289	0.003441
53	C	-0.082493	-0.079613	-0.086482	0.00288	0.003989
61	C	-0.014685	-0.014474	-0.014347	0.00021	-0.000338

62	C	-0.081301	-0.078782	-0,085017	0.00252	0.003716
63	C	-0.083096	-0.079939	-0,086158	0.00316	0.003062
71	C	-0.018993	-0.016291	-0,019202	0.00270	0.000209
72	C	-0.084703	-0.080476	-0,087996	0.00423	0.003293
73	C	-0.085746	-0.082772	-0,089200	0.00297	0.003454
81	C	-0.020903	-0.019689	-0,021408	0.00121	0.000505
82	C	-0.082883	-0.080303	-0,086311	0.00258	0.003428
83	C	-0.084314	-0.080413	-0,087649	0.00390	0.003335

Kinetic

This section documents the calculation of kinetic parameters for complex **1** in the solvent-free dehydrogenation of formic acid at 40 – 60 °C, using the standard molar ratio Ru/FA/NEt_3 = 1/1204/843. It also describes how fractional conversion (*X*), turnover frequency (TOF), and observed rate constants (k_{obs}) were derived and used to make the kinetic tables, and how activation parameters were obtained from Arrhenius and Eyring analyses. Detailed results are present here, with replicate-level data and calculation steps provided for readers. Induction periods were consistently observed in the first runs, as discussed in the main manuscript (See Fig.S.83). Because this interval represents an important step in the activation pre-catalysts into the active catalytic species, the conversion rate at the end-time was adopted for calculating the k_{obs}. This procedure provides a general kinetic description that inherently accounts for the induction period. A validation of the end-point method, using induction-growth kinetic model (X_{model}), is described at the end of this section.

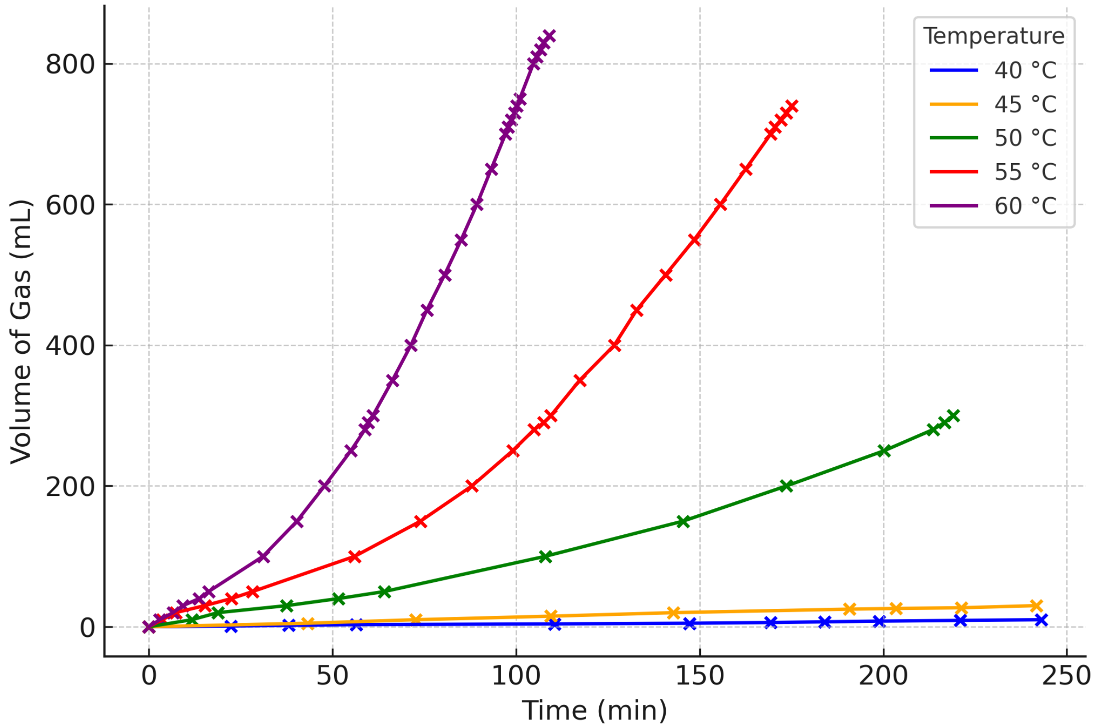

Fig. S.83: Kinetic curves within the temperature range of 40 – 60°C.

Definitions and symbols

- **V_d** = Gas volume displaced in the water column during a run (mL).
- **V_T** = Maximum gas volume expected for 100% conversion under our setup. Here, V_T = 896 mL.
- ***X*** = Fractional conversion (0 – 1). When reported as %, $X \times 100$.
- **t** = Elapsed time at which conversion is read (min).
- ***k_{obs}*** = Observed rate constant (min^{-1} or s^{-1}) obtained under excess substrate/base.
- **Replicate handling** — All entries come from triplicate runs at each temperature; averages are given with standard deviations (SD) where indicated.

Note on induction. Each kinetic run shows an induction period. The point estimate method below uses the pair (*X*, t) for each replicate to extract k_{obs} via the integrated expression. This yields a consistent comparative metric across temperatures and matches the values used in this section. The conversion values were obtained by averaging 3 experiments, and the results are presented in Table S.45.

Table S. 45: Conversion *vs* time.

Temp. (°C)	Conversion (%)			Average	SD	Total conversion time		
40	1.12	1.12	1.12	1.12	0.00	240	244	245
45	3.35	2.79	2.79	2.98	10.83	240	241	244
50	33.48	31.81	32.37	32.55	2.62	216	220	221
55	82.59	79.24	80.91	80.92	2.07	175	177	173
60	94.87	95.98	93.75	94.87	1.18	110	106	111

Equations

- **Conversion from displaced volume**

$$X = \frac{V_d}{V_T}\ (V_T = 896\,mL) \quad \textbf{Eq.(1)}$$

$$\%\,Conv. = X \times 100 \quad \textbf{Eq.(2)}$$

- **Observed rate constant (k_{obs})**

Assuming $\frac{dX}{dt} = k_{obs}\,(1 - X)$,with $X(0) = 0$, it provides:

$$\frac{dX}{1 - X} = k_{obs}\,dt$$

$$\int_0^{X(t)} \frac{dX}{1 - X} = \int_0^t k_{obs}\,dt$$

Evaluate the integrals:

- Left side: $\int \frac{dX}{1-X} = -\ln(1-X)$
- Right side: $\int k_{obs}\, dt = k_{obs}\, t$
- Applying the limits: $-\ln(1-X(t)) + \ln(1-0) = k_{obs}\, t$; {ln(1-0) = 0}
- Rearrange to the linear form: $\ln(1-X) = -k_{obs}\, t$
- Solve for k_{obs}:

$$k_{obs} = -\frac{\ln(1-X)}{t} \qquad \textbf{Eq.(3)}$$

Notes: This result holds for $0 \leq X < 1$; k_{obs} has units of time^{-1} (*e.g.*, min^{-1} or s^{-1}).

Worked example (50°C)

Triplicates recorded from the Table S. 45.

- Times (t) = 216, 220 and 221 min.
- Conversion (%) = 33.48, 31.81 and 32.37
- Fractions = 0.3348, 0.3181 and 0.3237

Compute per replicate using Eq.(3):

- Rep 1: $k_{obs} = 1.887 \times 10^{-3}$ min^{-1} = 18.87×10^{-4} min^{-1}
- Rep 2: $k_{obs} = 1.740 \times 10^{-3}$ min^{-1} = 17.40×10^{-4} min^{-1}
- Rep 3: $k_{obs} = 1.769 \times 10^{-3}$ min^{-1} = 17.69×10^{-4} min^{-1}
- Average (50°C): $k_{obs} = 17.99 \times 10^{-4}$ min^{-1}

Table S.46: Values of the k_{obs} obtained within temperature range 40 – 60 °C.

Temp. (°C)	Conversion (%) Replicates			Fractional Conversion (X) Replicates			Time (min)			k_{obs} (× 10^{-4} min^{-1}) Replicates			Average k_{obs} (× 10^{-4} min^{-1})
40	1.16	1.12	1.12	0.0112	0.0112	0.0112	240	244	245	0.46	0.46	0.46	0.46
45	3.35	2.79	2.79	0.0335	0.0279	0.0279	240	241	244	1.42	1.17	1.16	1.25
50	33.48	31.81	32.37	0.3348	0.3180	0.3237	216	220	221	18.87	17.40	17.69	17.99
55	82.59	79.24	80.91	0.8259	0.7924	0.8091	175	177	173	99.89	88.82	95.74	94.81
60	94.87	95.98	93.75	0.9487	0.9598	0.9375	110	106	111	269.94	303.25	249.78	274.32

Arrhenius and Eyring plots.

Activation parameters were obtained from Arrhenius and Eyring analyses using average k_{obs} in the Table S.46, measured at 313 – 333 K. Linear fits of ln k_{obs} vs 1/T and ln(k_{obs}/T) *vs* 1/T afforded E_a = 296.83 k*J* mol^{-1} and $\Delta H^‡$ = 294.12 k*J* mol^{-1}, respectively. The Eyring intercept yielded $\Delta S^‡$ = + 575.16 *J* mol^{-1} K^{-1} (kith k_{obs} in s^{-1}). $\Delta G^‡$ was calculated as $\Delta G^‡ = \Delta H^‡ - T\Delta S^‡$, at 60°C (*e.g.*, 102.49 k*J* mol^{-1}).

Arrhenius (using ln k_{obs} *vs* 1/T)

Form:

$$\ln k = \ln A - \frac{E_a}{R}\frac{1}{T} \quad \textbf{Eq. (4)}$$

Fitted line from data:

$$\ln k = -35702\,\frac{1}{T} + 103.83$$

Goodness of fit: R^2 = 0.9776.

Table S.47: Values used to construct the Arrhenius plot.

Entry	T (°C)	1/T (K)	lnk_{obs} (min^{-1})
1	40	0.003193358	-9.982730826
2	45	0.003143171	-8.986407721
3	50	0.003094538	-6.320487168
4	55	0.003047387	-4.658382552
5	60	0.003001651	-3.596037347

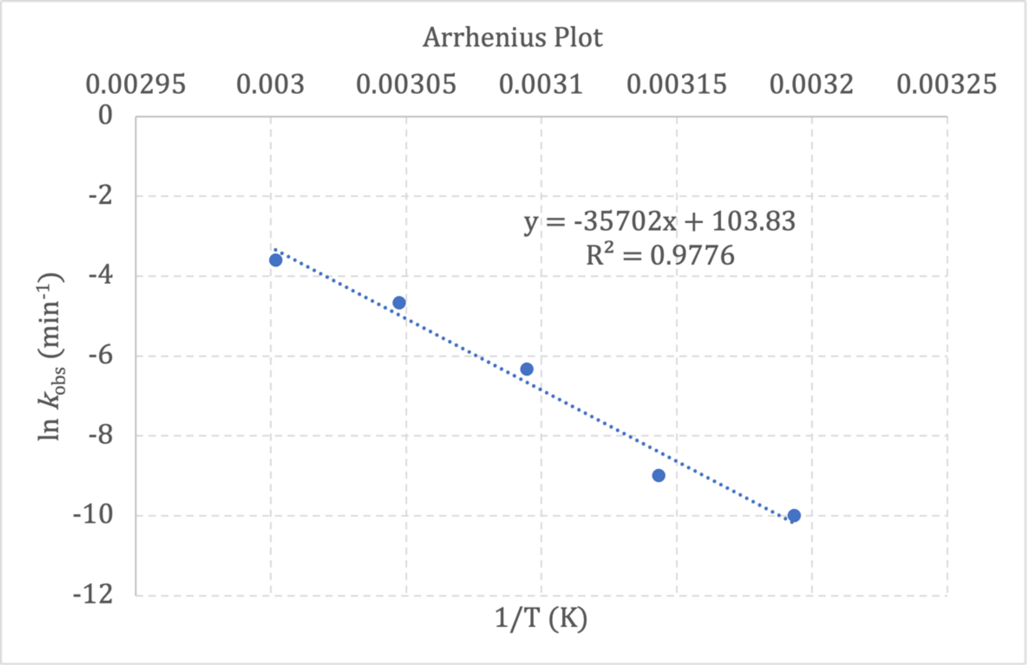

Fig. S.84: Arrhenius plot; ln k_{obs} (min^{-1}) *vs* 1/T (K).

Eyring (using ln (k_{obs}/T) *vs* 1/T).

Form (Eyring - Polanyi, natural log):

$$\ln\frac{k}{T} = \ln\frac{k_B}{h} + \frac{\Delta S^{\ddagger}}{R} - \frac{\Delta H^{\ddagger}}{R}\frac{1}{T} \quad \textbf{Eq. (5)}$$

Fitted line from data:

$$\ln\frac{k}{T} = -35379\frac{1}{T} + 92.936$$

Goodness of fit: $R^2 = 0.9773$.

Notes: R = 8.314 J mol^{-1} K^{-1}; to use k_B/h, so for Eyring you must either convert k to s^{-1} or subtract ln 60 from the interceptS if you fit with k_{obs} in min^{-1}.

Table S.48: Values used to construct the Eyring plot.

Entry	Temperature (°C)	1/T (K)	lnk_{obs}/T (s^{-1}/K)
1	40	0.00319336	-19.823558
2	45	0.00314317	-18.840869
3	50	0.00309454	-16.193097
4	55	0.00304739	-14.546386
5	60	0.00300165	-13.498982

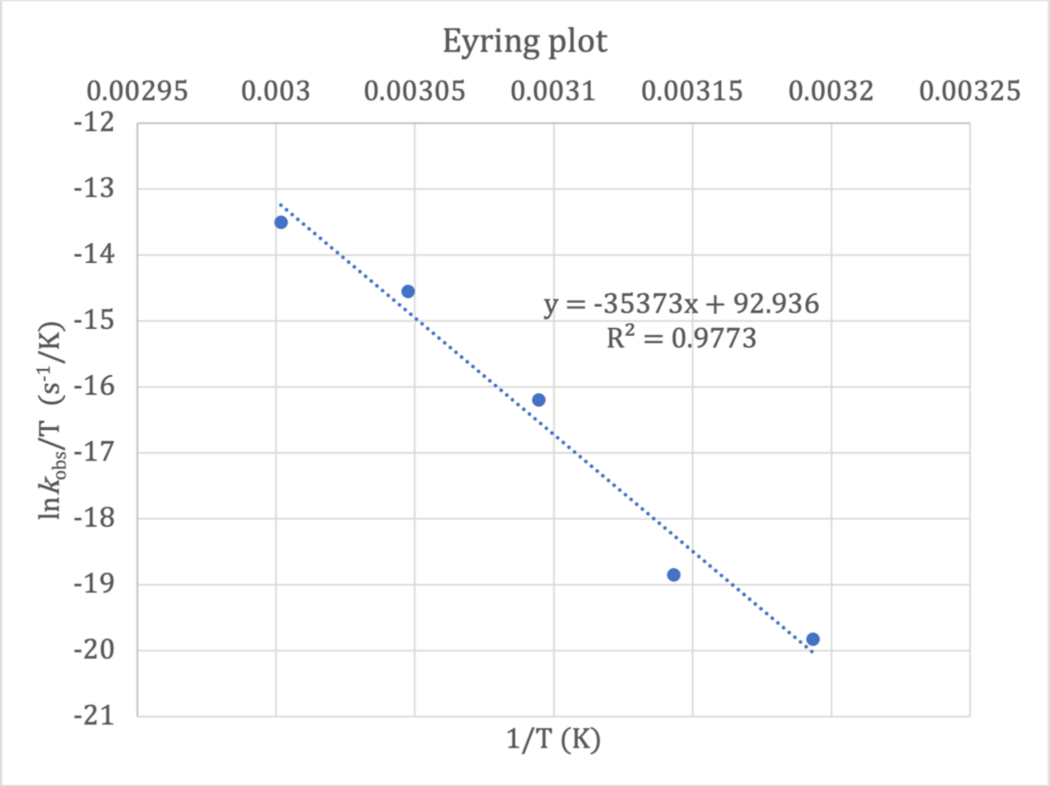

Fig. S.85: Eyring plot, lnk_{obs}/T (s^{-1}/K) *vs* 1/T (K).

Step-by-step: how to obtain each parameter

(A) Activation energy E_a (Arrhenius)

1. Compute x = 1/T and y = ln k_{obs} (with k_{obs} in any time^{-1} unit, here it was min^{-1},

2. Fit y = mx + b

3. Use $m = -E_a/R$; *e.g.* $E_a = -mR$

From the data: $m = -35702$ K, *e.g.* $\mathbf{E_a = 296.83}$ **k*J* mol^{-1}**

(B) Enthalpy of activation $\Delta H^{\ddagger}$ (Eyring)

1. Convert k_{obs} to s^{-1}

2. Compute x = 1/T and y = ln(k_{obs}/T)

3. Fit y = mx + b

4. Use $m = -\Delta H^{\ddagger}/R$; *e.g.* $\Delta H^{\ddagger} = -mR$

From the data: $m = -35379$ K

$\Delta H^{\ddagger} = -(-35373) \times 8.314462618$

$\mathbf{\Delta H^{\ddagger} = 294.107}$ **k*J* mol^{-1}**

(C) Entropy of activation $\Delta S^{\ddagger}$ (Eyring)

1. From the same Eyring fit, take the intercept b.

2. Use $b = \ln(k_B/h) + \Delta S^{\ddagger}/R$ (valid when k_{obs} was in s^{-1}).

3. Rearrange: $\Delta S^{\ddagger} = R\,[b - \ln(k_B/h)]$

From the data:

- b = 92.936
- R = 8.314462618 *J* mol^{-1} K^{-1}
- k_B = Boltzmann constant, 1.380649×10^{-23} *J* K^{-1}
- h = Planck constant, $6.62607015 \times 10^{-34}$ *J* s

ln(k_B/h) = 23.759977810504218

$\Delta S^{\ddagger} = 8.314462618 \times (92.936 - 23.7599778105)$

$\mathbf{\Delta S^{\ddagger} = 575.161}$ ***J* mol^{-1} K^{-1}**

(D) Gibbs free energy of activation $\Delta G^{\ddagger}$ (from Gibbs).

- $\Delta G^{\ddagger}$ at 60°C

1. Using $\Delta G^{\ddagger}(T) = \Delta H^{\ddagger} - T\Delta S^{\ddagger}$

2. With $\Delta H^{\ddagger}$ = 294.107 k*J* mol^{-1} and $\Delta S^{\ddagger}$ = 575.161 *J* mol^{-1} K^{-1} (or 0.575161 k*J* mol^{-1} K^{-1}),

3. T = at 333.15 K

$\Delta G^{\ddagger}$(333.15 K) = 294.107 – (333.15 × 0.575161)

$\Delta G^{\ddagger}$ = 102.492 k*J* mol^{-1}.

Table S.49: Summary of the activation parameters based on Arrhenius and Eyring plots.

$E_a{}^{a}$		$\Delta H^{\ddagger b}$		$\Delta S^{\ddagger\, b}$		$\Delta G^{\ddagger}$	
k*J* mol^{-1}	kcal mol^{-1}	k*J* mol^{-1}	kcal mol^{-1}	*J* mol^{-1}K^{-1}	cal mol^{-1}K^{-1}	k*J* mol^{-1}	kcal mol^{-1}
296.83	70.94	294.12	70.29	+575.16	+137.47	102.49	24.50

a = from Arrhenius plot. *b* = from Eyring plot.

Induction–growth kinetic model (X_{model})

It can be viewed as a validation of the end-point method. While the end-point approach yields a conservative observed rate constant (k_{obs}) that includes the induction period, the induction–growth model explicitly separates the induction time (τ) from the pseudo–first-order growth constant (k). The close agreement between the two approaches confirms that the end-point treatment provides reliable kinetic parameters for Arrhenius and Eyring analysis, even when induction times are present.

The evolution of gas (H_2 + CO_2) was monitored by water displacement; conversion was defined as $X=V_d/V_T$ (V_T = 896 mL, corresponding to 100% conversion). The first run at each temperature (40 – 60 °C) did not reach full conversion and showed a visible induction period. Data were fitted to a delay-plus-first-order model, labeled here as X_{model}. This model is defined by two regimes depending on t and the τ, as follows:

$$X(t) = \begin{cases} 0, & t \leq \tau \\ 1 - e^{[-k(t-\tau)]}, & t > \tau \end{cases} \qquad \textbf{Eq. (6)}$$

by minimizing $\sum_i[X_i - X_{model}(t_i;\tau,k)]^2$ in Excel (Solver; GRG Nonlinear; bounds $0 \leq \tau \leq$ a reaction duration time, and $0 < k \leq 1$ min^{-1}).

Data at 60°C (values from 1st run in the Fig. 8 in the main text) .

Table S.50: Values obtained from Excel-Solver using X_{model} at 60°C.

Time (min)	V_d(60°C)	X=V_d/V_T	X_{model}	τ (min)	k (min^{-1})	RSS
0	0	0	0	**39.2831564**	**0.0247981**	**0.06590337**
15	30	0.03348214	0			
30	100	0.11160714	0			
45	180	0.20089286	0.132176458			
60	300	0.33482143	0.401745141			
75	420	0.46875	0.587578743			
90	600	0.66964286	0.715687569			
105	820	0.91517857	0.804002444			
110.3	850	0.94866071	0.828141366			

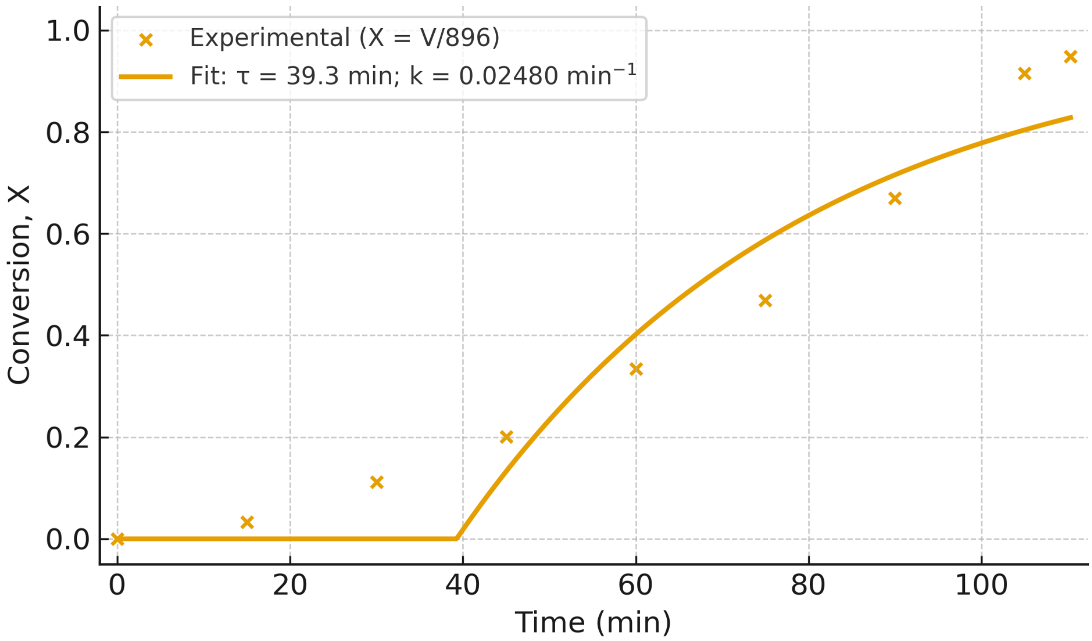

Fig. S.86: Experimental conversion (points from Fig. 8 main text) and the fitted curve (line) results at 60°C using induction-growth kinetic model (complex **1** as pre-catalyst for the first run).

The best-fit parameters at 60°C were:

- τ =39.28 min
- k = 0.02480 min^{-1} (4.133×10^{-4} s^{-1})

with a residual sum of squares (RSS) of 0.0659. Fig. S.86 shows experimental conversion points (X = Vd/896) and the fitted curve (time *vs* X_{model}) . The fitted growth constant is

consistent with end-point estimates $k_{obs} = -\ln(1-X) / t$, indicating that the end-point method provides a conservative bound while the X_{model} explicitly separates induction and growth.

Step-by-step how to use the Induction–growth model (X_{model}) in the Excel.

Objective**:** Extract the induction time τ (min) and the pseudo–first-order growth constant k (min^{-1}) from time–conversion data of the solvent-free dehydrogenation of formic acid.

1) Data and normalization

1. Record time t (min) and displaced volume V(mL) of the evolved gases (H_2 + CO_2).
2. Define the conversion as

   $X = V_d / V_T$,

   where V_T is the volume corresponding to 100% conversion (experiment-specific; report the value used, here it was 896 mL). Clip X to [0, 1] if needed.

Excel (English locale)

- Column A: Time (min) → t_i
- Column B: Volume (mL) → V_i
- Column C (Conversion):`=B2/$B$max_value` (replace with your V_T cell or number)

2) Model definition

Model the conversion with an induction delay followed by pseudo–first-order growth:

$$X(t) = \begin{cases} 0, & t \le \tau \\ 1 - e^{[-k(t-\tau)]}, & t > \tau \end{cases}$$

Parameters: τ (min), k (min^{-1}).

Excel (English locale)

- Reserve two cells for the parameters (*e.g.*, **E2** = τ; **F2** = k).
- Column D (X_{model}):
- Excel code:

```
=IF(A2<=$E$2, 0, 1-EXP(-$F$2*(A2-$E$2)))
```

Fill down to the last data row. (Use absolute references `$E$2`, `$F$2` so all rows use the same τ, k.)

3) Objective function (least squares)

Minimize with a residual sum of squares (RSS):

$$RSS(\tau, k) = \sum_i [X_i - X_{model}(t_i; \tau, k)]^2$$

Excel (English locale)

- In a separate cell (*e.g.*, **G2**):
- Excel code:

```
=SUM((C2:Cn - D2:Dn)^2)
```

Replace "`n`" with the last row of data.

4) Optimization in Excel (Solver)

- **Data → Solver**
  - *Set Objective*: the RSS cell (*e.g.*, `G2`).
  - *To*: Min.
  - *By Changing Variable Cells*: `E2:F2` (τ, k).
  - *Subject to the Constraints*:
    - `E2 >= 0` (τ non-negative)
    - `F2 > 0` and, for stability, `F2 <= 1` (min^{-1})
  - *Solving Method*: GRG Nonlinear**.**
  - *Initial guesses*: *e.g.*, `E2 = 20` min, `F2 = 0.02` min^{-1}.
  - Click **Solve** → **Keep Solver Solution**. (The appearance of the results table is similar to the Table S.50)

5) Visual validation

Plot X (column C) and X_{model} (column D) *vs* time in one chart (Example = Fig. S 86). A good fit exhibits:

(i) a flat region up to $t \approx \tau$, and
(ii) a smooth rise captured by the exponential for $t > \tau$.

Induction – growth kinetic model applied in all temperature curves of Fig. S.83.

This section presents the kinetic analyses performed with the induction–growth model (X_{model}), applied to the solvent-free dehydrogenation of formic acid catalyzed by complex **1** in the first run at 40 – 60 °C (Fig. S.83). The fits were obtained using the Excel-Solver routine, which allowed simultaneous optimization of the induction time (τ) and the pseudo–first-order growth constant (k). The results are reported below for each temperature, including the comparison between experimental conversion data and the model curves.

Table S.51: Values obtained from Excel-Solver using X_{model} at **60ºC**.

Time (min)	V_d (60°C)	$X=V_d/V_T$	X_{model}	τ (min)	k (min^{-1})	RSS
0	0	0	0	**44.5391216**	**0.02836372**	**0.14419807**
2.97	10	0.01116071	0			
6.59	20	0.02232143	0			
9.31	30	0.03348214	0			
13.69	40	0.04464286	0			
16.28	50	0.05580357	0			
31.1	100	0.11160714	0			
40.34	150	0.16741071	0			
47.84	200	0.22321429	0.089376			
55.03	250	0.27901786	0.25737224			
58.83	280	0.3125	0.33325159			
59.74	290	0.32366071	0.35024083			
61.03	300	0.33482143	0.37358531			
66.33	350	0.390625	0.46101655			
71.37	400	0.44642857	0.53281202			
75.72	450	0.50223214	0.5870405			
80.55	500	0.55803571	0.63991045			
85.08	550	0.61383929	0.68332846			
89.35	600	0.66964286	0.71944999			
93.23	650	0.72544643	0.7486866			
97.14	700	0.78125	0.7750679			
97.82	710	0.79241071	0.77936467			
98.64	720	0.80357143	0.78443704			
99.56	730	0.81473214	0.78998932			
100.21	740	0.82589286	0.79382569			
101.01	750	0.83705357	0.79845131			
104.73	800	0.89285714	0.81863384			
105.64	810	0.90401786	0.82325519			
106.67	820	0.91517857	0.82834402			
107.58	830	0.92633929	0.83271794			
109	840	0.9375	0.8393216			

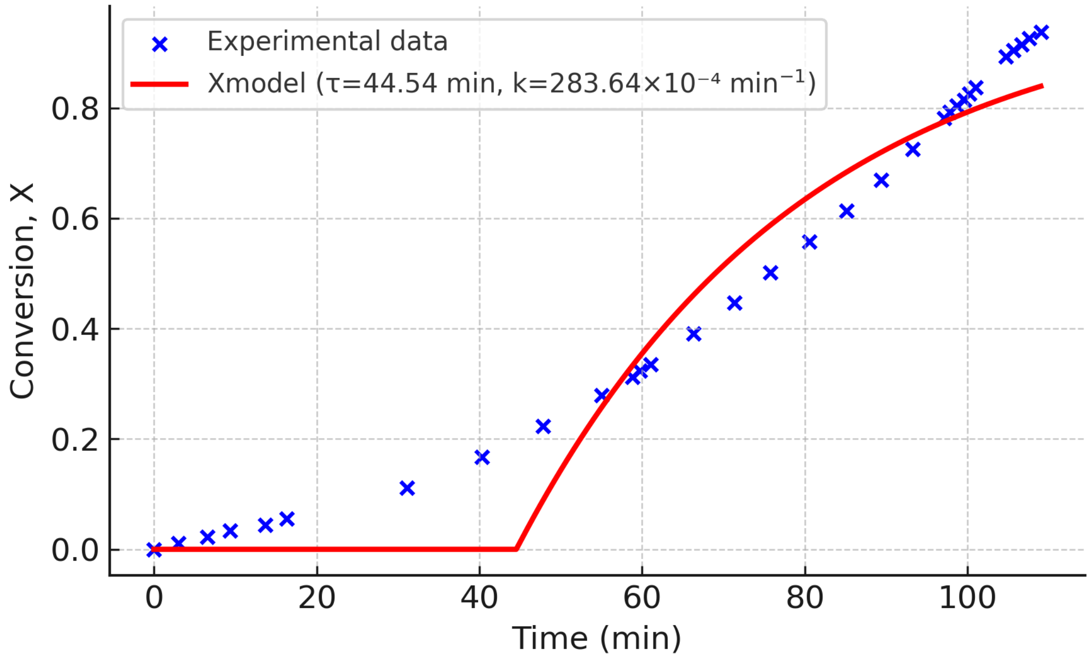

Fig. S.87: Experimental conversion data (blue points, from Table S.51 and Fig. S.83) and the fitted curve (red line) at **60 °C**, obtained using the X_{model}. Complex **1** was employed as the pre-catalyst in the first run.

Table S.52: Values obtained from Excel-Solver using X_{model} at **55ºC**.

Time (min)	V_d (55°C)	$X=V_d/V_T$	X_{model}	τ (min)	k (min^{-1})	RSS
0	0	0	0	**70.6409451**	**0.01299603**	**0.08611508**
3.69	10	0.01116071	0			
7.28	20	0.02232143	0			
15.21	30	0.03348214	0			
22.49	40	0.04464286	0			
28.27	50	0.05580357	0			
56.05	100	0.11160714	0			
73.98	150	0.16741071	0.04246641			
87.99	200	0.22321429	0.20185813			
99.07	250	0.27901786	0.30889602			
104.93	280	0.3125	0.35957403			
107.54	290	0.32366071	0.38093277			
109.5	300	0.33482143	0.39650265			
117.32	350	0.390625	0.45482183			
126.77	400	0.44642857	0.51782837			
132.85	450	0.50223214	0.55446124			
140.73	500	0.55803571	0.59782976			
148.54	550	0.61383929	0.6366464			
155.66	600	0.66964286	0.6687595			
162.52	650	0.72544643	0.6970124			
169.35	700	0.78125	0.72274741			
170.55	710	0.79241071	0.72703769			
172.07	720	0.80357143	0.73237687			
173.59	730	0.81473214	0.73761162			
175	740	0.82589286	0.74237594			

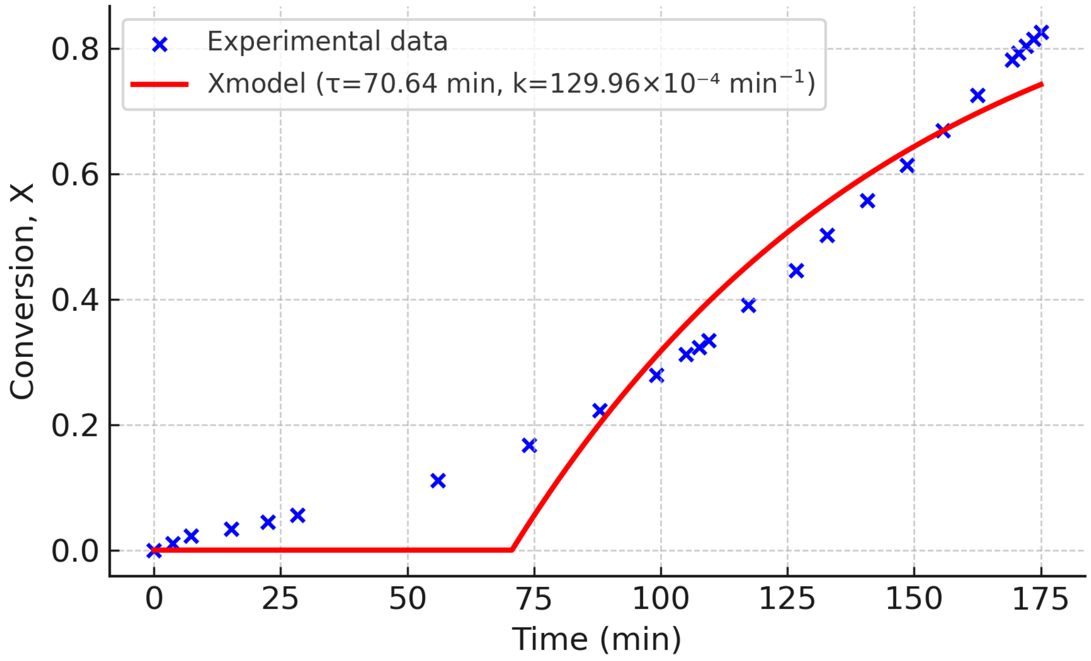

Fig. S.87: Experimental conversion data (blue points, from Table S.52 and Fig. S.83) and the fitted curve (red line) at **55 °C**, obtained using the X_{model}. Complex **1** was employed as the pre-catalyst in the first run.

Table S.53: Values obtained from Excel-Solver using X_{model} at **50°C**.

Time (min)	V_d (50°C)	$X=V_d/V_T$	X_{model}	τ (min)	k (min^{-1})	RSS
0	0	0	0	**20.0000074**	**0.00181531**	**0.00603177**
11.73	10	0.01116071	0			
18.77	20	0.02232143	0			
37.54	30	0.03348214	0.03133895			
51.62	40	0.04464286	0.05578377			
64.14	50	0.05580357	0.07700158			
107.94	100	0.11160714	0.14754796			
145.48	150	0.16741071	0.2037046			
173.64	200	0.22321429	0.2433876			
200.23	250	0.27901786	0.27904118			
213.52	280	0.3125	0.29622651			
216.65	290	0.32366071	0.30021396			
219	300	0.33482143	0.30319287			

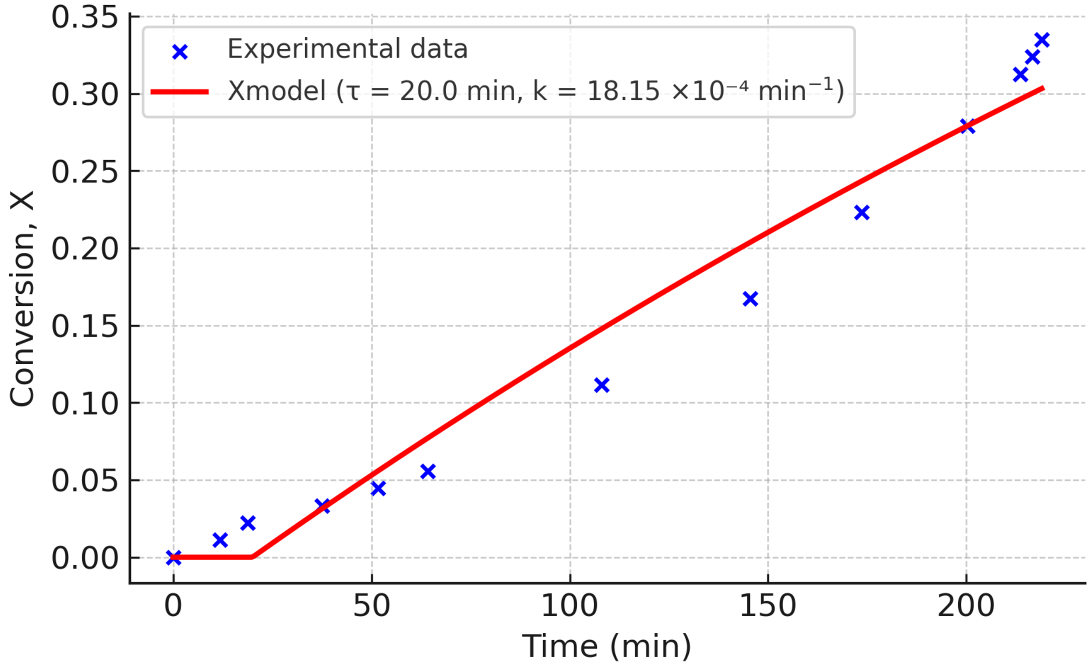

Fig. S.88: Experimental conversion data (blue points, from Table S.53 and Fig. S.83) and the fitted curve (red line) at **50 °C**, obtained using the X_{model}. Complex **1** was employed as the pre-catalyst in the first run.

Table S.54: Values obtained from Excel-Solver using X_{model} at **45°C**.

Time (min)	V_d (45°C)	$X=V_d/V_T$	X_{model}	τ (min)	k (min^{-1})	RSS
0	0	0	0	**0**	**0.00014478**	**8.6343E-06**
43.25	5	0.00558036	0.00624215			
72.71	10	0.01116071	0.01047171			
109.39	15	0.01674107	0.01571268			
142.8	20	0.02232143	0.02046227			
190.79	25	0.02790179	0.02724448			
203.51	26	0.02901786	0.02903426			
221.32	27	0.03013393	0.03153469			
241.67	30	0.03348214	0.03438385			

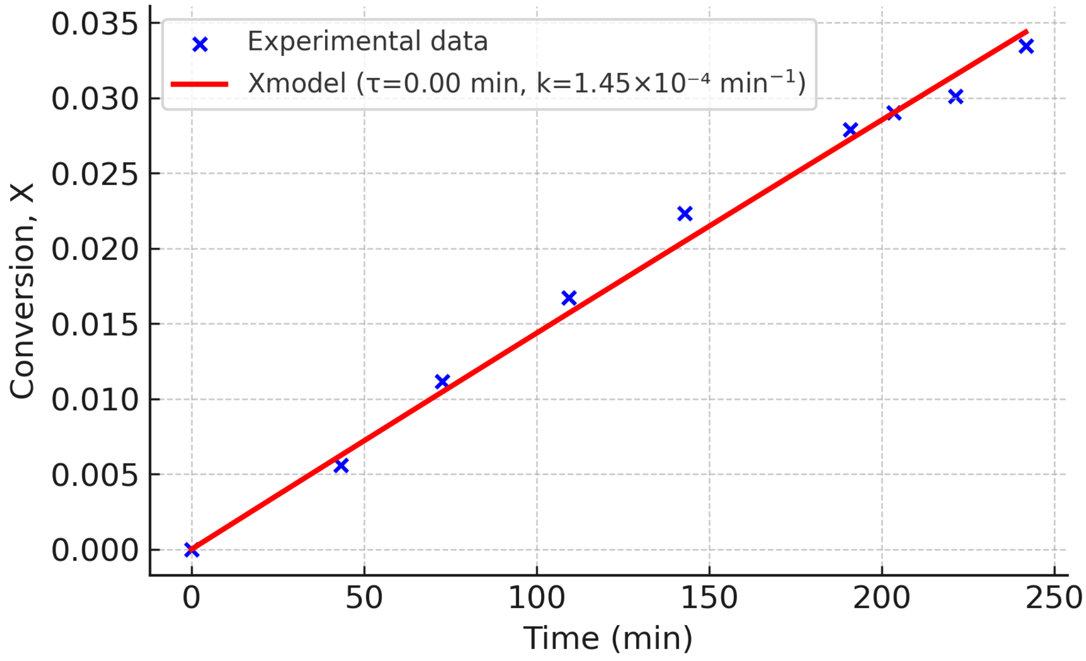

Fig. S.89: Experimental conversion data (blue points, from Table S.54 and Fig. S.83) and the fitted curve (red line) at **45 °C**, obtained using the X_{model}. Complex **1** was employed as the pre-catalyst in the first run.

Table S.55: Values obtained from Excel-Solver using X_{model} at **40°C**.

Time (min)	V_d (45°C)	$X=V_d/V_T$	X_{model}	τ (min)	k (min^{-1})	RSS
0	0	0	0	**25.8211504**	**5.0856E-05**	**7.8751E-06**
22.34	1	0.00111607	0			
38.05	2	0.00223214	0.00062172			
56.45	3	0.00334821	0.00155645			
110.45	4	0.00446429	0.00429464			
147.27	5	0.00558036	0.00615737			
169.36	6	0.00669643	0.00727323			
184.09	7	0.0078125	0.00801661			
198.82	8	0.00892857	0.00875944			
220.91	9	0.01004464	0.00987238			
243	10	0.01116071	0.01098408			

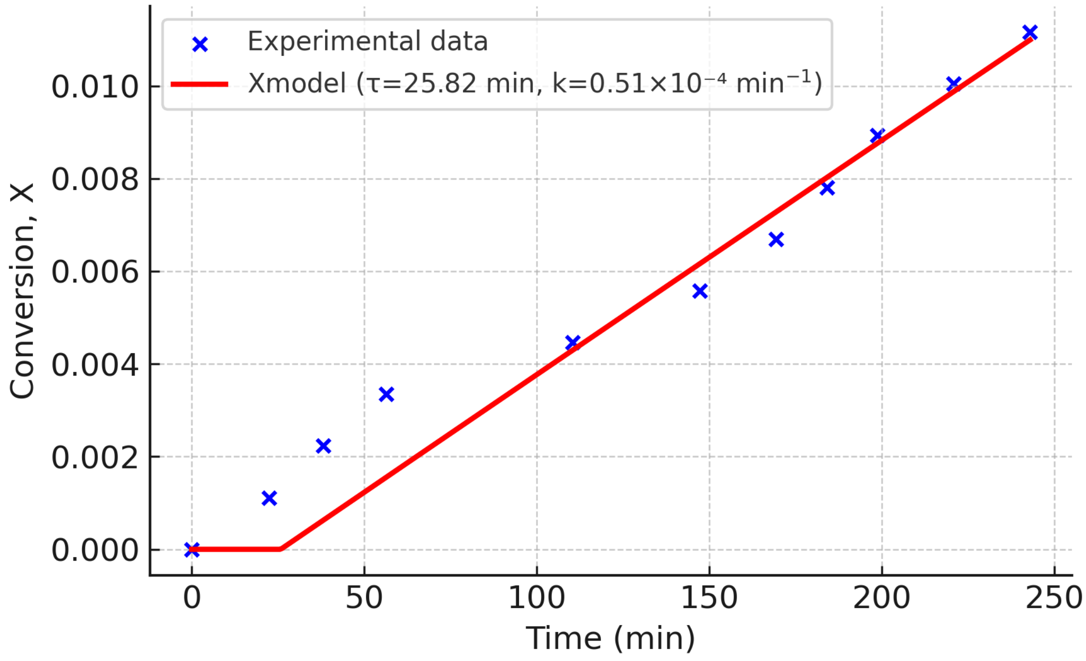

Fig. S.90: Experimental conversion data (blue points, from Table S.55 and Fig. S.83) and the fitted curve (red line) at **40 °C**, obtained using the X_{model}. Complex **1** was employed as the pre-catalyst in the first run.

Comparative analysis of end-point and X_{model} approaches.

The end-point method provides a simple and effective way to estimate apparent rate constants from the final conversion values. In the present system, its use is justified because the first runs consistently displayed induction periods, which represent the activation of the pre-catalyst into the active catalytic species for formic acid dehydrogenation. The X_{model} approach explicitly accounts for this induction time, yielding parameters (τ, k) with a clearer mechanistic interpretation.

Tabel S.56: Comparison of k_{obs} values obtained by the endpoint method with k values obtained by X_{model}, including induction time and RSS for the temperature range 40 – 60°C.

Temp. (°C)	Average k_{obs} ($\times 10^{-4}$ min^{-1})	k (X_{model}) ($\times 10^{-4}$ min^{-1})	*τ (min)*	RSS
40	0.46	0.51	25.82	$7.87E^{-06}$
45	1.25	1.45	0.00	$8.63E^{-06}$
50	17.99	18.15	20.00	0.0060
55	94.81	129.96	70.64	0.0861
60	274.32	283.64	44.54	0.1442

Across 40 – 60 °C, k values obtained from the X_{model} are in close agreement with k_{obs} from the end-point method, with the largest deviation observed at 55 °C where induction times are longest. Induction periods range from ca. 26 min at 40 °C to over 70 min at 55 °C, highlighting their relevance in the activation of the pre-catalyst. RSS values

remain low at lower temperatures, indicating excellent fits, and increase at higher temperatures, consistent with greater variability among replicates. This close agreement indicates that the end-point method remains reliable, while the induction–growth model offers additional insight into catalyst activation and kinetic behavior.

Comparison of the activation parameters using rate constants from both methods

Table S.57: Arrhenius / Eyring input using k from X_{model}.

Temp. (°C)	T (K)	$k_{(X\text{model})} \times 10^{-4}$ min^{-1}	k (s^{-1})	1/T (K^{-1})	ln$k$ (min^{-1})	ln(k/T) (s^{-1} K^{-1})
40	313.15	0.51	9E-07	0.003193	-9.883685	-19.724712
45	318.15	1.45	2.4e-06	0.003143	-8.838777	-18.695644
50	323.15	18.15	3.02e-05	0.003095	-6.31167	-16.184131
55	328.15	129.96	0.0002166	0.003047	-4.343114	-14.230929
60	333.15	283.64	0.0004727	0.003002	-3.562635	-13.465572

Table S.58: Active parameters obtained from both methods.

Method	E_a (kcal·mol^{-1})	$\Delta H^‡$ (kcal·mol^{-1})	$\Delta S^‡$ (cal·mol^{-1}·K^{-1})	$\Delta G^‡$ (333 K) (kcal·mol^{-1})	R^2
End-point	70.9	70.3	+137	24.5	0.977
X_{model}	71.1	70.5	+138	24.4	0.974

The activation parameters derived from both the end-point method and the induction–growth kinetic model (X_{model}) is highly consistent across the 40 – 60 °C temperature range. Both approaches yield high correlation coefficients ($R^2 \approx 0.97$), confirming the reliability of the kinetic treatment.

Although the X_{model} provides additional mechanistic detail by explicitly separating the induction time (τ) from the pseudo–first-order growth constant (k), the end-point method offers a more straightforward treatment. In the present study, the end-point approach was chosen for the main text because it delivers apparent rate constants that inherently account for the induction period, while avoiding overinterpretation of datasets where τ cannot be precisely defined at all temperatures. The excellent agreement between the activation parameters obtained by both methods validates the robustness of the end-point treatment and supports its inclusion in the main manuscript.
